# Supplementary material for: The effector-triggered immunity landscape of tomato against Pseudomonas syringae
Source: Nat Commun. 2024 Jun 14;15:5102. doi: 10.1038/s41467-024-49425-4 (PMC11178782; doi:10.1038/s41467-024-49425-4)
Supplement: Supplementary file 1 — Supplementary Information [file 41467_2024_49425_MOESM1_ESM.pdf]

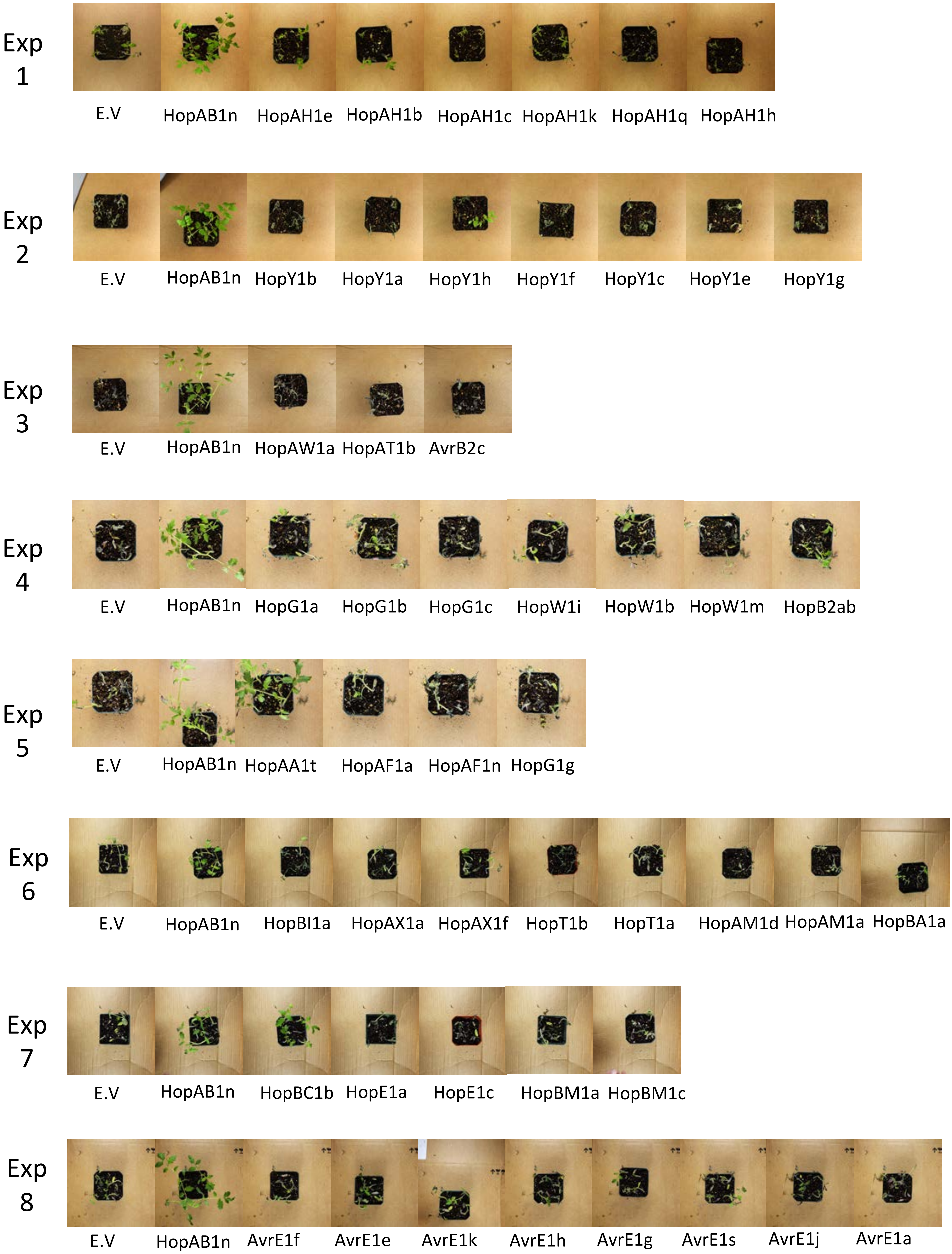
Exp 1
E.V
HopAB1n
HopAH1e
HopAH1b
HopAH1c
HopAH1k
HopAH1q
HopAH1h
Exp 2
E.V
HopAB1n
HopY1b
HopY1a
HopY1h
HopY1f
HopY1c
HopY1e
HopY1g
Exp 3
E.V
HopAB1n
HopAW1a
HopAT1b
AvrB2c
Exp 4
E.V
HopAB1n
HopG1a
HopG1b
HopG1c
HopW1i
HopW1b
HopW1m
HopB2ab
Exp 5
E.V
HopAB1n
HopAA1t
HopAF1a
HopAF1n
HopG1g
Exp 6
E.V
HopAB1n
HopBI1a
HopAX1a
HopAX1f
HopT1b
HopT1a
HopAM1d
HopAM1a
HopBA1a
Exp 7
E.V
HopAB1n
HopBC1b
HopE1a
HopE1c
HopBM1a
HopBM1c
Exp 8
E.V
HopAB1n
AvrE1f
AvrE1e
AvrE1k
AvrE1h
AvrE1g
AvrE1s
AvrE1j
AvrE1a

Exp 9

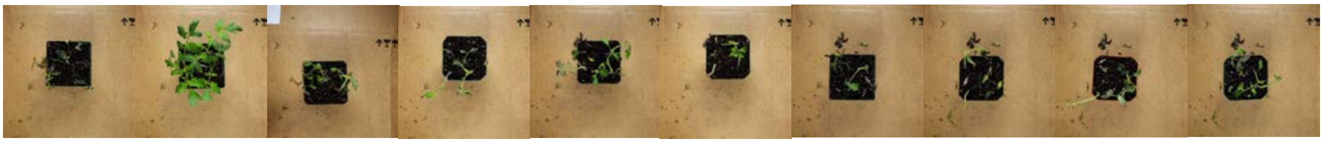

E.V HopAB1n AvrE1p AvrE1i AvrE1aa AvrE1b AvrE1c AvrE1l AvrE1m AvrE1n

Exp 10

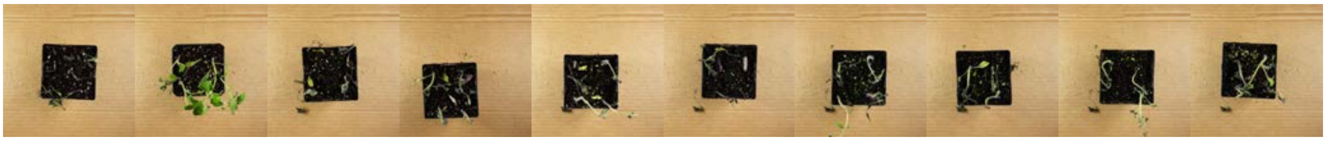

E.V HopAB1n AvrE1o AvrE1q AvrE1x AvrE1y AvrE1z HopBN1a HopBN1b HopBN1c

Exp 11

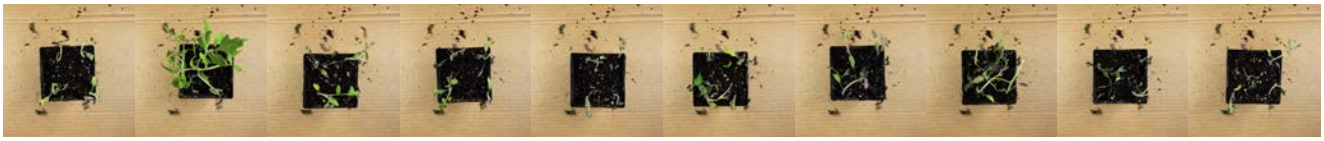

E.V HopAB1n HopL1a HopAU1a HopAU1b HopBN1d HopBN1e HopBN1f HopBN1g HopBN1h

Exp 12

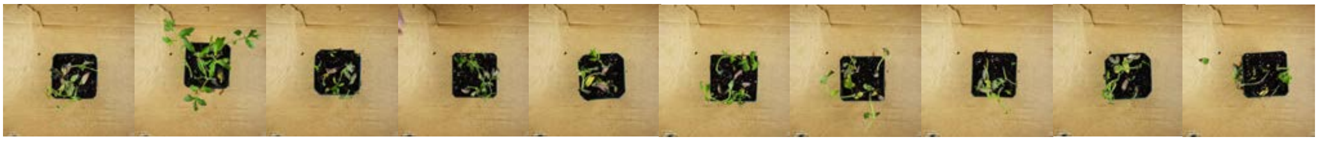

E.V HopAB1n HopR1a HopR1b HopR1f HopR1c HopR1g HopAZ1a HopAZ1i HopAZ1c

Exp 13

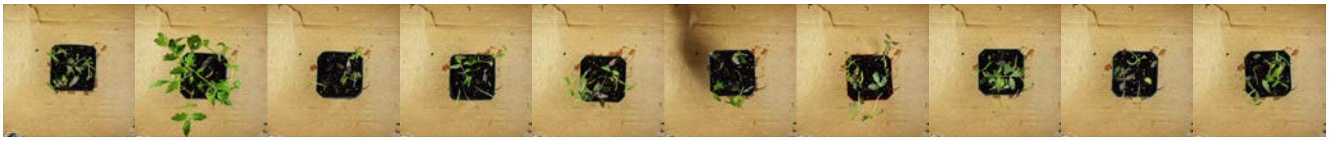

E.V HopAB1n HopAZ1p HopAZ1h HopAZ1d HopAZ1e HopAZ1n HopAZ1s HopR1e HopR1h

Exp 14

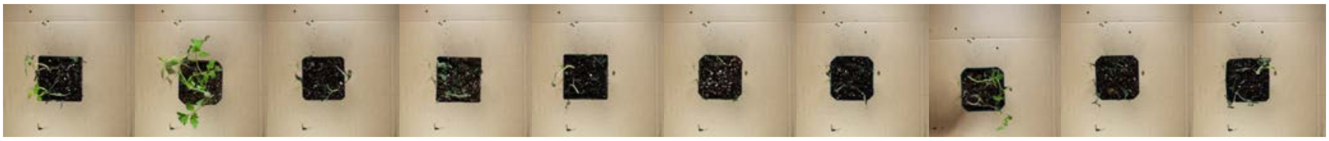

E.V HopAB1n HopB1d HopB1b HopQ1b HopQ1a HopC1c HopC1d HopC1a HopC1b

Exp 15

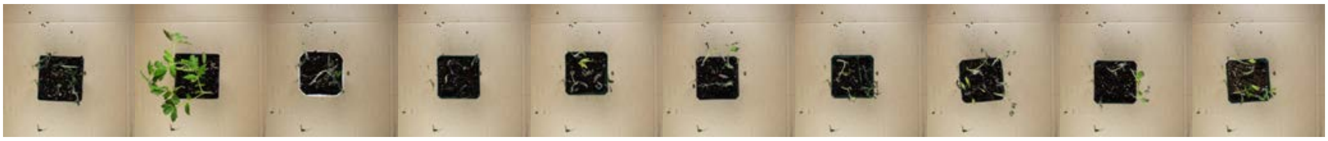

E.V HopAB1n HopBL1a HopBL1b HopH1d HopH1e HopBM1a HopBM1b HopD1b HopQ1d

Exp 16

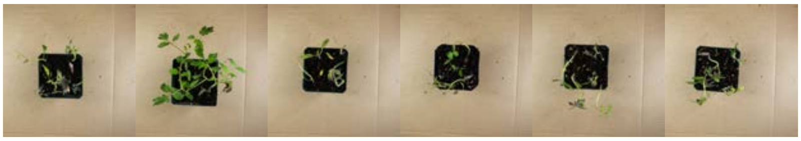

E.V HopAB1n HopW1a HopW1n HopW1c HopW1e

Exp 17

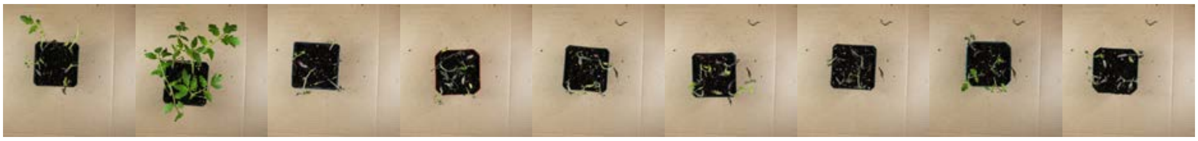

E.V HopAB1n HopW1h HopW1f HopW1g HopW1l HopW1s HopW1u HopW1j

Exp 18

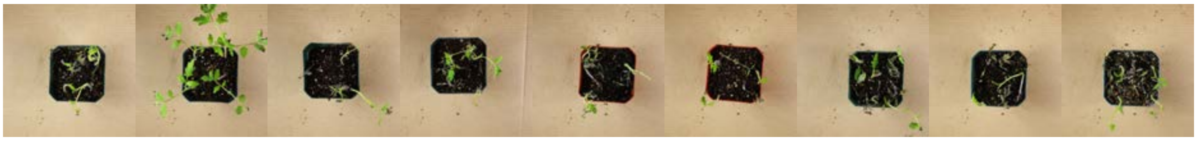

E.V HopAB1n HopBD1b HopBD1a HopAD1a AvrB2f HopBD1c HopBD1f AvrB1b

Exp 19

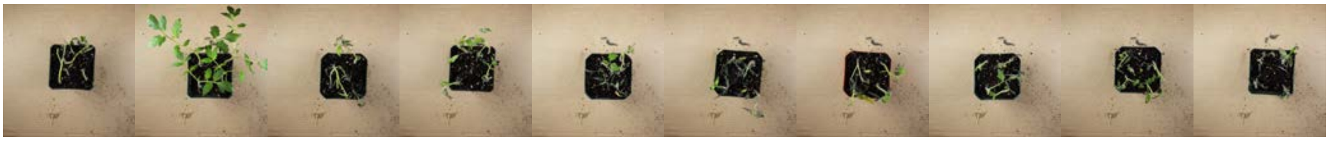

E.V HopAB1n HopO2e HopO2a HopO2d HopO2b HopO1f HopO1c HopO1a HopO1b

Exp 20

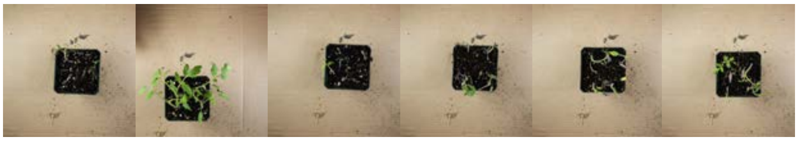

E.V HopAB1n HopO1d HopM1v HopM1d HopO1g

Exp 21

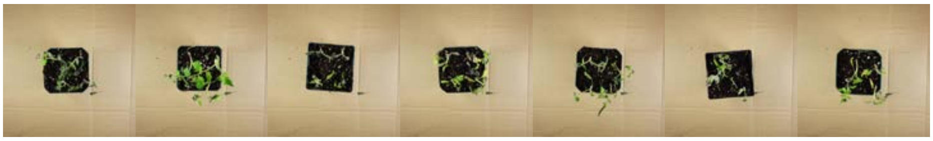

E.V HopAB1n HopAG1m HopAG1g HopAG1d HopAG1c HopAG1l

Exp 22

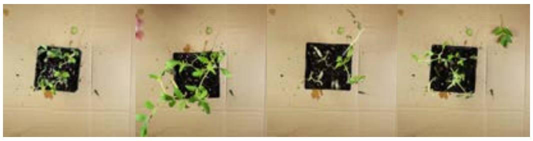

E.V HopAB1n HopV1k HopV1o

Exp 23

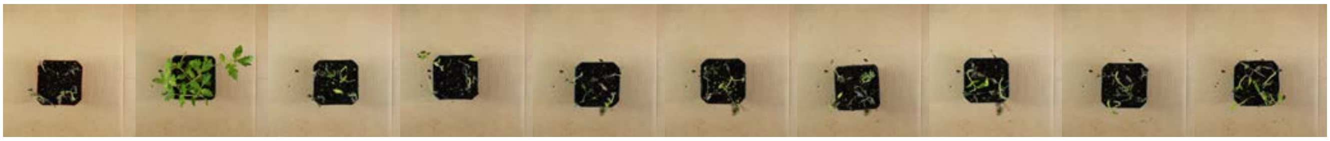

E.V HopAB1n HopF1e HopF1a HopF1q HopF1d HopF1p HopF1c HopF1i HopF1m

Exp 24

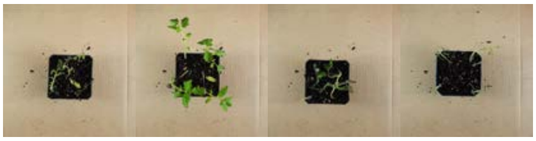

E.V HopAB1n HopF1g HopF1j

Exp 25

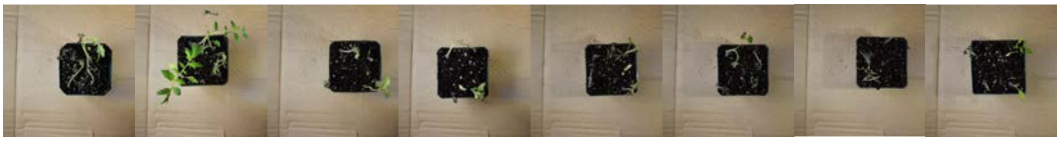

E.V | HopAB1n | AvrB1a | AvrB2g | AvrB2e | AvrB2a | AvrB2b | HopBD1d

Exp 26

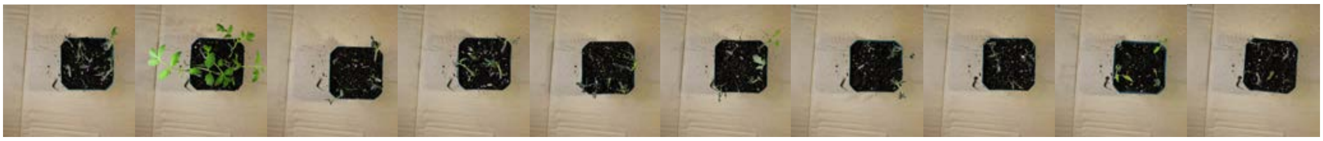

E.V | HopAB1n | HopBG1a | HopF1l | HopF1r | HopF3a | HopF1b | HopF1h | HopF1s | HopF3c

Exp 27

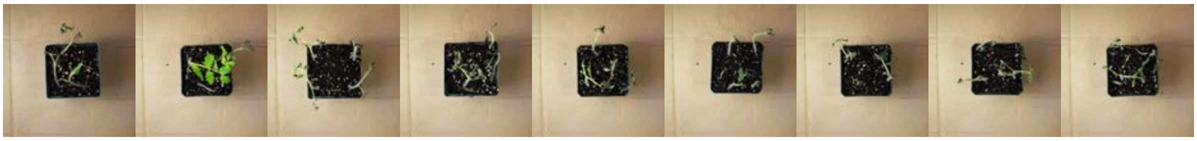

E.V | HopAB1n | HopBD1g | HopBD1j | HopA1g | HopA1f | HopA1i | HopA1j | HopA1k

Exp 28

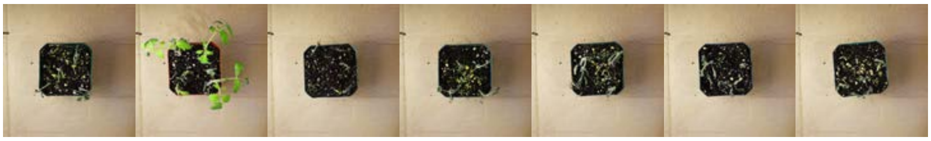

E.V | HopAB1n | HopM1af | HopV1g | HopV1d | HopV1n | HopN1a

Exp 29

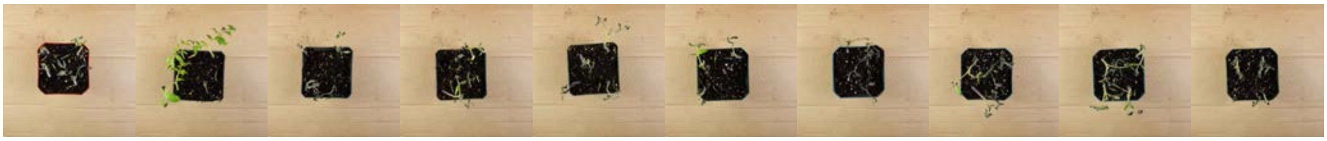

E.V | HopAB1n | AvrPto1f | AvrPto1d | AvrPto1m | AvrPto1k | AvrPto1e | AvrPto1i | AvrPto1h | AvrPto1c

Exp 30

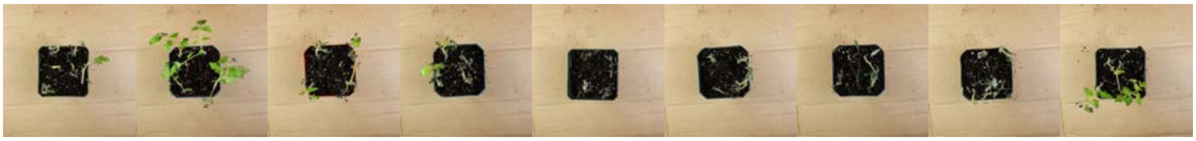

E.V | HopAB1n | AvrPto1b | HopA1d | HopA1b | HopA1l | HopA1c | HopA1a | HopBC1a

Exp 31

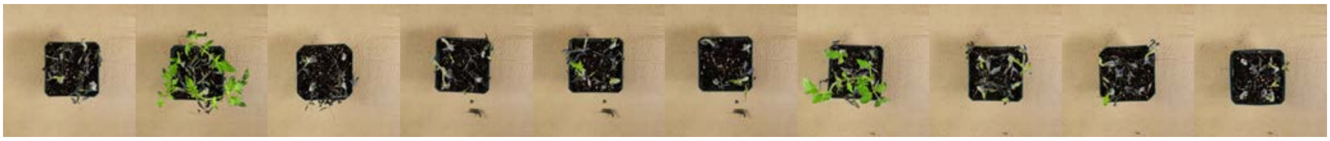

E.V | HopAB1n | HopB1c | HopB1e | HopB2e | HopB4a | HopBF1b | HopBH1b | HopBH1d | HopBP1e

Exp 32

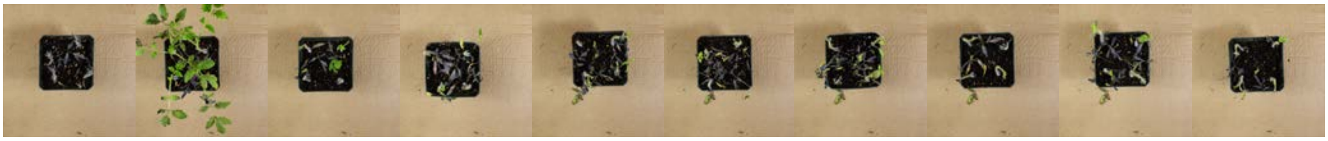

E.V | HopAB1n | HopBR1a | HopF1v | HopI1m | HopN1b | HopO1e | HopT1c | HopT1e | HopV1a

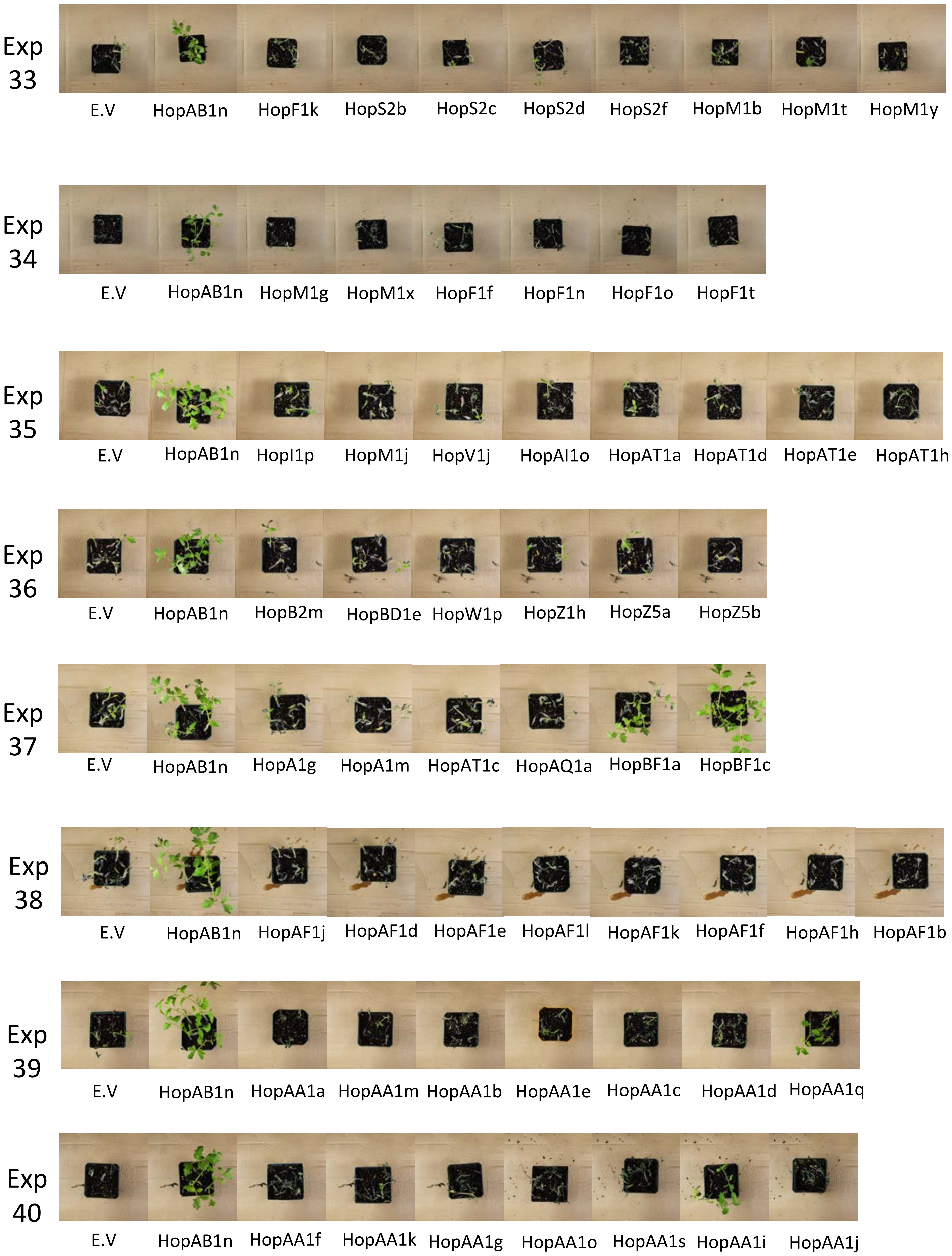

Exp 33
E.V
HopAB1n
HopF1k
HopS2b
HopS2c
HopS2d
HopS2f
HopM1b
HopM1t
HopM1y
Exp 34
E.V
HopAB1n
HopM1g
HopM1x
HopF1f
HopF1n
HopF1o
HopF1t
Exp 35
E.V
HopAB1n
HopI1p
HopM1j
HopV1j
HopAI1o
HopAT1a
HopAT1d
HopAT1e
HopAT1h
Exp 36
E.V
HopAB1n
HopB2m
HopBD1e
HopW1p
HopZ1h
HopZ5a
HopZ5b
Exp 37
E.V
HopAB1n
HopA1g
HopA1m
HopAT1c
HopAQ1a
HopBF1a
HopBF1c
Exp 38
E.V
HopAB1n
HopAF1j
HopAF1d
HopAF1e
HopAF1l
HopAF1k
HopAF1f
HopAF1h
HopAF1b
Exp 39
E.V
HopAB1n
HopAA1a
HopAA1m
HopAA1b
HopAA1e
HopAA1c
HopAA1d
HopAA1q
Exp 40
E.V
HopAB1n
HopAA1f
HopAA1k
HopAA1g
HopAA1o
HopAA1s
HopAA1i
HopAA1j

Exp 41

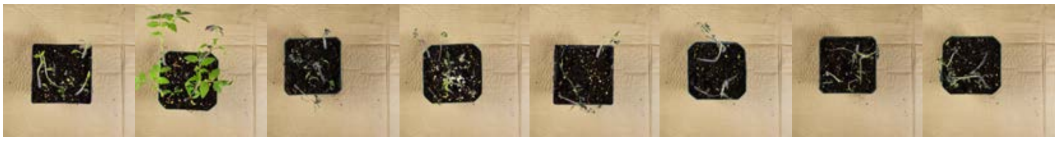

E.V HopAB1n HopX1b HopX1c HopX1i HopX1e HopX1d HopX1g

Exp 42

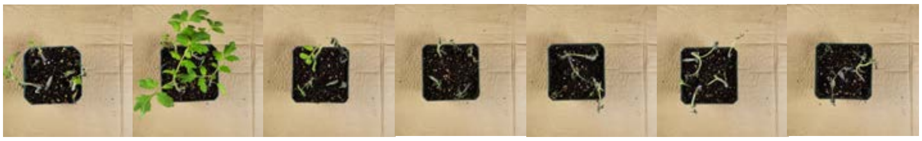

E.V HopAB1n HopF4a AvrRpm1a AvrRpm1h AvrRpm1d AvrRpm1e

Exp 43

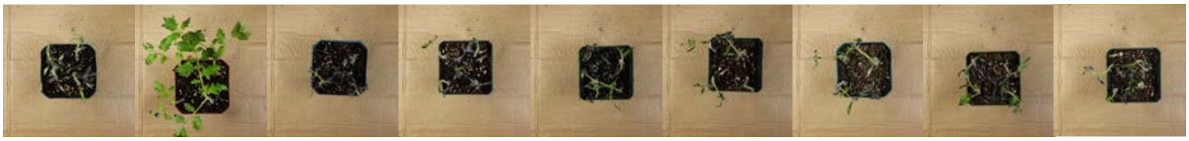

E.V HopAB1n AvrRpm1f AvrRpm1i HopAB1i HopK1d HopK1e HopK1a HopK1b

Exp 44

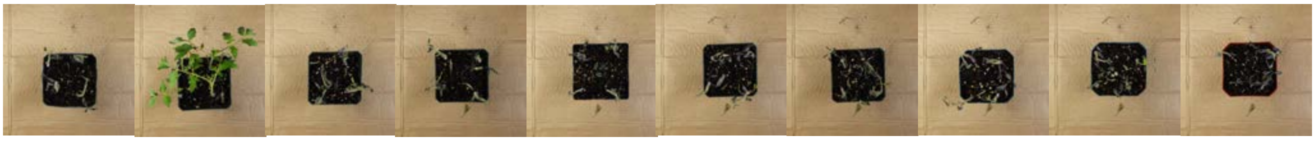

E.V HopAB1n HopAA1l HopAA1n HopAA1p HopAA1r HopAA1v HopAH1aa HopB2x HopK1c

Exp 45

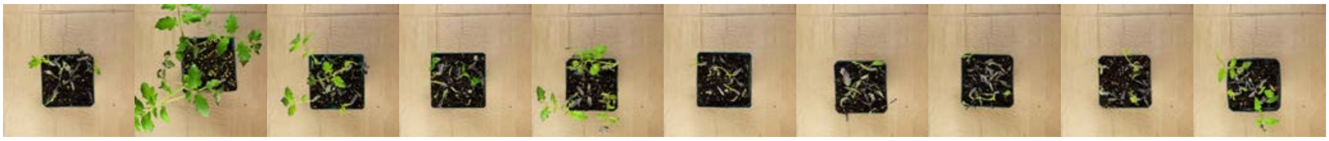

E.V HopAB1n HopAB1r HopAB1s HopAB1j HopAB1g HopAB1d HopAB1a HopAB1o HopAB1p

Exp 46

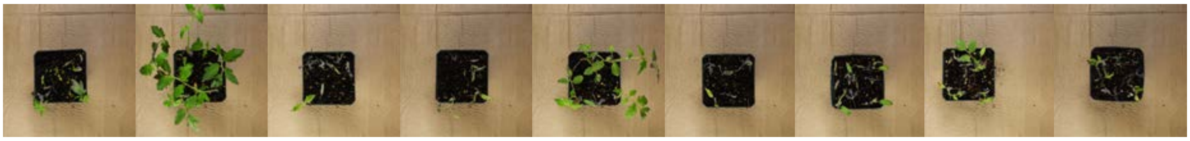

E.V HopAB1n HopAB1e HopAB1aa HopAB1ab HopAB1k HopAB1l HopAB1q HopAB1u

Exp 47

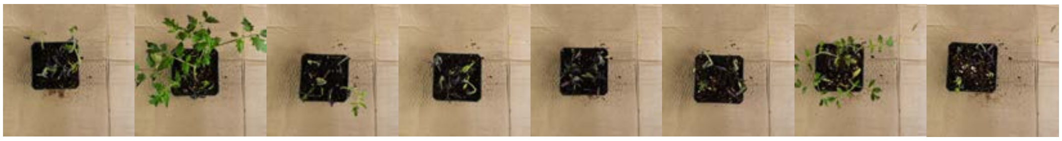

E.V HopAB1n HopU1a HopI1j HopI1f HopI1h HopBJ1b HopS2e

Exp 48

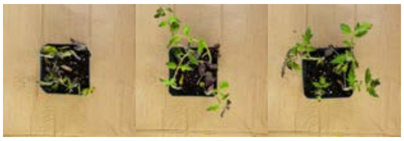

E.V HopAB1n HopBJ1a

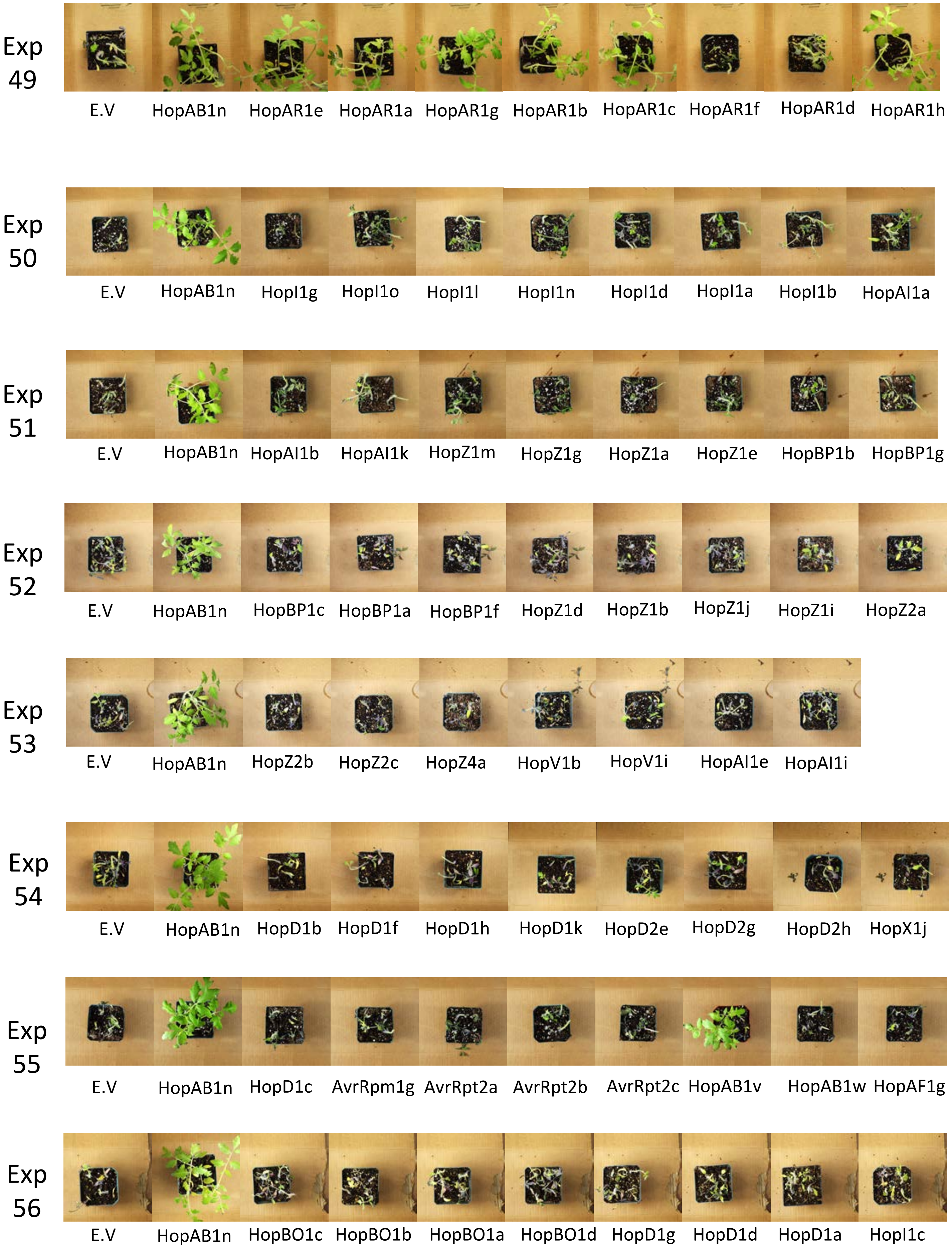
Exp 49
E.V HopAB1n HopAR1e HopAR1a HopAR1g HopAR1b HopAR1c HopAR1f HopAR1d HopAR1h
Exp 50
E.V HopAB1n HopI1g HopI1o HopI1l HopI1n HopI1d HopI1a HopI1b HopAI1a
Exp 51
E.V HopAB1n HopAI1b HopAI1k HopZ1m HopZ1g HopZ1a HopZ1e HopBP1b HopBP1g
Exp 52
E.V HopAB1n HopBP1c HopBP1a HopBP1f HopZ1d HopZ1b HopZ1j HopZ1i HopZ2a
Exp 53
E.V HopAB1n HopZ2b HopZ2c HopZ4a HopV1b HopV1i HopAI1e HopAI1i
Exp 54
E.V HopAB1n HopD1b HopD1f HopD1h HopD1k HopD2e HopD2g HopD2h HopX1j
Exp 55
E.V HopAB1n HopD1c AvrRpm1g AvrRpt2a AvrRpt2b AvrRpt2c HopAB1v HopAB1w HopAF1g
Exp 56
E.V HopAB1n HopBO1c HopBO1b HopBO1a HopBO1d HopD1g HopD1d HopD1a HopI1c

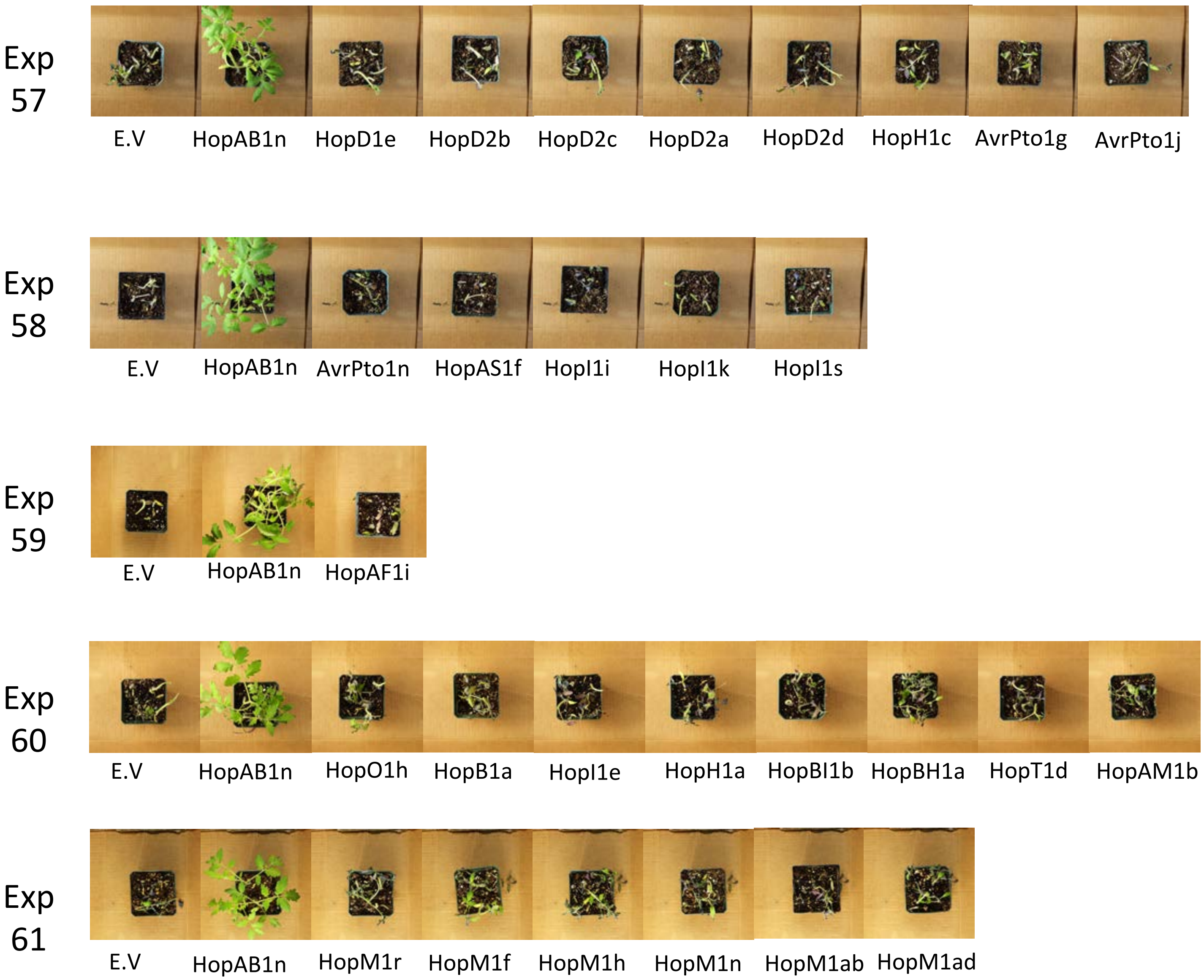

Supplementary Figure 1.Compiled images of primary ETI screening of PsyTEC in tomato Glamour. Images of tomato plants spray inoculated with PtoDC3000 expressing representative PsyTEC effector alleles 7 days post infection that were used to determine the Disease score presented in Fig 2. Effector allele names are presented above the images. Each experiment included a positive ETI control, HopAB1n, and a negative control, Empty Vector. Experiments were repeated at least twice with similar results.

Supplementary Figure 2

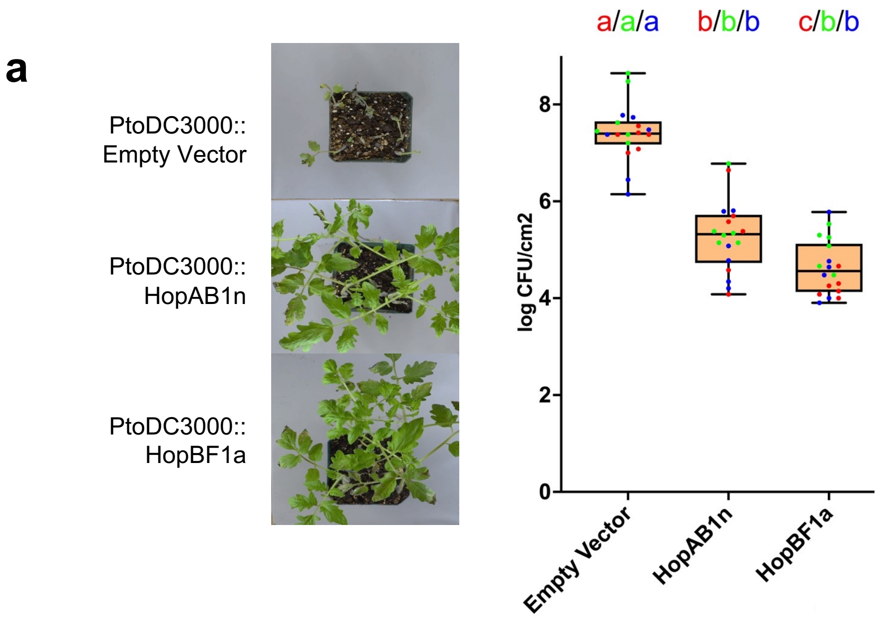

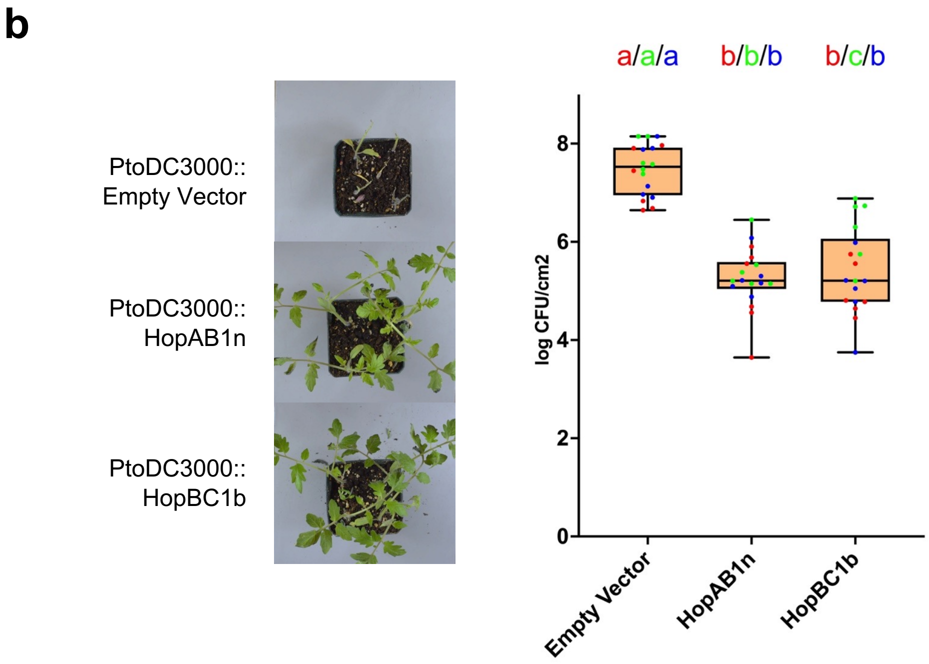

Supplementary Figure 2. Validation of HopBF1 and HopBC1 ETI by growth assays. Bacterial growth assays were conducted in tomato plants using PtoDC3000 carrying **(a)** HopBF1a and **(b)** HopBC1b alleles, which both trigger an ETI in tomato var. Glamour but not in *A. thaliana*. The empty vector strain was used as a control for disease, and the HopAB1n allele served as a control for ETI elicitation. Each dot represents a plant, with dots of the same color indicating plants from the same replicate. Box plots display pooled data from three replicates (n=6 individual plants per replicate) with error bars representing SEM. The boxes show the first quartile, median, and third quartile, with whiskers extending to the smallest and largest values. Letters are used to indicate groups following ANOVA post-hoc Tukey-test (P < 0.05). The color of the letter indicates the statistical group of the same color replicates. The left panels show representative pictures taken 7 days post-inoculation.

Supplementary Figure 3

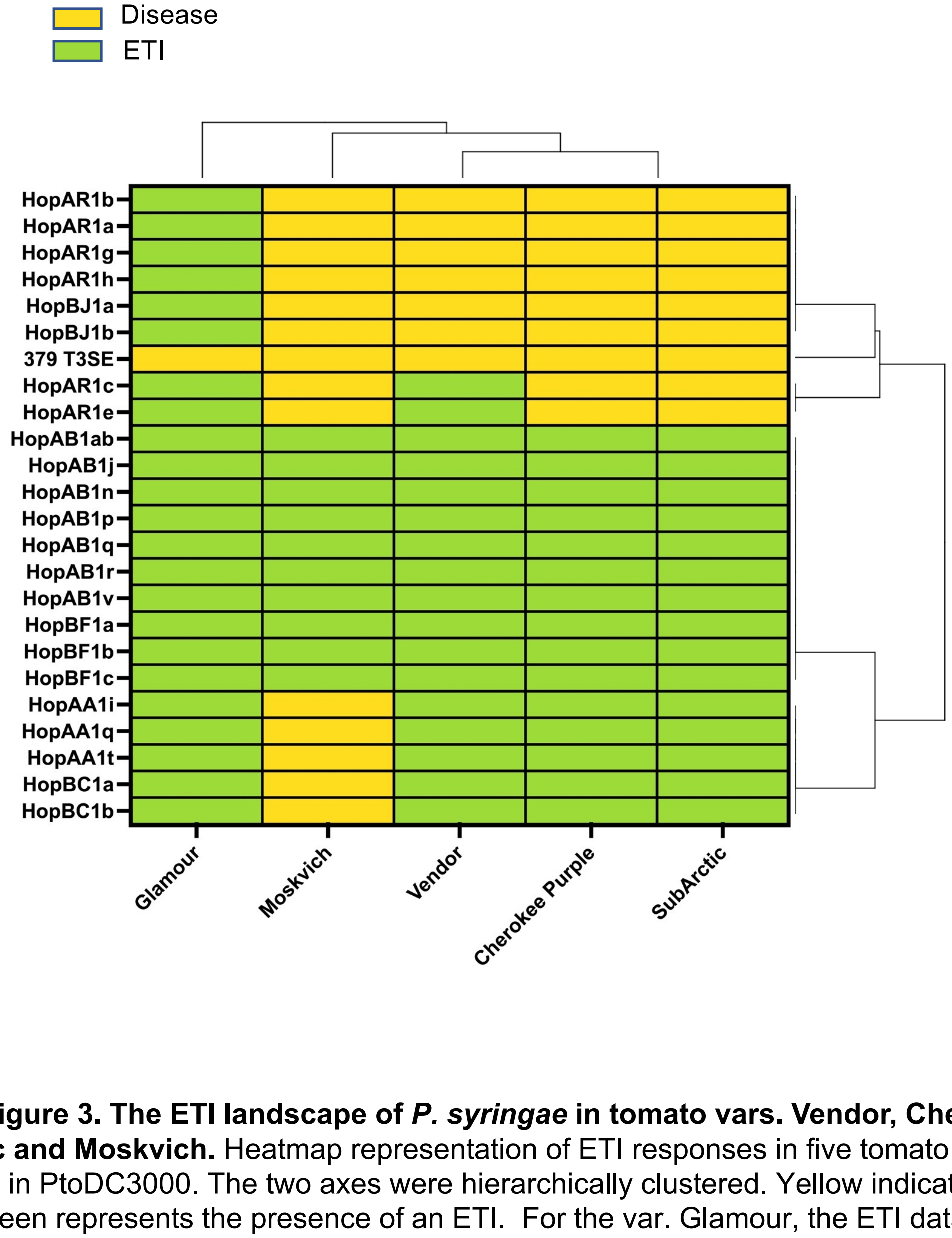

Supplementary Figure 3. The ETI landscape of *P. syringae* in tomato vars. Vendor, Cherokee Purple, SubArctic and Moskvich. Heatmap representation of ETI responses in five tomato varieties for effector expressed in PtoDC3000. The two axes were hierarchically clustered. Yellow indicates the absence of ETI, green represents the presence of an ETI. For the var. Glamour, the ETI data is based on PIDIQ data from Fig. 2 and Supplementary Fig. 1. ETI data for vars. Vendor, Cherokee Purple, SubArctic, and Moskvich are based on visual phenotypes presented in Supplementary Fig. 4.

HopAB1n | E.V. | HopX1c | HopX1b | HopX1i | HopX1e

Exp 1

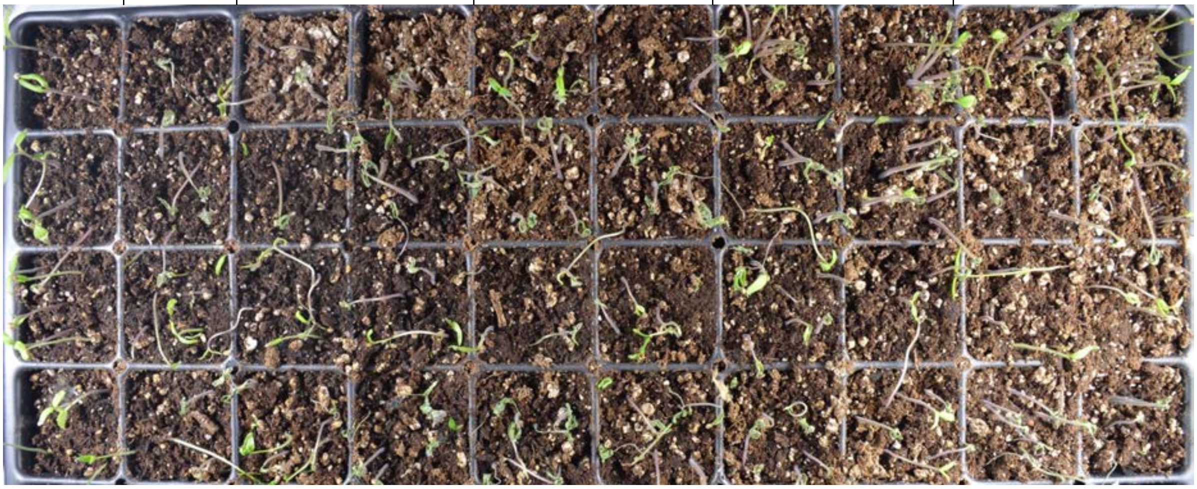

HopAB1n | E.V. | HopX1d | HopX1g | HopF4a | HopX1j

Exp 2

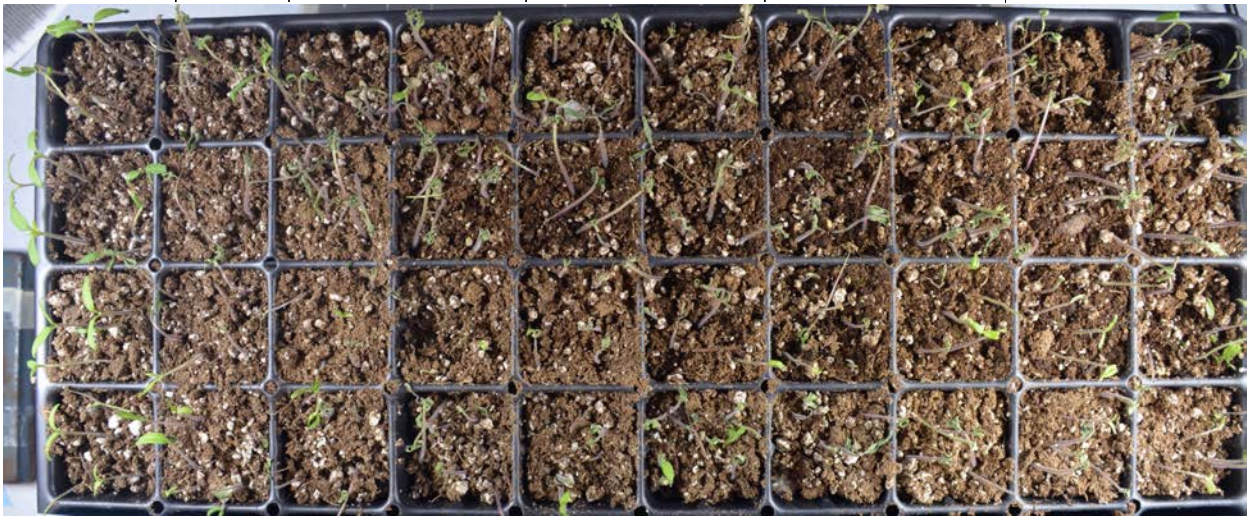

HopAB1n | E.V. | HopO1h | HopO2e | HopO2a | XXXXXXXX

Exp 3

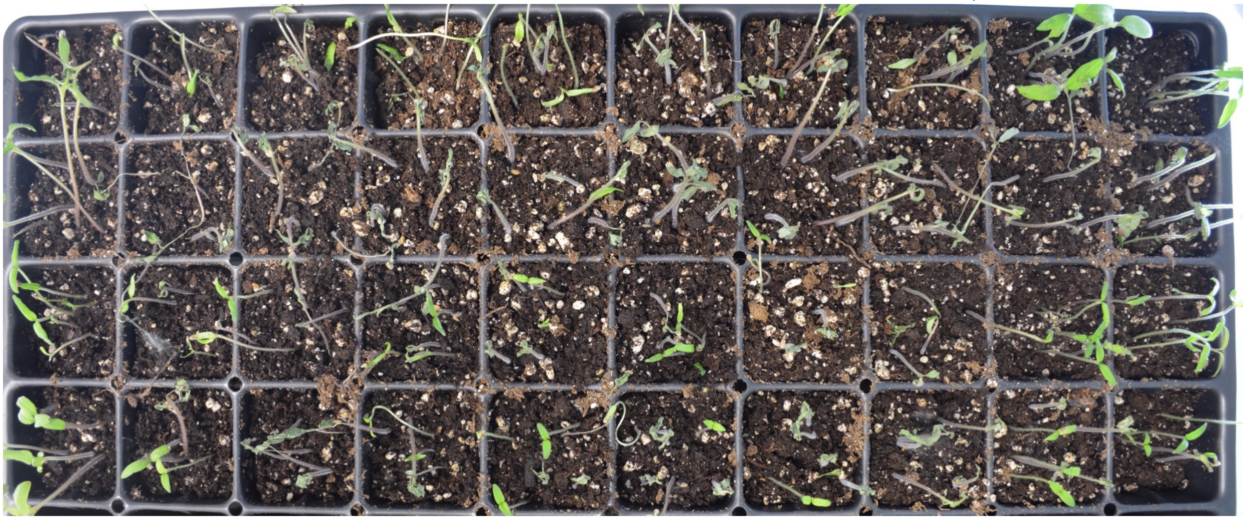

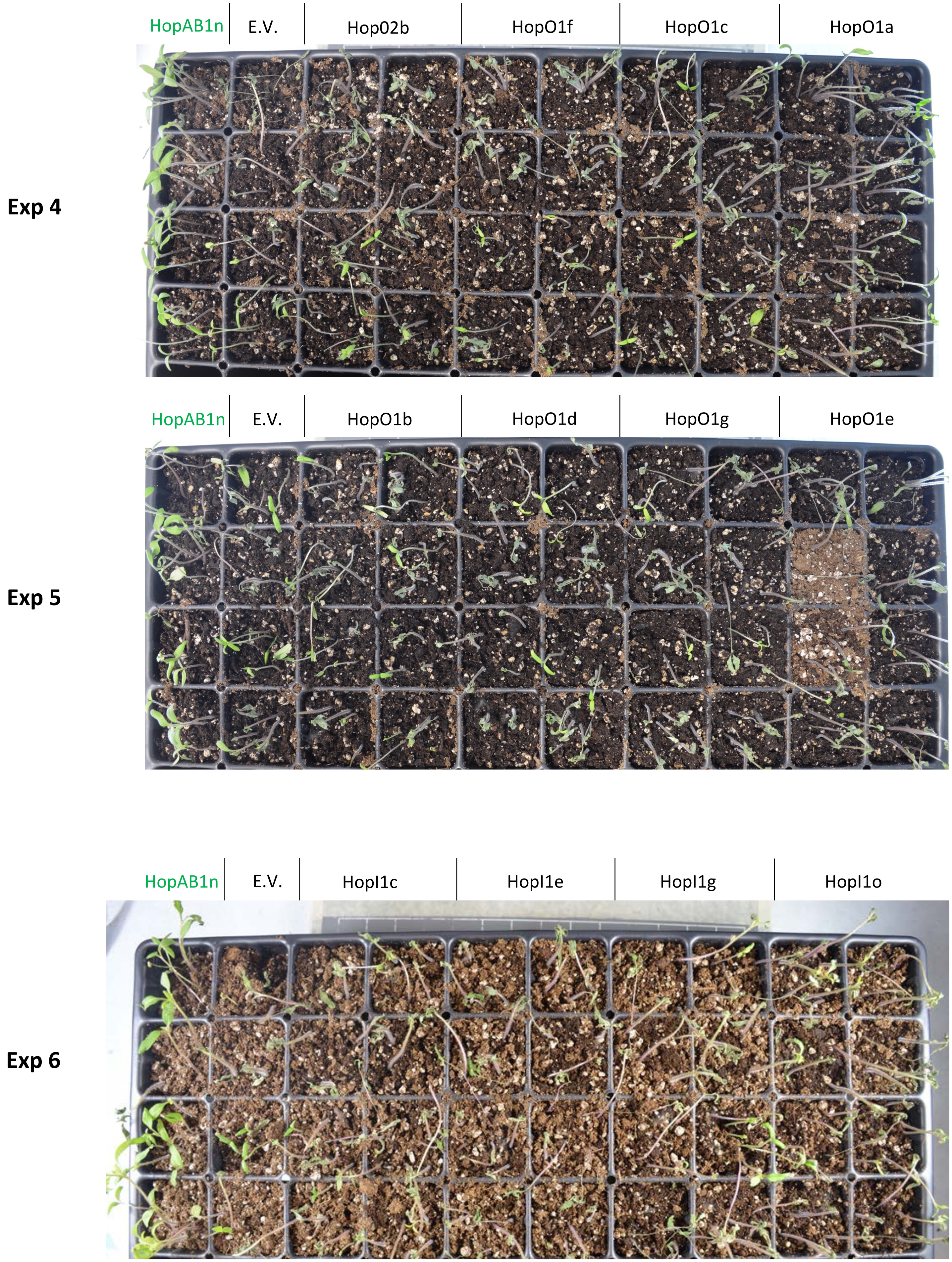
Exp 4
HopAB1n
E.V.
Hop02b
HopO1f
HopO1c
HopO1a
Exp 5
HopAB1n
E.V.
HopO1b
HopO1d
HopO1g
HopO1e
Exp 6
HopAB1n
E.V.
HopI1c
HopI1e
HopI1g
HopI1o

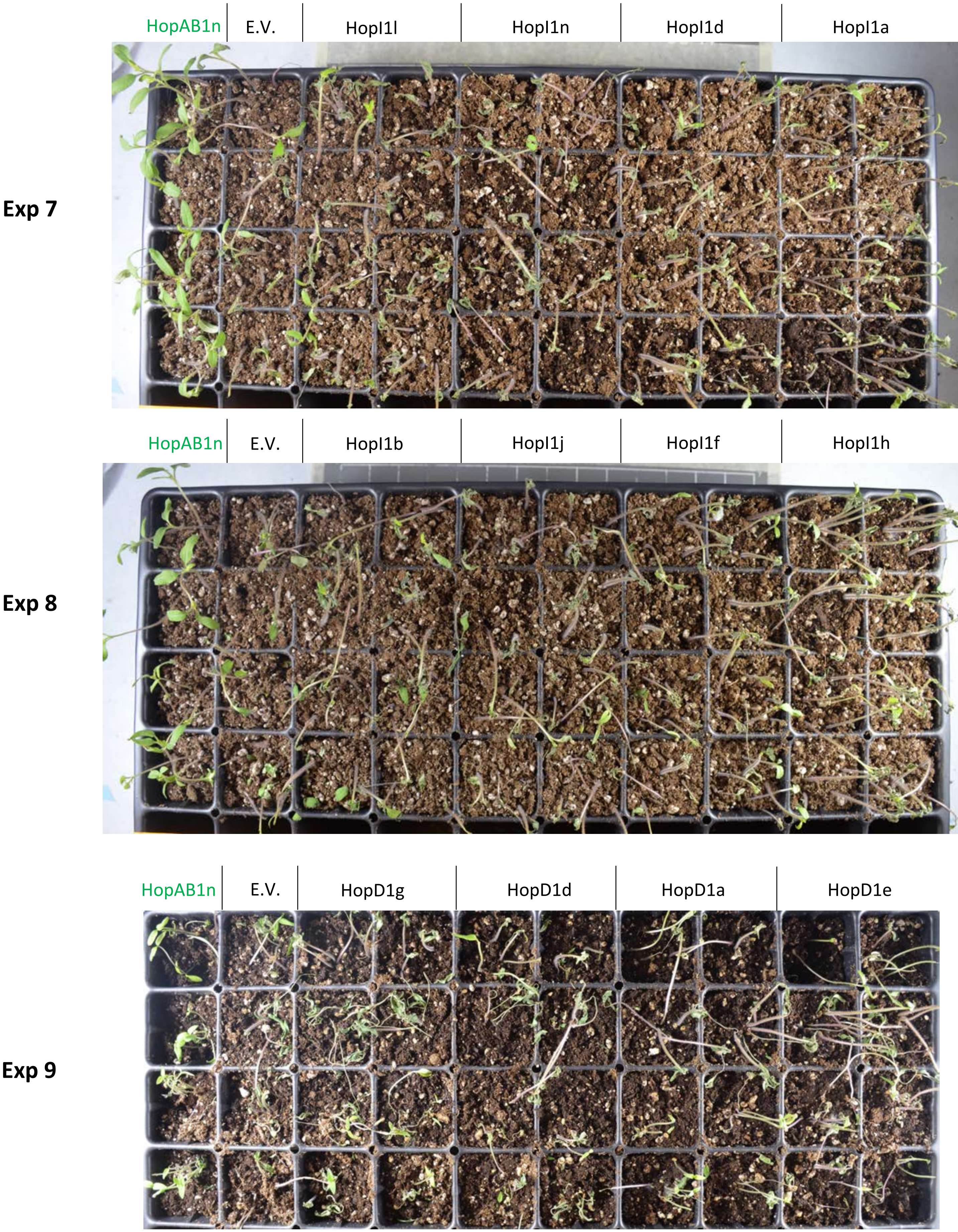

HopAB1n
E.V.
HopI1l
HopI1n
HopI1d
HopI1a
Exp 7
HopAB1n
E.V.
HopI1b
HopI1j
HopI1f
HopI1h
Exp 8
HopAB1n
E.V.
HopD1g
HopD1d
HopD1a
HopD1e
Exp 9

Exp 10

HopAB1n | E.V. | HopD1c | HopD2b | HopD2c | HopD2a

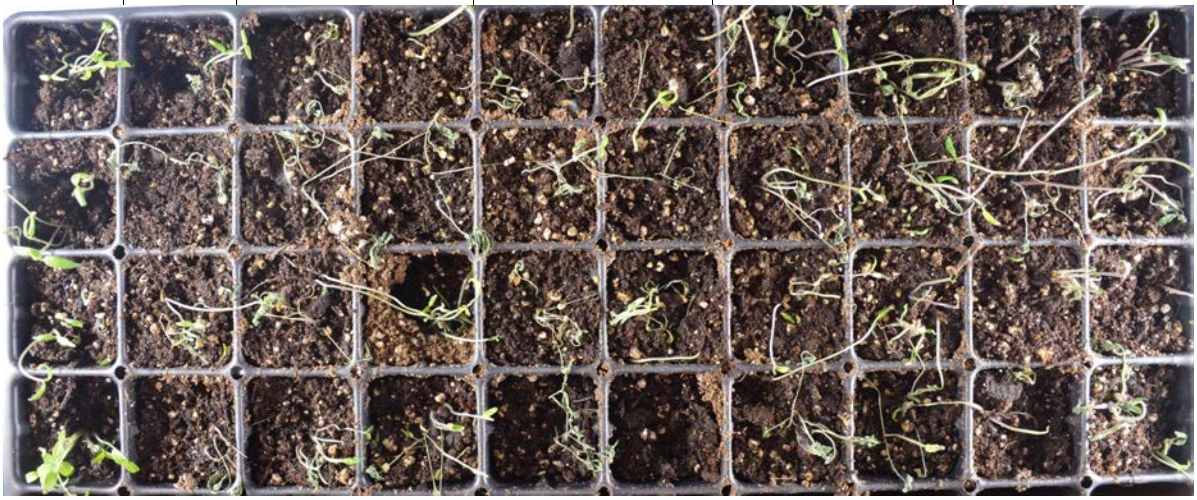

Exp 11

HopAB1n | E.V. | HopBO1c | HopBO1b | HopBO1a | HopBO1d

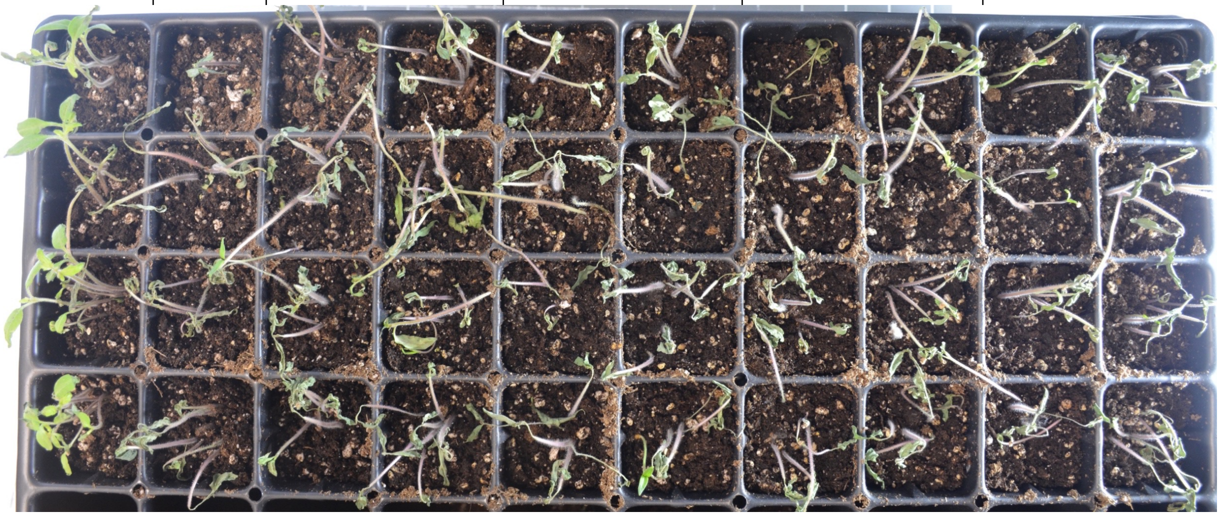

Exp 12

HopAB1n | E.V. | AvrRpm1a | AvrRpm1h | AvrRpm1d | AvrRpm1e

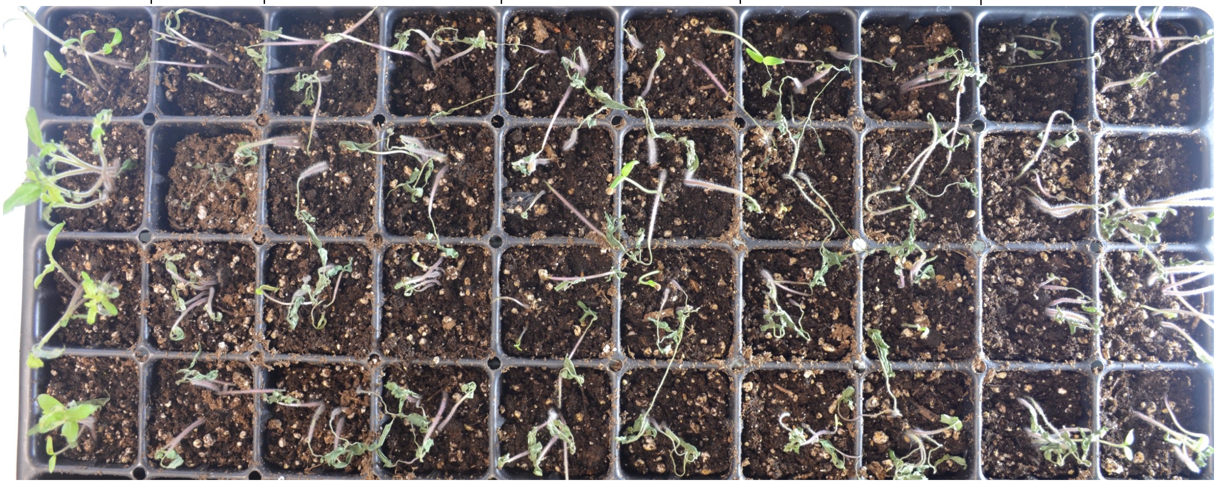

HopAB1n | E.V. | AvrRpm1f | AvrRpm1i | HopU1a

Exp 13

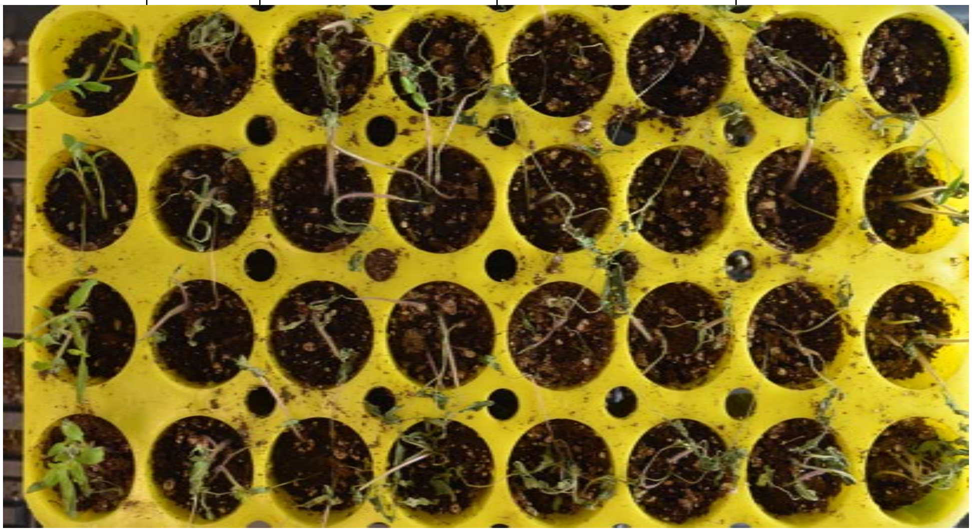

HopAB1n | E.V. | HopI1p | HopG1a | HopG1b | HopG1c

Exp 14

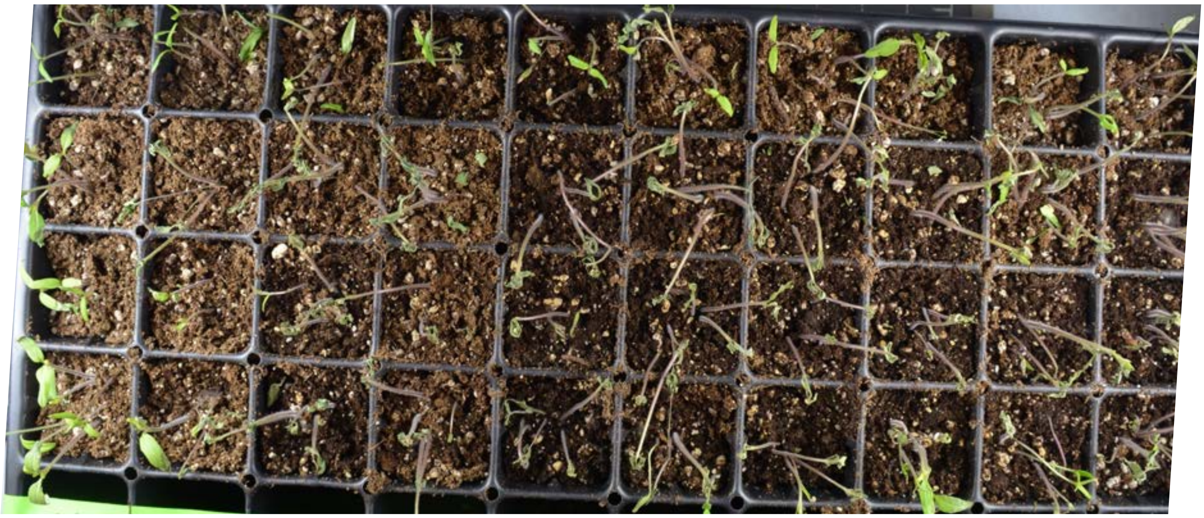

HopAB1n | E.V. | HopQ1b | HopQ1a | HopAI1a | HopAI1b

Exp 15

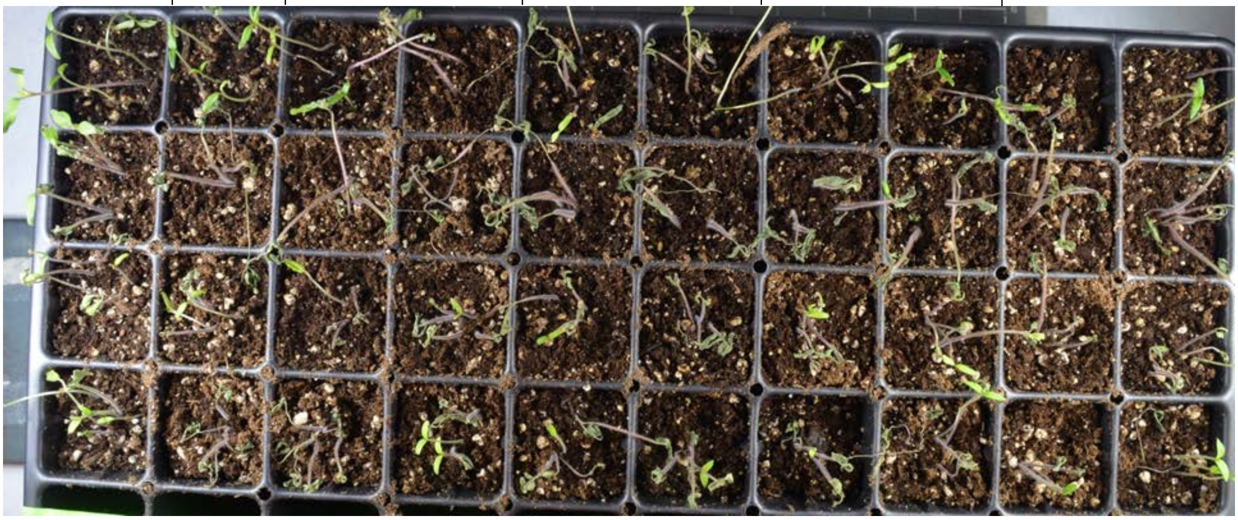

HopAB1n	E.V.	HopD2d	HopB1a	HopB1d	HopB1b

Exp 16

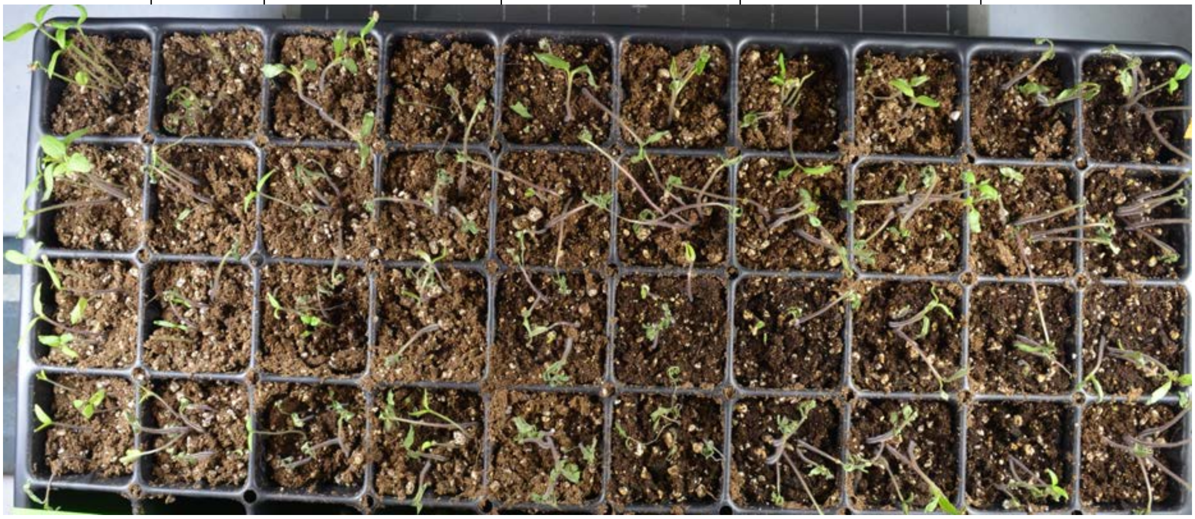

HopAB1n	E.V.	HopAR1g	HopAR1b	HopAR1c	HopAR1e

Exp 17

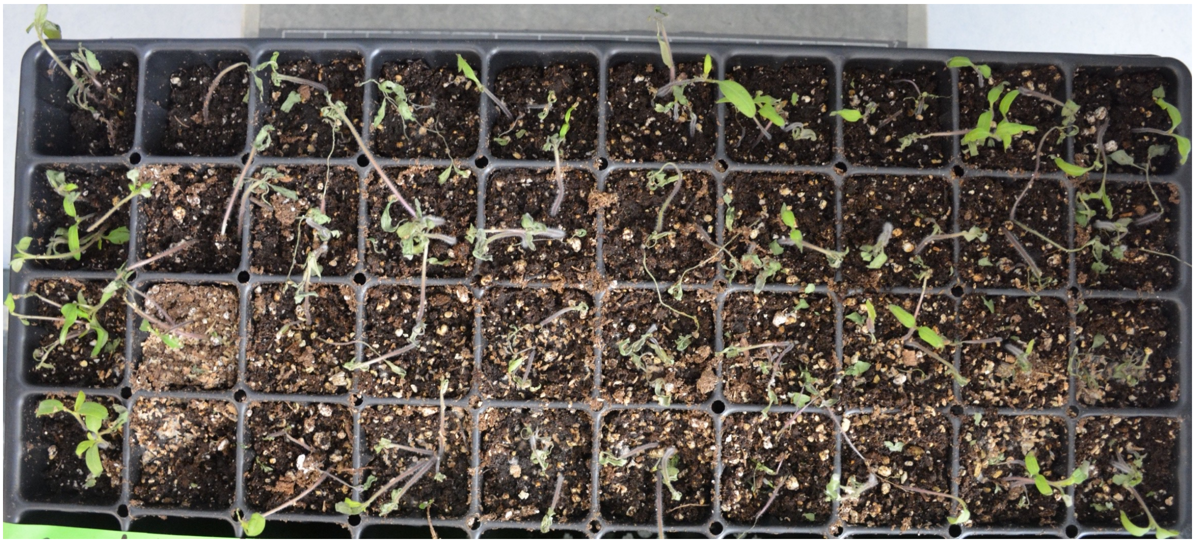

HopAB1n	E.V.	HopAI1g	HopAI1m	HopAI1k	HopZ1m

Exp 18

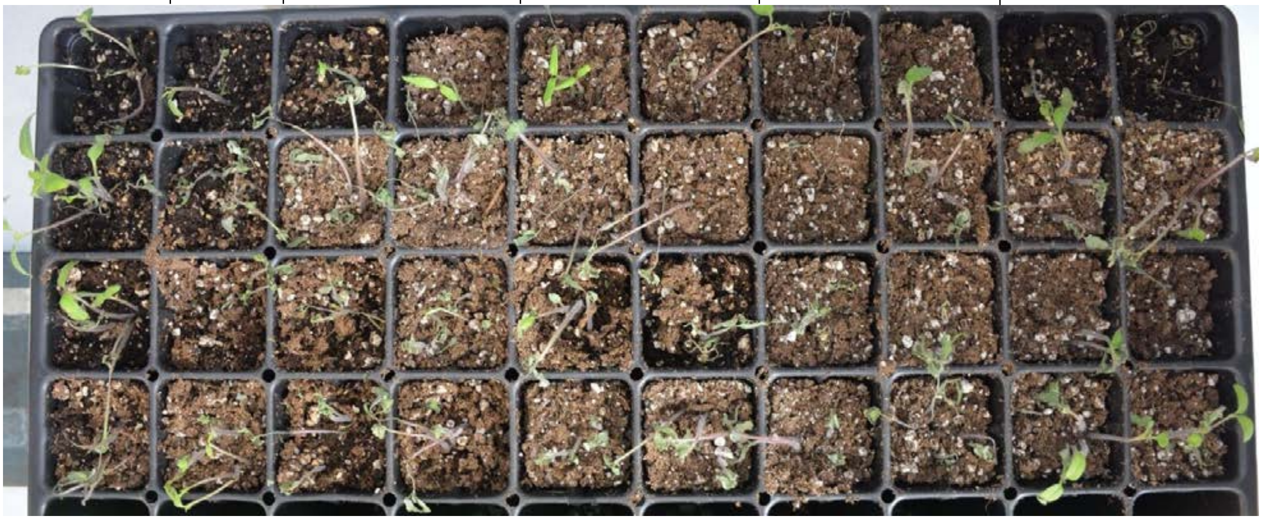

HopAB1n | E.V. | HopZ1g | HopZ1a | HopZ1e | HopAR1a

Exp 19

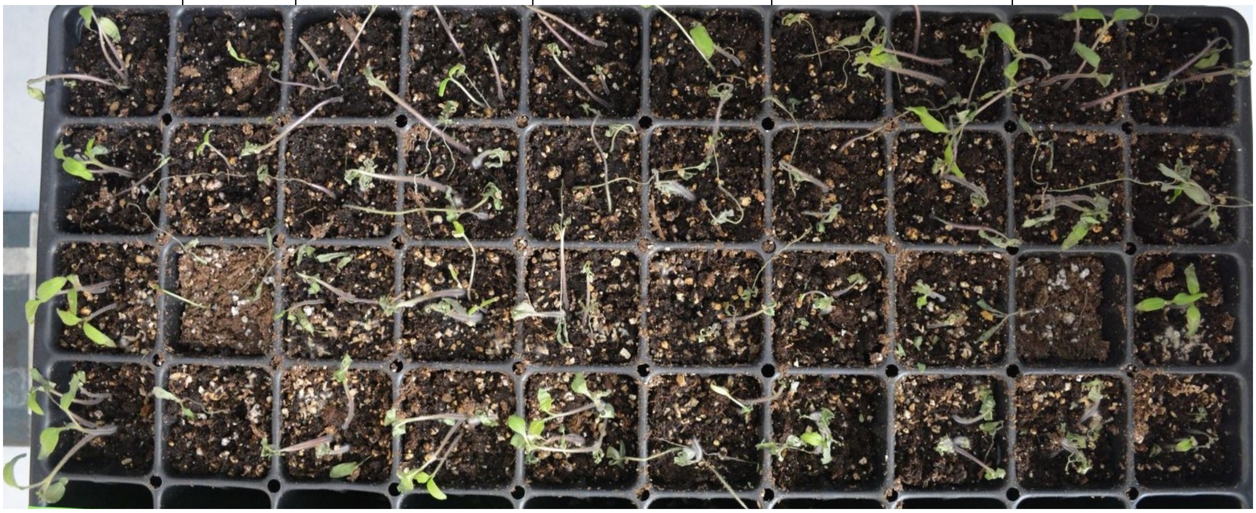

HopAB1n | E.V. | AvrPto1f | AvrPto1d | AvrPto1m | AvrPto1k

Exp 20

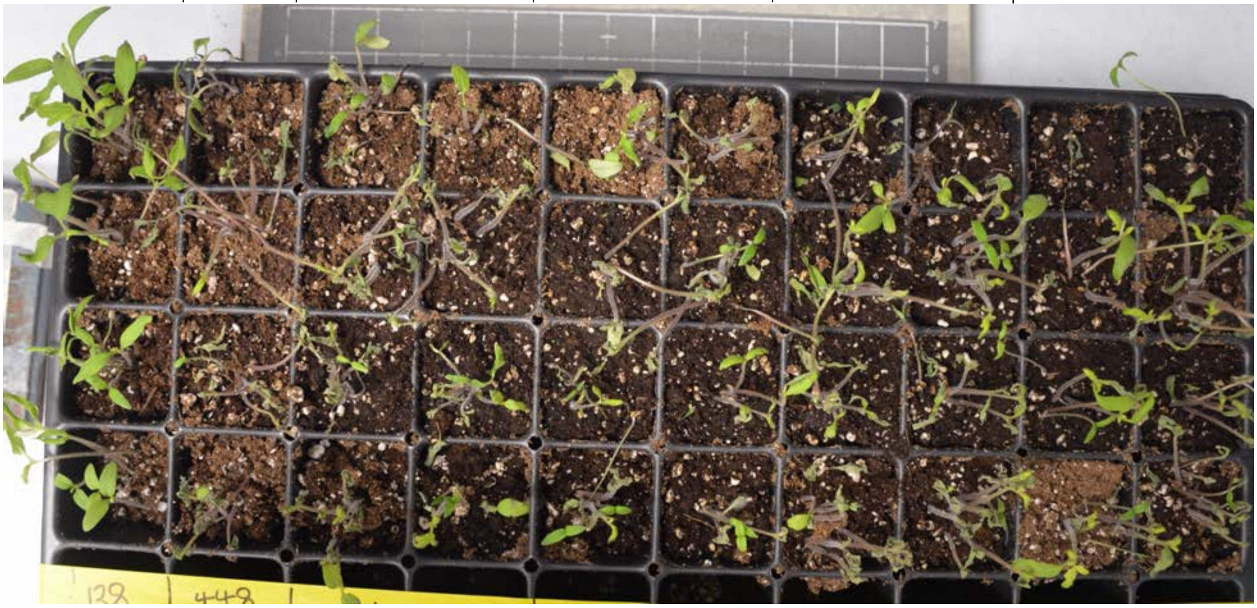

HopAB1n | E.V. | AvrPto1b | XXXXX | XXXXXX | XXXXXX

Exp 21

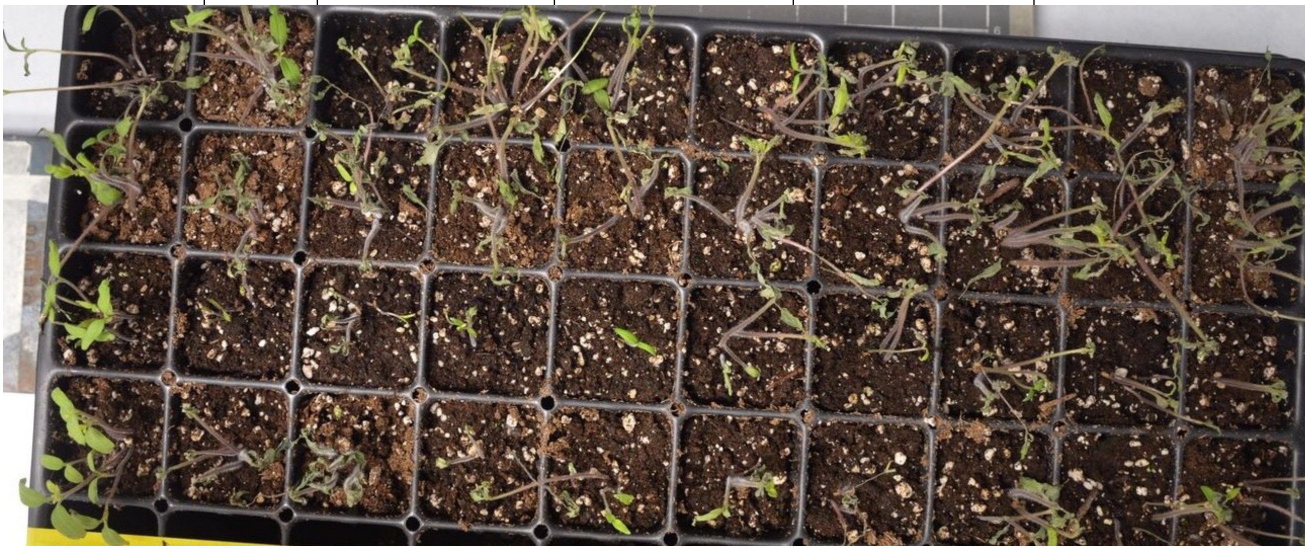

HopAB1n | E.V. | HopAA1f | HopAA1k | HopAA1g | HopAA1o

Exp 22

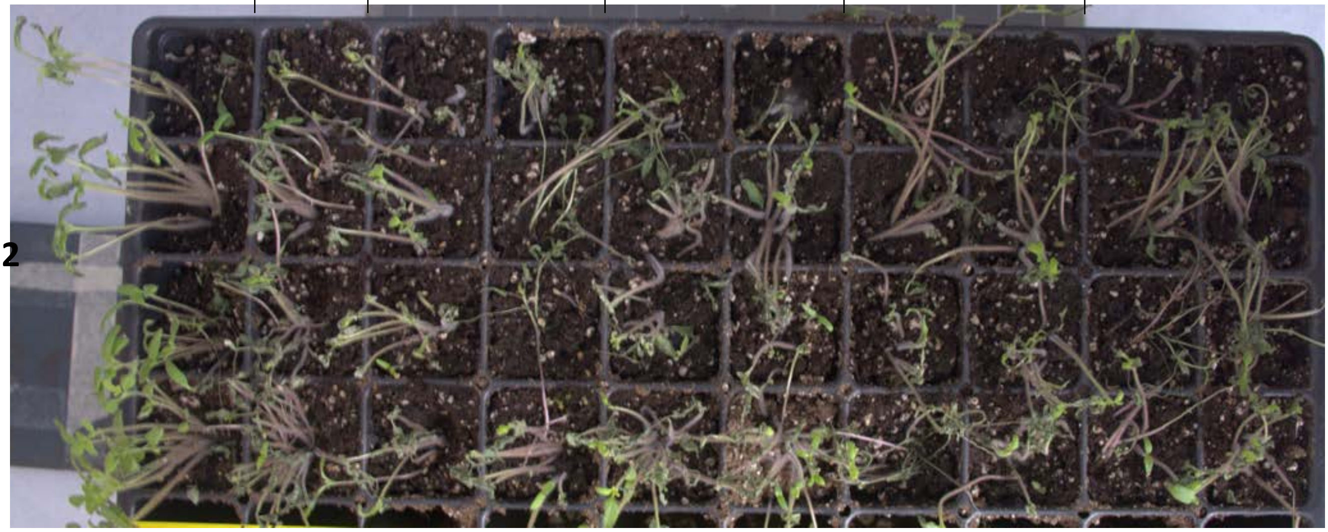

HopAB1n | E.V. | AvrPto1e | AvrPto1i | AvrPto1h | AvrPto1c

Exp 23

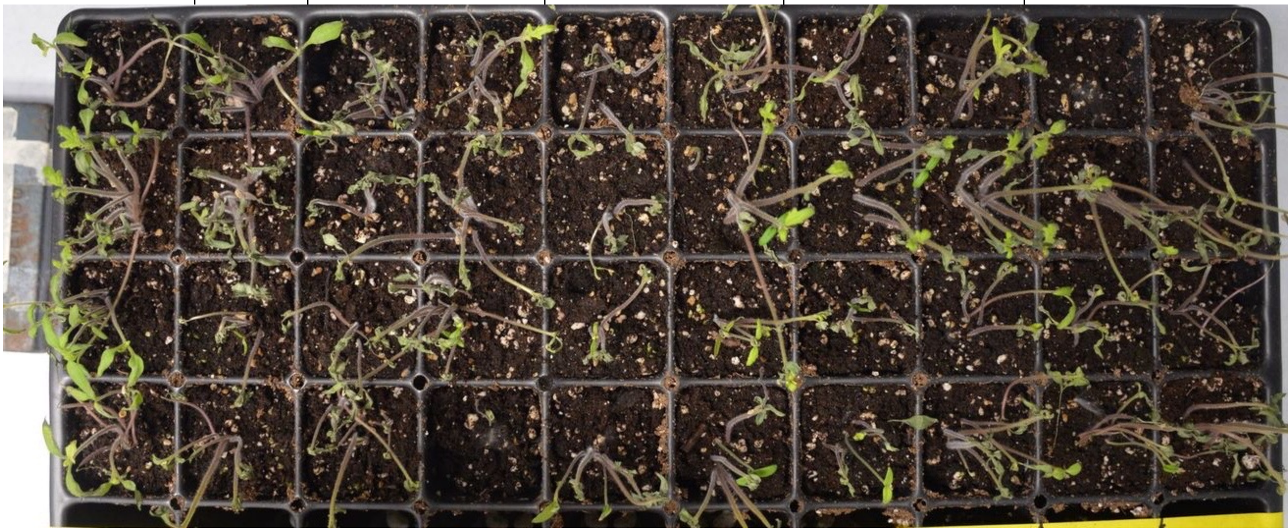

HopAB1n | E.V. | HopF1j | HopF3a | HopF1b | HopF1h

Exp 24

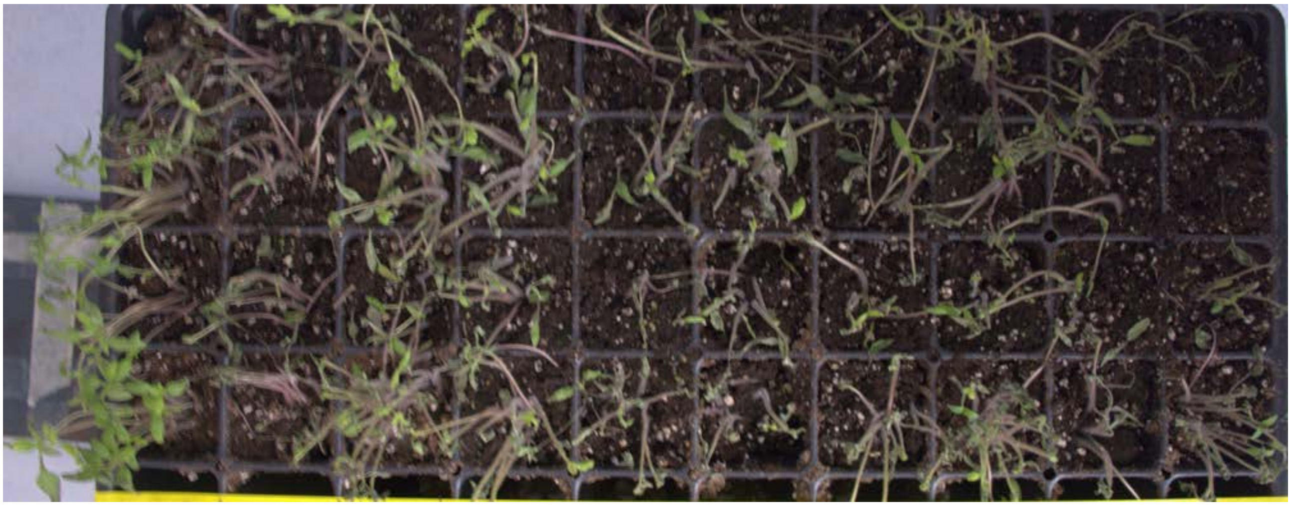

HopAB1n | E.V. | HopAA1m | HopAA1c | HopAA1d | HopAA1q

Exp 25

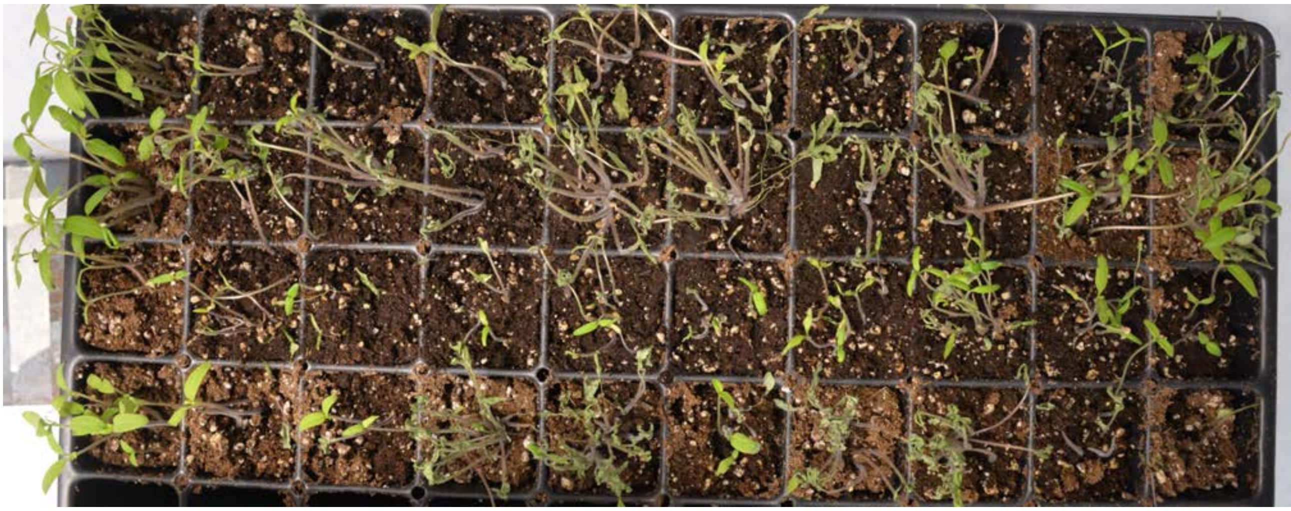

HopAB1n | E.V. | HopAF1d | HopAF1e | HopAF1l | HopAF1k

Exp 26

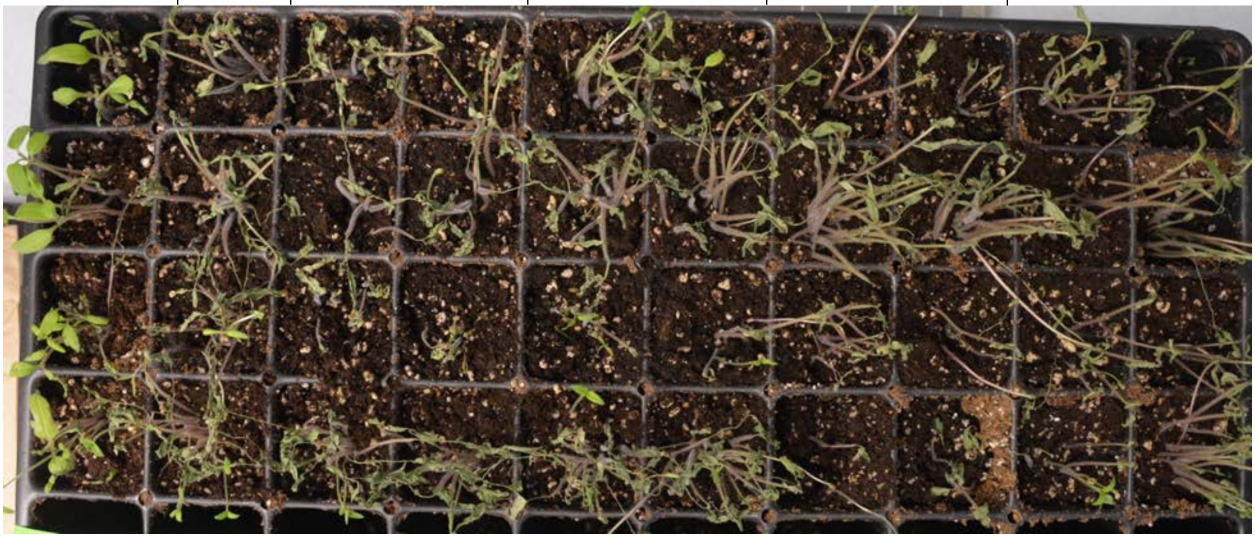

HopAB1n | E.V. | HopAF1n | HopAG1m | HopAG1g | HopAG1d

Exp 27

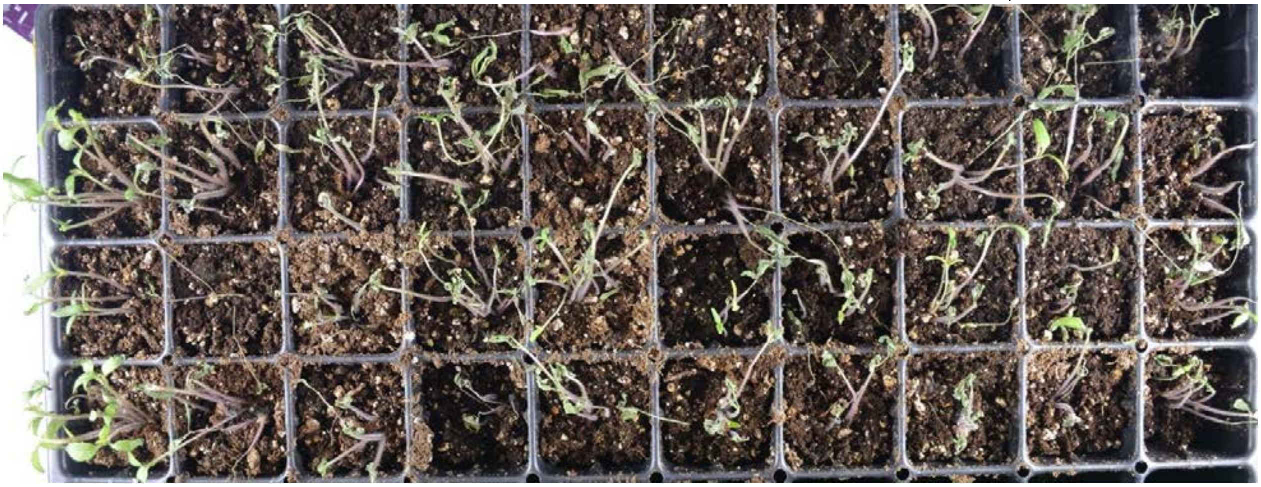

Exp 28

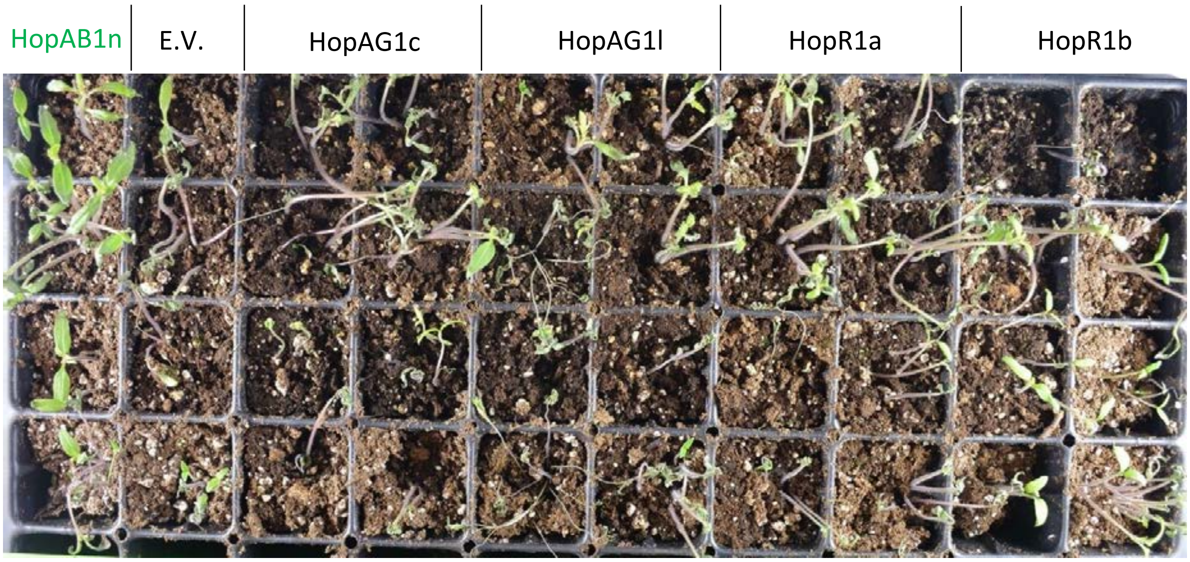

Exp 29

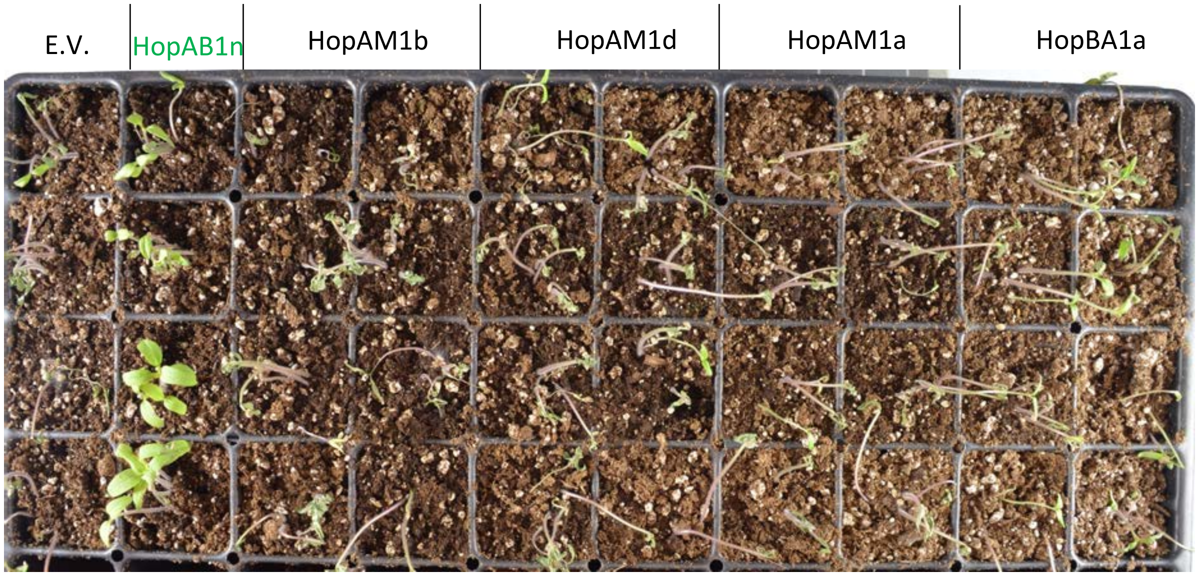

Exp 30

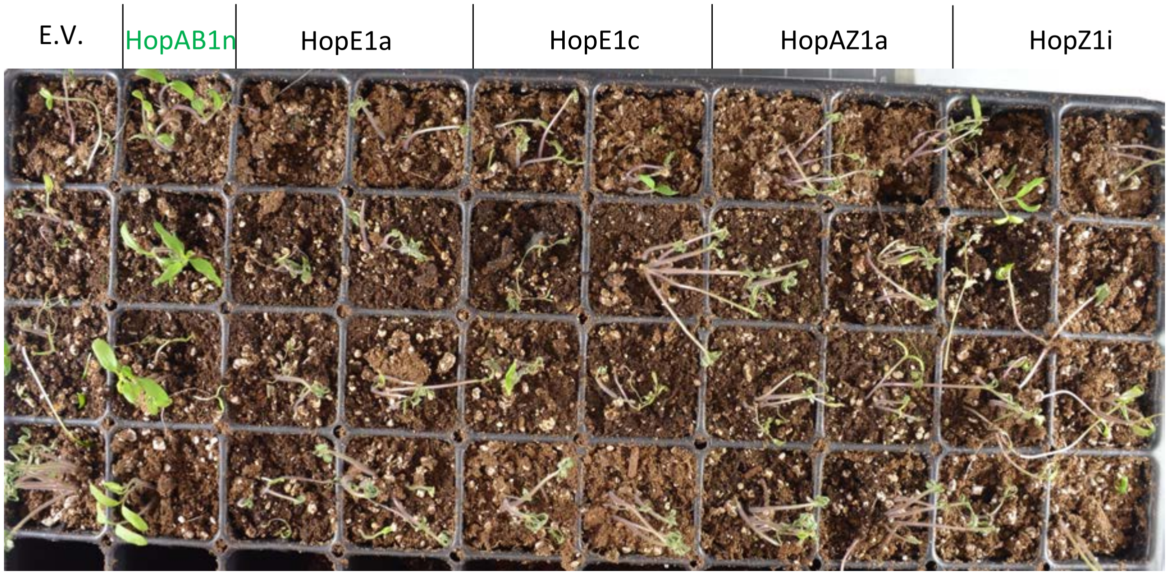

E.V. | HopAB1n | HopAZ1c | HopAZ1p | HopAZ1h | HopAZ1d

Exp 31

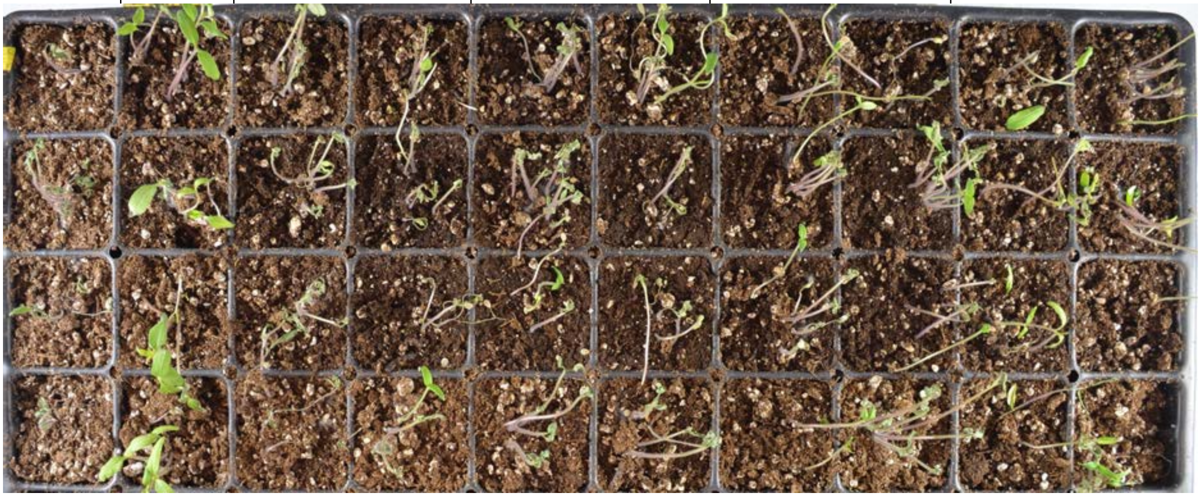

E.V. | HopAB1n | HopBM1a | HopBM1b | HopBG1a | HopF1e

Exp 32

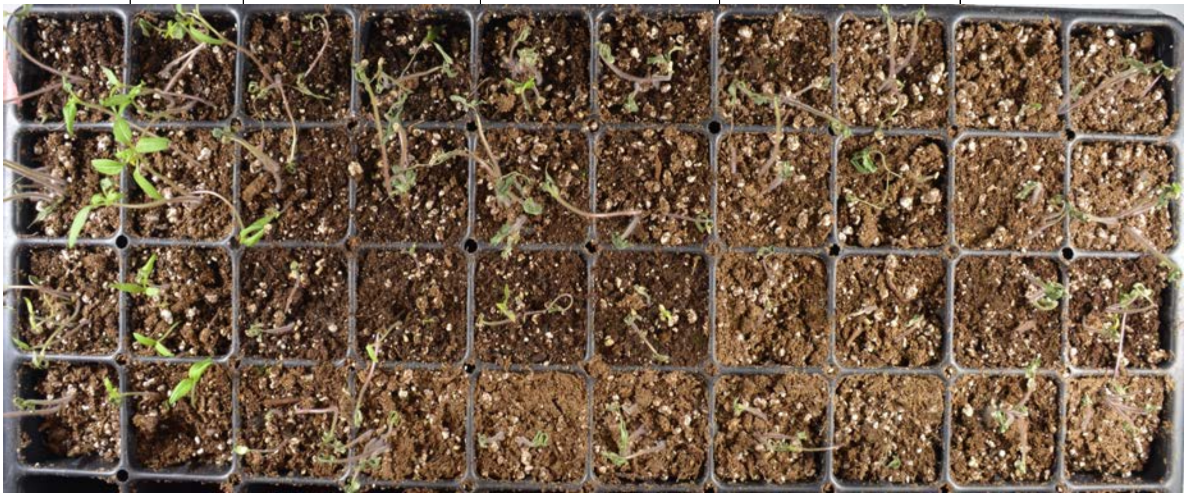

E.V. | HopAB1n | HopBP1c | HopBP1a | HopBP1f | HopZ1d

Exp 33

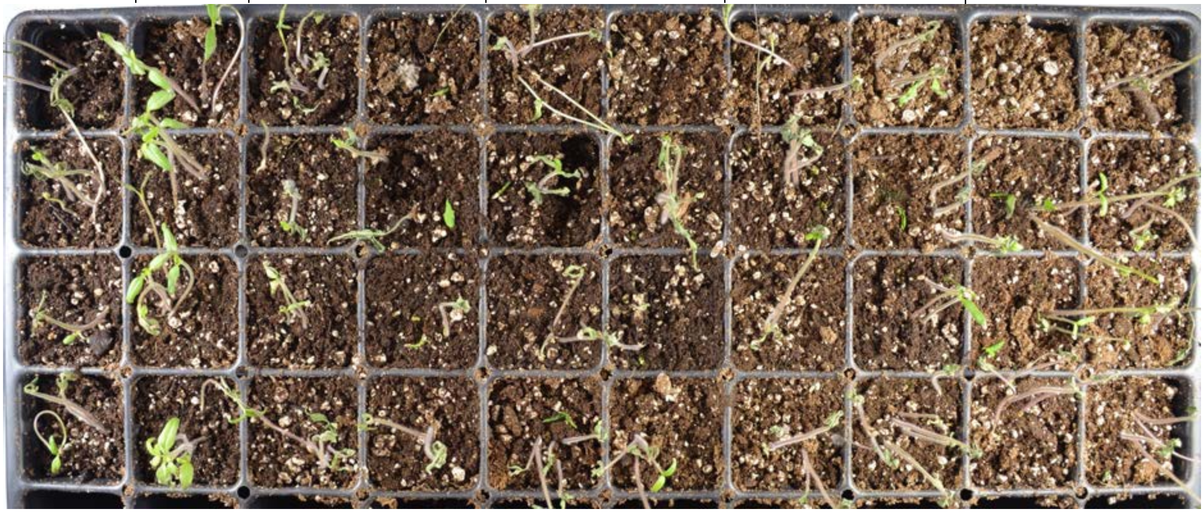

E.V. | HopAB1n | HopZ1b | HopZ1j | HopZ1i | HopZ2a

Exp 34

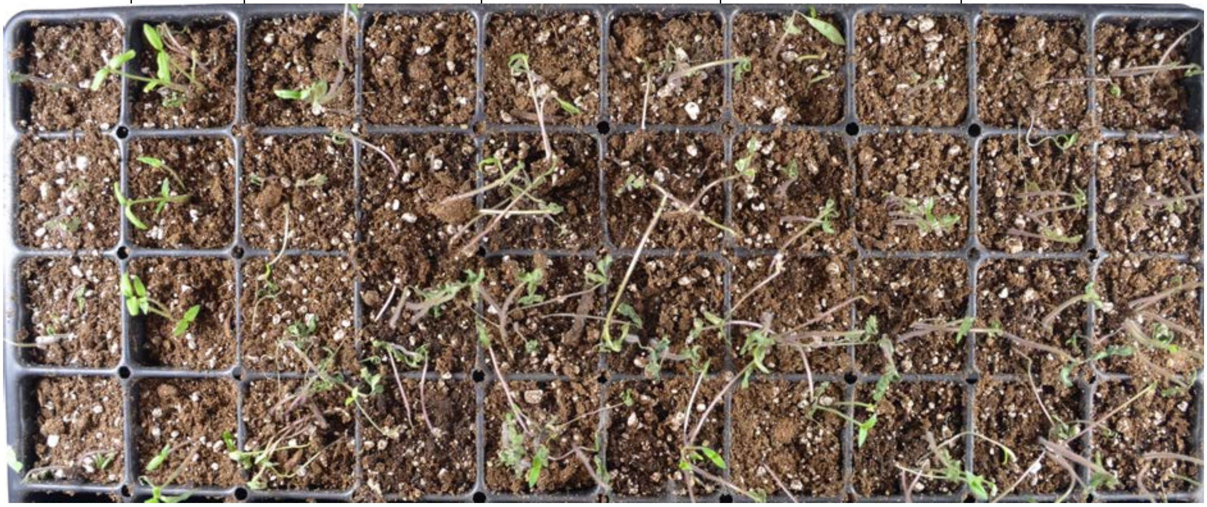

E.V. | HopAB1n | HopF1a | HopF1q | HopBP1b | HopBP1g

Exp 35

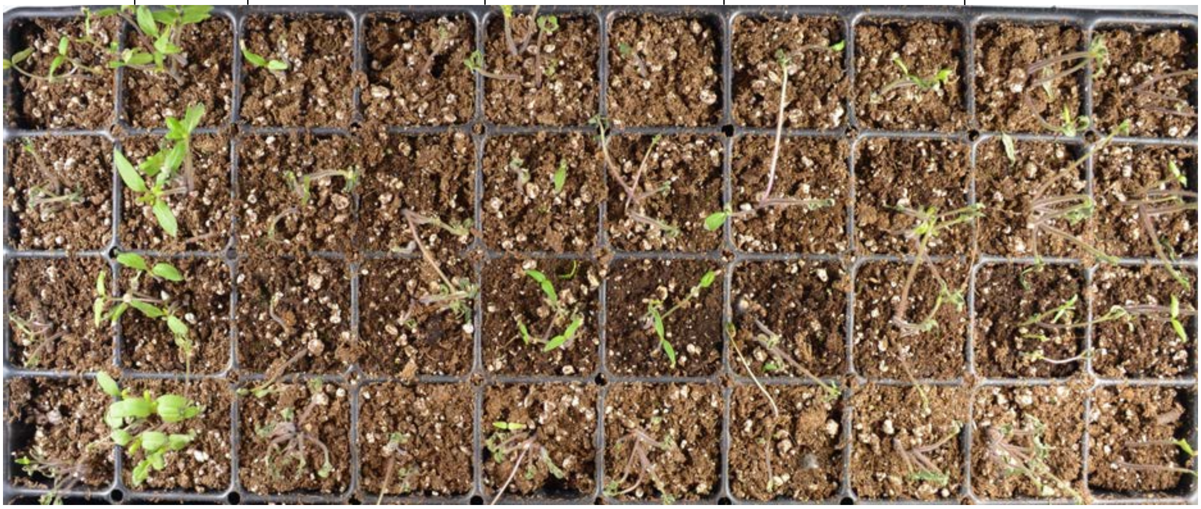

E.V. | HopAB1n | HopZ2b | HopZ2c | HopZ4a | AvrE1f

Exp 36

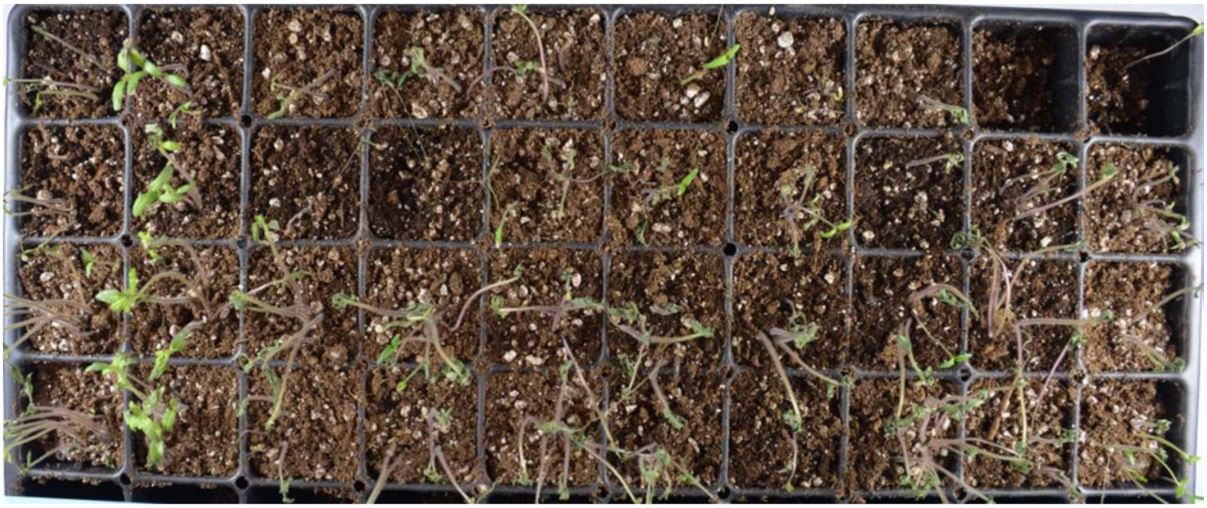

E.V. | HopAB1n | AvrE1e | AvrE1k | AvrE1h | AvrE1g

Exp 37

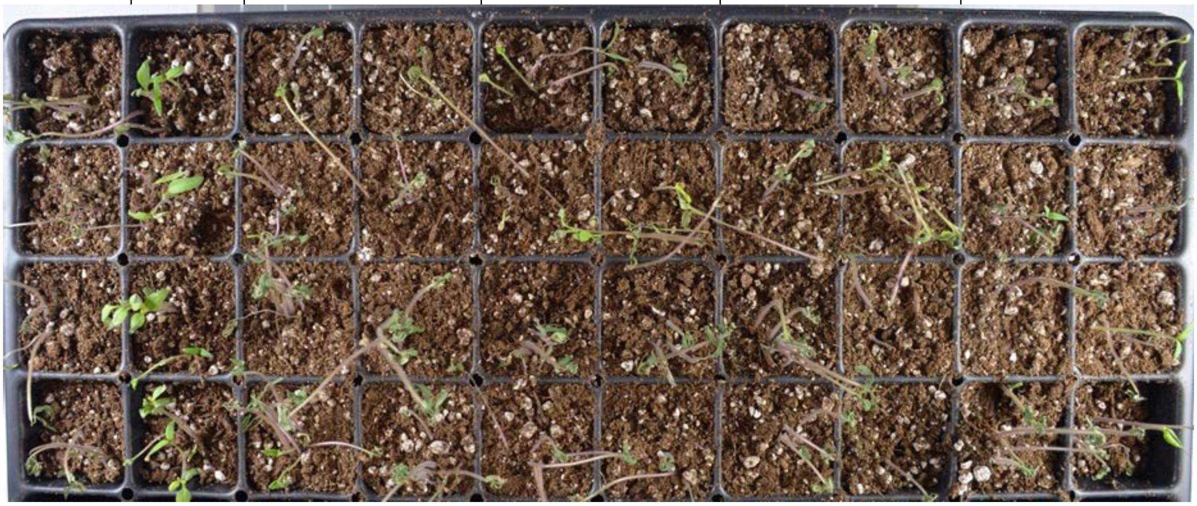

HopAB1n | E.V. | AvrE1s | AvrE1j | AvrE1a | AvrE1p

Exp 38

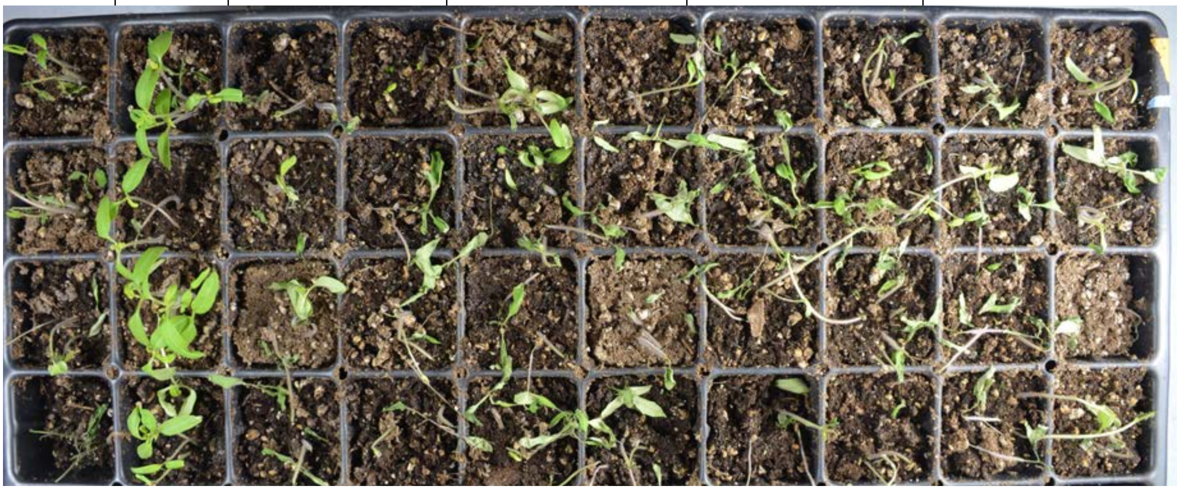

HopAB1n | E.V. | AvrE1i | HopF1d | HopF1p | HopF1c

Exp 39

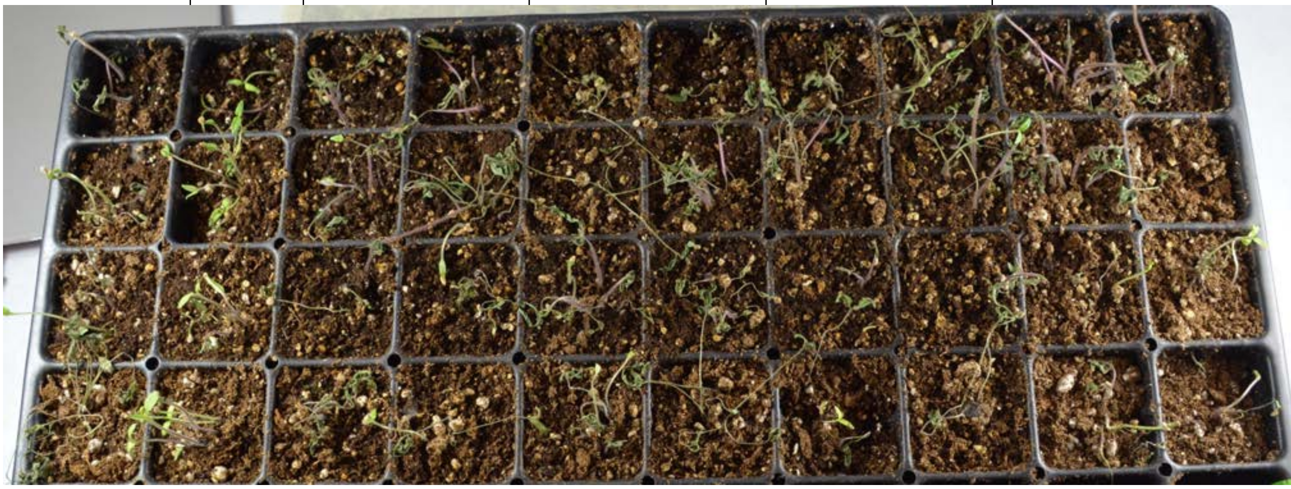

Exp 40

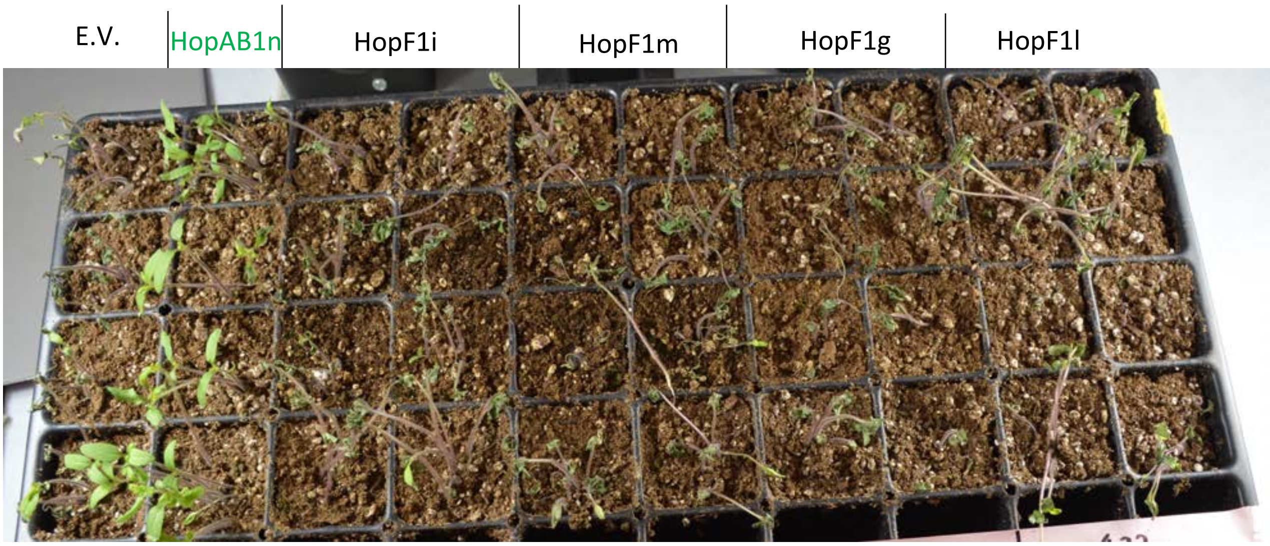

Exp 41

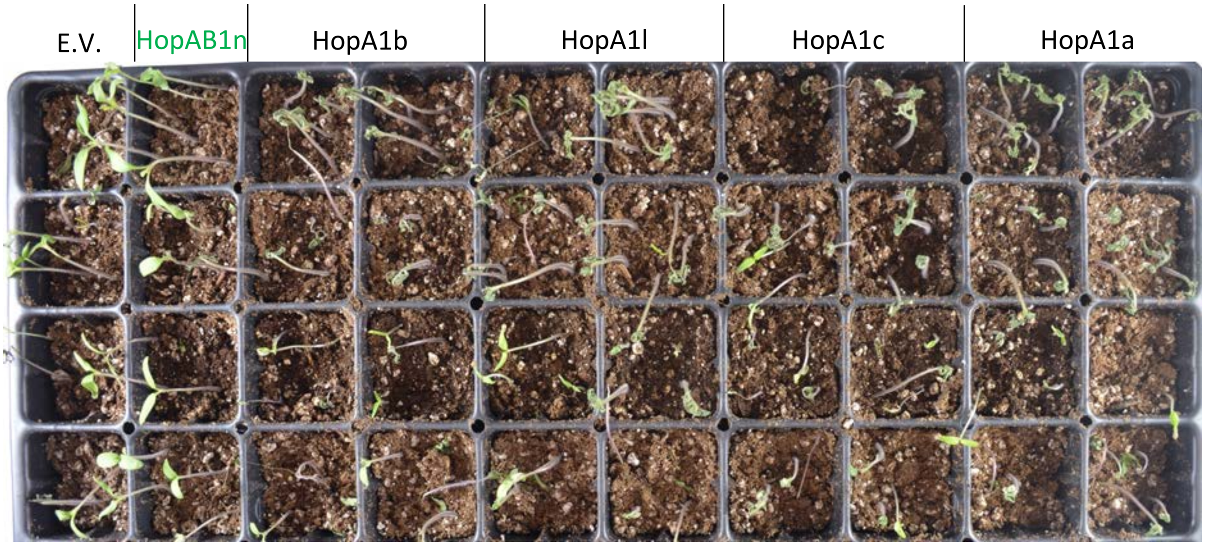

Exp 42

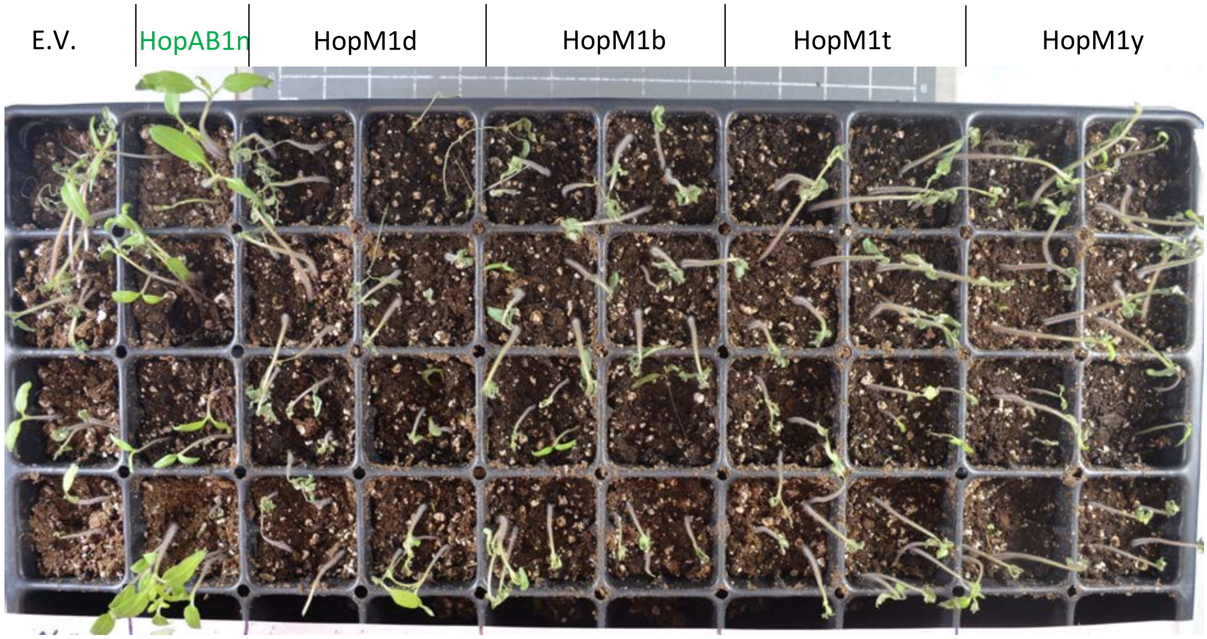

E.V. | HopAB1n | HopM1g | HopM1x | HopM1r | HopM1f

Exp 43

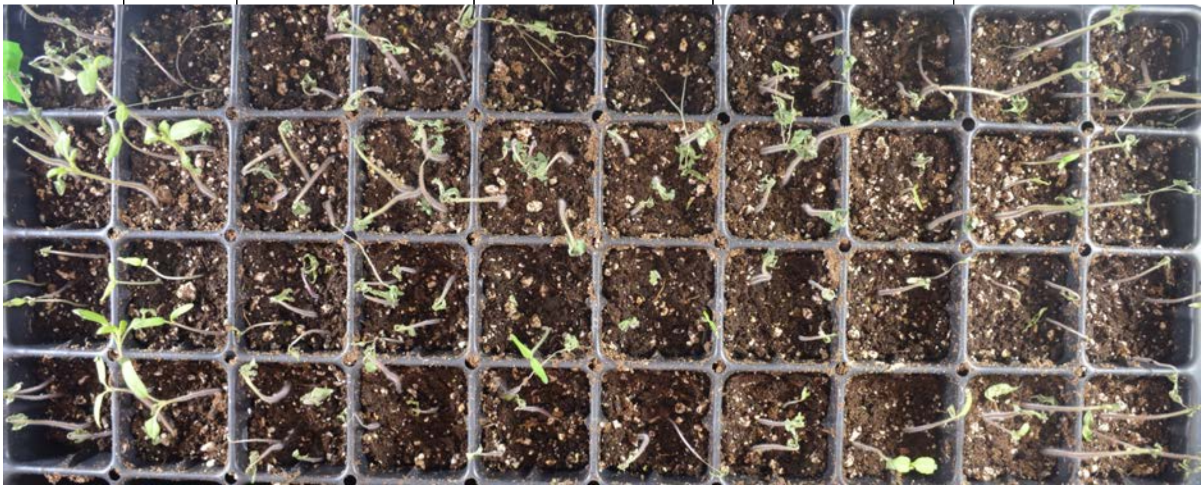

E.V. | HopAB1n | HopM1h | HopM1n | HopM1j | HopV1b

Exp 44

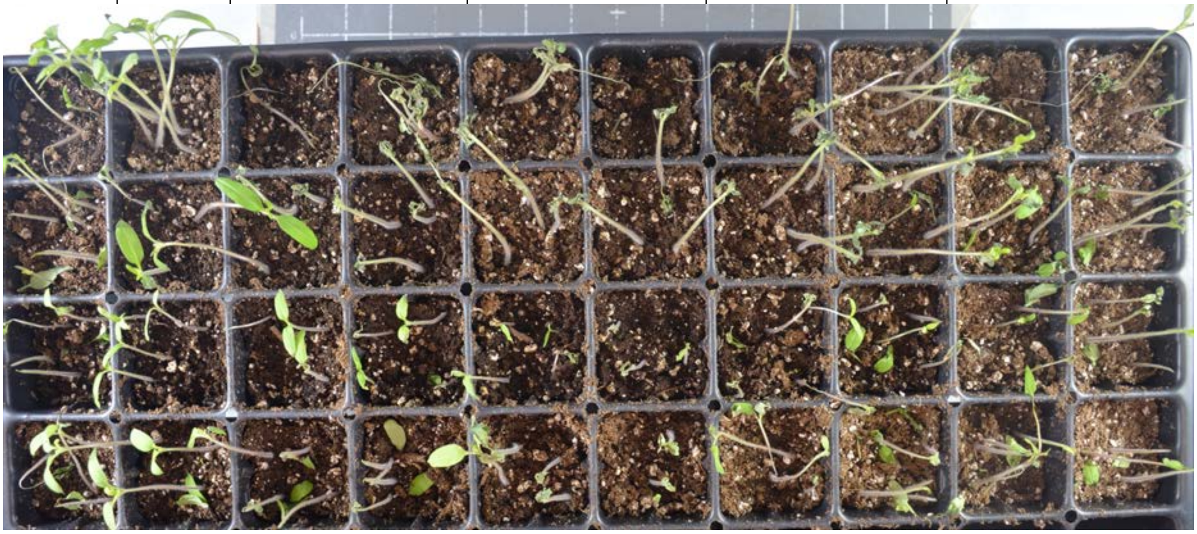

HopAB1n | E.V. | HopV1j | HopV1i | HopV1k | HopV1o

Exp 45

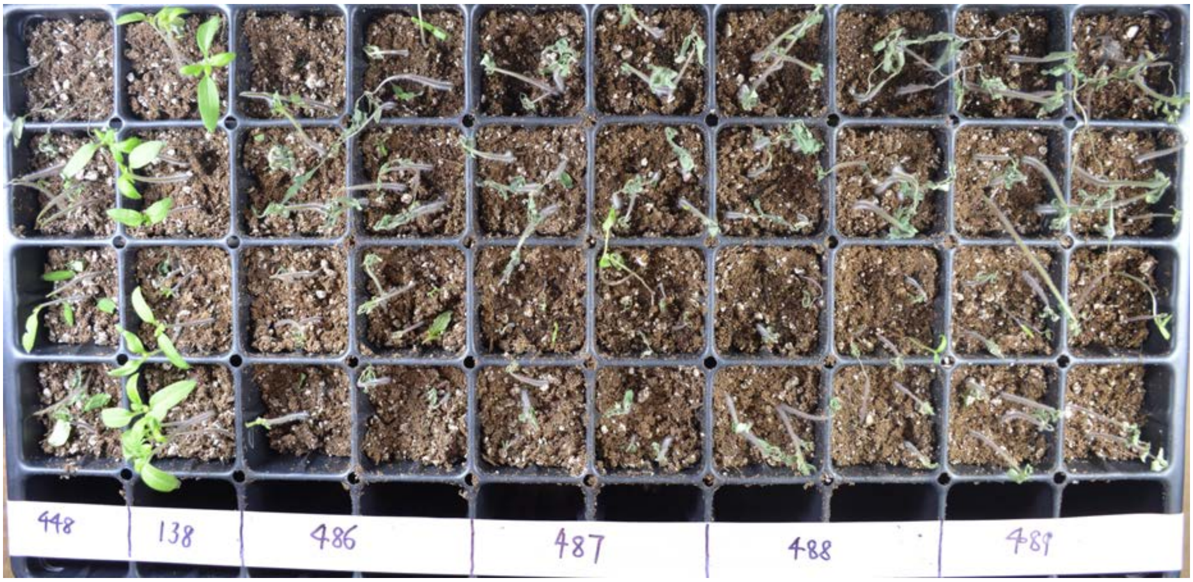

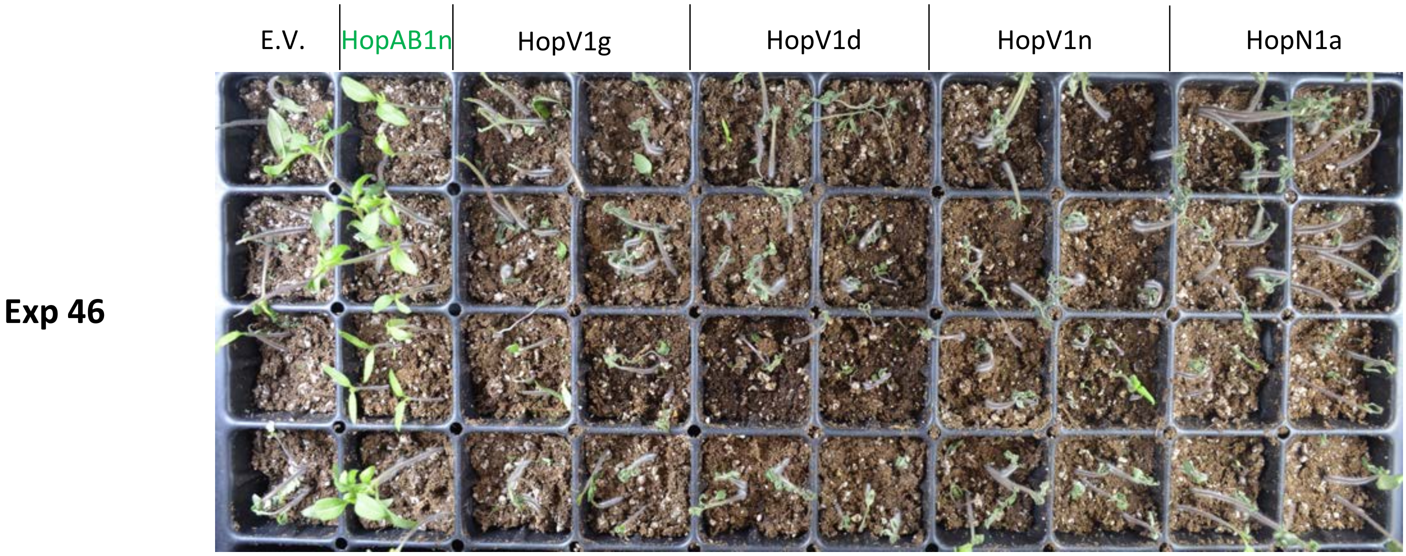
E.V.
HopAB1n
HopV1g
HopV1d
HopV1n
HopN1a
Exp 46

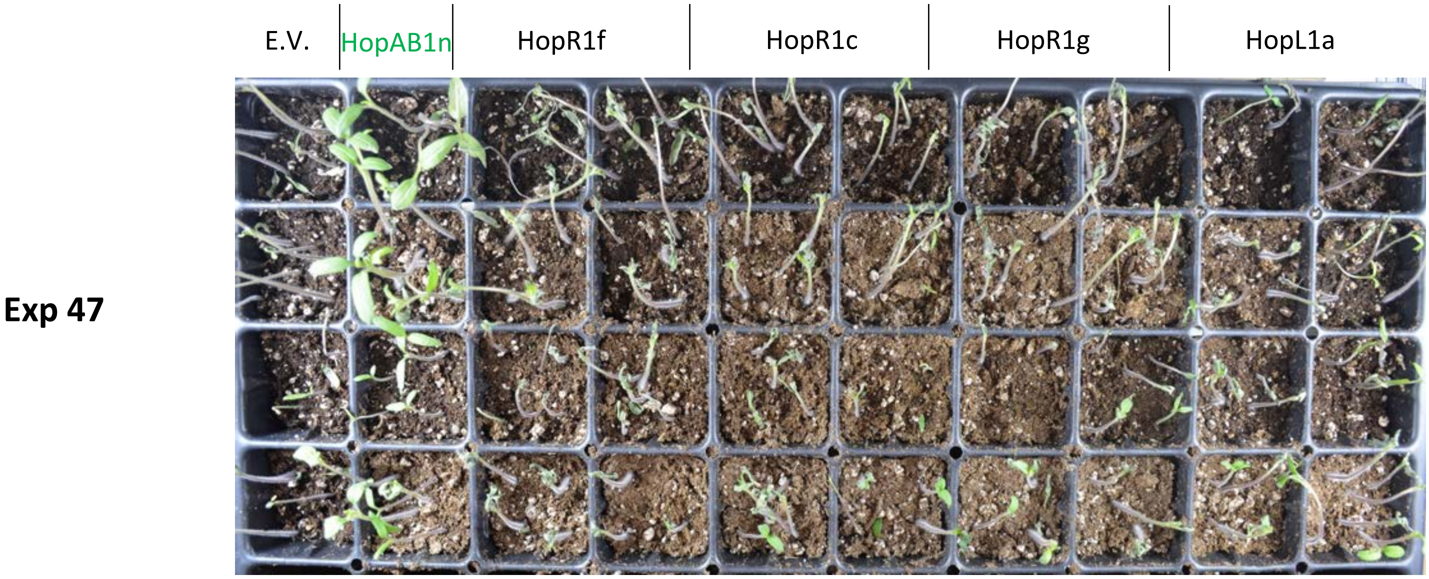
E.V.
HopAB1n
HopR1f
HopR1c
HopR1g
HopL1a
Exp 47

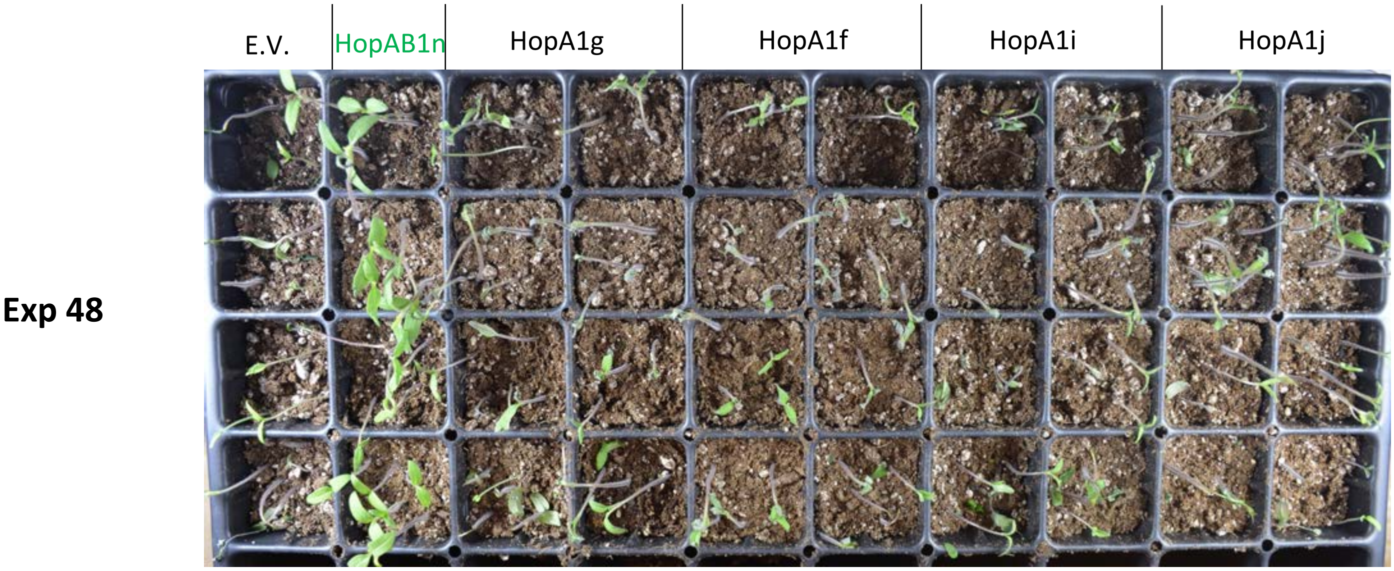
E.V.
HopAB1n
HopA1g
HopA1f
HopA1i
HopA1j
Exp 48

Exp 49

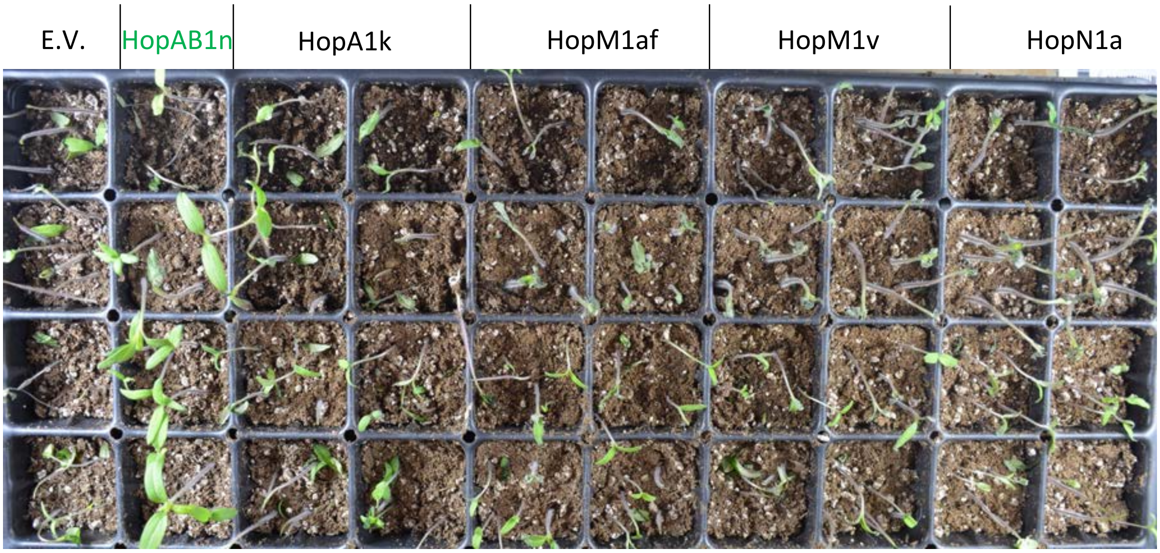

Exp 50

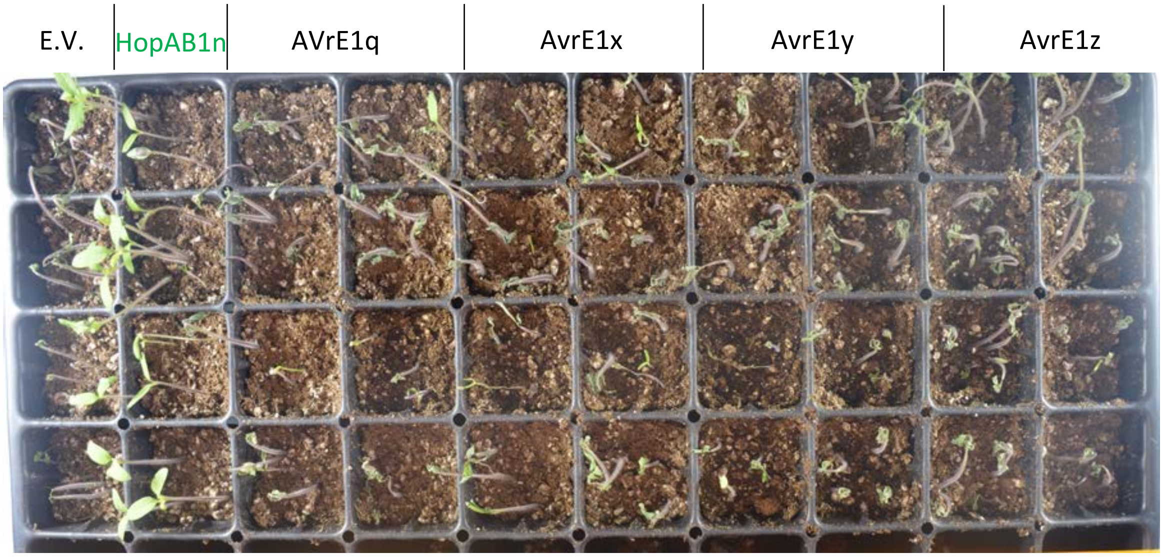

Exp 51

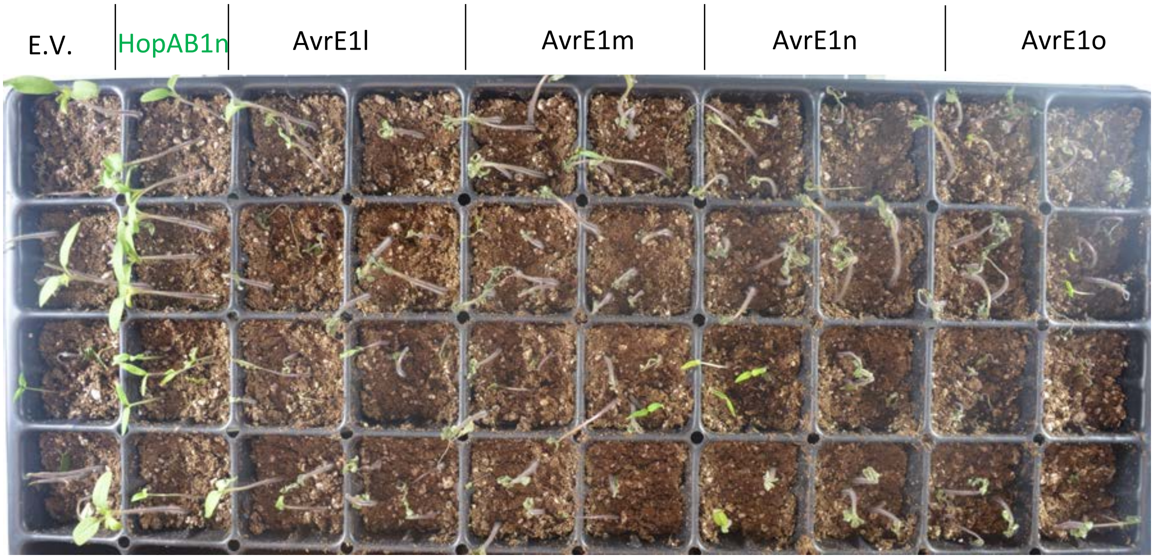

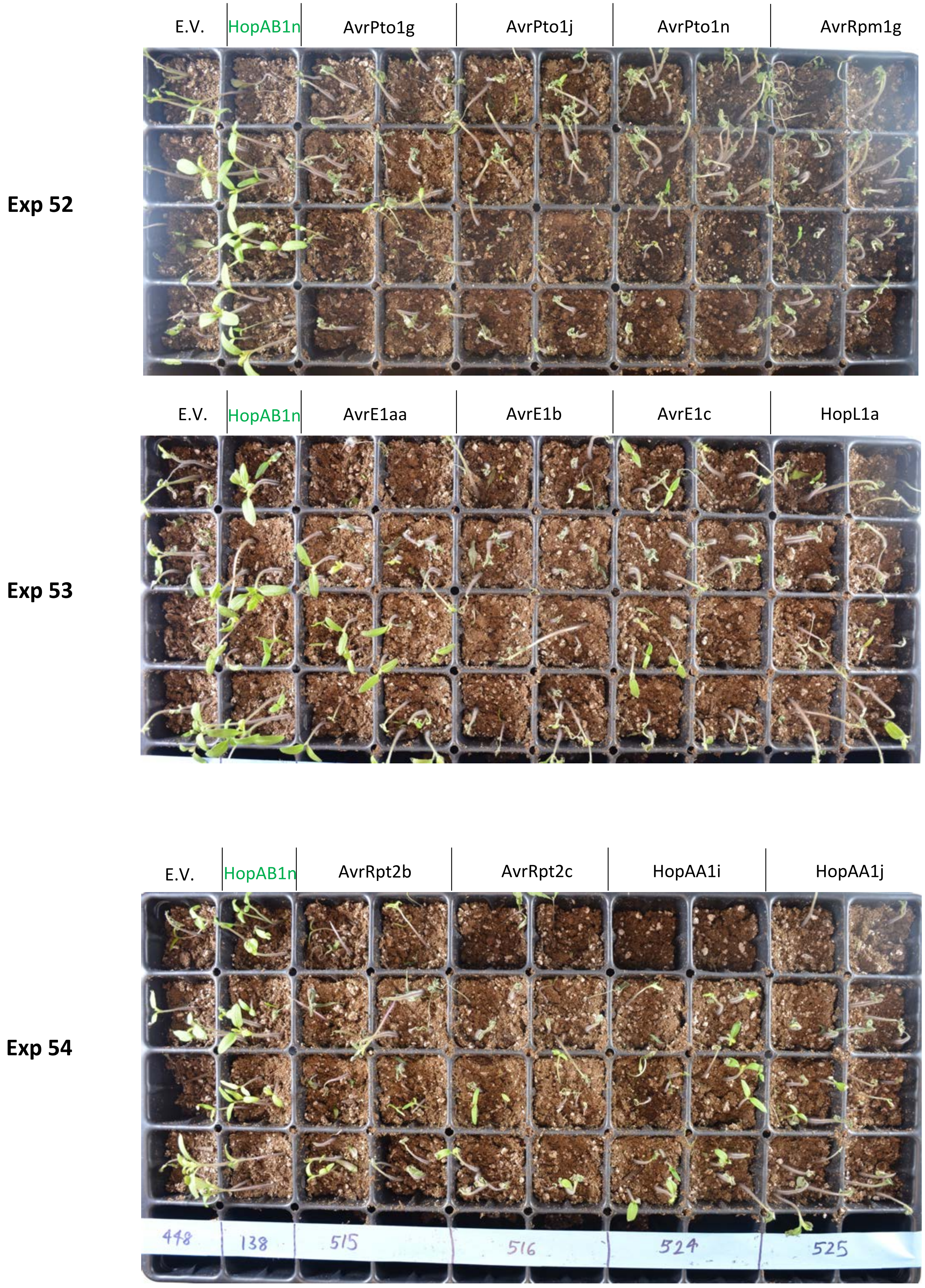
Exp 52
E.V.
HopAB1n
AvrPto1g
AvrPto1j
AvrPto1n
AvrRpm1g
Exp 53
E.V.
HopAB1n
AvrE1aa
AvrE1b
AvrE1c
HopL1a
Exp 54
E.V.
HopAB1n
AvrRpt2b
AvrRpt2c
HopAA1i
HopAA1j
448
138
515
516
524
525

Exp 55

E.V. | HopAB1n | HopAA1l | HopAA1n | HopAA1p | HopAA1r

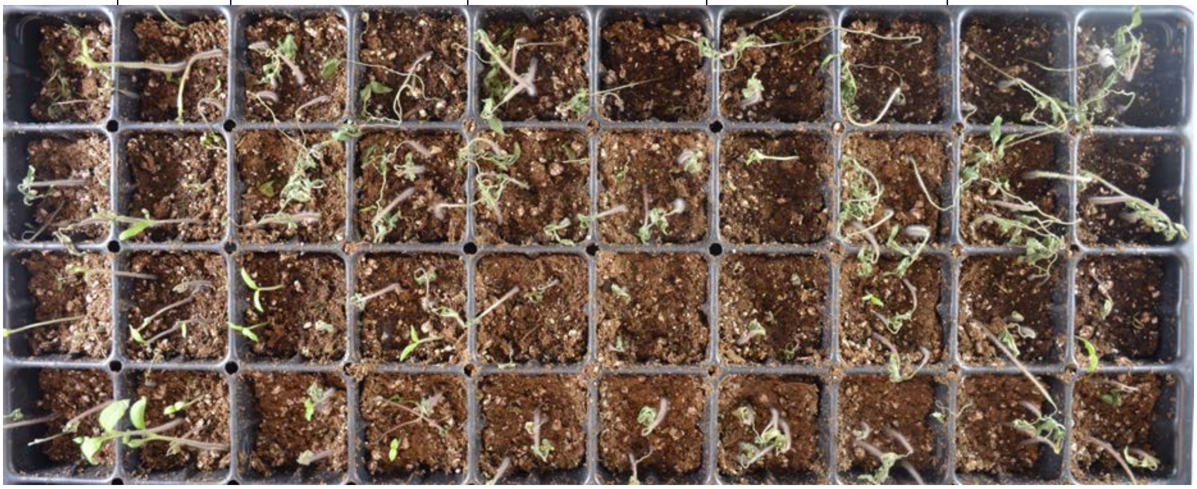

Exp 56

E.V. | HopAB1n | HopB4a | XXXX | HopBD1c | HopBD1e

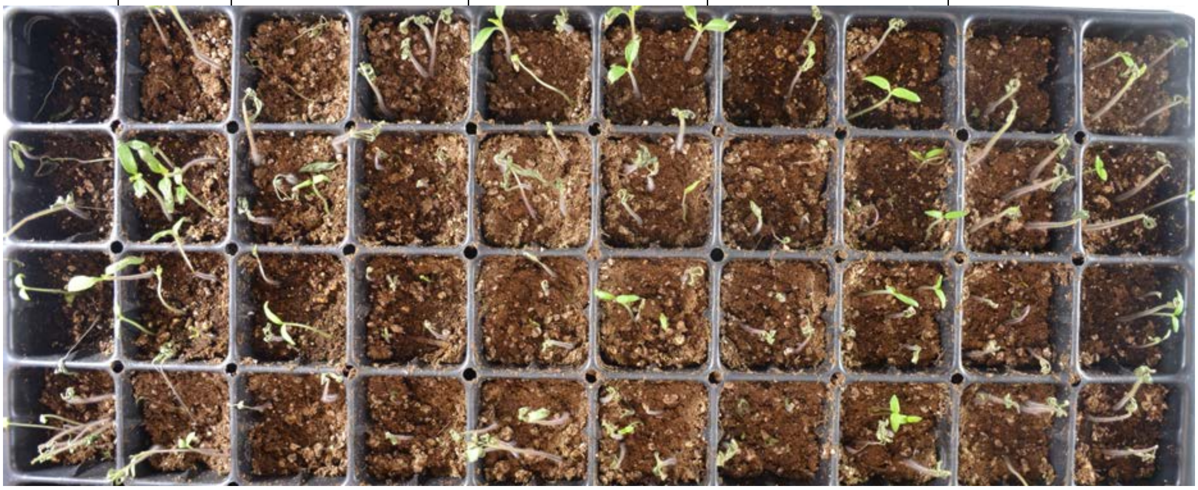

Exp 57

E.V. | HopAB1n | HopB1e | HopB2e | HopB2m | HopB2x

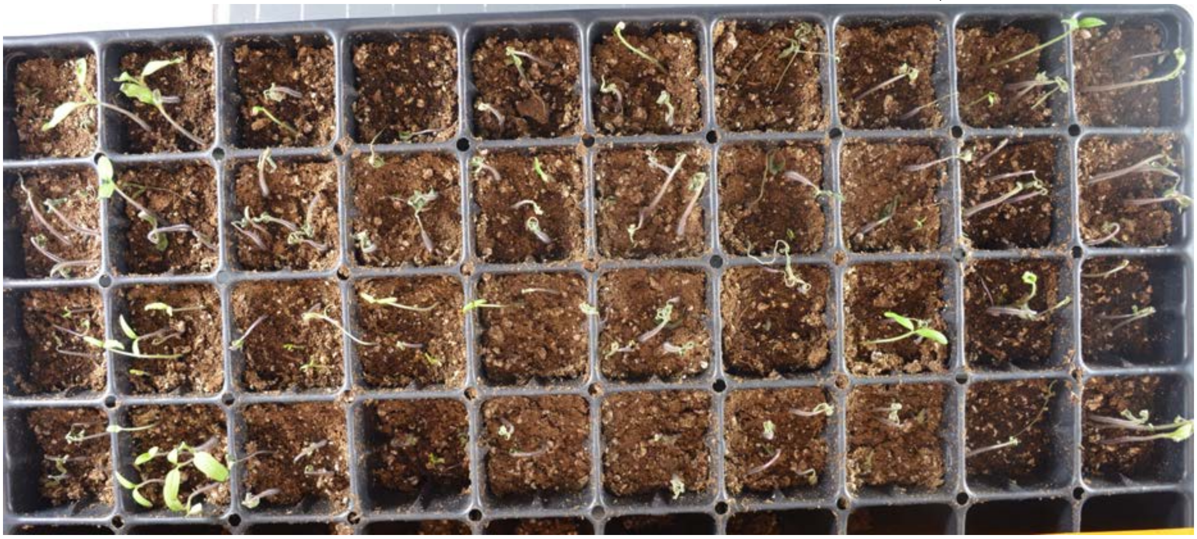

Exp 58

E.V.	HopAB1n	HopAZ1e	HopAZ1n	HopAZ1s	HopB1c

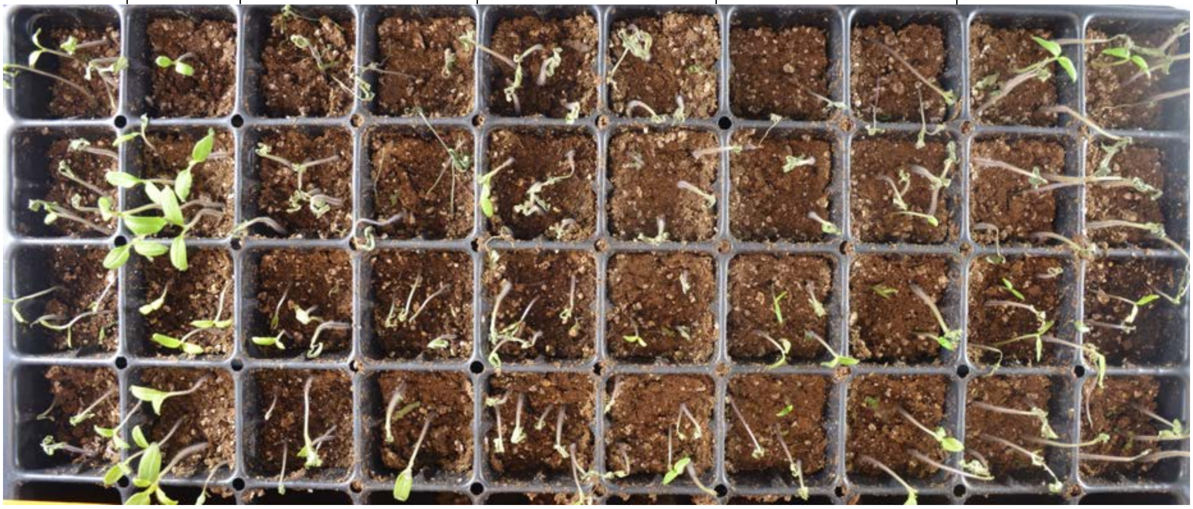

Exp 59

E.V.	HopAB1n	HopAF1f	HopAFh	HopAF1b	HopBD1f

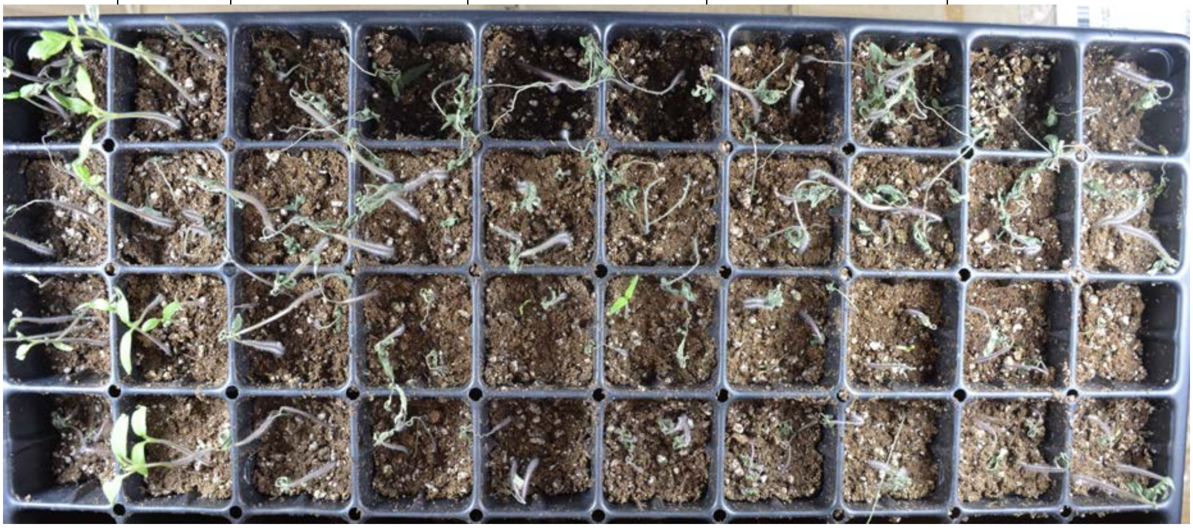

Exp 60

E.V.	HopAB1n	HopBF1b	HopBH1b	HopBH1d	HopBJ1a

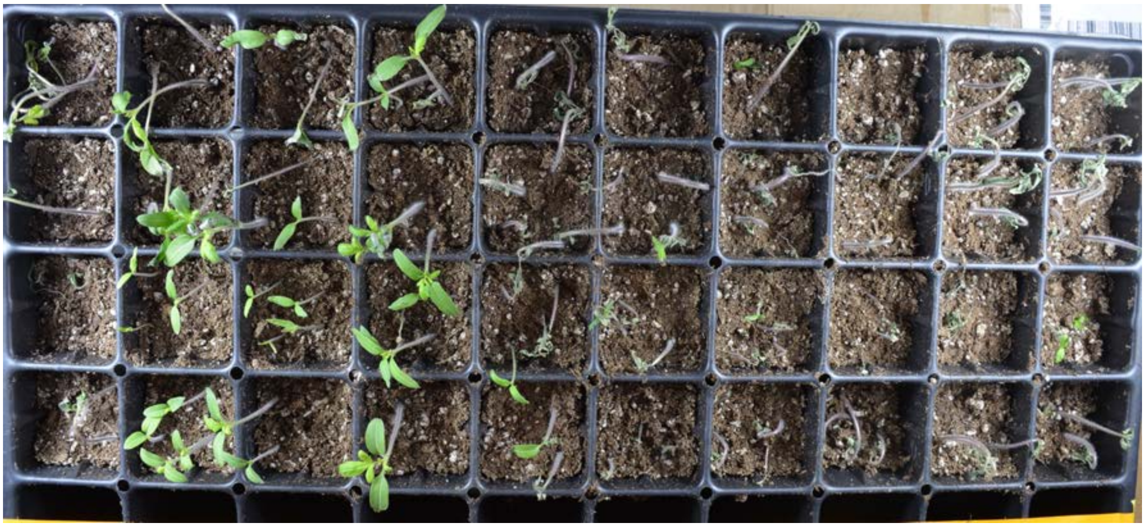

E.V. | HopAB1n | HopBI1a | HopAX1a | HopAX1f | HopBH1a

Exp 61

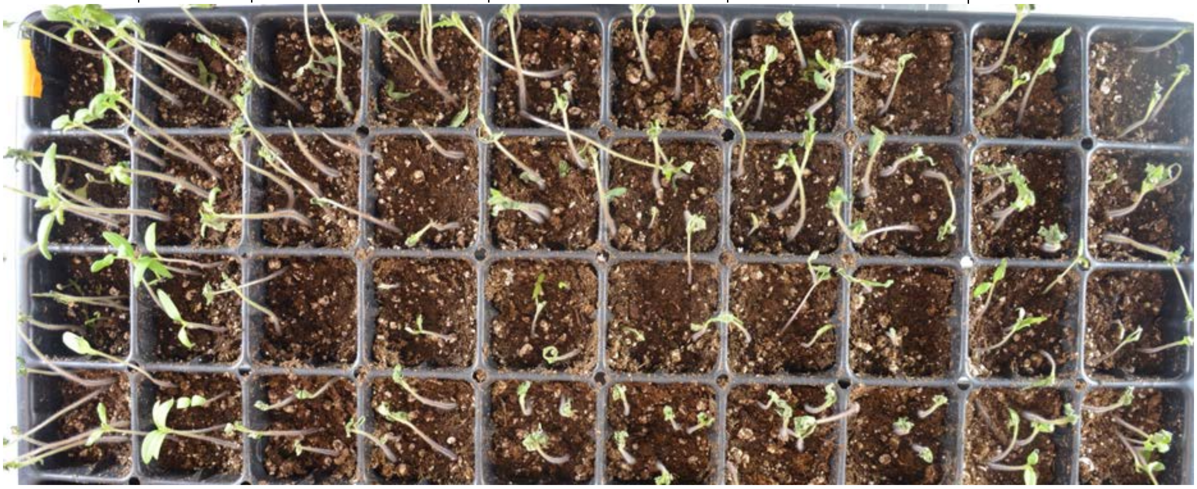

E.V. | HopAB1n | HopAT1e | HopAT1h | HopAU1b | HopAI1e

Exp 62

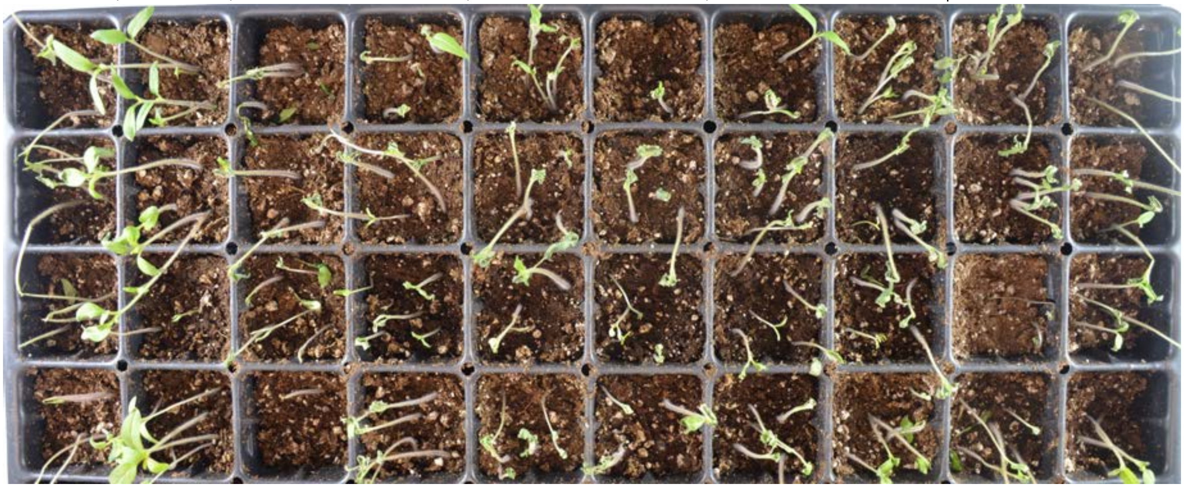

E.V. | HopAB1n | HopAU1a | HopAD1a | HopBI1b | HopAT1d

Exp 63

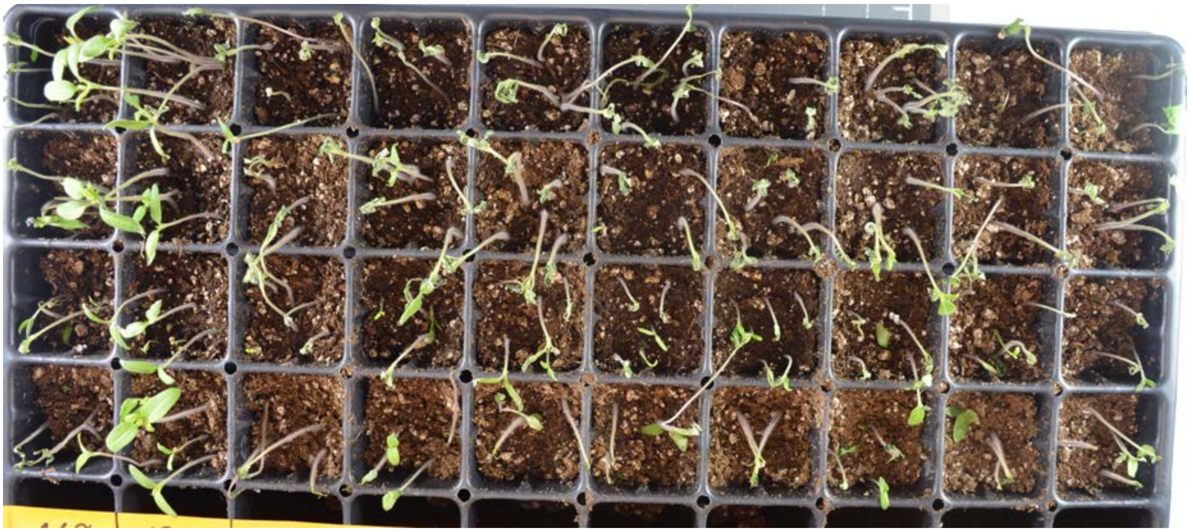

Exp 64

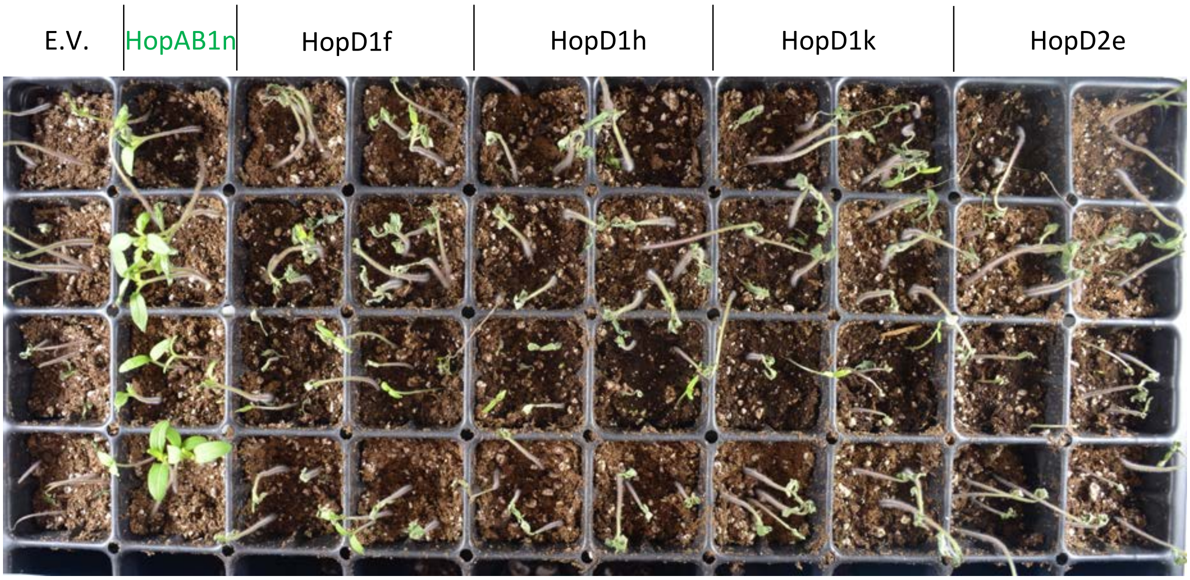

Exp 65

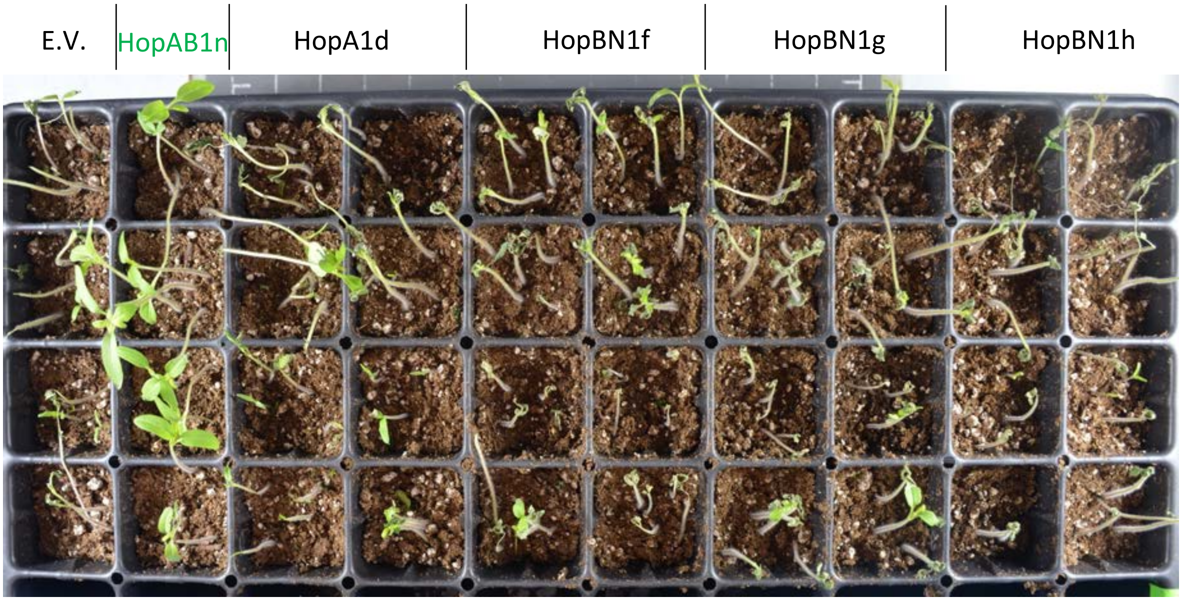

Exp 66

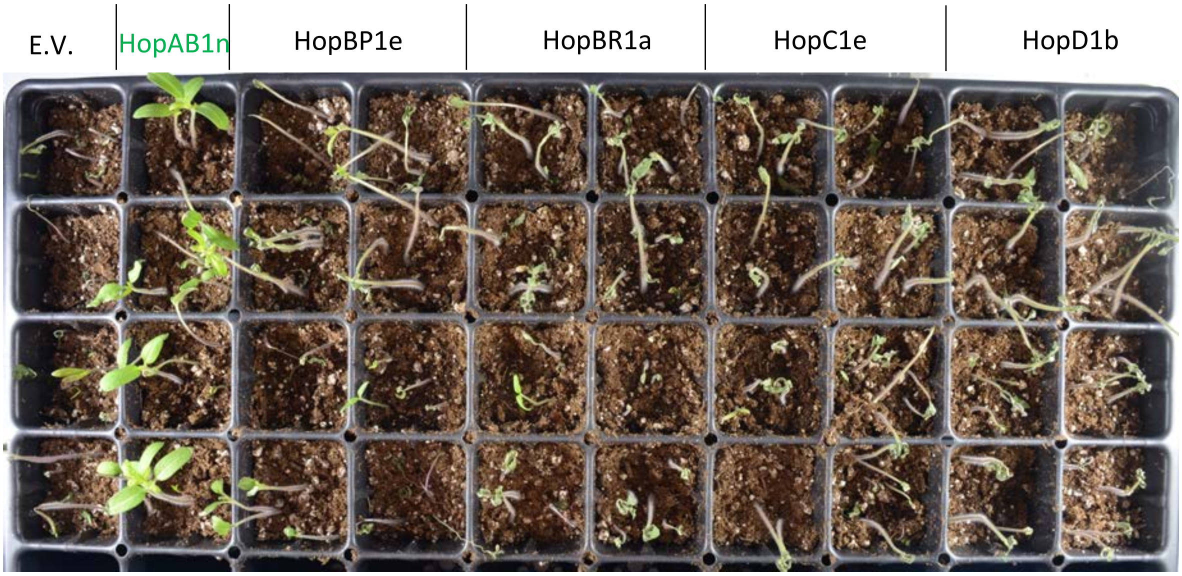

E.V. | HopAB1n | HopF1n | HopF1o | HopF1t | HopF1v

Exp 67

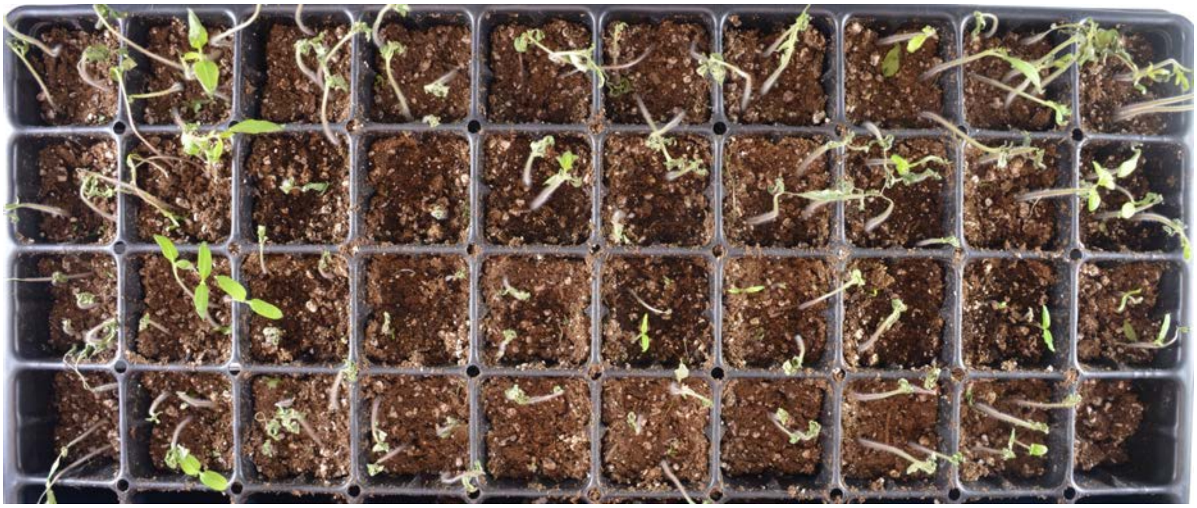

E.V. | HopAB1n | HopT1b | HopF1r | HopF1j | HopF3a

Exp 68

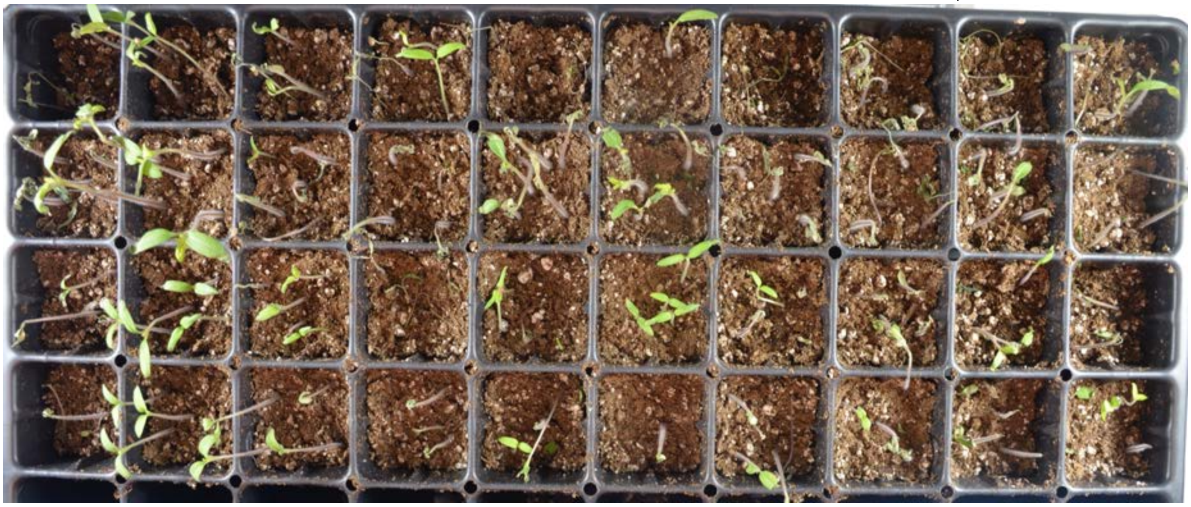

E.V. | HopAB1n | HopS2b | HopS2c | HopS2d | HopS2f

Exp 69

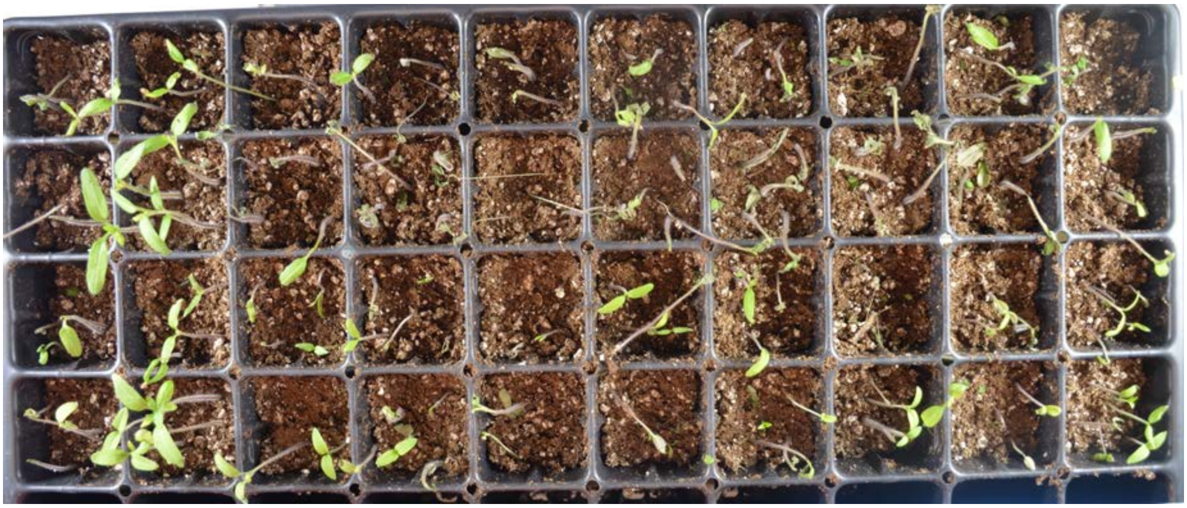

E.V. | HopAB1n | HopF1b | HopF1h | HopF1s | HopF1k

Exp 70

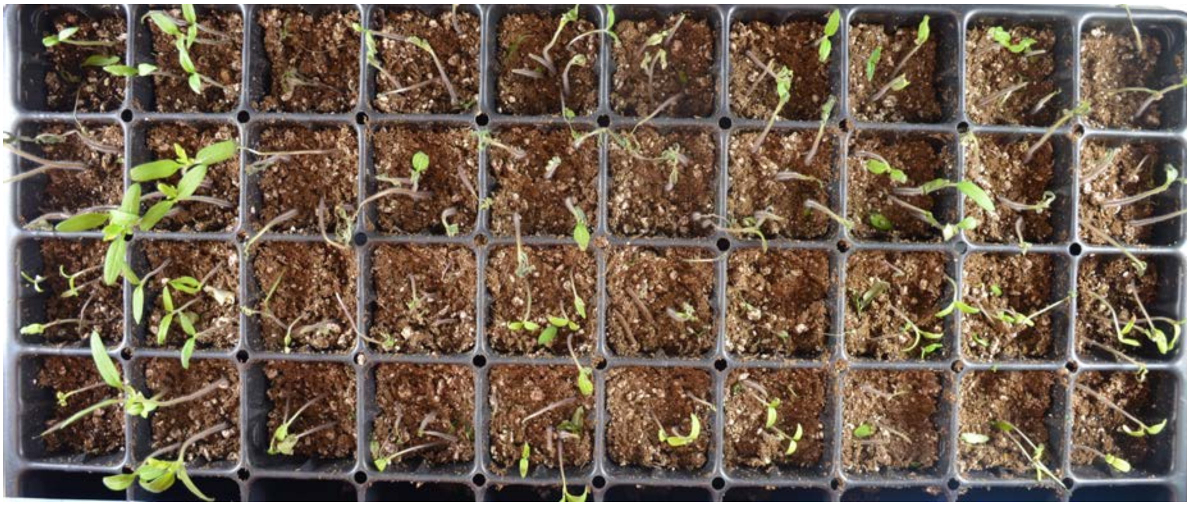

HopAB1n | E.V. | HopD2g | HopD2h | HopF1f | HopF3c

Exp 71

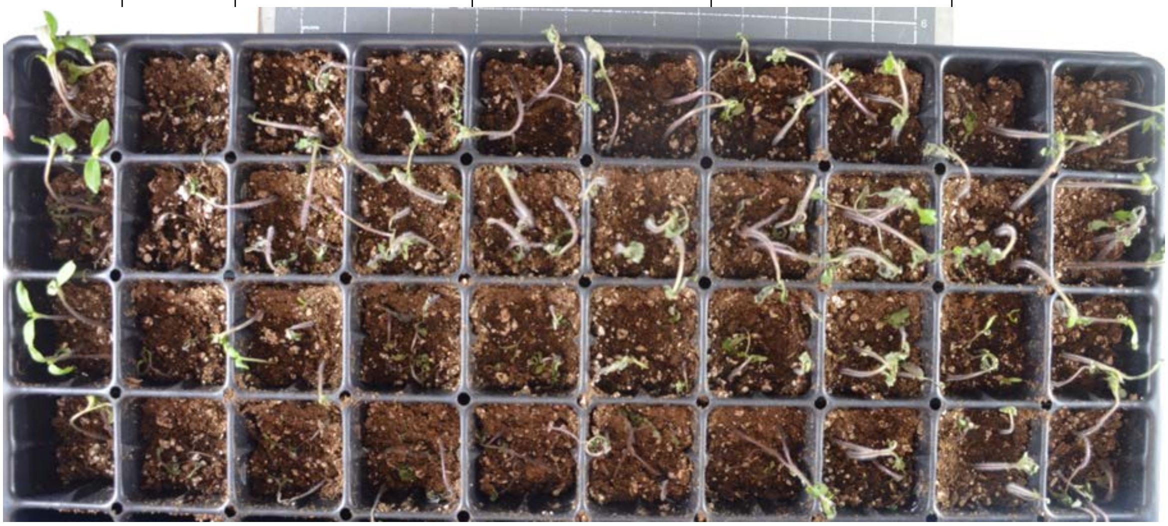

HopAB1n | E.V. | HopG1g | HopI1i | HopI1k | HopI1m

Exp 72

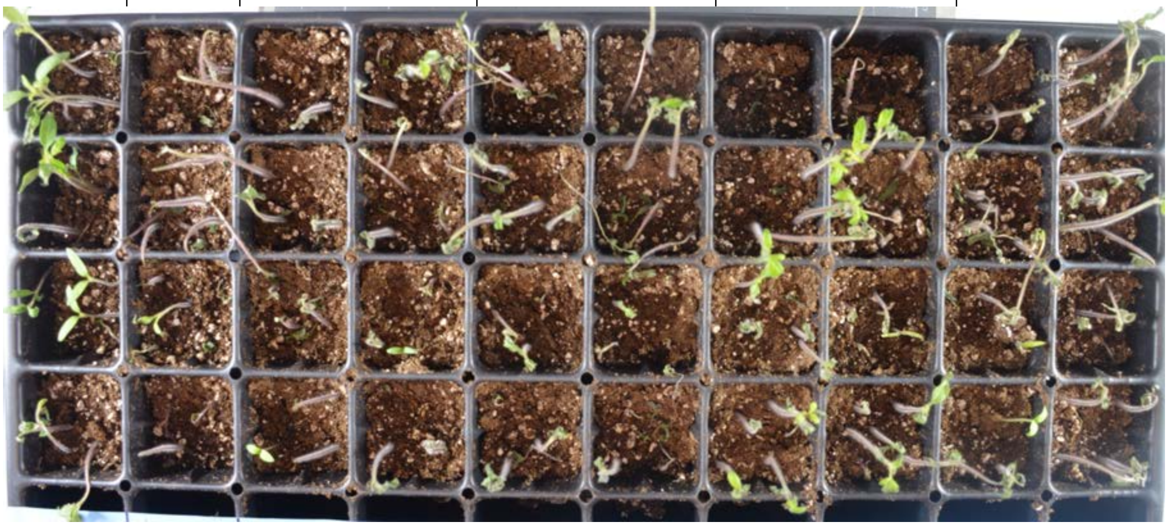

HopAB1n | E.V. | HopBN1a | HopBN1b | HopBN1c | HopBN1d

Exp 73

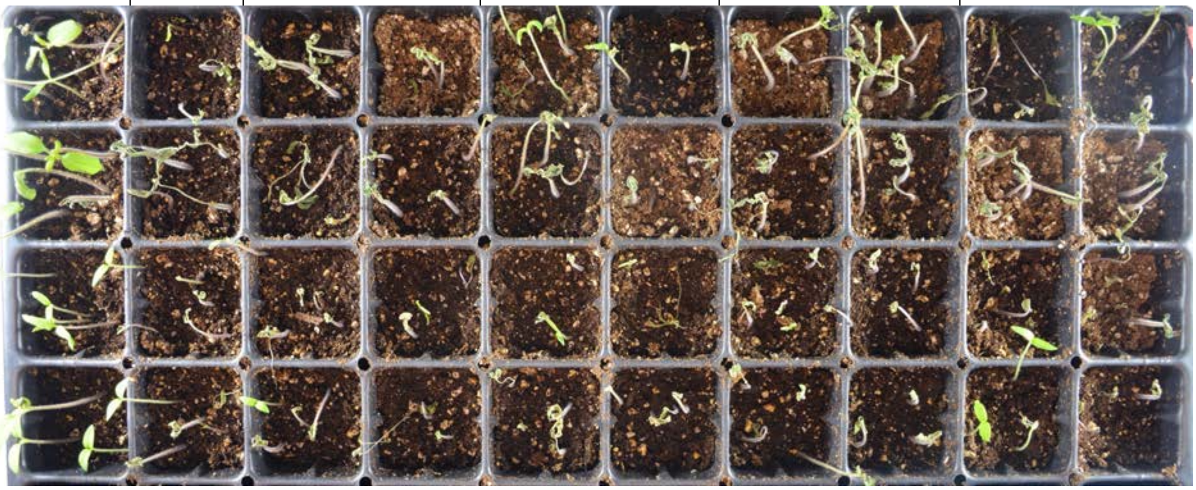

HopAB1n | E.V. | HopBN1e | HopI1s | HopK1c | HopM1ab

Exp 74

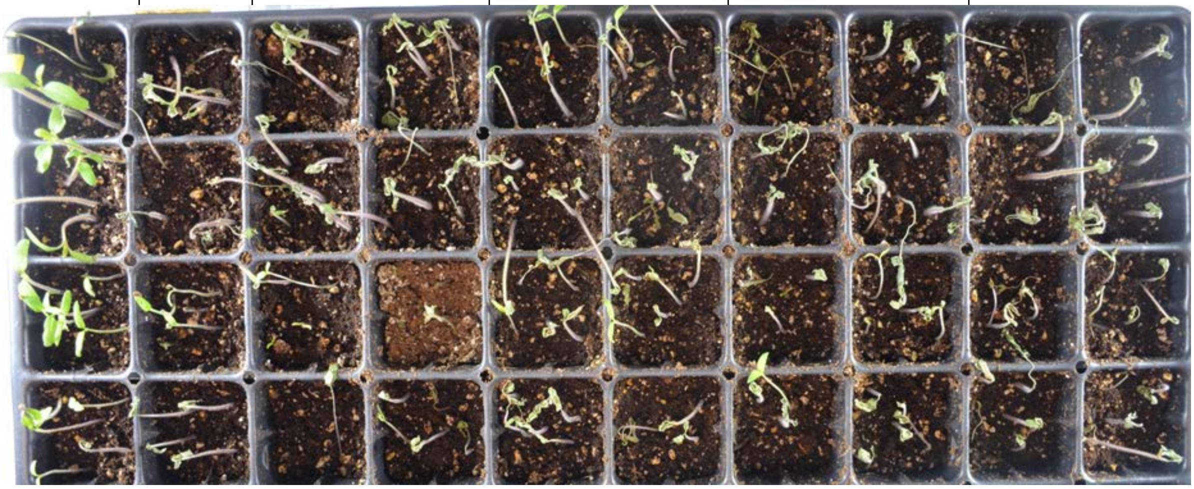

HopAB1n | E.V. | HopM1ad | HopN1b | XXXX | XXXX

Exp 75

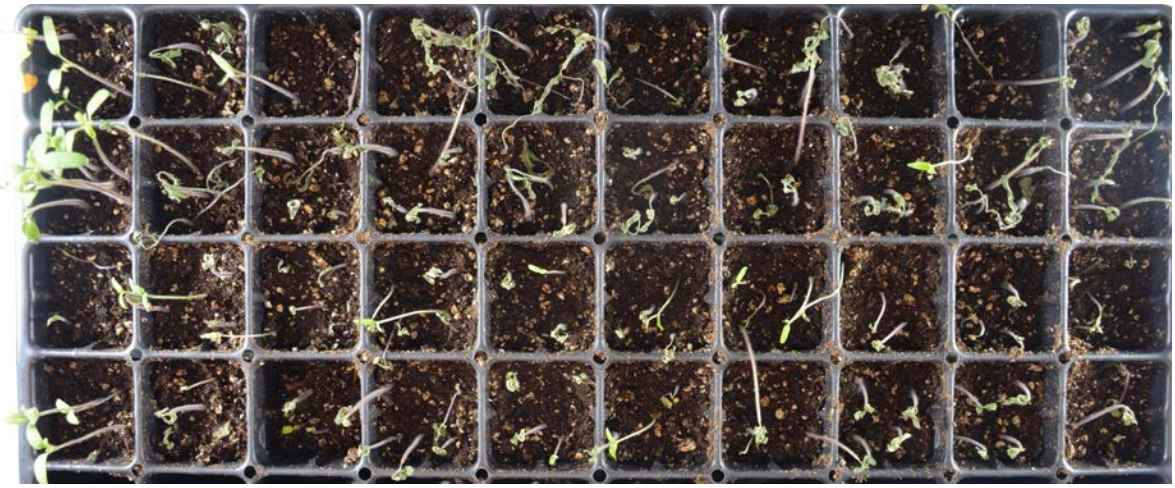

Exp 76

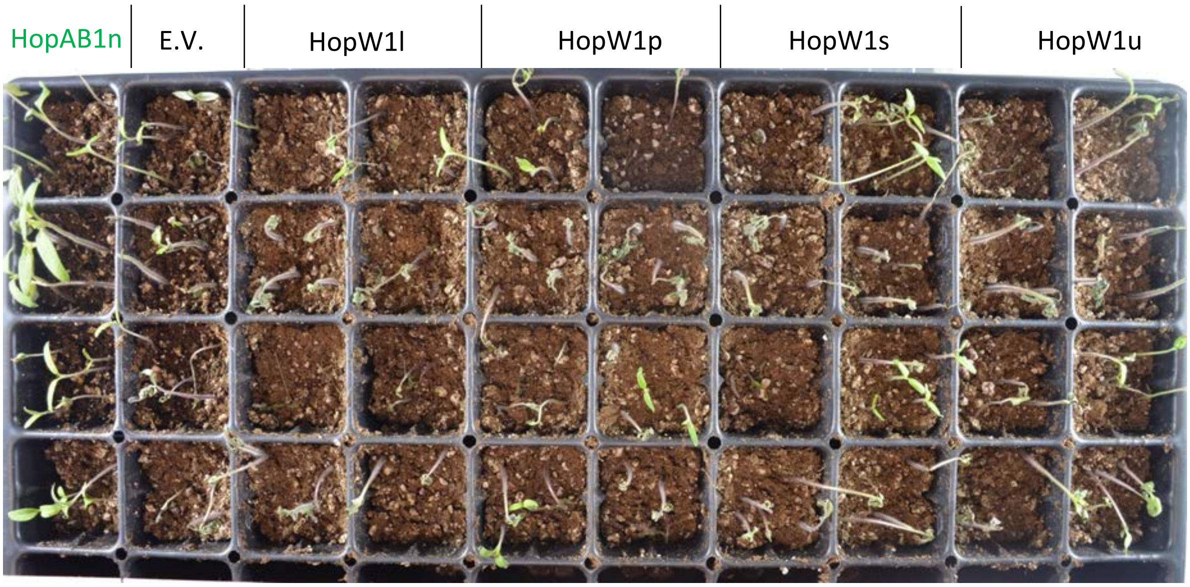

Exp 77

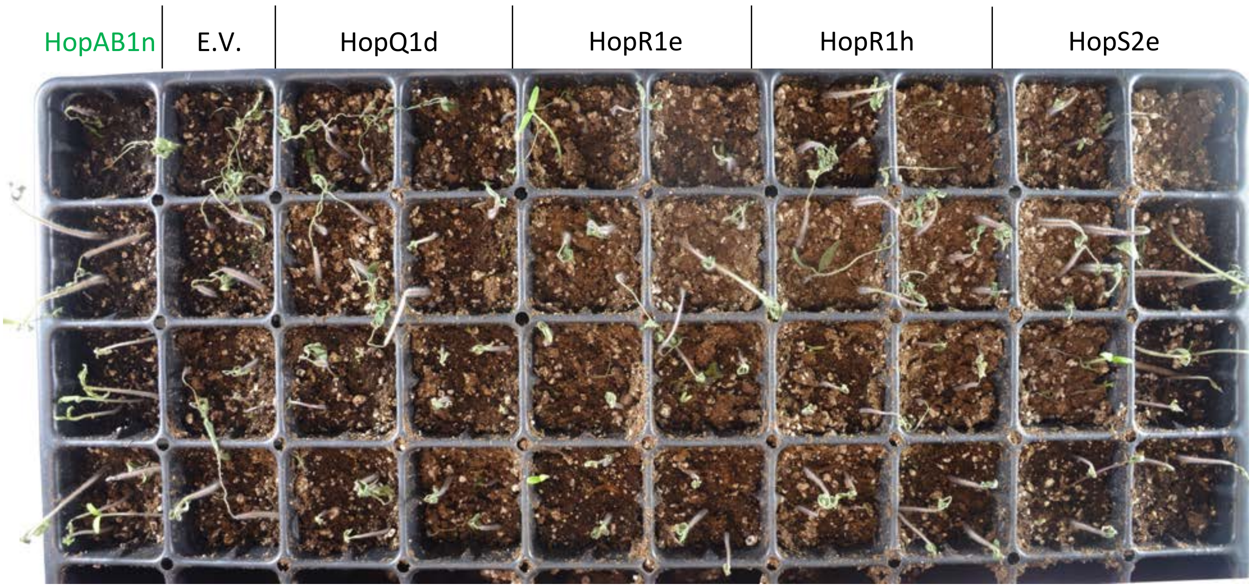

Exp 78

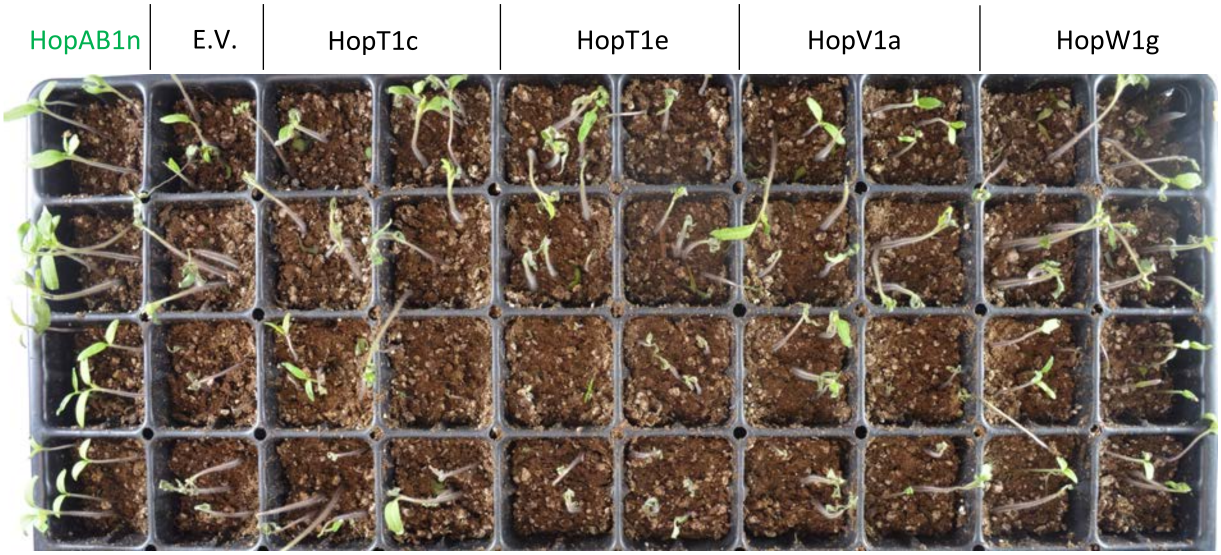

HopAB1n | E.V. | XXXX | XXX | XXXX | HopY1e

Exp 79

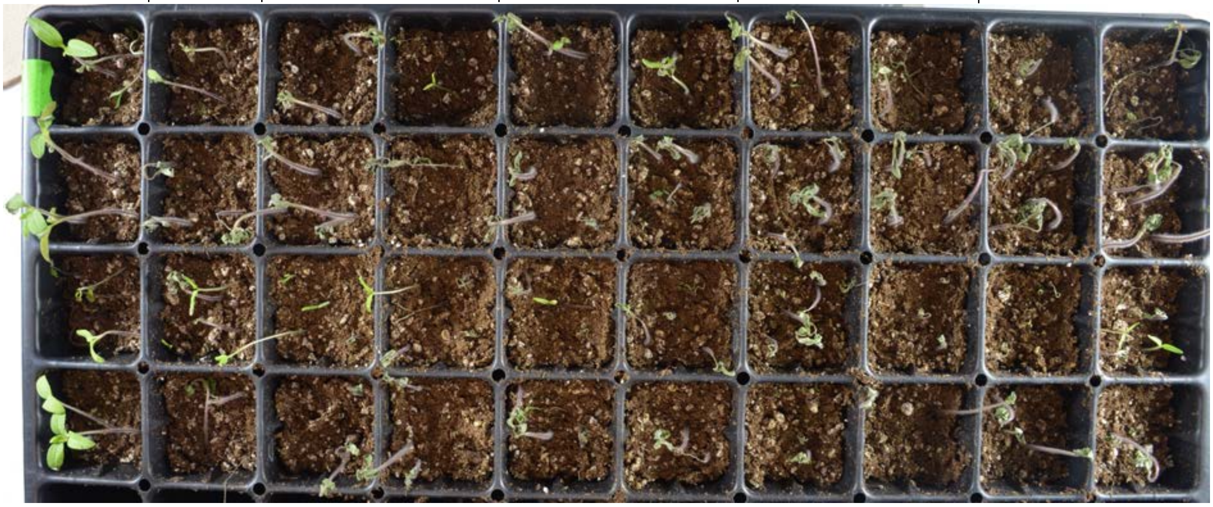

HopAB1n | E.V. | HopY1g | HopZ1h | HopZ5a | HopZ5b

Exp 80

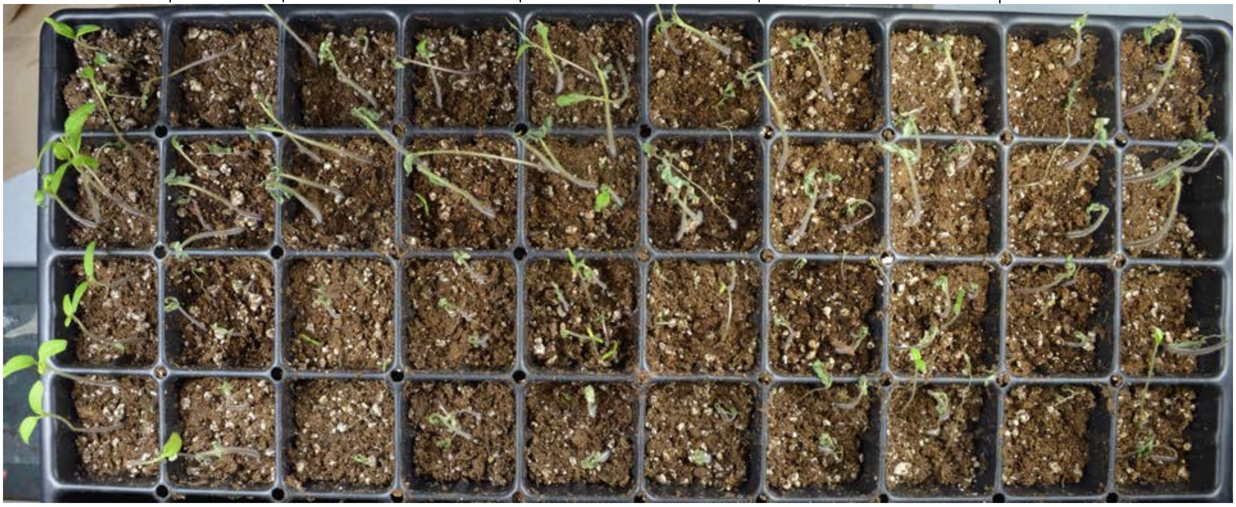

HopAB1n | E.V. | XXXX | HopAA1b | HopAA1e | XXXXX

Exp 81

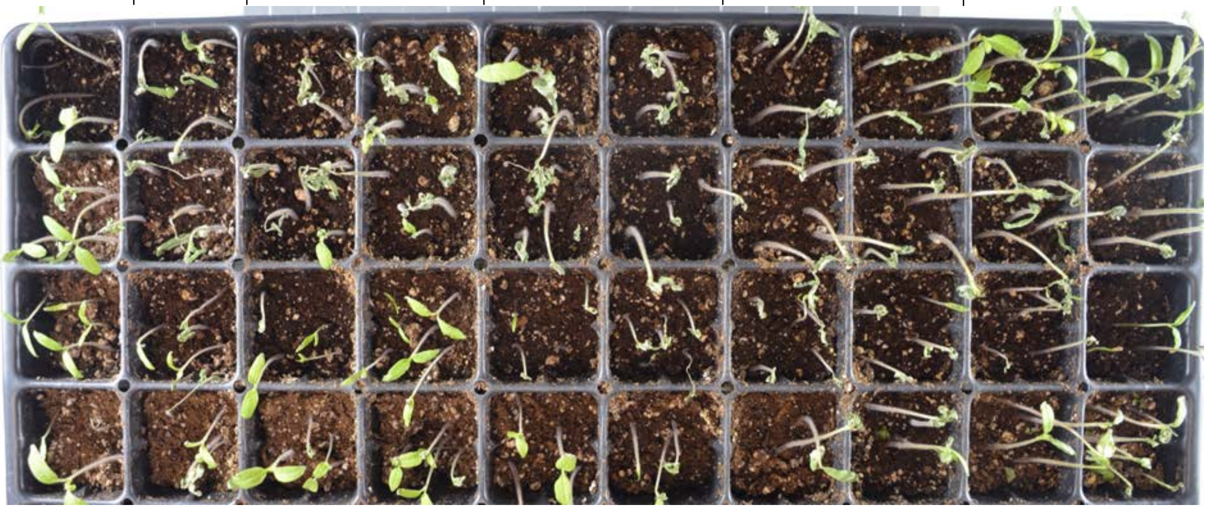

HopAB1n | E.V. | HopX1c | HopX1b | HopX1i | HopX1e

Exp 82

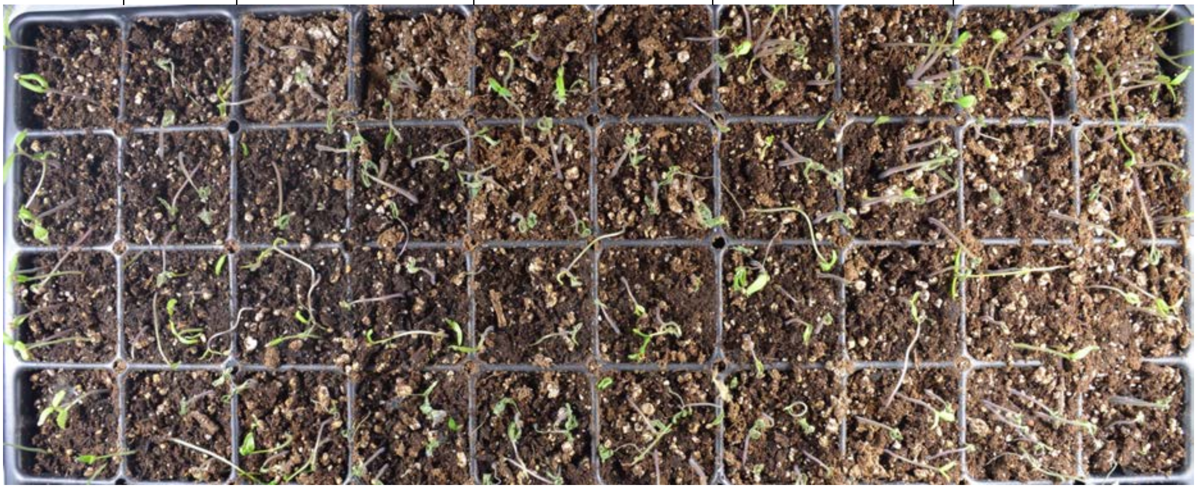

E.V. | HopAB1n | HopBJ1b | XXXXXX | AvrB1b | XXXXXXX

Exp 83

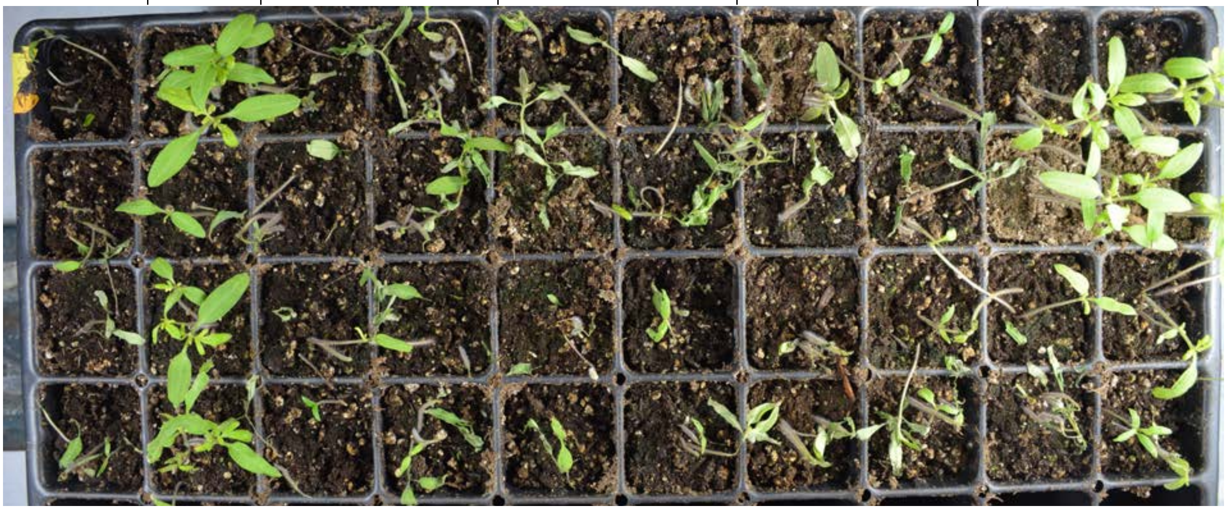

HopAB1n | E.V. | HopAR1f | HopAR1d | HopAR1h | HopC1c

Exp 84

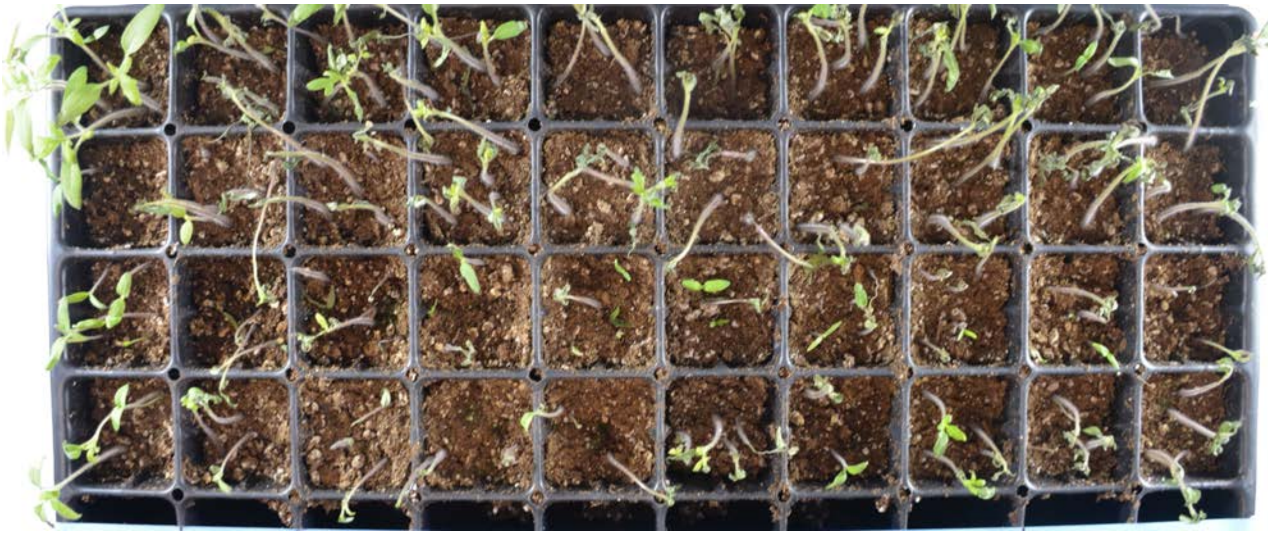

HopAB1n | E.V. | HopBL1a | HopBL1b | HopAT1c | HopAH1e

Exp 85

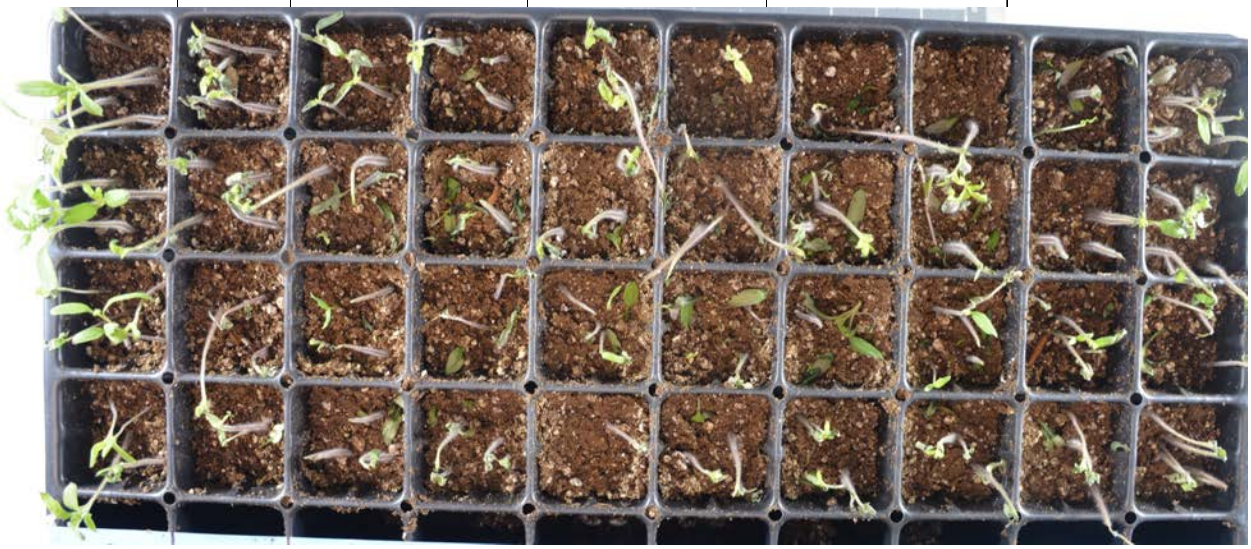

HopAB1n | E.V. | HopAA1a | XXXXXX | HopAA1v | HopB1aa

Exp 86

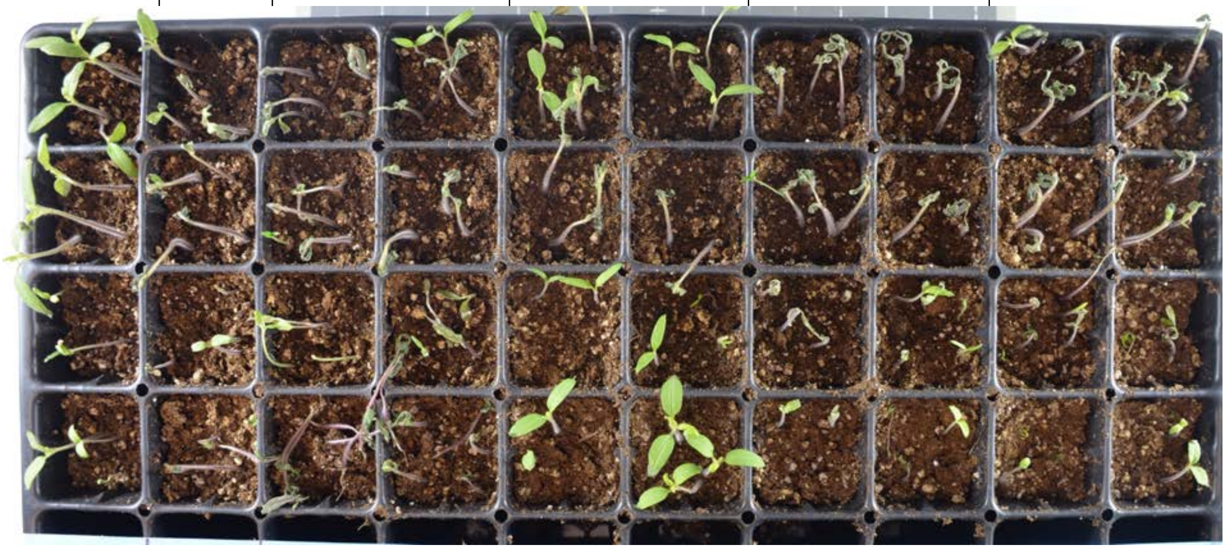

HopAB1n | E.V. | HopAQ1a | HopBD1d | HopBD1g | HopAB1l

Exp 87

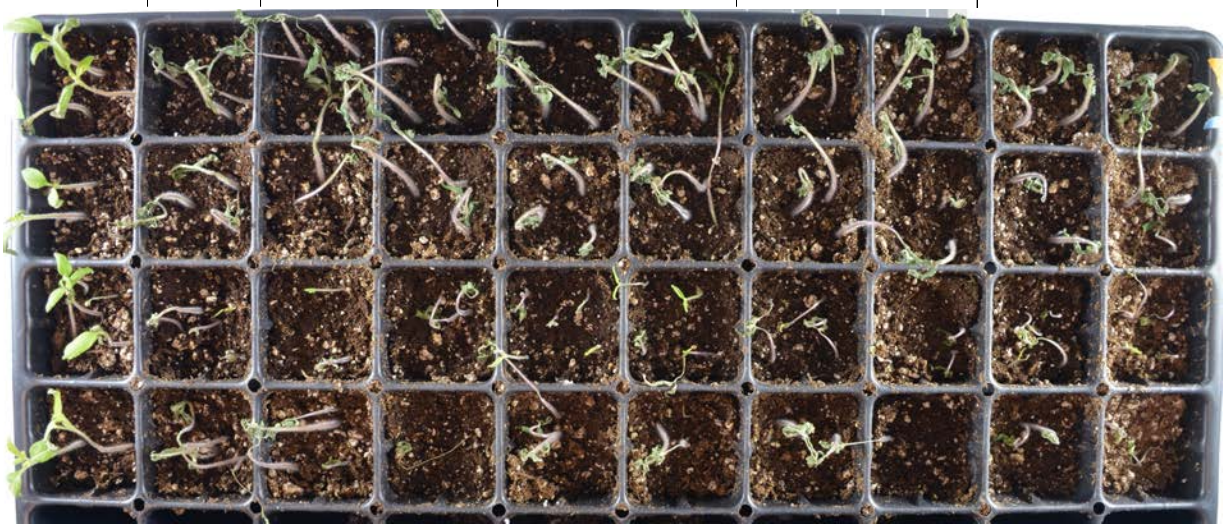

Exp 88

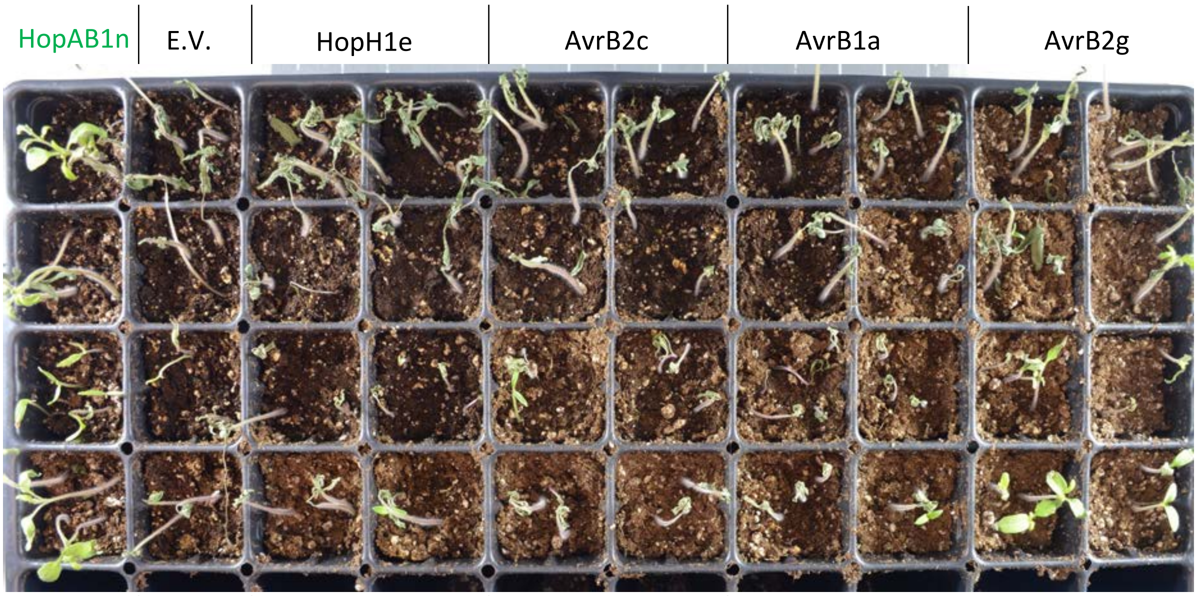

Exp 89

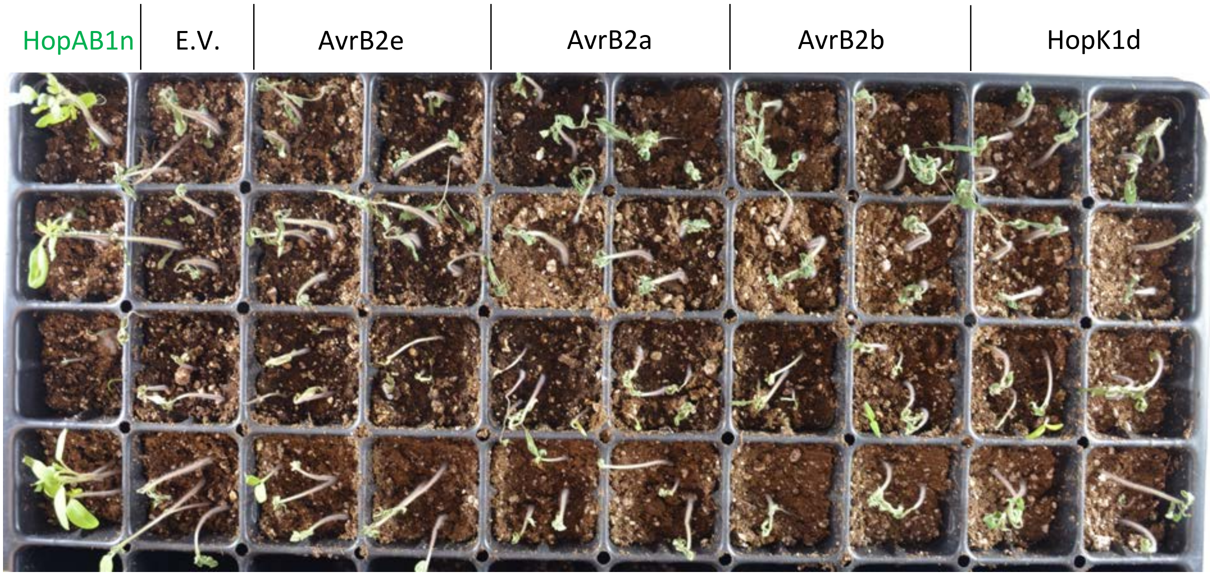

Exp 90

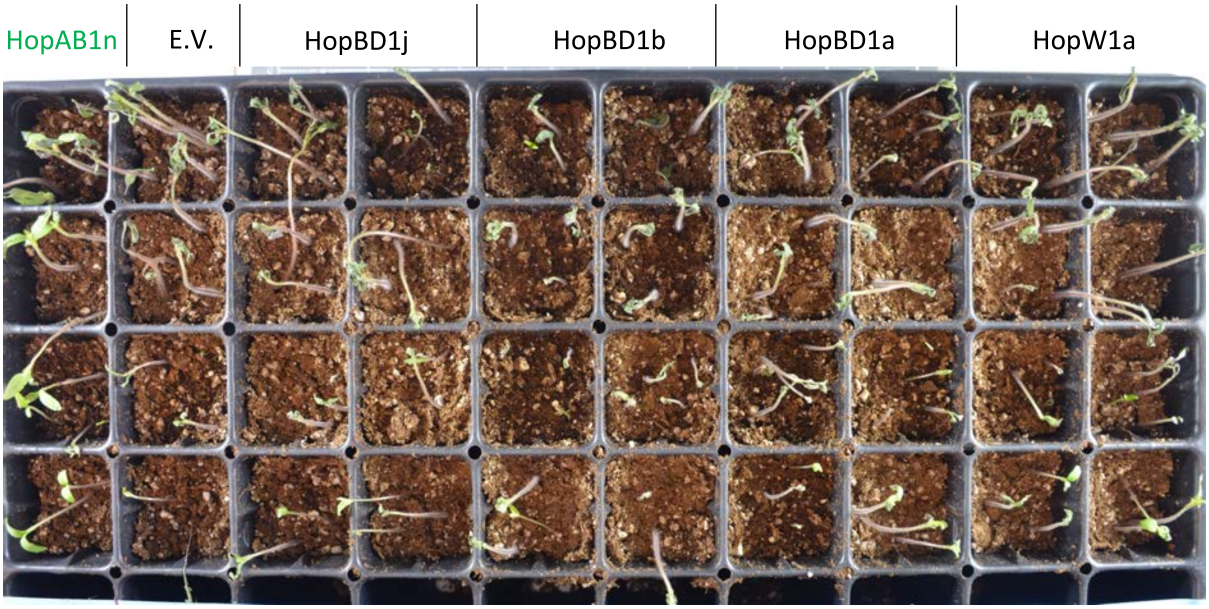

HopAB1n | E.V. | HopW1i | HopW1n | HopW1c | HopW1b

Exp 91

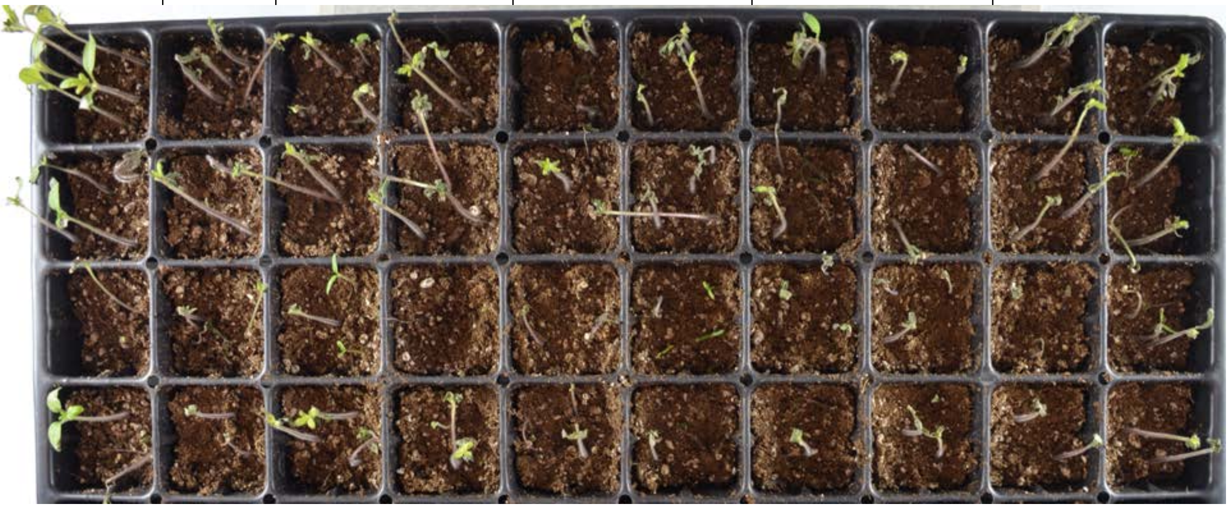

E.V. | HopAB1n | AvrE1e | AvrE1k | AvrE1h | AvrE1g

Exp 92

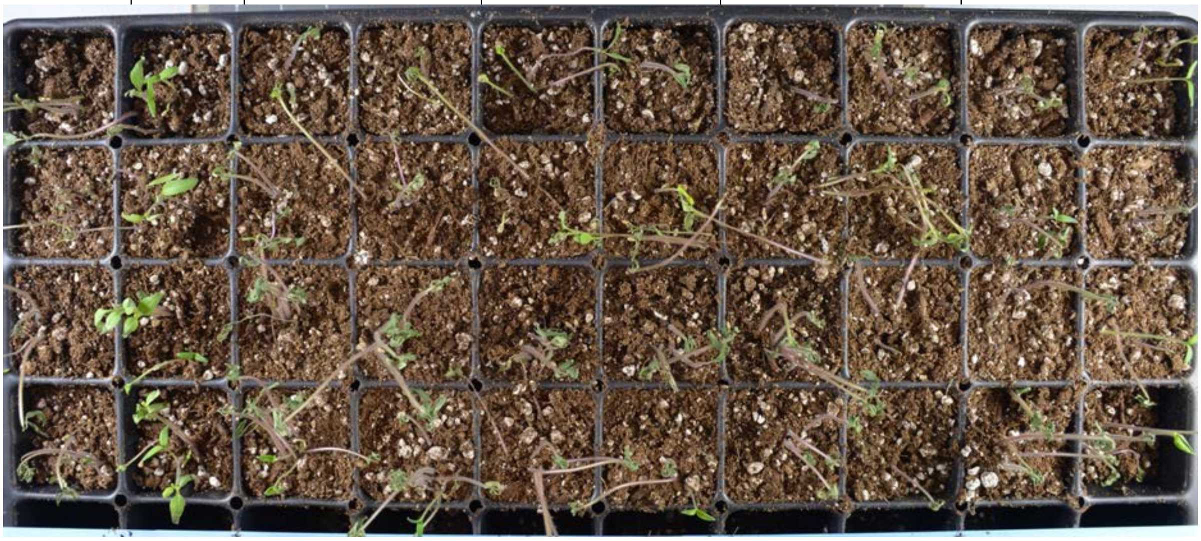

HopAB1n | E.V. | HopC1d | HopC1a | HopC1b | HopAW1a

Exp 93

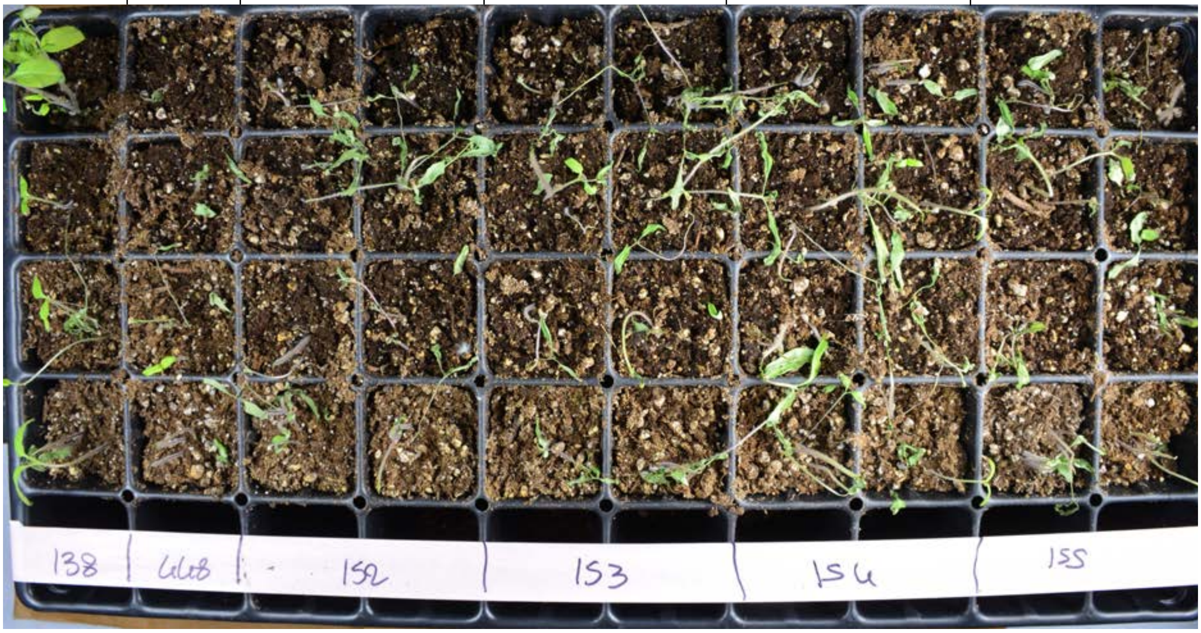

HopAB1n	E.V.	HopAT1b	HopAH1b	HopAH1c	HopAH1k

Exp 94

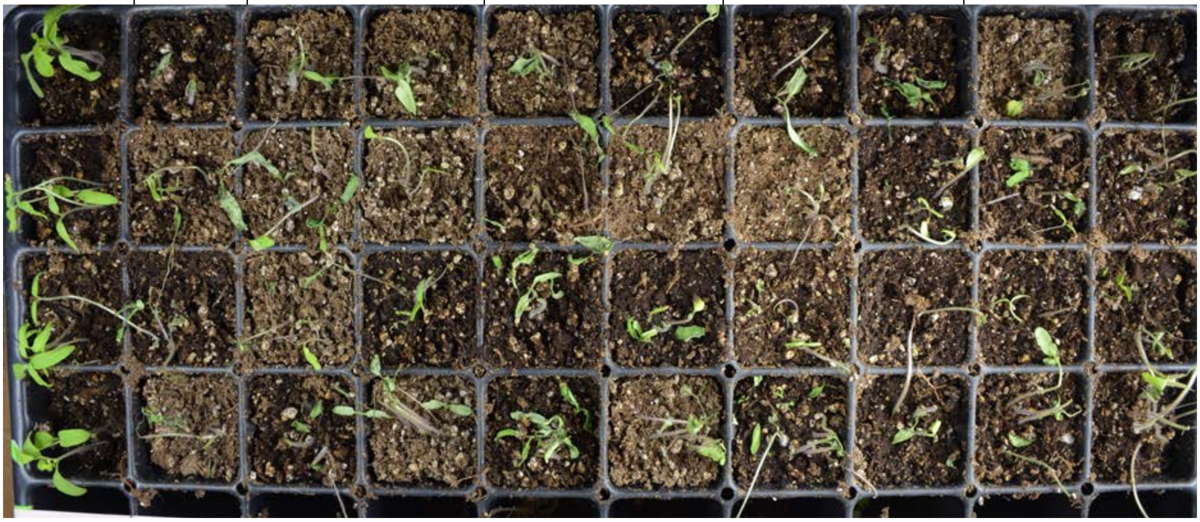

HopAB1n	E.V.	HopAH1q	HopAH1h	HopH1c	HopH1a

Exp 95

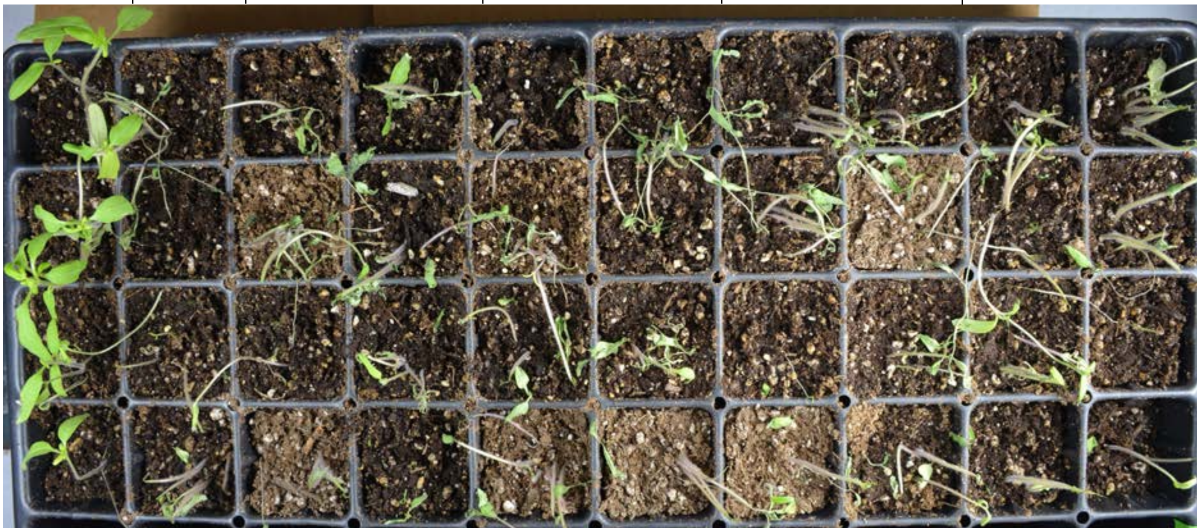

HopAB1n	E.V.	HopAB1p	HopAB1e	HopH1d	HopK1a

Exp 96

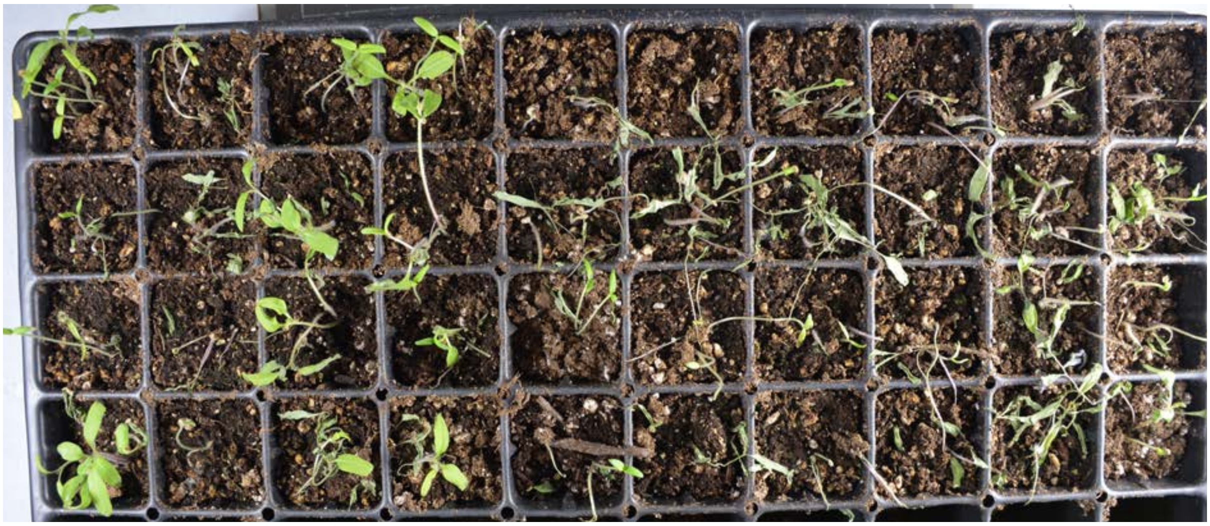

HopAB1n | E.V. | HopAB1g | HopAB1d | HopAB1a | HopAB1o

Exp 97

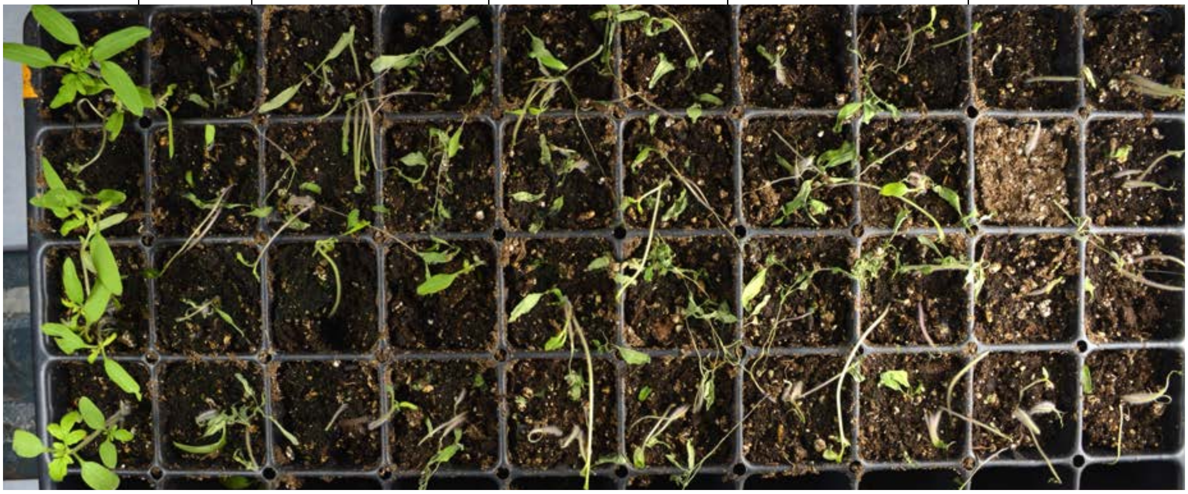

HopAB1n | E.V. | HopAB1r | HopAB1i | HopAB1s | HopAB1j

Exp 98

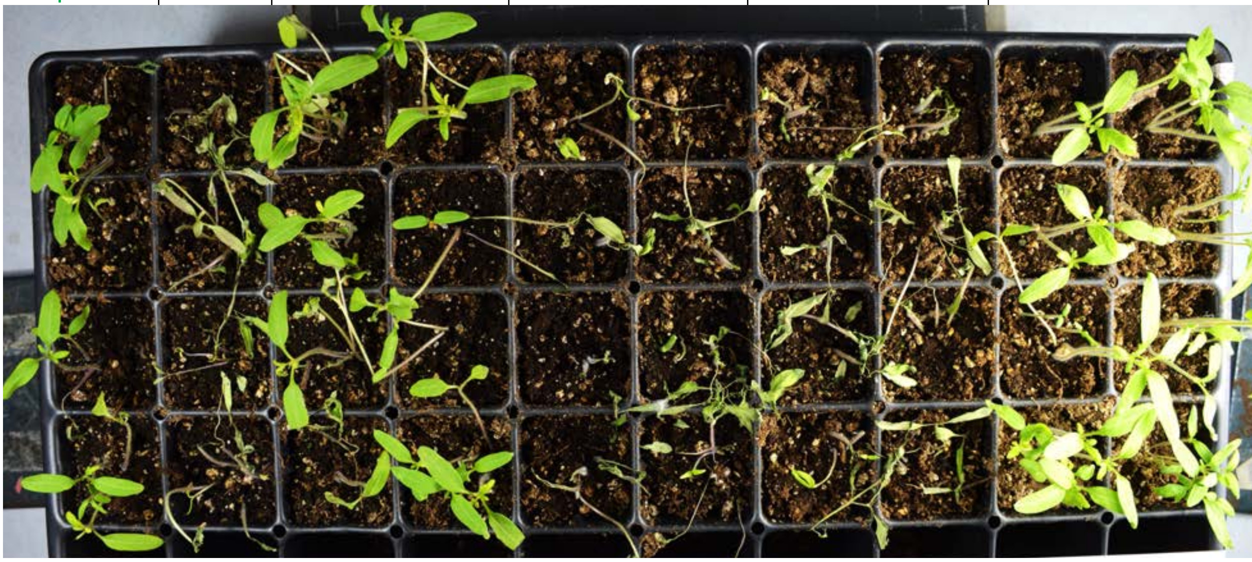

HopAB1n | E.V. | HopK1a | HopK1b | HopW1e | HopW1m

Exp 99

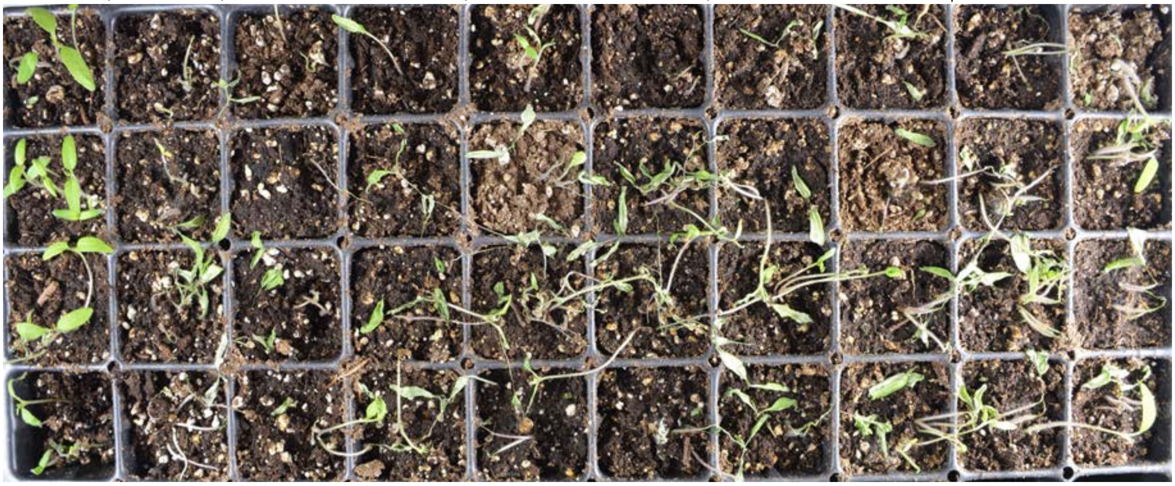

Exp 100

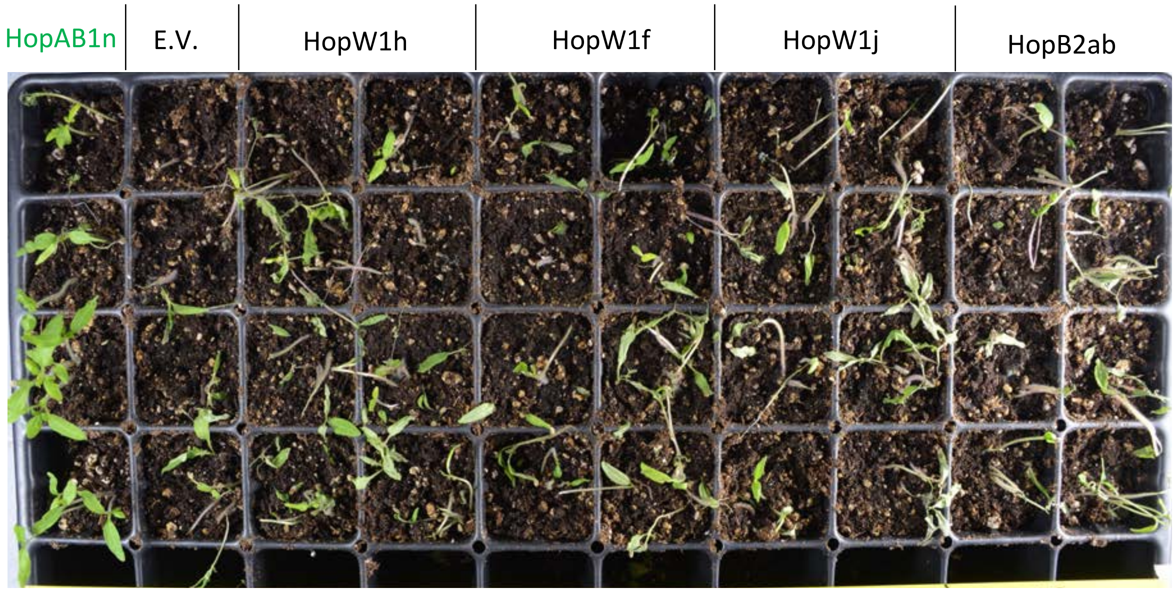

Exp 101

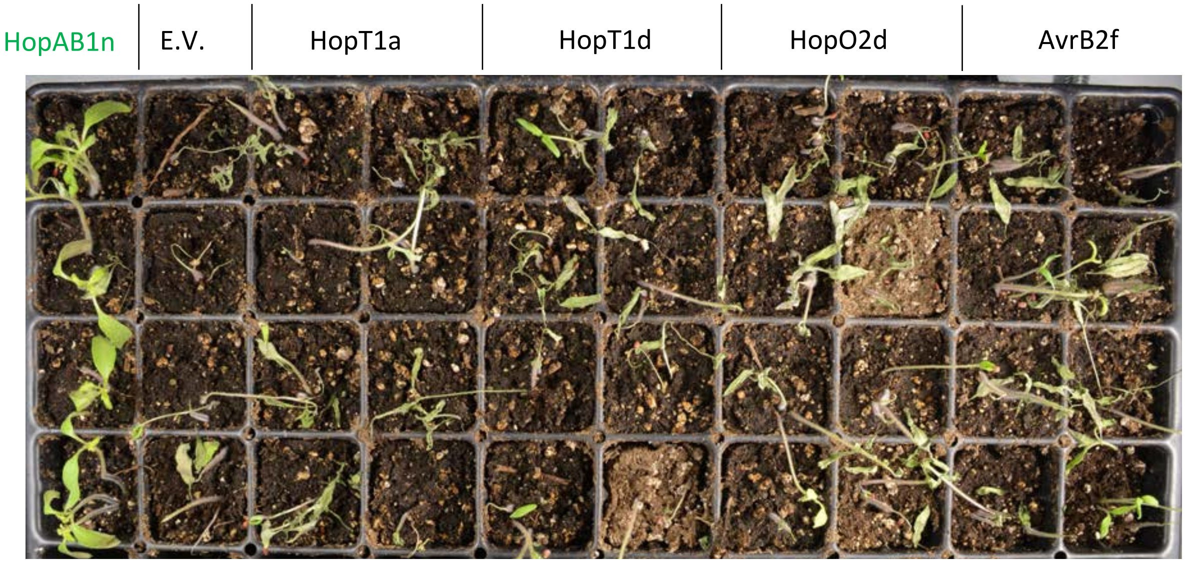

Exp 102

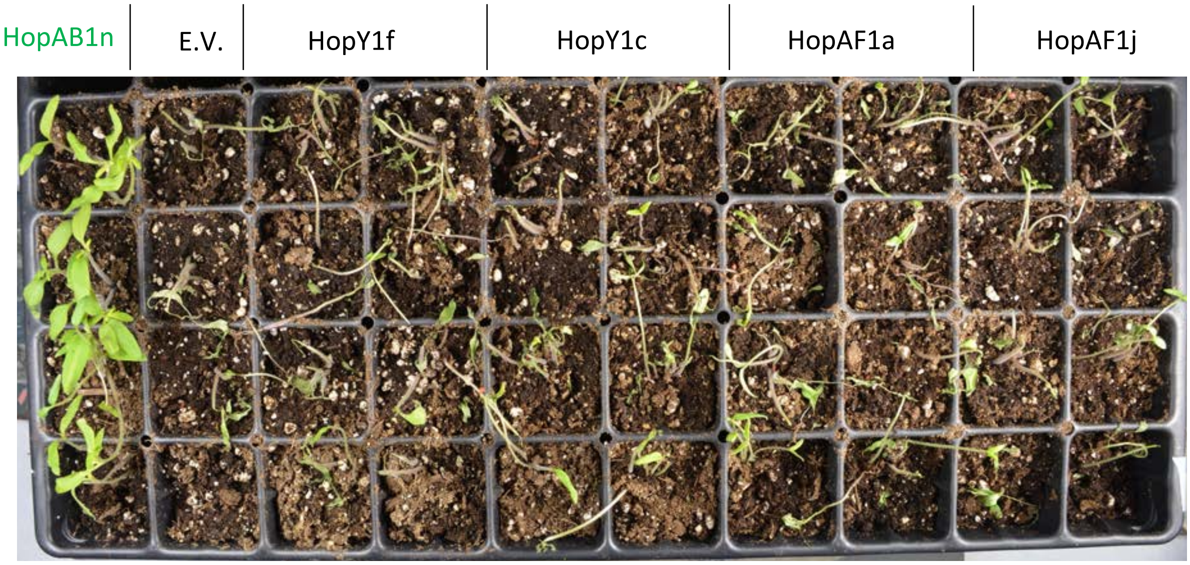

HopAB1n | E.V. | HopAB1ab | HopAB1k | HopAB1q

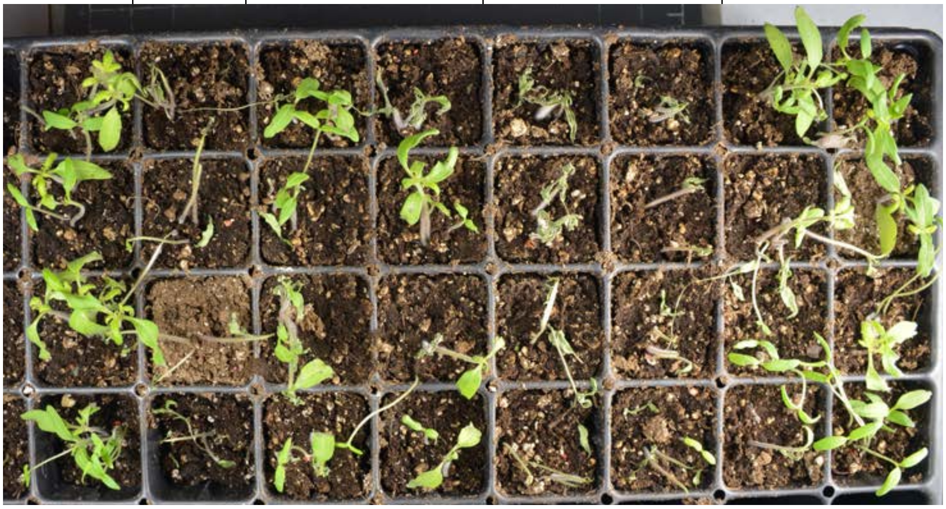

Exp 103

HopAB1n | E.V. | HopAB1u | HopAB1v | HopAB1w | HopAF1g

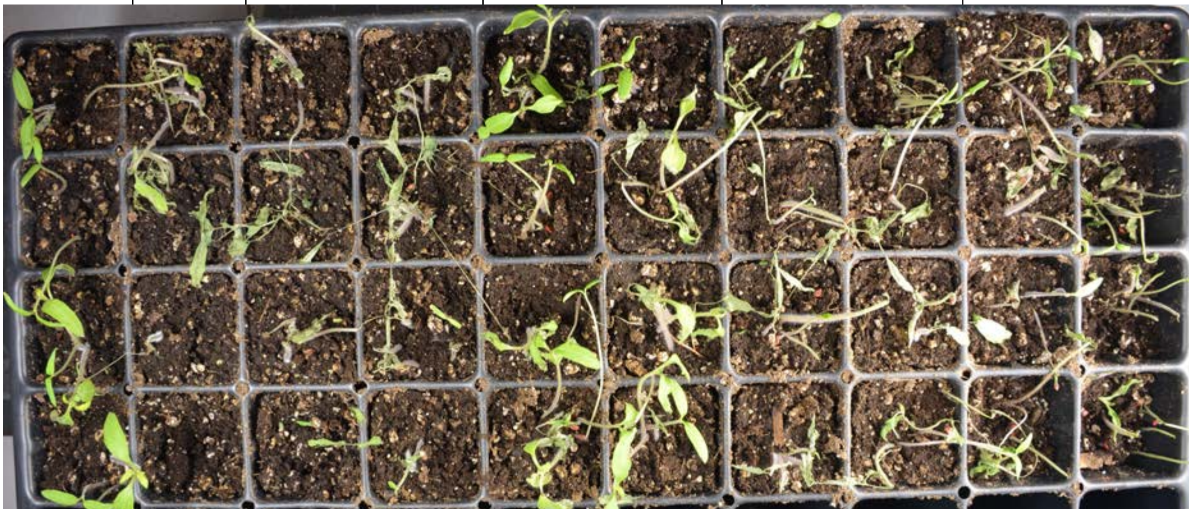

Exp 104

HopAB1n | E.V. | HopAF1i | HopAH1aa | HopBC1a | XXXXXX

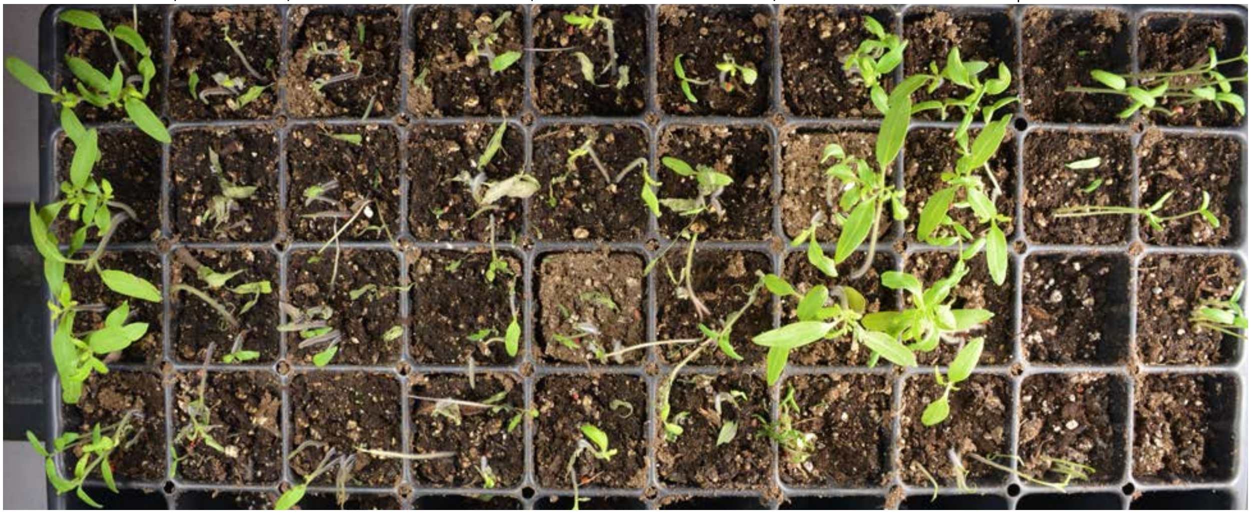

Exp 105

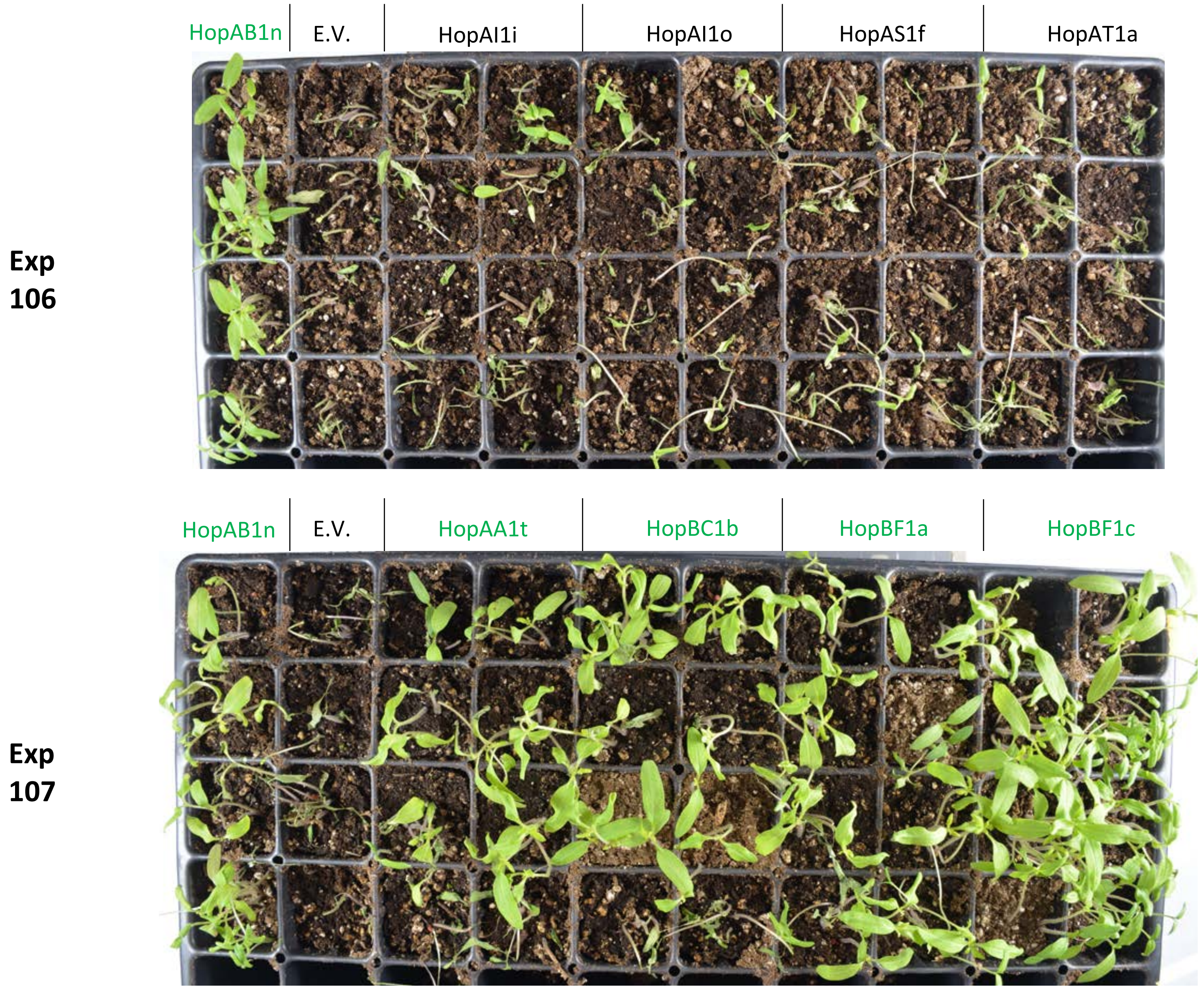

Supplementary Figure 4. Compiled images of primary ETI screening of PsyTEC in tomato vars. Vendor, Cherokee Purple, SubArctic, and Moskvich. Compiled images of PsyTEC screen in four tomato accessions, tomato vars. Vendor, Cherokee Purple, SubArctic, and Moskvich. For each experiment, the top row corresponds to tomato Vendor, followed by Cherokee Purple, SubArctic, and Moskvich. Each experiment included a positive ETI control, HopAB1n, and a negative ETI control, Empty Vector. Images were taken 6-10 days post-inoculation. PtoDC3000 strains carrying PsyTEC alleles are indicated above each flat. Green labels indicate that an ETI is observed for at least one accession.

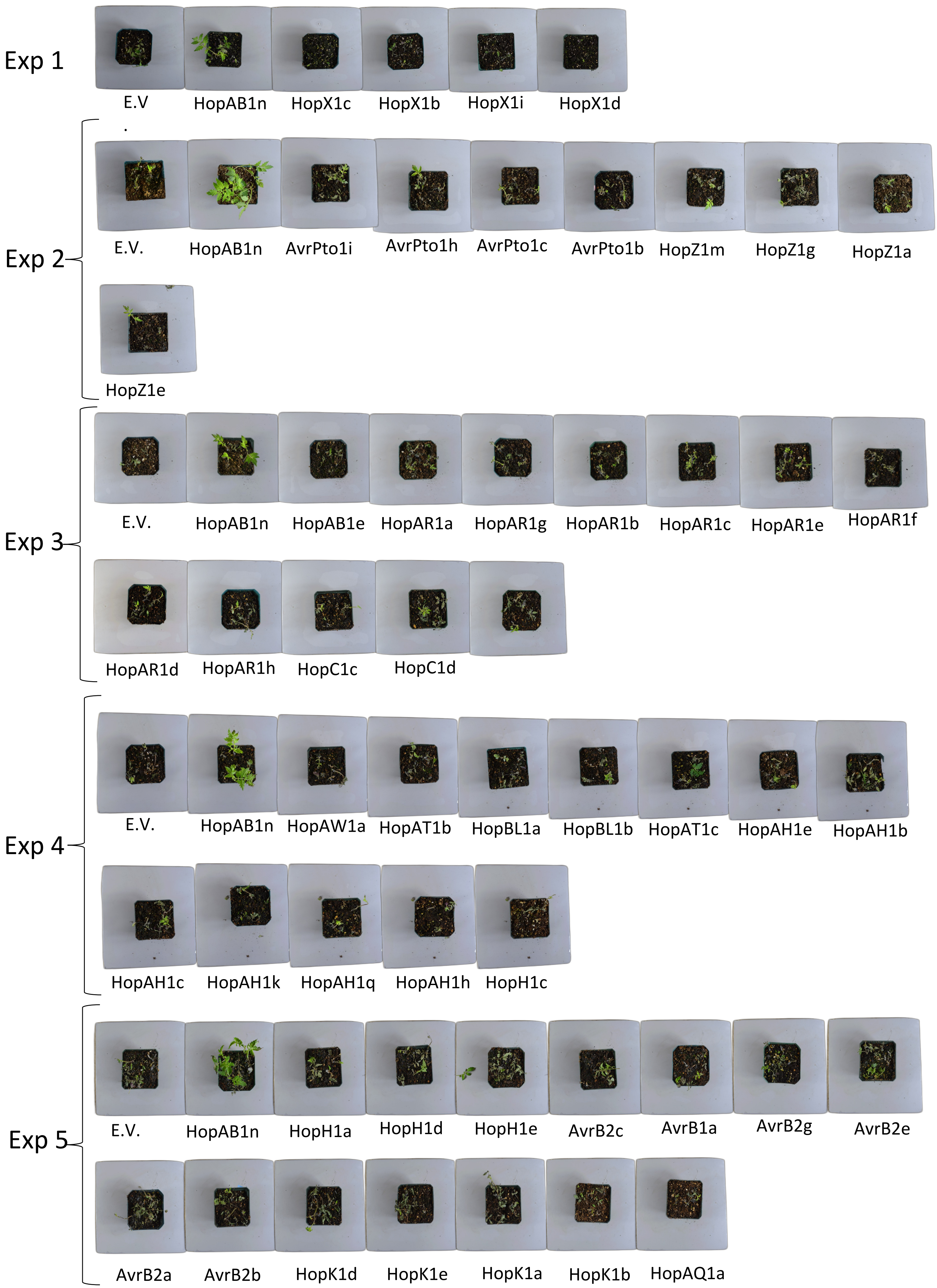
Exp 1
E.V
HopAB1n
HopX1c
HopX1b
HopX1i
HopX1d
Exp 2
E.V.
HopAB1n
AvrPto1i
AvrPto1h
AvrPto1c
AvrPto1b
HopZ1m
HopZ1g
HopZ1a
HopZ1e
Exp 3
E.V.
HopAB1n
HopAB1e
HopAR1a
HopAR1g
HopAR1b
HopAR1c
HopAR1e
HopAR1f
HopAR1d
HopAR1h
HopC1c
HopC1d
Exp 4
E.V.
HopAB1n
HopAW1a
HopAT1b
HopBL1a
HopBL1b
HopAT1c
HopAH1e
HopAH1b
HopAH1c
HopAH1k
HopAH1q
HopAH1h
HopH1c
Exp 5
E.V.
HopAB1n
HopH1a
HopH1d
HopH1e
AvrB2c
AvrB1a
AvrB2g
AvrB2e
AvrB2a
AvrB2b
HopK1d
HopK1e
HopK1a
HopK1b
HopAQ1a

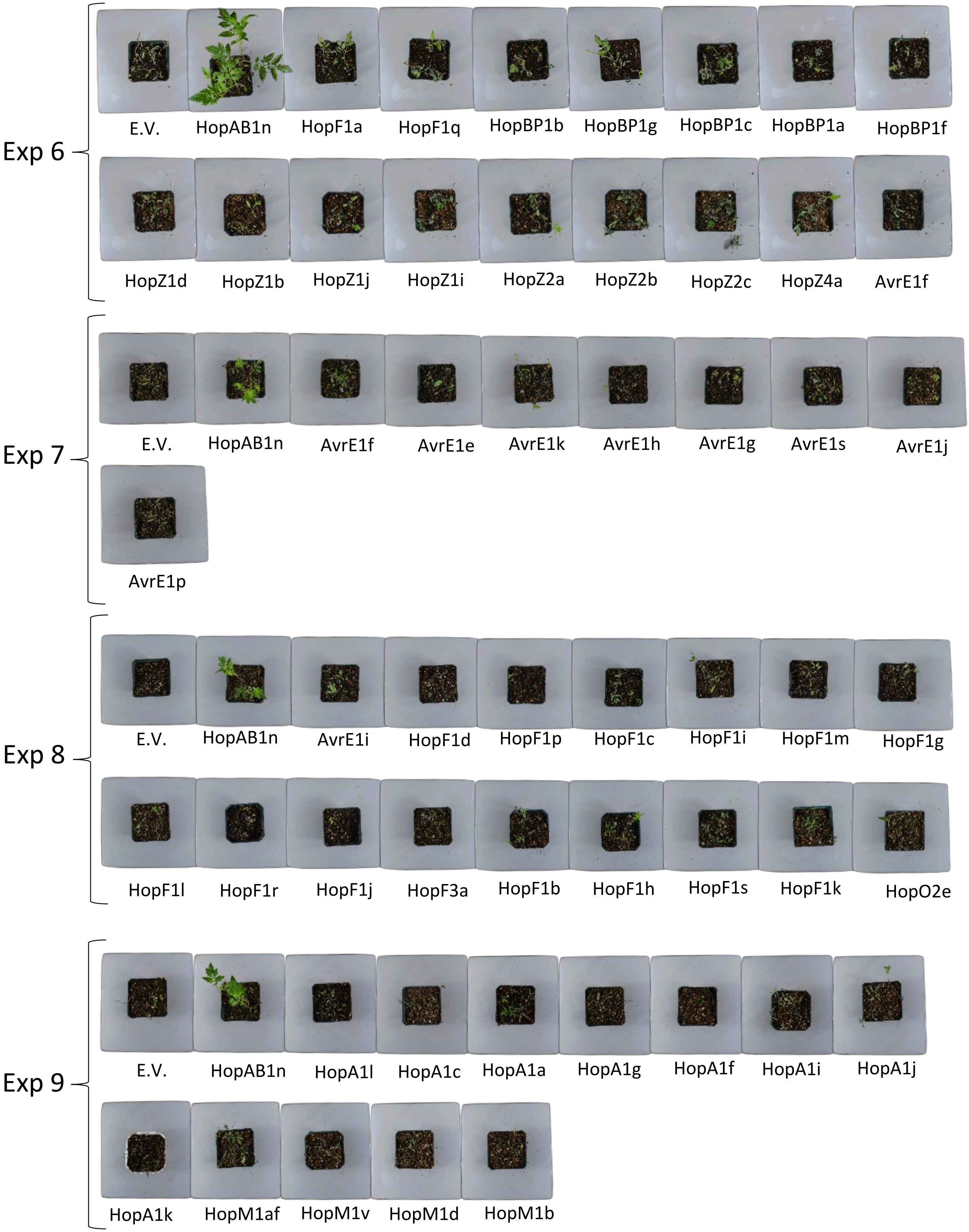
Exp 6
E.V.
HopAB1n
HopF1a
HopF1q
HopBP1b
HopBP1g
HopBP1c
HopBP1a
HopBP1f
HopZ1d
HopZ1b
HopZ1j
HopZ1i
HopZ2a
HopZ2b
HopZ2c
HopZ4a
AvrE1f
Exp 7
E.V.
HopAB1n
AvrE1f
AvrE1e
AvrE1k
AvrE1h
AvrE1g
AvrE1s
AvrE1j
AvrE1p
Exp 8
E.V.
HopAB1n
AvrE1i
HopF1d
HopF1p
HopF1c
HopF1i
HopF1m
HopF1g
HopF1l
HopF1r
HopF1j
HopF3a
HopF1b
HopF1h
HopF1s
HopF1k
HopO2e
Exp 9
E.V.
HopAB1n
HopA1l
HopA1c
HopA1a
HopA1g
HopA1f
HopA1i
HopA1j
HopA1k
HopM1af
HopM1v
HopM1d
HopM1b

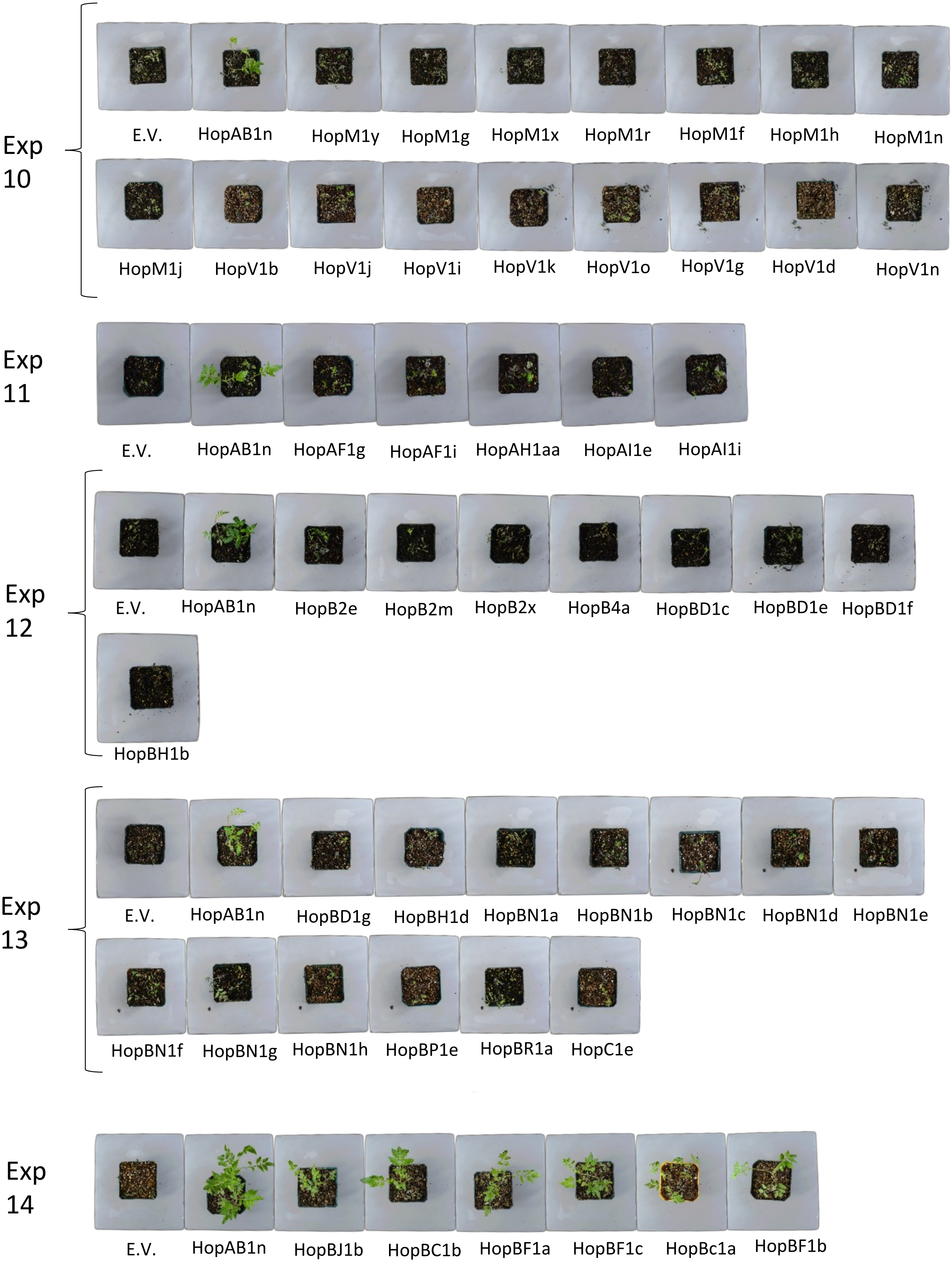
Exp 10
E.V.
HopAB1n
HopM1y
HopM1g
HopM1x
HopM1r
HopM1f
HopM1h
HopM1n
HopM1j
HopV1b
HopV1j
HopV1i
HopV1k
HopV1o
HopV1g
HopV1d
HopV1n
Exp 11
E.V.
HopAB1n
HopAF1g
HopAF1i
HopAH1aa
HopAI1e
HopAI1i
Exp 12
E.V.
HopAB1n
HopB2e
HopB2m
HopB2x
HopB4a
HopBD1c
HopBD1e
HopBD1f
HopBH1b
Exp 13
E.V.
HopAB1n
HopBD1g
HopBH1d
HopBN1a
HopBN1b
HopBN1c
HopBN1d
HopBN1e
HopBN1f
HopBN1g
HopBN1h
HopBP1e
HopBR1a
HopC1e
Exp 14
E.V.
HopAB1n
HopBJ1b
HopBC1b
HopBF1a
HopBF1c
HopBc1a
HopBF1b

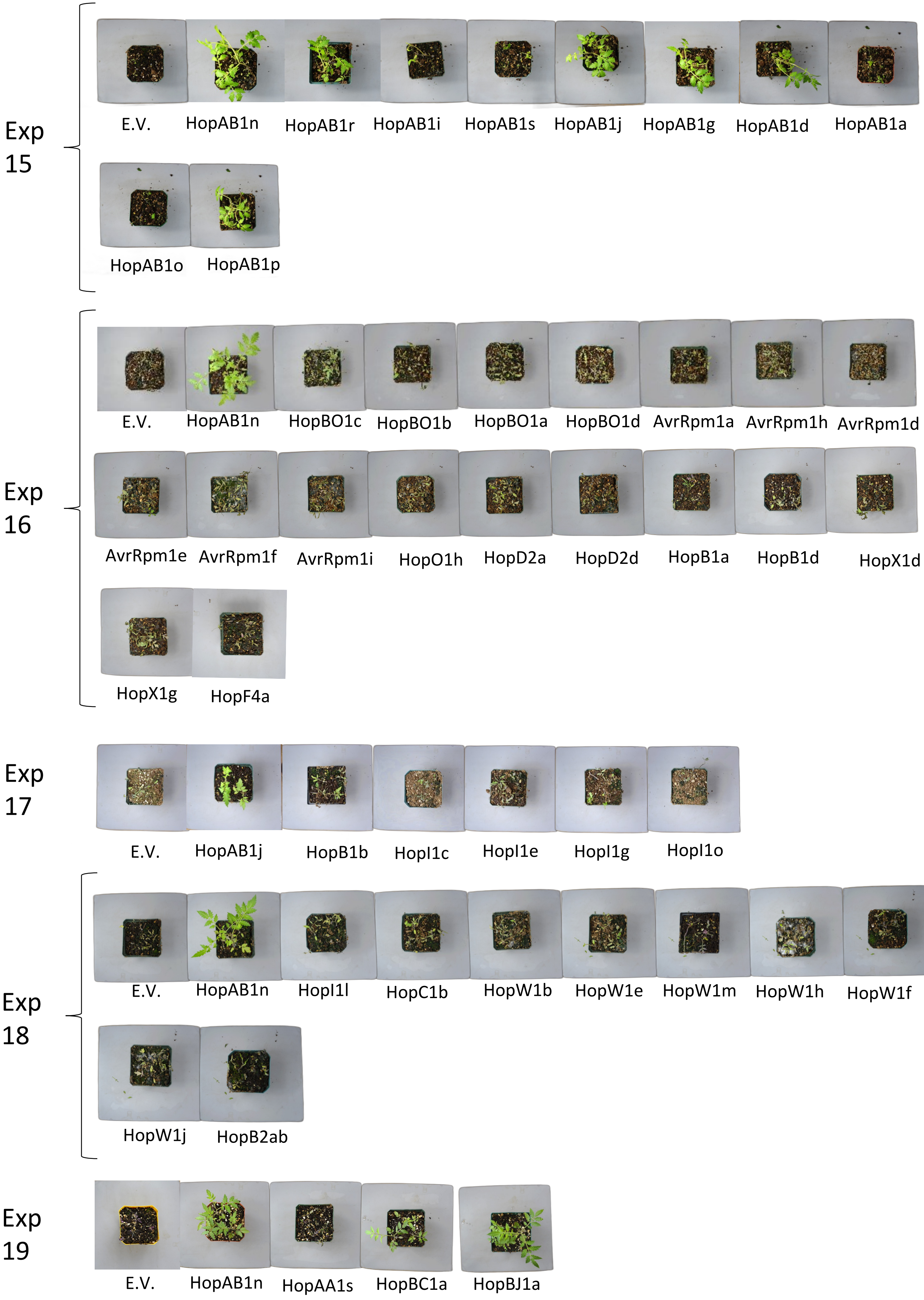
Exp 15
E.V.
HopAB1n
HopAB1r
HopAB1i
HopAB1s
HopAB1j
HopAB1g
HopAB1d
HopAB1a
HopAB1o
HopAB1p
Exp 16
E.V.
HopAB1n
HopBO1c
HopBO1b
HopBO1a
HopBO1d
AvrRpm1a
AvrRpm1h
AvrRpm1d
AvrRpm1e
AvrRpm1f
AvrRpm1i
HopO1h
HopD2a
HopD2d
HopB1a
HopB1d
HopX1d
HopX1g
HopF4a
Exp 17
E.V.
HopAB1j
HopB1b
HopI1c
HopI1e
HopI1g
HopI1o
Exp 18
E.V.
HopAB1n
HopI1l
HopC1b
HopW1b
HopW1e
HopW1m
HopW1h
HopW1f
HopW1j
HopB2ab
Exp 19
E.V.
HopAB1n
HopAA1s
HopBC1a
HopBJ1a

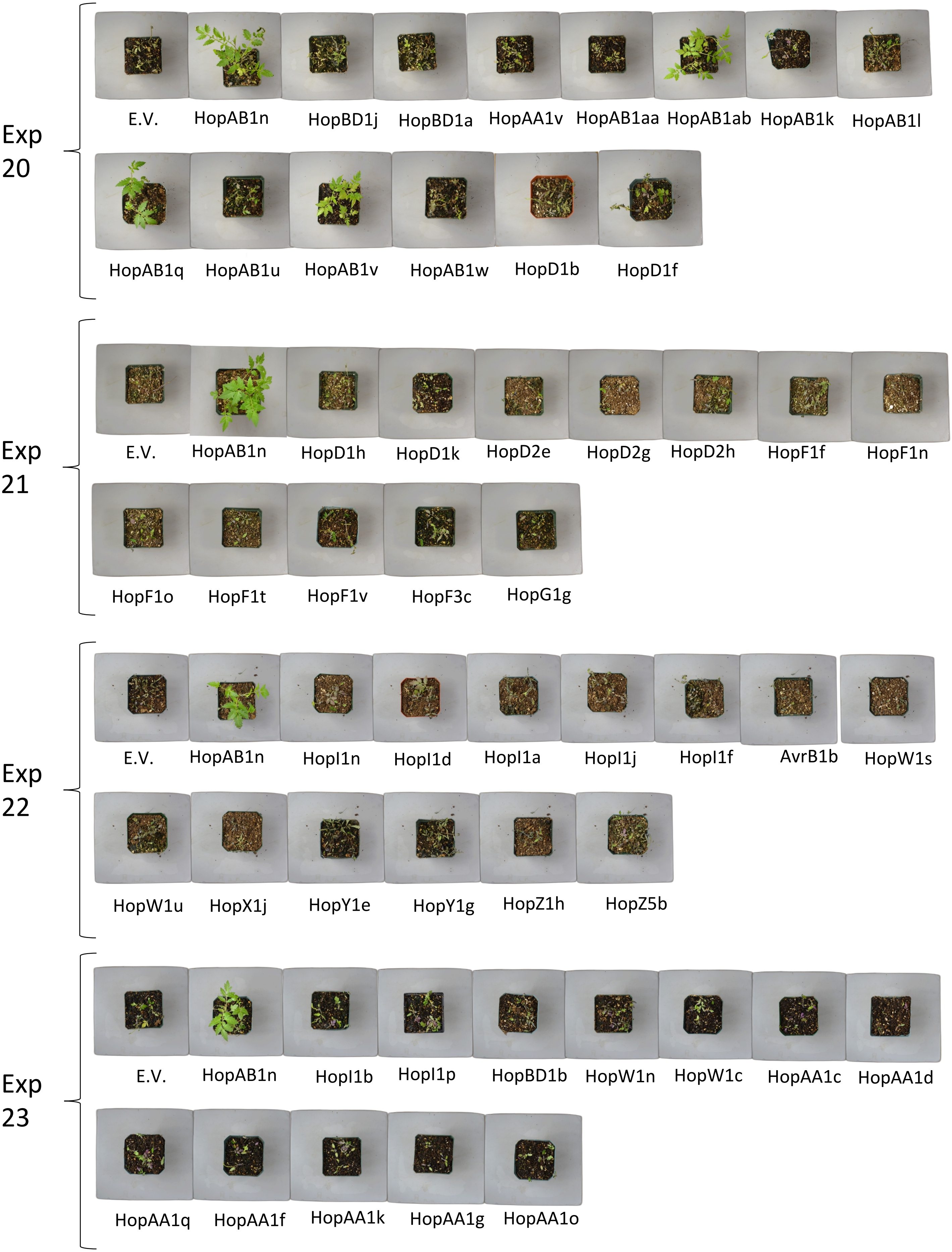
Exp 20
E.V.
HopAB1n
HopBD1j
HopBD1a
HopAA1v
HopAB1aa
HopAB1ab
HopAB1k
HopAB1l
HopAB1q
HopAB1u
HopAB1v
HopAB1w
HopD1b
HopD1f
Exp 21
E.V.
HopAB1n
HopD1h
HopD1k
HopD2e
HopD2g
HopD2h
HopF1f
HopF1n
HopF1o
HopF1t
HopF1v
HopF3c
HopG1g
Exp 22
E.V.
HopAB1n
HopI1n
HopI1d
HopI1a
HopI1j
HopI1f
AvrB1b
HopW1s
HopW1u
HopX1j
HopY1e
HopY1g
HopZ1h
HopZ5b
Exp 23
E.V.
HopAB1n
HopI1b
HopI1p
HopBD1b
HopW1n
HopW1c
HopAA1c
HopAA1d
HopAA1q
HopAA1f
HopAA1k
HopAA1g
HopAA1o

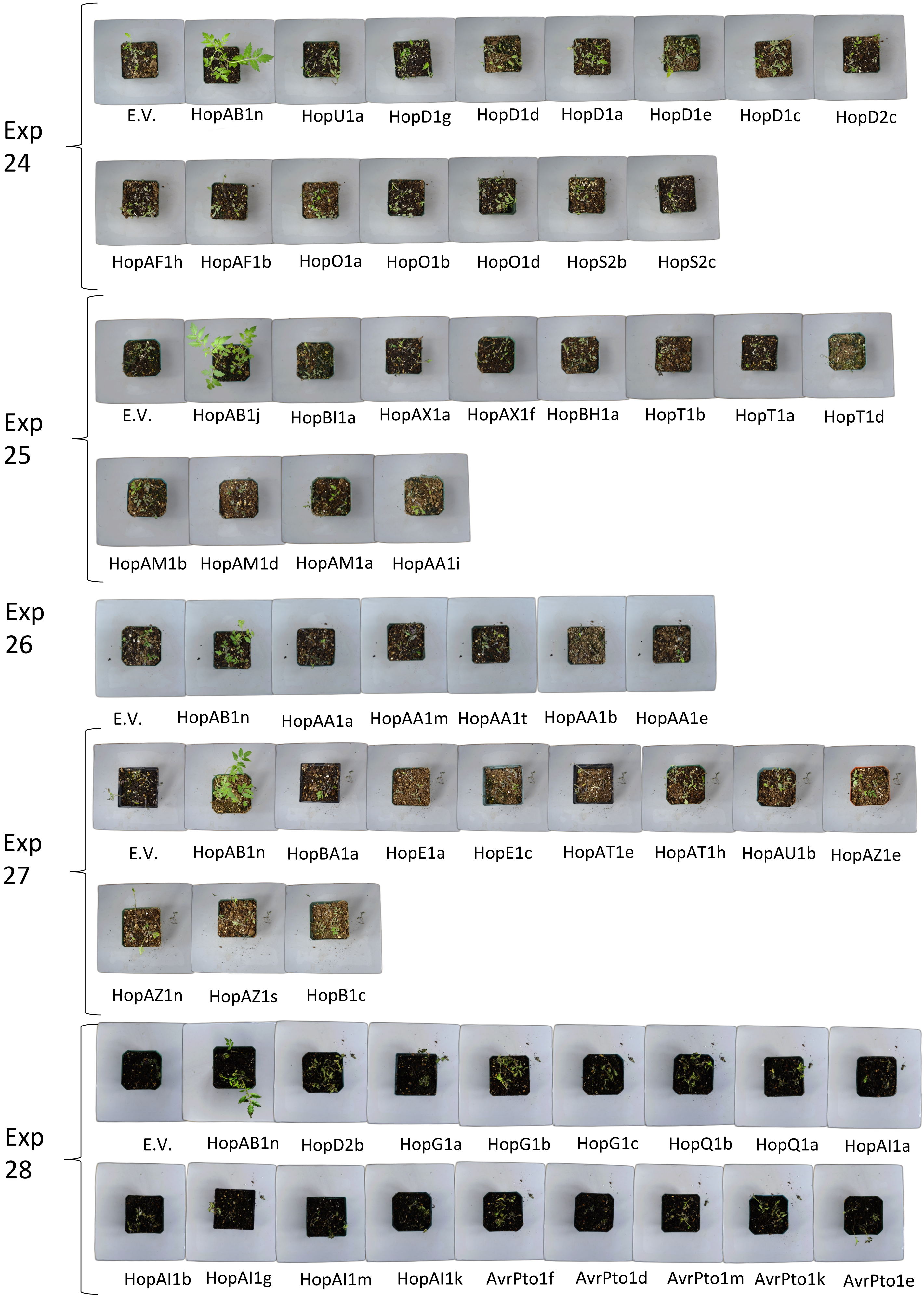
Exp 24
E.V.
HopAB1n
HopU1a
HopD1g
HopD1d
HopD1a
HopD1e
HopD1c
HopD2c
HopAF1h
HopAF1b
HopO1a
HopO1b
HopO1d
HopS2b
HopS2c
Exp 25
E.V.
HopAB1j
HopBI1a
HopAX1a
HopAX1f
HopBH1a
HopT1b
HopT1a
HopT1d
HopAM1b
HopAM1d
HopAM1a
HopAA1i
Exp 26
E.V.
HopAB1n
HopAA1a
HopAA1m
HopAA1t
HopAA1b
HopAA1e
Exp 27
E.V.
HopAB1n
HopBA1a
HopE1a
HopE1c
HopAT1e
HopAT1h
HopAU1b
HopAZ1e
HopAZ1n
HopAZ1s
HopB1c
Exp 28
E.V.
HopAB1n
HopD2b
HopG1a
HopG1b
HopG1c
HopQ1b
HopQ1a
HopAI1a
HopAI1b
HopAI1g
HopAI1m
HopAI1k
AvrPto1f
AvrPto1d
AvrPto1m
AvrPto1k
AvrPto1e

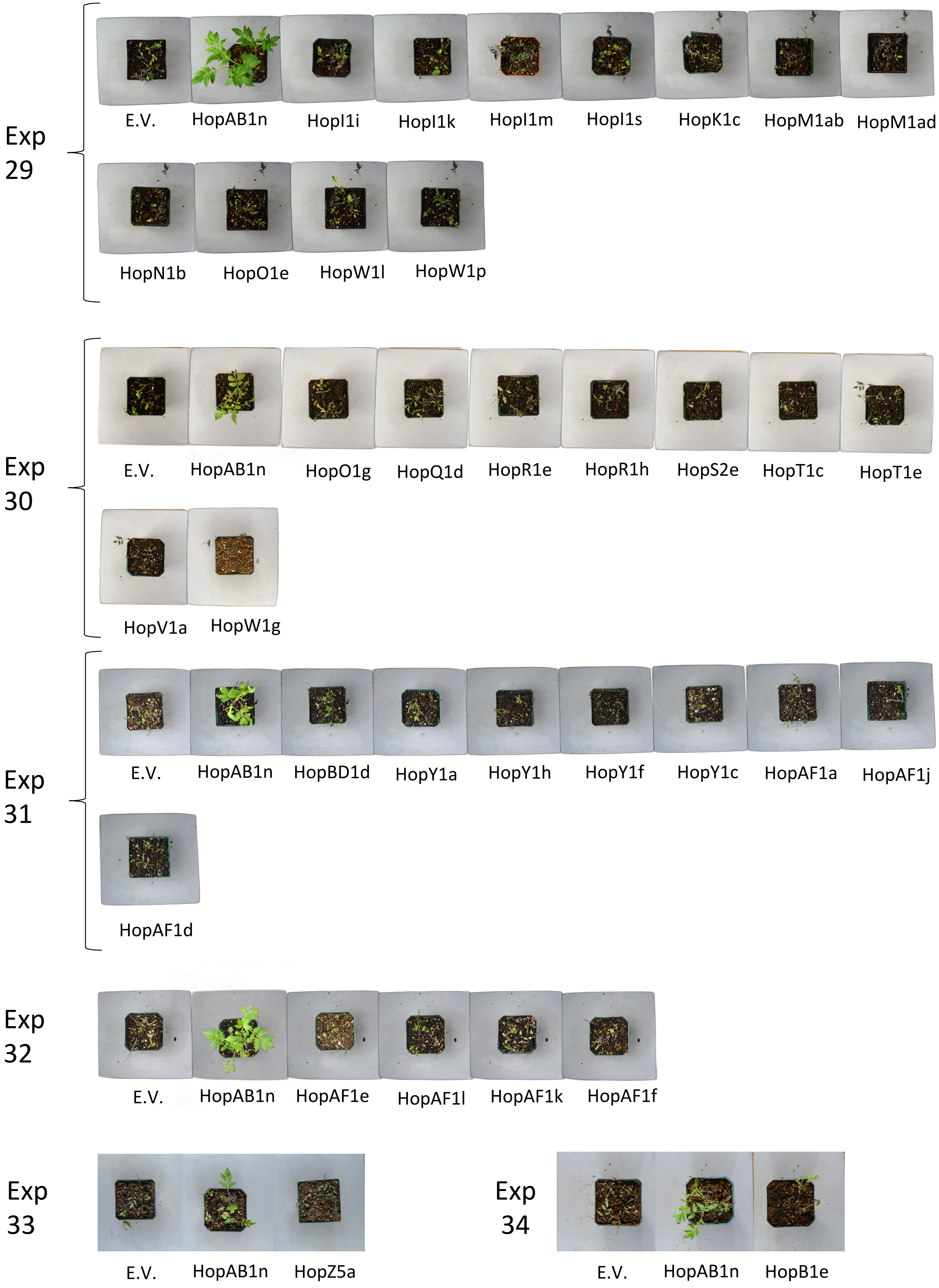
Exp 29
E.V.
HopAB1n
HopI1i
HopI1k
HopI1m
HopI1s
HopK1c
HopM1ab
HopM1ad
HopN1b
HopO1e
HopW1l
HopW1p
Exp 30
E.V.
HopAB1n
HopO1g
HopQ1d
HopR1e
HopR1h
HopS2e
HopT1c
HopT1e
HopV1a
HopW1g
Exp 31
E.V.
HopAB1n
HopBD1d
HopY1a
HopY1h
HopY1f
HopY1c
HopAF1a
HopAF1j
HopAF1d
Exp 32
E.V.
HopAB1n
HopAF1e
HopAF1l
HopAF1k
HopAF1f
Exp 33
E.V.
HopAB1n
HopZ5a
Exp 34
E.V.
HopAB1n
HopB1e

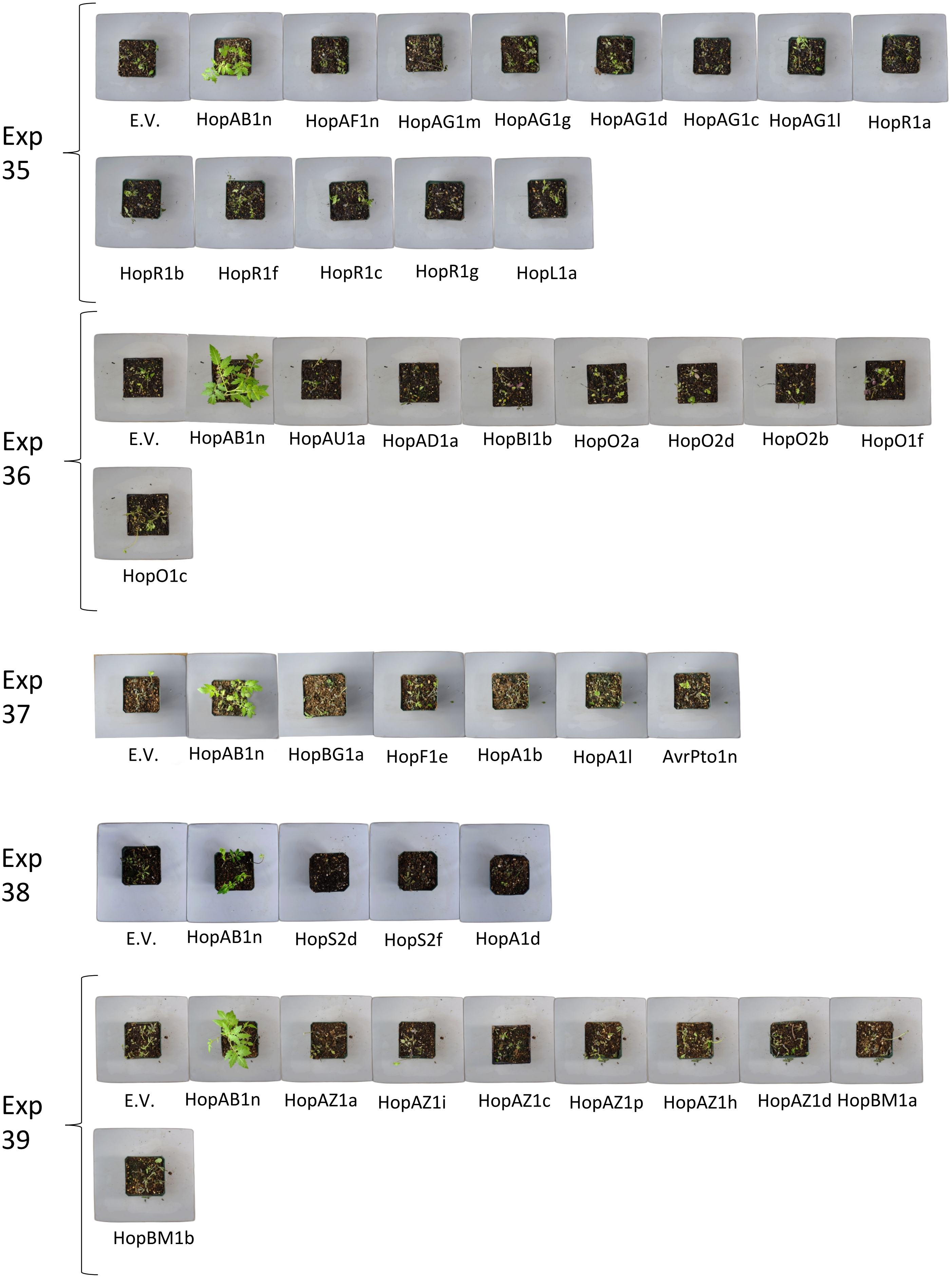
Exp 35
E.V.
HopAB1n
HopAF1n
HopAG1m
HopAG1g
HopAG1d
HopAG1c
HopAG1l
HopR1a
HopR1b
HopR1f
HopR1c
HopR1g
HopL1a
Exp 36
E.V.
HopAB1n
HopAU1a
HopAD1a
HopBI1b
HopO2a
HopO2d
HopO2b
HopO1f
HopO1c
Exp 37
E.V.
HopAB1n
HopBG1a
HopF1e
HopA1b
HopA1l
AvrPto1n
Exp 38
E.V.
HopAB1n
HopS2d
HopS2f
HopA1d
Exp 39
E.V.
HopAB1n
HopAZ1a
HopAZ1i
HopAZ1c
HopAZ1p
HopAZ1h
HopAZ1d
HopBM1a
HopBM1b

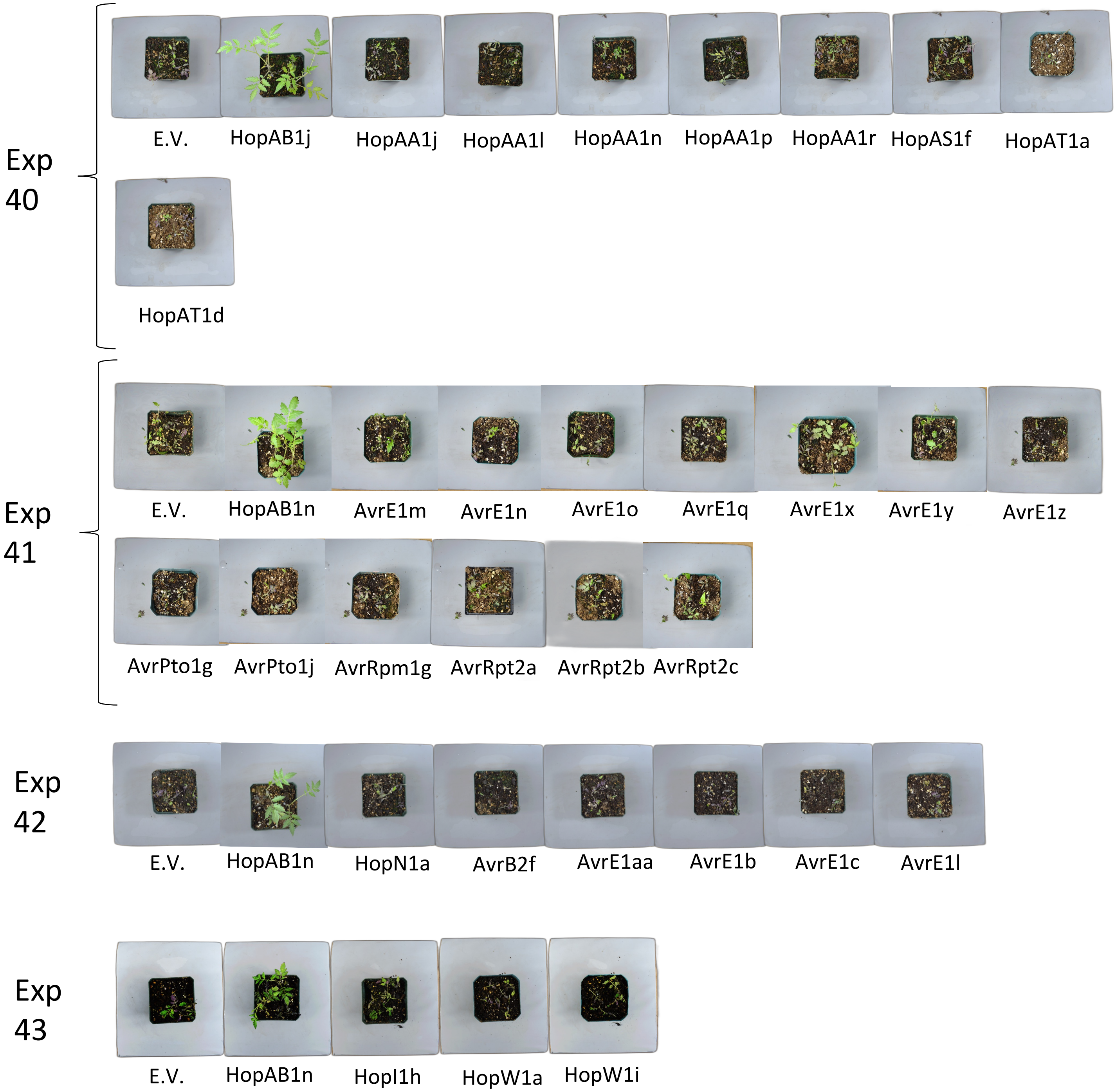

Supplementary Figure 5. Compiled images of primary ETI screening of PsyTEC in *S. arcanum*. Images of *S. arcanum* plants spray inoculated with PtoDC3000 Δ-avrPto Δ hopAB1 expressing representative PsyTEC effector alleles 5-8 days post inoculation that were used to determine the disease score presented in Fig 3. Effector allele names are presented above the images. Each experiment included a positive ETI control, HopAB1, and a negative ETI control, Empty Vector.

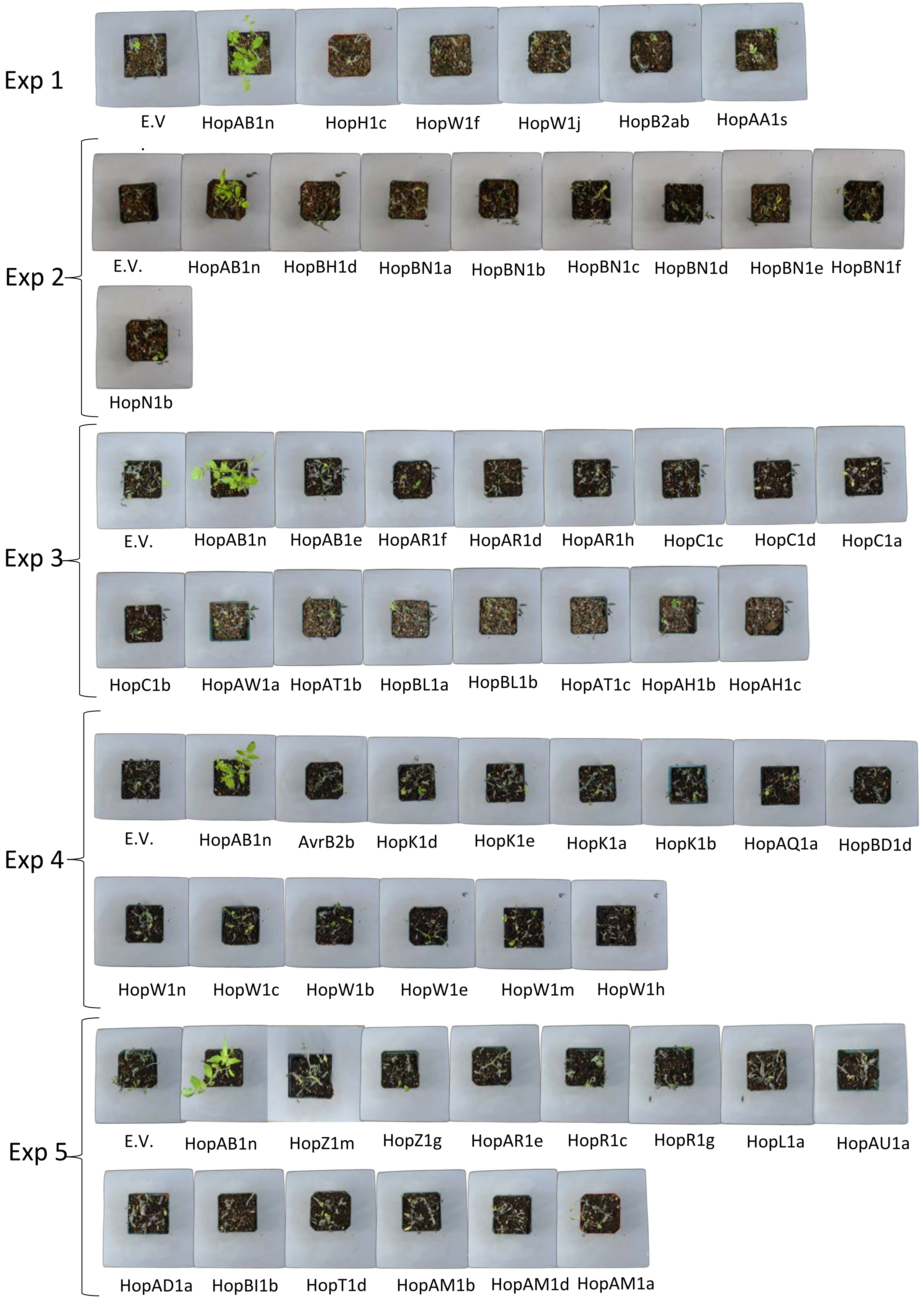
Exp 1
E.V
HopAB1n
HopH1c
HopW1f
HopW1j
HopB2ab
HopAA1s
Exp 2
E.V.
HopAB1n
HopBH1d
HopBN1a
HopBN1b
HopBN1c
HopBN1d
HopBN1e
HopBN1f
HopN1b
Exp 3
E.V.
HopAB1n
HopAB1e
HopAR1f
HopAR1d
HopAR1h
HopC1c
HopC1d
HopC1a
HopC1b
HopAW1a
HopAT1b
HopBL1a
HopBL1b
HopAT1c
HopAH1b
HopAH1c
Exp 4
E.V.
HopAB1n
AvrB2b
HopK1d
HopK1e
HopK1a
HopK1b
HopAQ1a
HopBD1d
HopW1n
HopW1c
HopW1b
HopW1e
HopW1m
HopW1h
Exp 5
E.V.
HopAB1n
HopZ1m
HopZ1g
HopAR1e
HopR1c
HopR1g
HopL1a
HopAU1a
HopAD1a
HopBI1b
HopT1d
HopAM1b
HopAM1d
HopAM1a

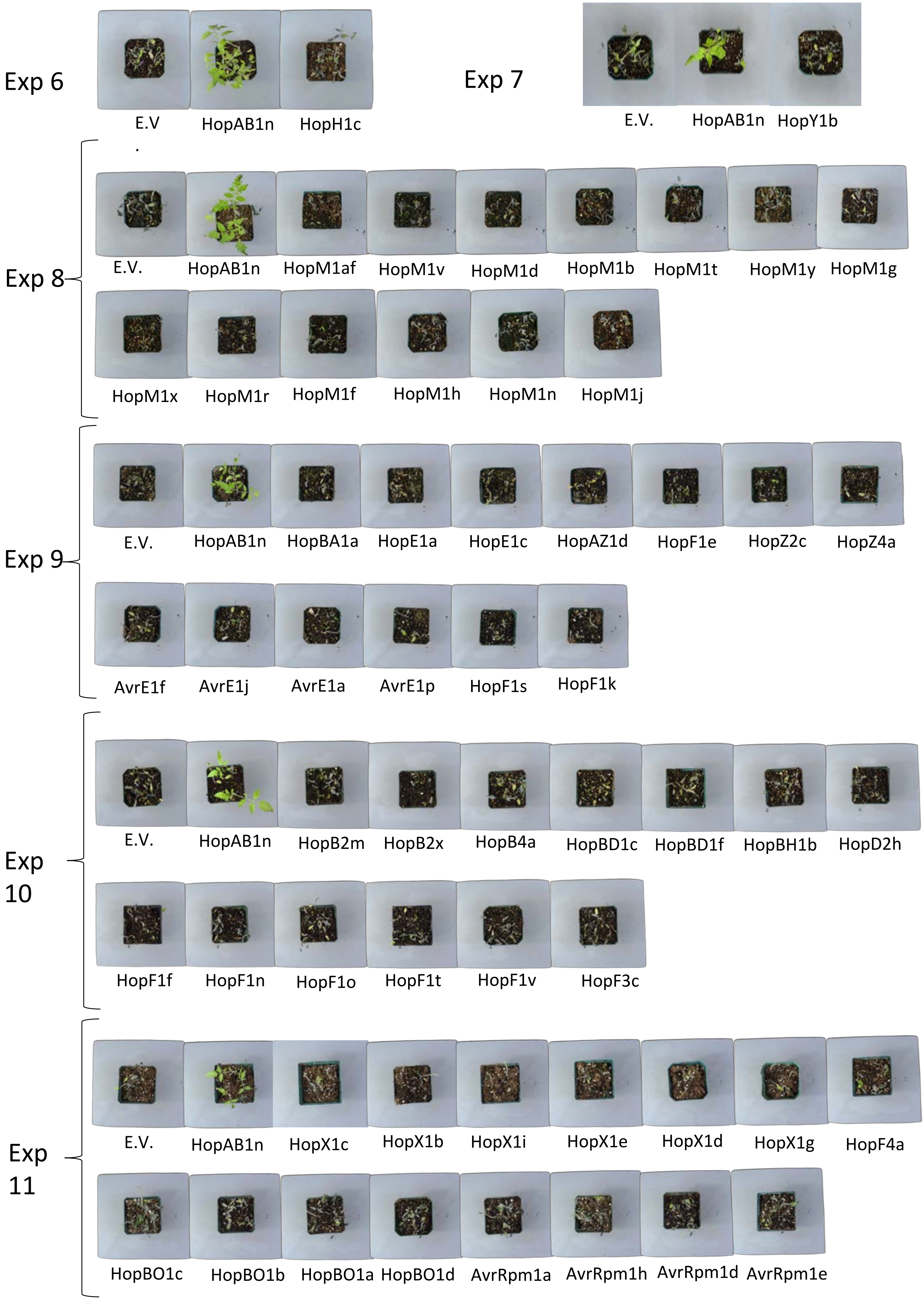
Exp 6
E.V
HopAB1n
HopH1c
Exp 7
E.V.
HopAB1n
HopY1b
Exp 8
E.V.
HopAB1n
HopM1af
HopM1v
HopM1d
HopM1b
HopM1t
HopM1y
HopM1g
HopM1x
HopM1r
HopM1f
HopM1h
HopM1n
HopM1j
Exp 9
E.V.
HopAB1n
HopBA1a
HopE1a
HopE1c
HopAZ1d
HopF1e
HopZ2c
HopZ4a
AvrE1f
AvrE1j
AvrE1a
AvrE1p
HopF1s
HopF1k
Exp 10
E.V.
HopAB1n
HopB2m
HopB2x
HopB4a
HopBD1c
HopBD1f
HopBH1b
HopD2h
HopF1f
HopF1n
HopF1o
HopF1t
HopF1v
HopF3c
Exp 11
E.V.
HopAB1n
HopX1c
HopX1b
HopX1i
HopX1e
HopX1d
HopX1g
HopF4a
HopBO1c
HopBO1b
HopBO1a
HopBO1d
AvrRpm1a
AvrRpm1h
AvrRpm1d
AvrRpm1e

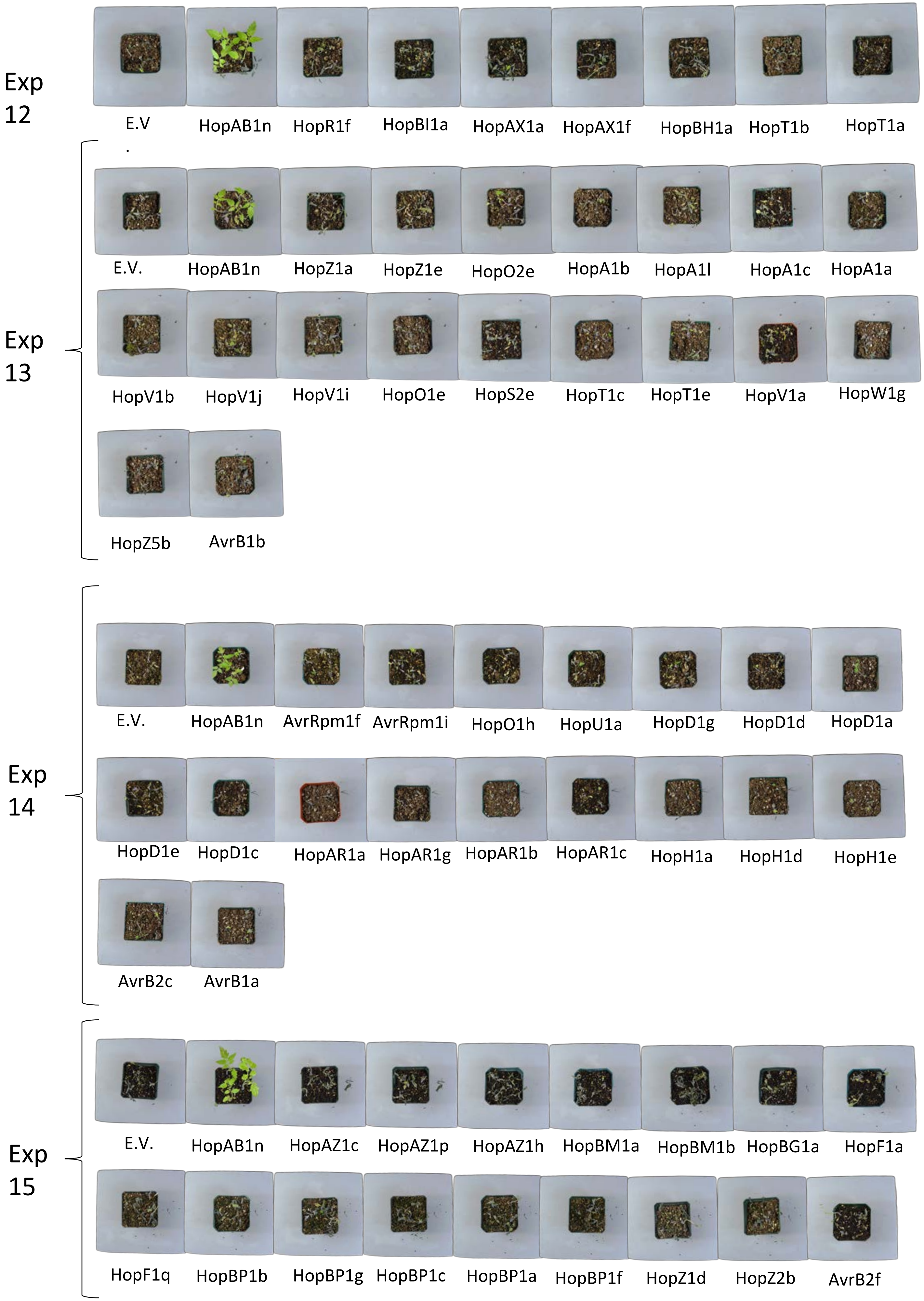

Exp 12
E.V
HopAB1n
HopR1f
HopBI1a
HopAX1a
HopAX1f
HopBH1a
HopT1b
HopT1a
Exp 13
E.V.
HopAB1n
HopZ1a
HopZ1e
HopO2e
HopA1b
HopA1l
HopA1c
HopA1a
HopV1b
HopV1j
HopV1i
HopO1e
HopS2e
HopT1c
HopT1e
HopV1a
HopW1g
HopZ5b
AvrB1b
Exp 14
E.V.
HopAB1n
AvrRpm1f
AvrRpm1i
HopO1h
HopU1a
HopD1g
HopD1d
HopD1a
HopD1e
HopD1c
HopAR1a
HopAR1g
HopAR1b
HopAR1c
HopH1a
HopH1d
HopH1e
AvrB2c
AvrB1a
Exp 15
E.V.
HopAB1n
HopAZ1c
HopAZ1p
HopAZ1h
HopBM1a
HopBM1b
HopBG1a
HopF1a
HopF1q
HopBP1b
HopBP1g
HopBP1c
HopBP1a
HopBP1f
HopZ1d
HopZ2b
AvrB2f

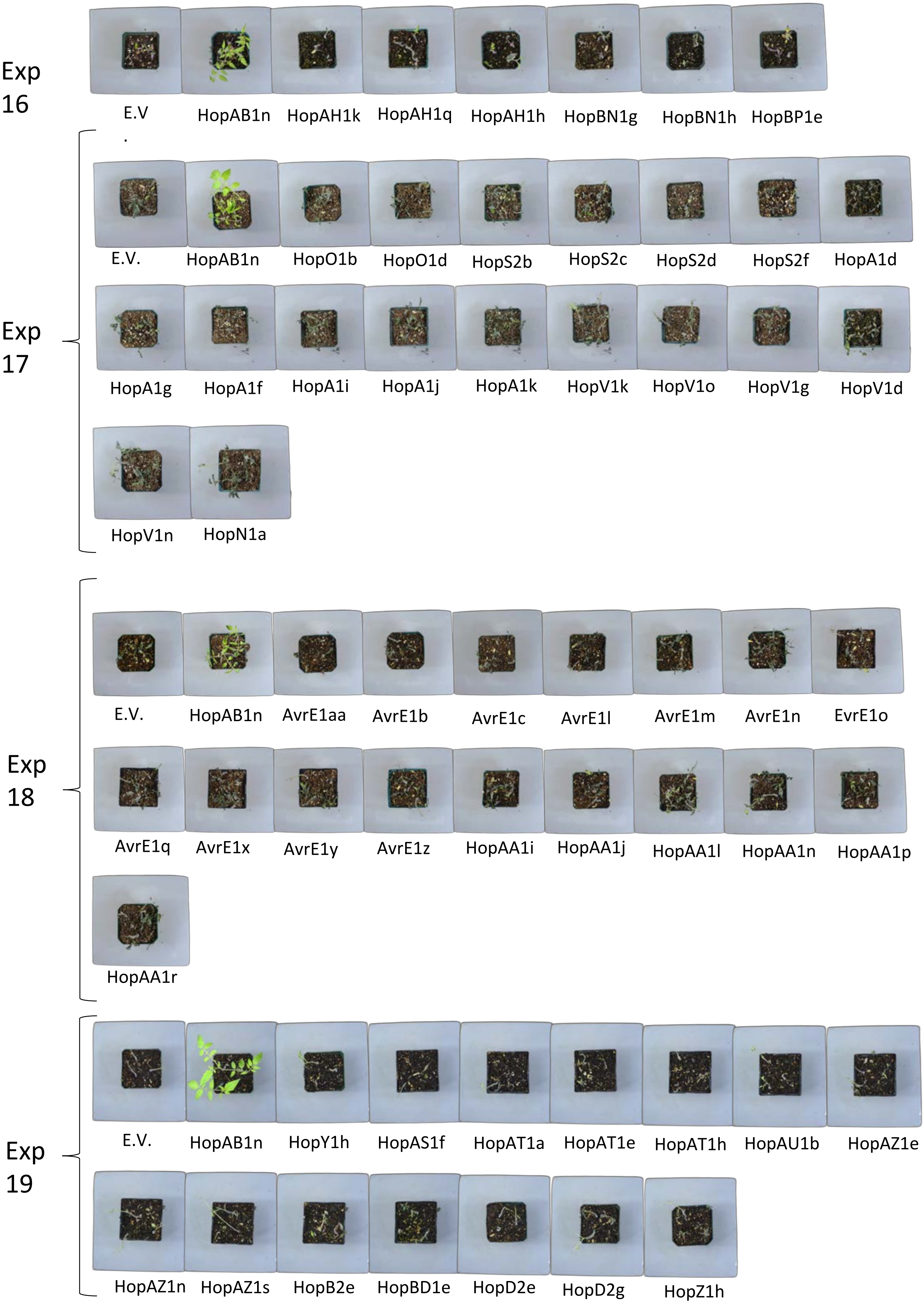
Exp 16
E.V
HopAB1n
HopAH1k
HopAH1q
HopAH1h
HopBN1g
HopBN1h
HopBP1e
Exp 17
E.V.
HopAB1n
HopO1b
HopO1d
HopS2b
HopS2c
HopS2d
HopS2f
HopA1d
HopA1g
HopA1f
HopA1i
HopA1j
HopA1k
HopV1k
HopV1o
HopV1g
HopV1d
HopV1n
HopN1a
Exp 18
E.V.
HopAB1n
AvrE1aa
AvrE1b
AvrE1c
AvrE1l
AvrE1m
AvrE1n
EvrE1o
AvrE1q
AvrE1x
AvrE1y
AvrE1z
HopAA1i
HopAA1j
HopAA1l
HopAA1n
HopAA1p
HopAA1r
Exp 19
E.V.
HopAB1n
HopY1h
HopAS1f
HopAT1a
HopAT1e
HopAT1h
HopAU1b
HopAZ1e
HopAZ1n
HopAZ1s
HopB2e
HopBD1e
HopD2e
HopD2g
HopZ1h

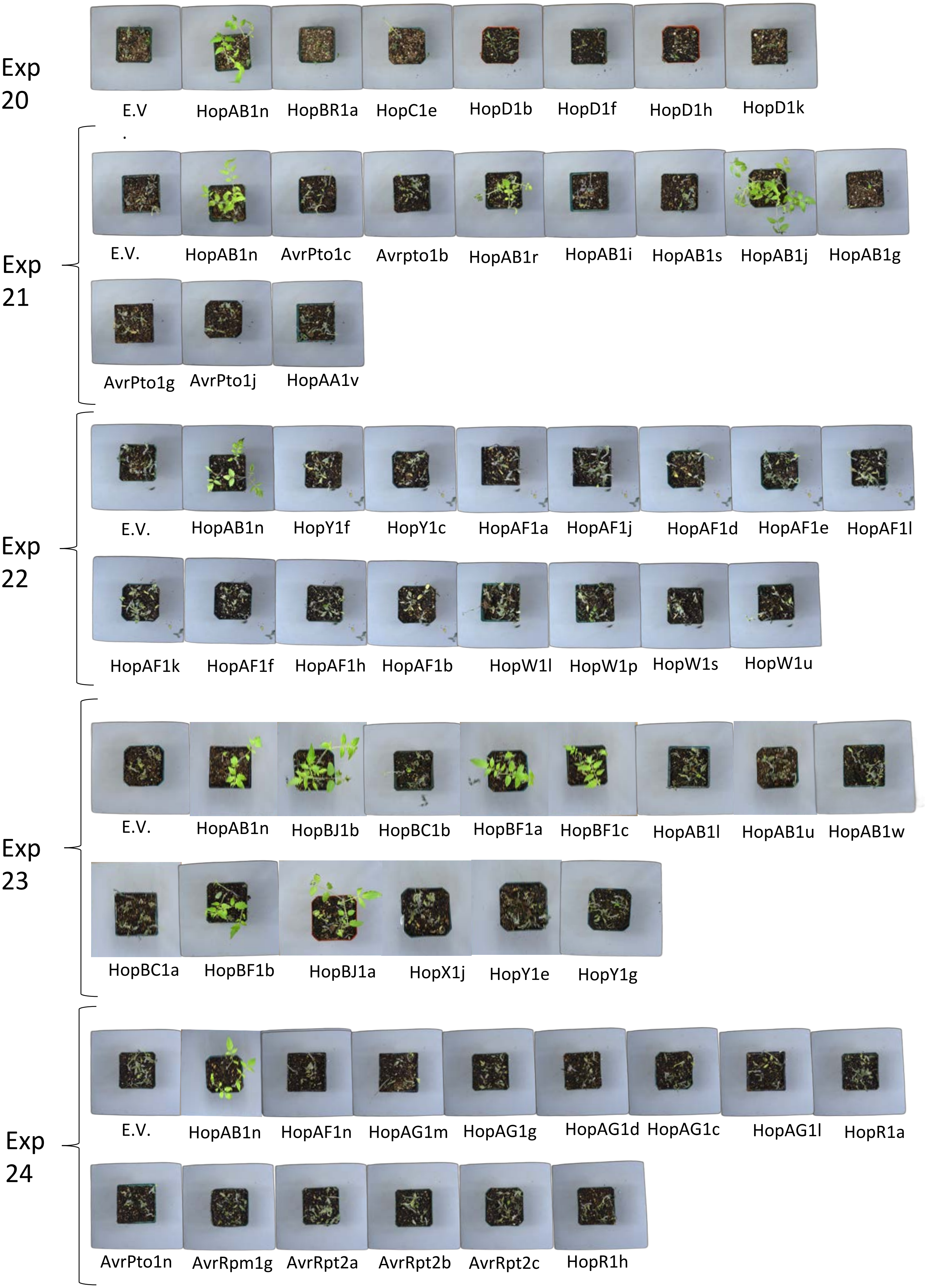
Exp 20
E.V
HopAB1n
HopBR1a
HopC1e
HopD1b
HopD1f
HopD1h
HopD1k
Exp 21
E.V.
HopAB1n
AvrPto1c
Avrpto1b
HopAB1r
HopAB1i
HopAB1s
HopAB1j
HopAB1g
AvrPto1g
AvrPto1j
HopAA1v
Exp 22
E.V.
HopAB1n
HopY1f
HopY1c
HopAF1a
HopAF1j
HopAF1d
HopAF1e
HopAF1l
HopAF1k
HopAF1f
HopAF1h
HopAF1b
HopW1l
HopW1p
HopW1s
HopW1u
Exp 23
E.V.
HopAB1n
HopBJ1b
HopBC1b
HopBF1a
HopBF1c
HopAB1l
HopAB1u
HopAB1w
HopBC1a
HopBF1b
HopBJ1a
HopX1j
HopY1e
HopY1g
Exp 24
E.V.
HopAB1n
HopAF1n
HopAG1m
HopAG1g
HopAG1d
HopAG1c
HopAG1l
HopR1a
AvrPto1n
AvrRpm1g
AvrRpt2a
AvrRpt2b
AvrRpt2c
HopR1h

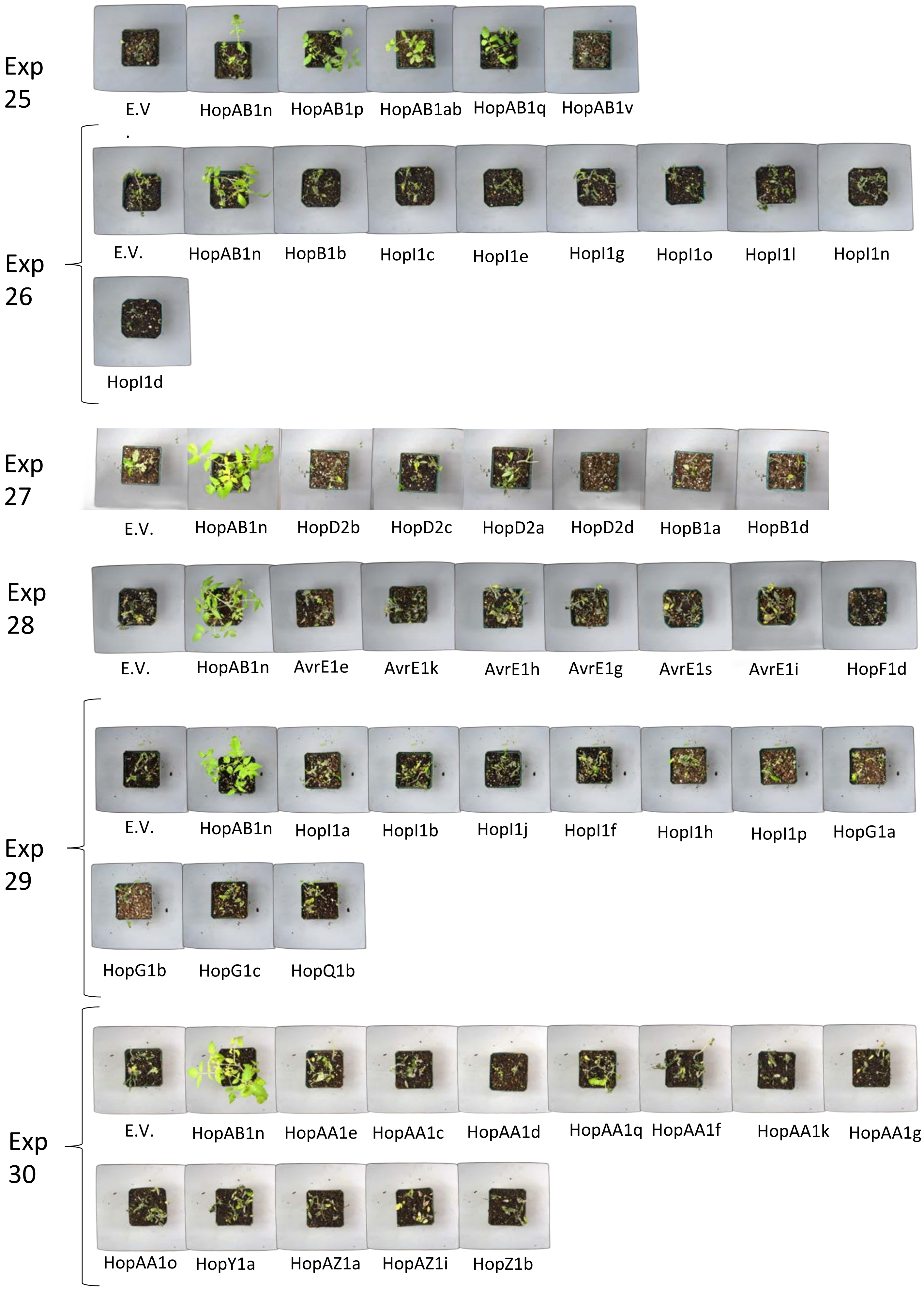

Exp 25
E.V
HopAB1n
HopAB1p
HopAB1ab
HopAB1q
HopAB1v
Exp 26
E.V.
HopAB1n
HopB1b
HopI1c
HopI1e
HopI1g
HopI1o
HopI1l
HopI1n
HopI1d
Exp 27
E.V.
HopAB1n
HopD2b
HopD2c
HopD2a
HopD2d
HopB1a
HopB1d
Exp 28
E.V.
HopAB1n
AvrE1e
AvrE1k
AvrE1h
AvrE1g
AvrE1s
AvrE1i
HopF1d
Exp 29
E.V.
HopAB1n
HopI1a
HopI1b
HopI1j
HopI1f
HopI1h
HopI1p
HopG1a
HopG1b
HopG1c
HopQ1b
Exp 30
E.V.
HopAB1n
HopAA1e
HopAA1c
HopAA1d
HopAA1q
HopAA1f
HopAA1k
HopAA1g
HopAA1o
HopY1a
HopAZ1a
HopAZ1i
HopZ1b

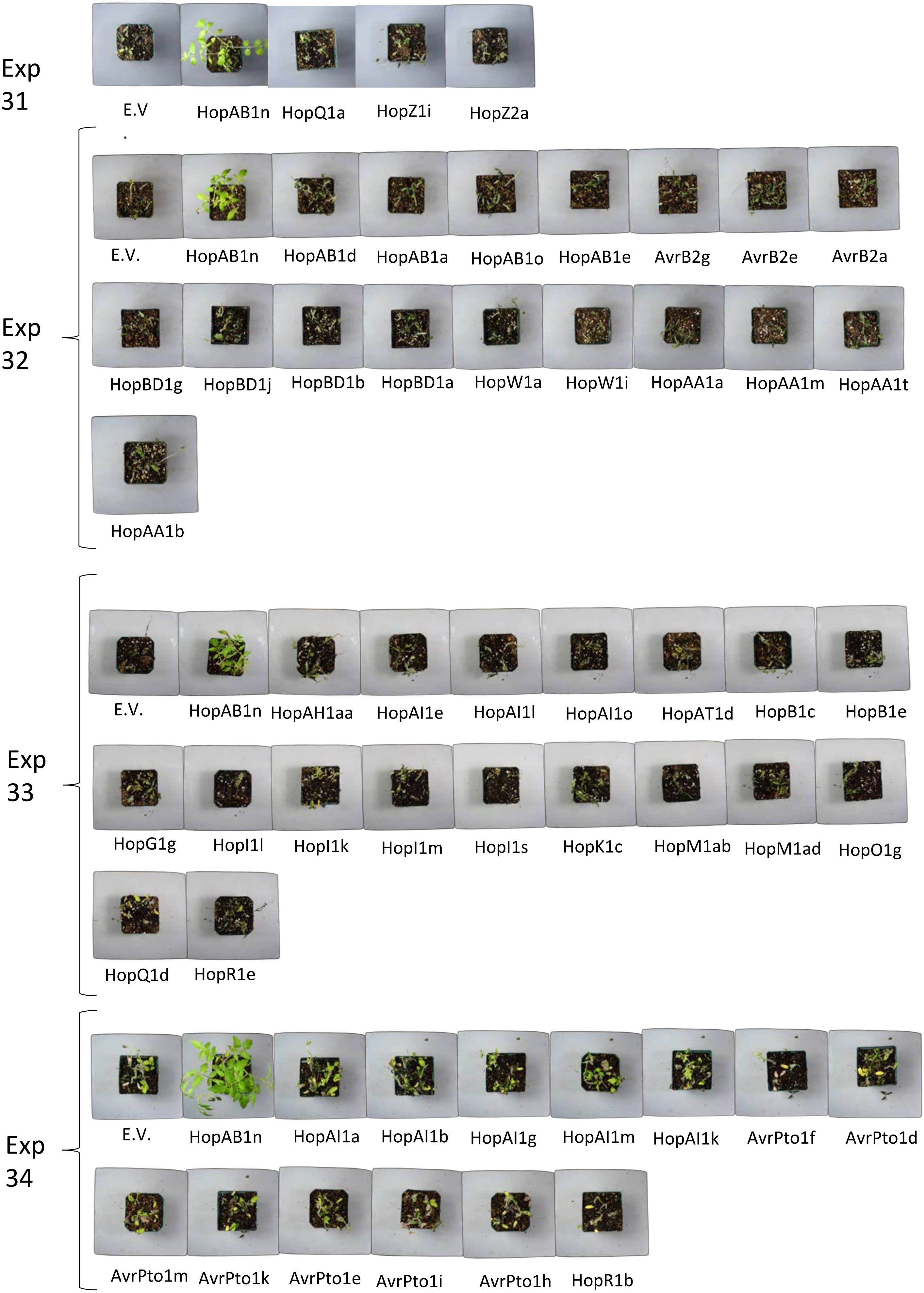

Exp 31
E.V
HopAB1n
HopQ1a
HopZ1i
HopZ2a
Exp 32
E.V.
HopAB1n
HopAB1d
HopAB1a
HopAB1o
HopAB1e
AvrB2g
AvrB2e
AvrB2a
HopBD1g
HopBD1j
HopBD1b
HopBD1a
HopW1a
HopW1i
HopAA1a
HopAA1m
HopAA1t
HopAA1b
Exp 33
E.V.
HopAB1n
HopAH1aa
HopAI1e
HopAI1l
HopAI1o
HopAT1d
HopB1c
HopB1e
HopG1g
HopI1l
HopI1k
HopI1m
HopI1s
HopK1c
HopM1ab
HopM1ad
HopO1g
HopQ1d
HopR1e
Exp 34
E.V.
HopAB1n
HopAI1a
HopAI1b
HopAI1g
HopAI1m
HopAI1k
AvrPto1f
AvrPto1d
AvrPto1m
AvrPto1k
AvrPto1e
AvrPto1i
AvrPto1h
HopR1b

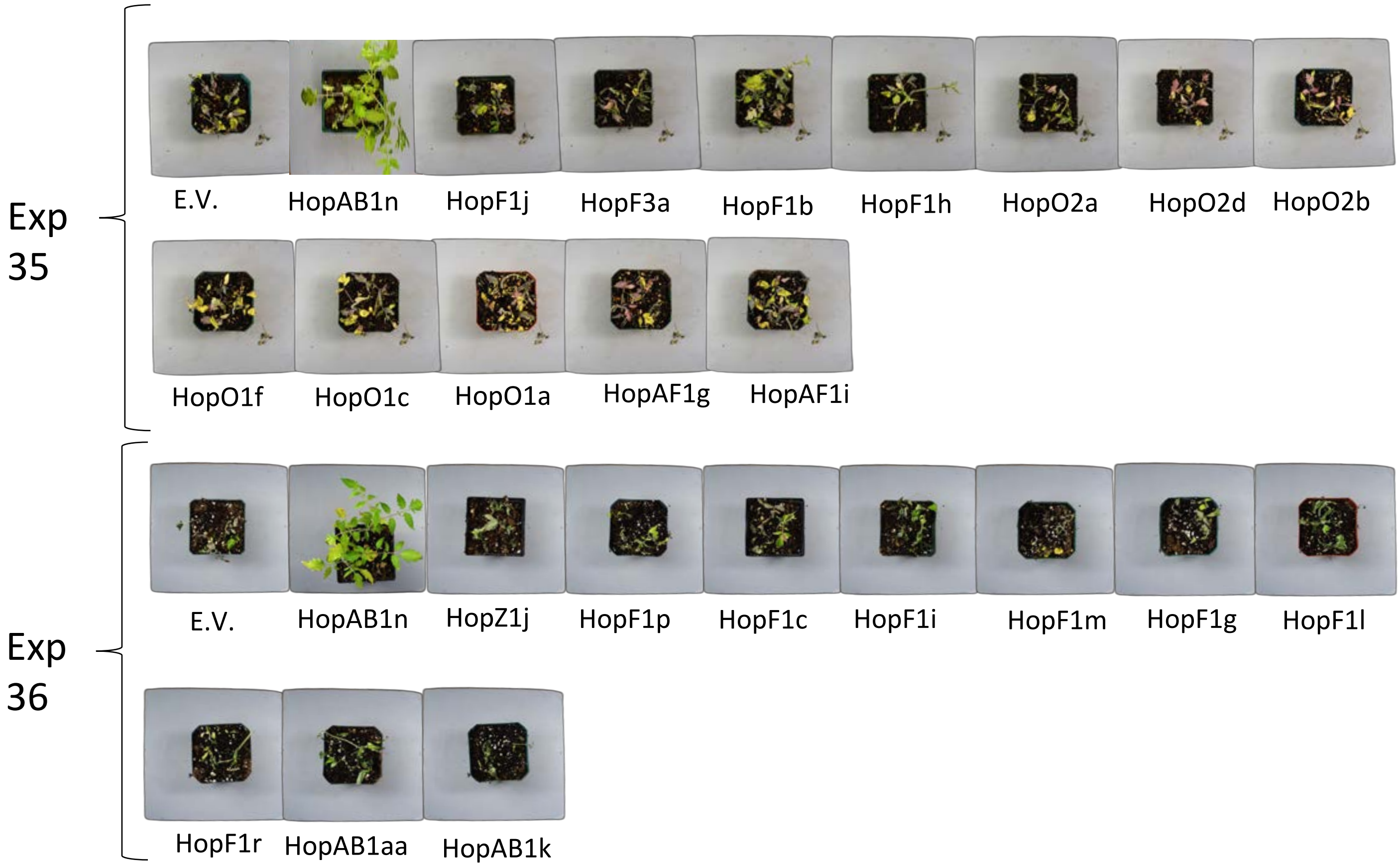

Supplementary Figure 6. Compiled images of primary ETI screening of PsyTEC in *S. pimpinellifolium*. Images of *S. pimpinellifolium* plants spray inoculated with PtoDC3000 $^{\Delta\ avrPto\ \Delta\ hopAB1}$ expressing representative PsyTEC effector alleles 5-8 days post inoculation that were used to determine the disease score presented in Fig 3. Effector allele names are presented above the images. Each experiment included a positive ETI control, HopAB1, and a negative ETI control, Empty Vector.

Supplementary Figure 7

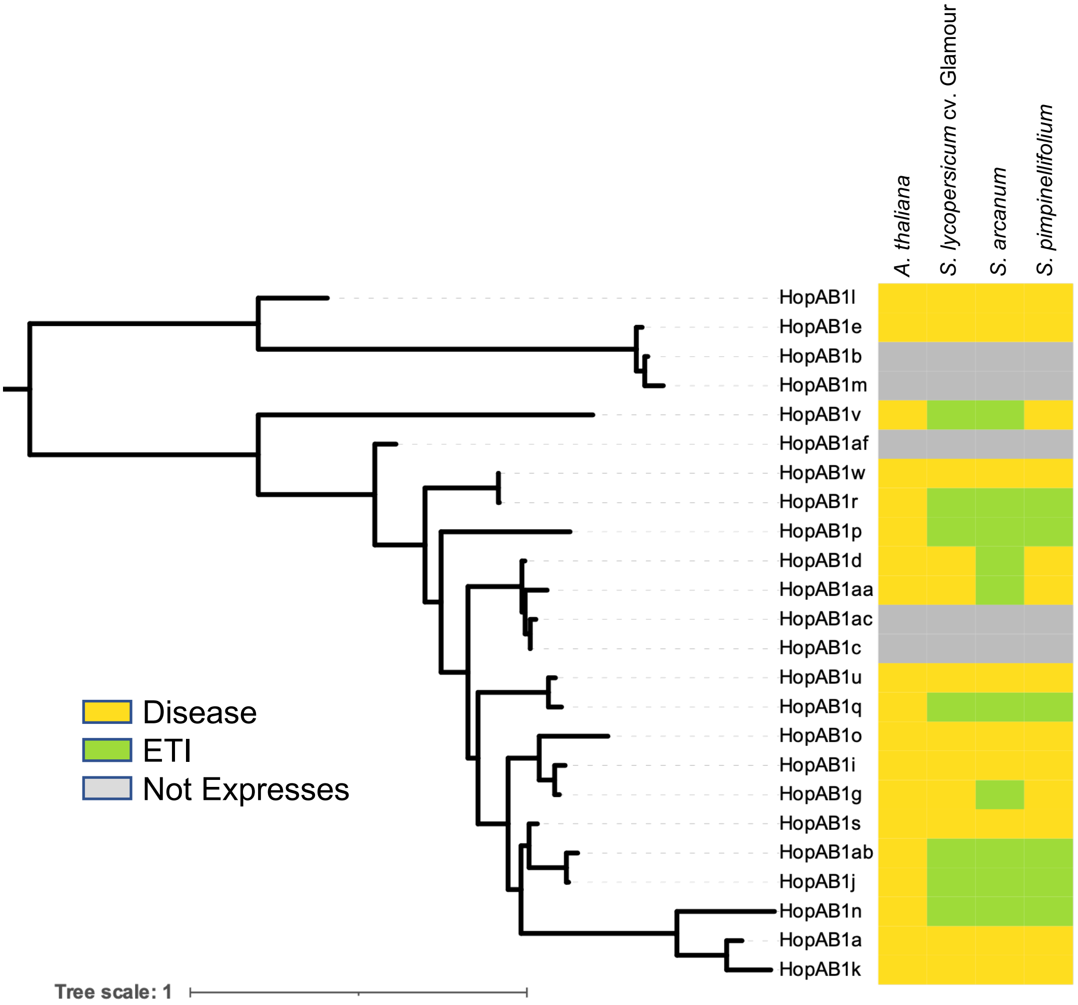

Supplementary Figure 7. Conservation of HopAB ETI responses in *A. thaliana*, tomato var. Glamour and wild tomato species. Maximum likelihood phylogenetic tree of PsyTEC alleles for the HopAB family[23]. A yellow box indicates that the corresponding allele does not trigger an ETI response in the corresponding plant species, while green indicates that the allele triggers an ETI response in the corresponding plant species. A grey box indicates that the allele is not expressed in PtoDC3000 and could not be tested.

a)

Exp 1 :

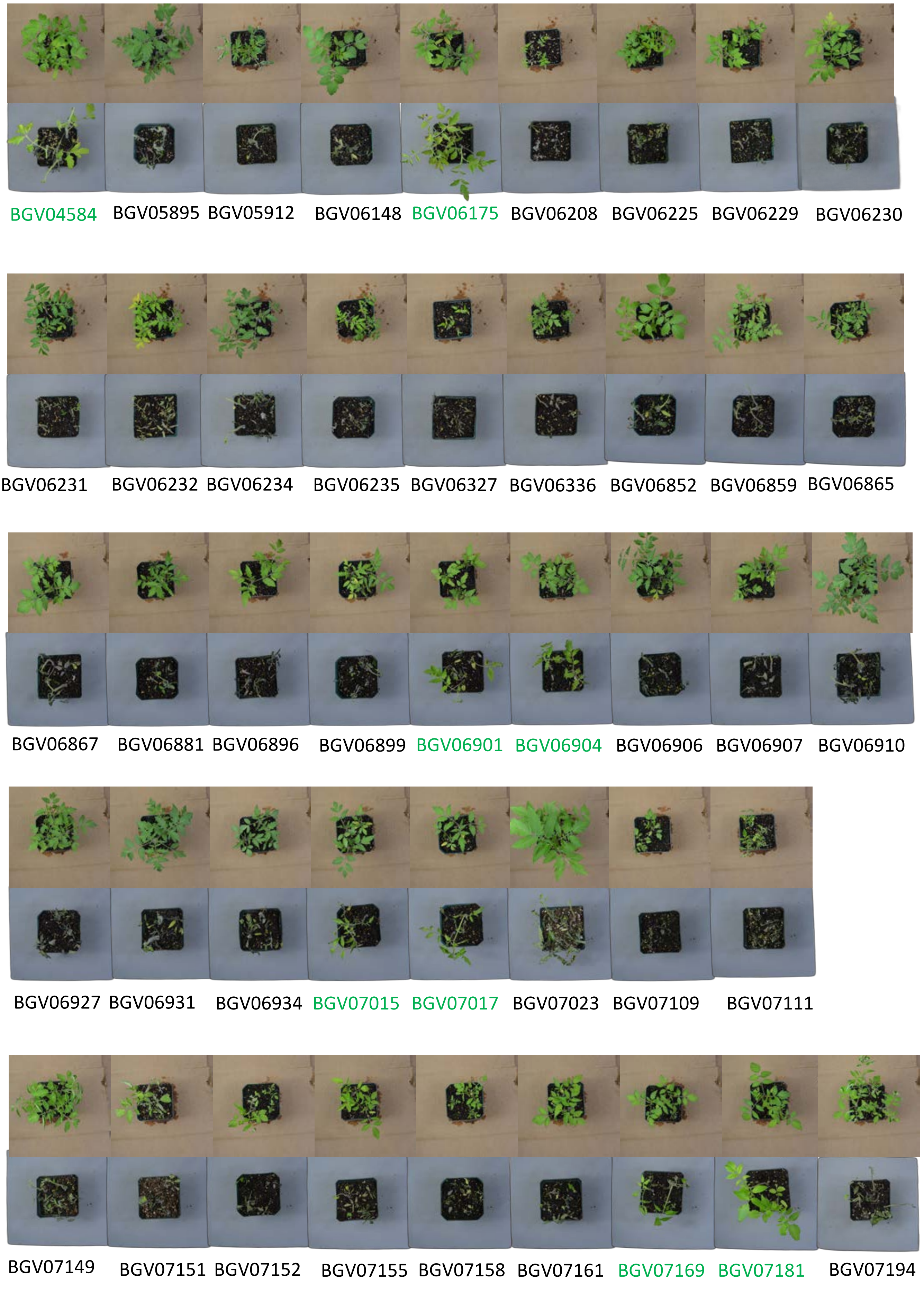
BGV04584 BGV05895 BGV05912 BGV06148 BGV06175 BGV06208 BGV06225 BGV06229 BGV06230
BGV06231 BGV06232 BGV06234 BGV06235 BGV06327 BGV06336 BGV06852 BGV06859 BGV06865
BGV06867 BGV06881 BGV06896 BGV06899 BGV06901 BGV06904 BGV06906 BGV06907 BGV06910
BGV06927 BGV06931 BGV06934 BGV07015 BGV07017 BGV07023 BGV07109 BGV07111
BGV07149 BGV07151 BGV07152 BGV07155 BGV07158 BGV07161 BGV07169 BGV07181 BGV07194

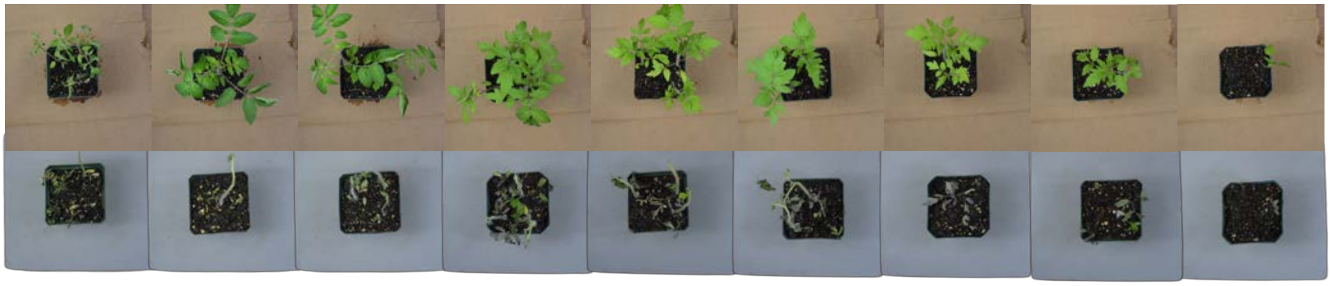

BGV07198 BGV07339 BGV07366 BGV07854 BGV07857 BGV07862 BGV07863 BGV07865 BGV07867

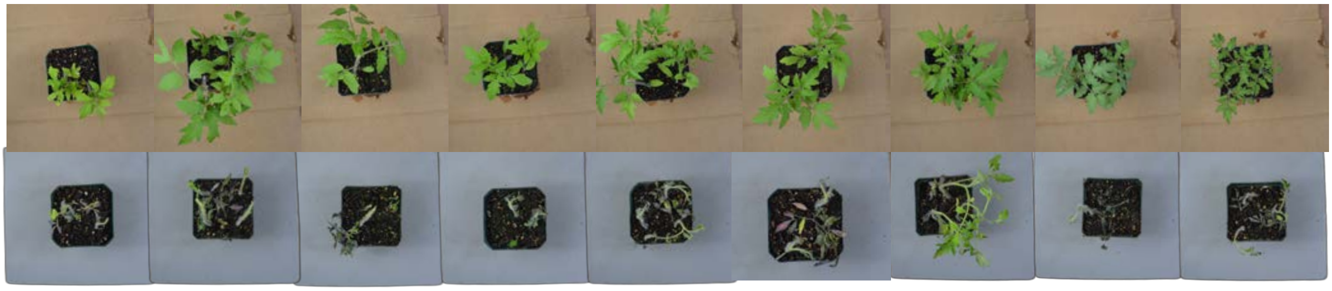

BGV07871 BGV07875 BGV07876 BGV07878 BGV07894 BGV07895 BGV07899 BGV07900 BGV07901

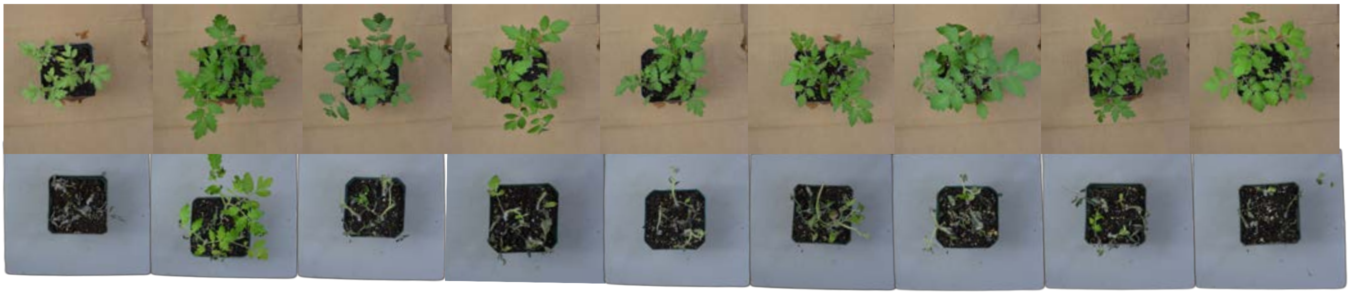

BGV07902 BGV07908 BGV07909 BGV07910 BGV07911 BGV07918 BGV07920 BGV07921 BGV07927

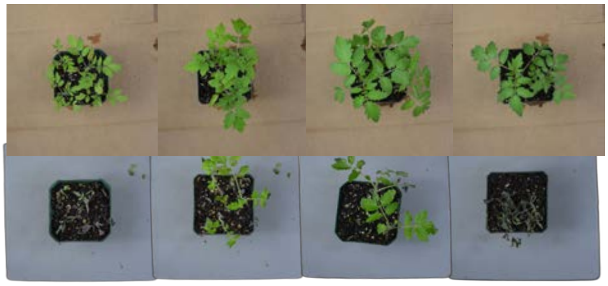

BGV07931 BGV07933 BGV07934 BGV07935

Exp 2 :

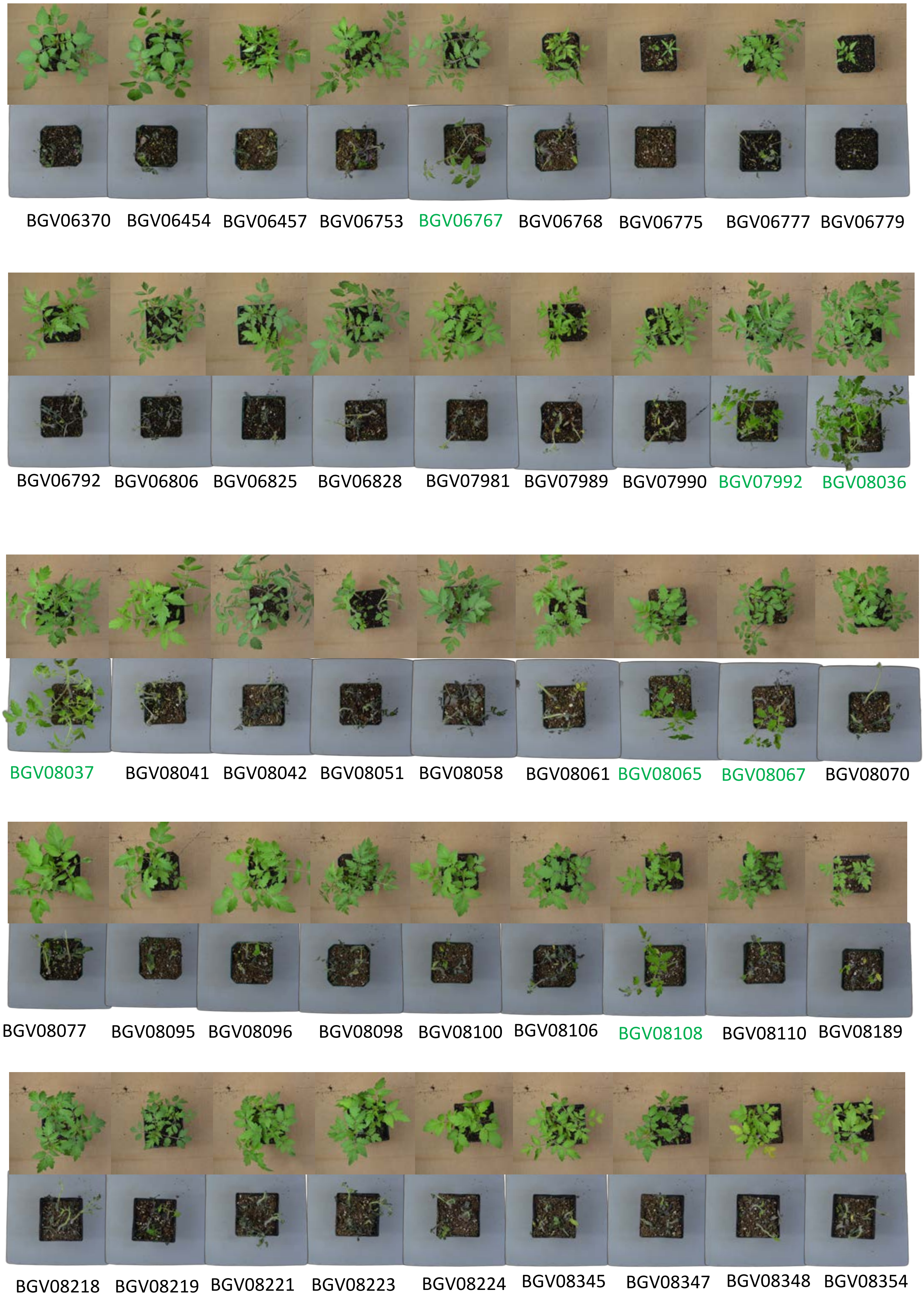

BGV06370
BGV06454
BGV06457
BGV06753
BGV06767
BGV06768
BGV06775
BGV06777
BGV06779
BGV06792
BGV06806
BGV06825
BGV06828
BGV07981
BGV07989
BGV07990
BGV07992
BGV08036
BGV08037
BGV08041
BGV08042
BGV08051
BGV08058
BGV08061
BGV08065
BGV08067
BGV08070
BGV08077
BGV08095
BGV08096
BGV08098
BGV08100
BGV08106
BGV08108
BGV08110
BGV08189
BGV08218
BGV08219
BGV08221
BGV08223
BGV08224
BGV08345
BGV08347
BGV08348
BGV08354

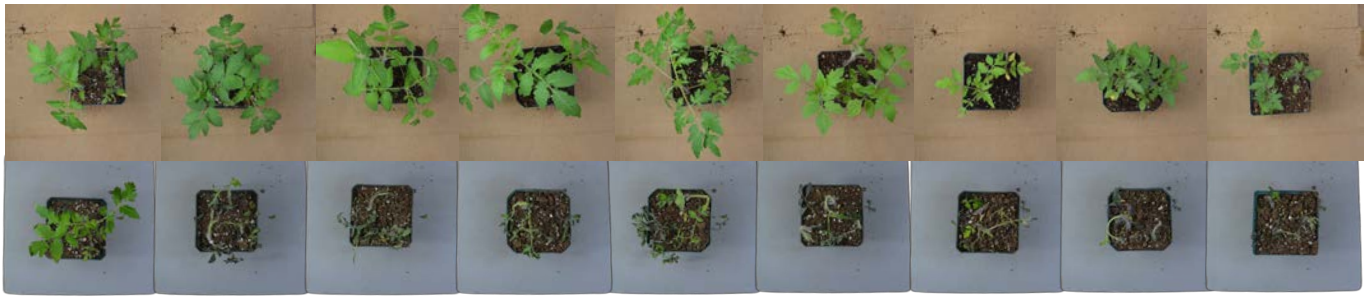

BGV12613 BGV12614 BGV12615 BGV12625 BGV12626 BGV12627 BGV12639 BGV12640 BGV13134

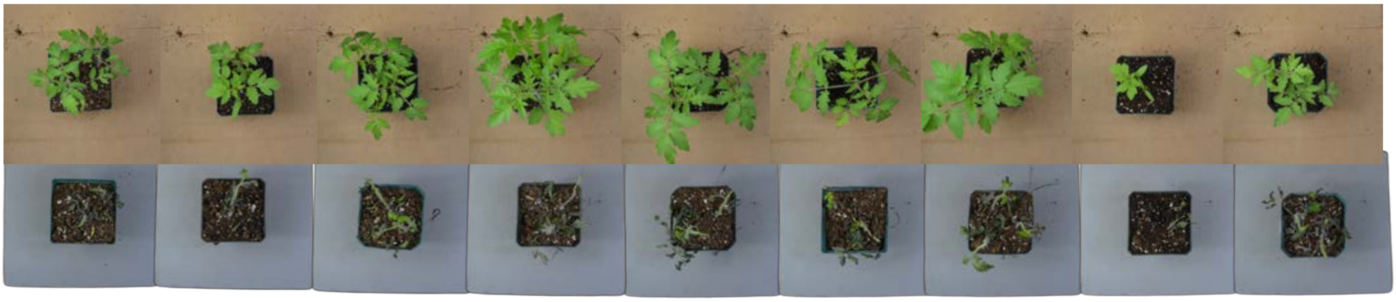

BGV13161 BGV13175 BGV13945 BGV14508 BGV14515 BGV14516 BGV14518 BGV14519 BGV14522

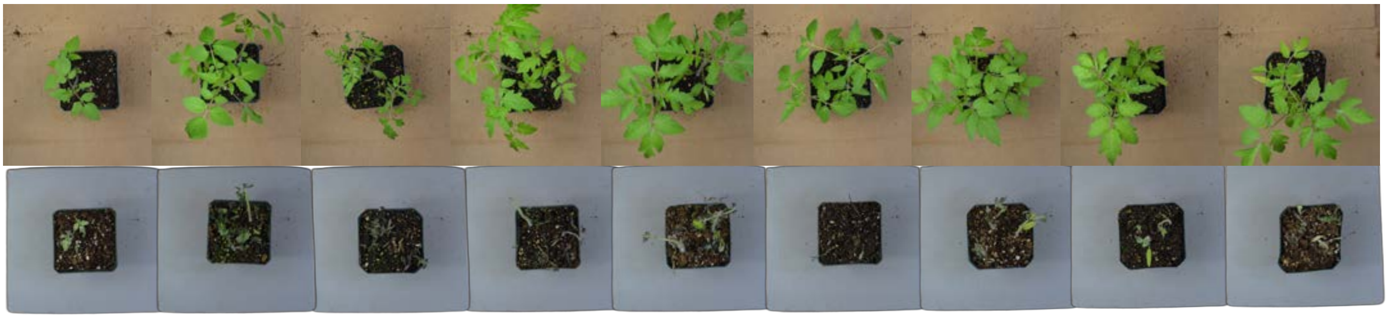

BGV15380 BGV15382 BGV15726 BGV15730 BGV15734 BGV16047 LA1712 LA2309 PI29026

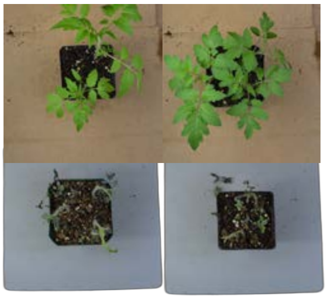

PI406890 PI487625

b)

Exp 1 :

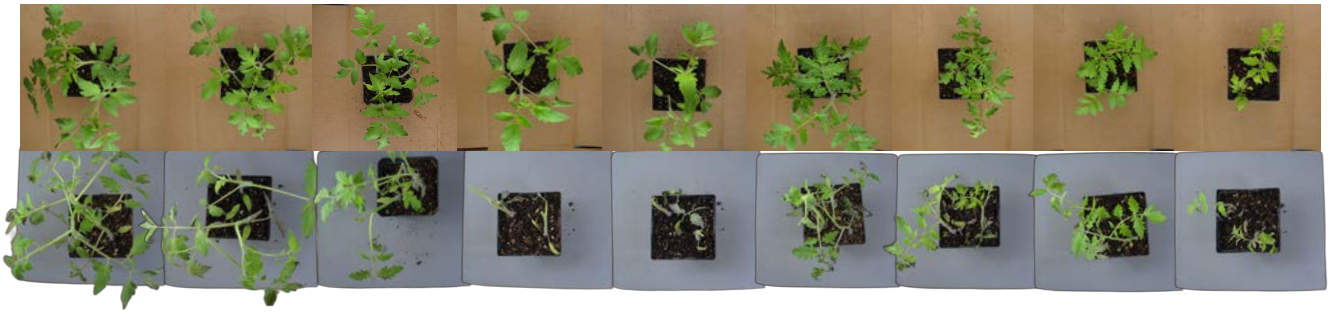
Glamour
Glamour
Glamour
RG-PtoS
RG-PtoS
BGV05895
BGV05912
BGV06225
BGV06229

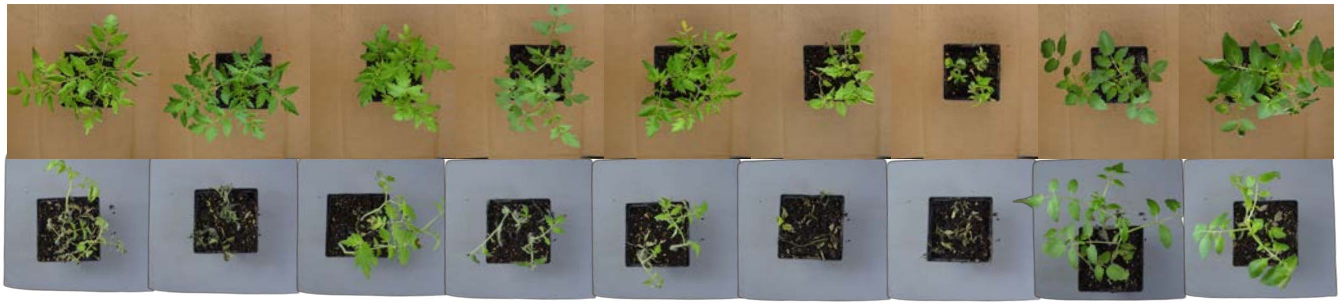
BGV06230
BGV06231
BGV06232
BGV06234
BGV06235
BGV06327
BGV06336
BGV06370
BGV06454

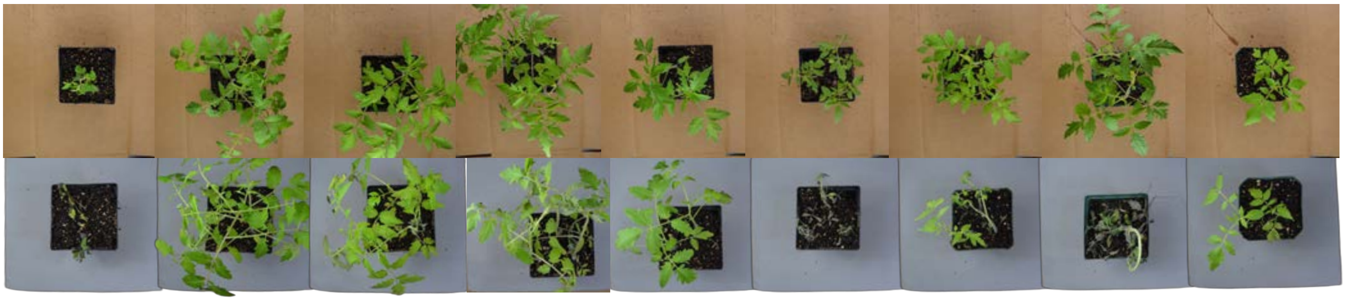
BGV06457
BGV06753
BGV06768
BGV0677
BGV06779
BGV06806
BGV06825
BGV06828
BGV06852

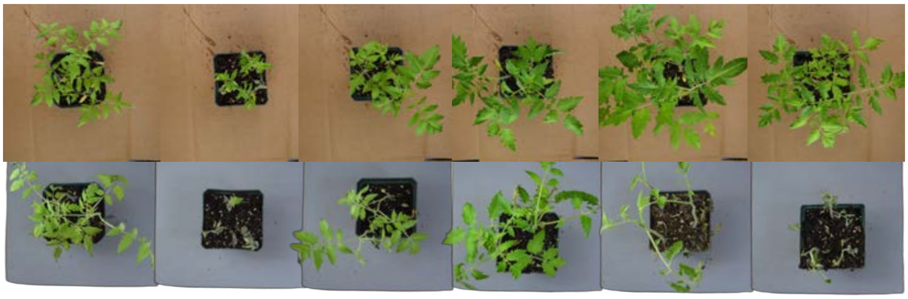
BGV06859
BGV06865
BGV06867
BGV06881
BGV06896
BGV06899

Exp 2 :

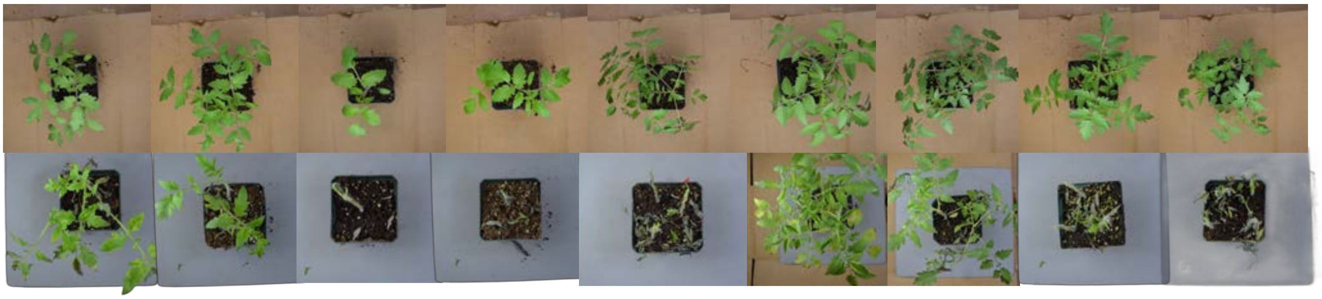

Glamour Glamour RG-PtoS RG-PtoS BGV06906 BGV06907 BGV06910 BGV06927 BGV06931

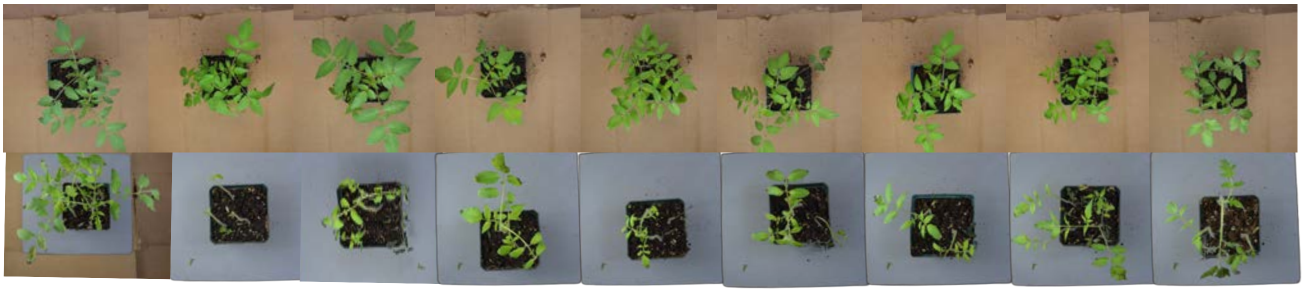

BGV06934 BGV07023 BGV07109 BGV07111 BGV07149 BGV07151 BGV07152 BGV07155 BGV07158

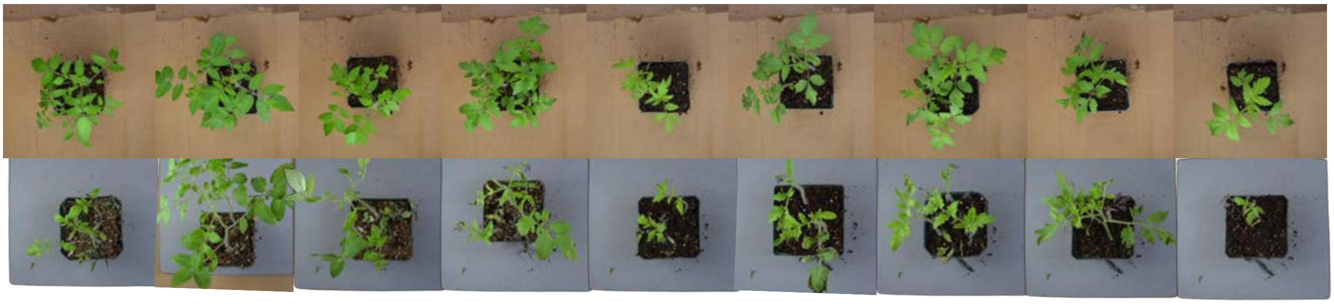

BGV07194 BGV07339 BGV07366 BGV07854 BGV07857 BGV07862 BGV07863 BGV07865 BGV07875

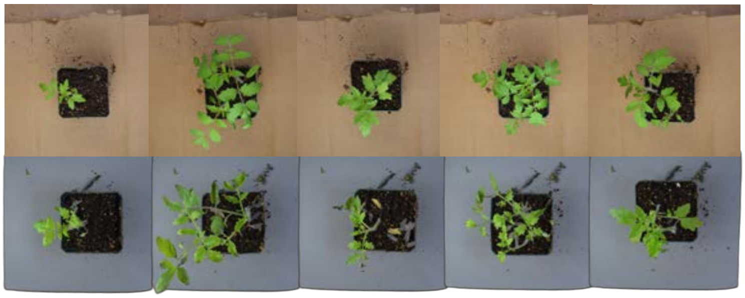

BGV07871 BGV07876 BGV07878 BGV07894 BGV07895

Exp 3 :

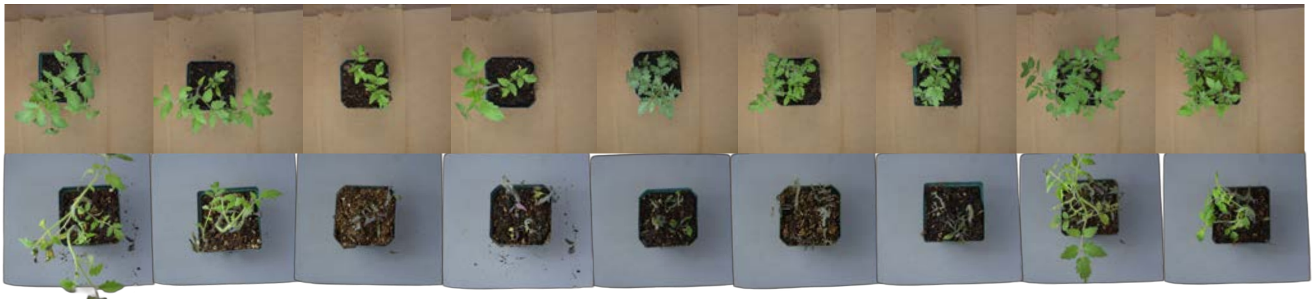

Glamour Glamour RG-PtoS RG-PtoS BGV07900 BGV07901 BGV07902 BGV07909 BGV07910

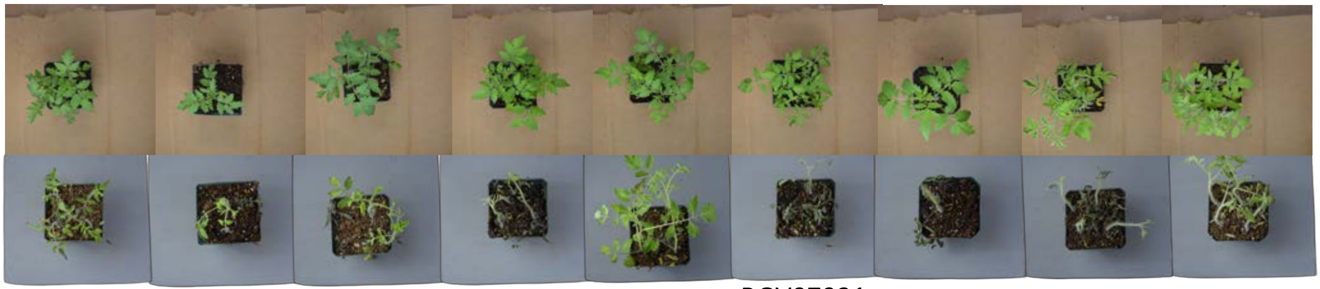

BGV07911 BGV07918 BGV07920 BGV07921 BGV07927 BGV07931 BGV07981 BGV07989 BGV07990

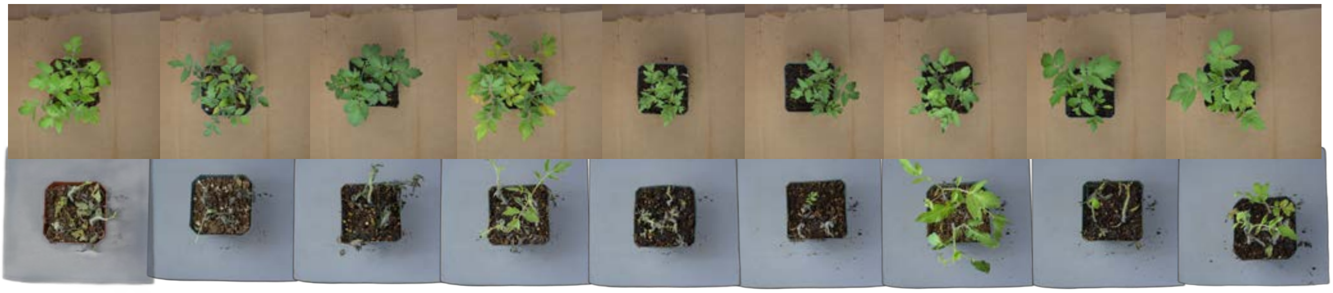

BGV08041 BGV08042 BGV08051 BGV08058 BGV08061 BGV08070 BGV08077 BGV08095 BGV08098

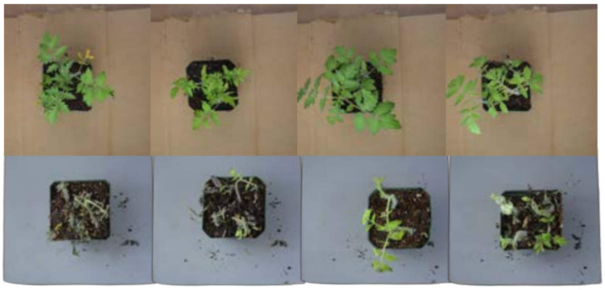

BGV08100 BGV08106 BGV08110 BGV08189

Exp 4 :

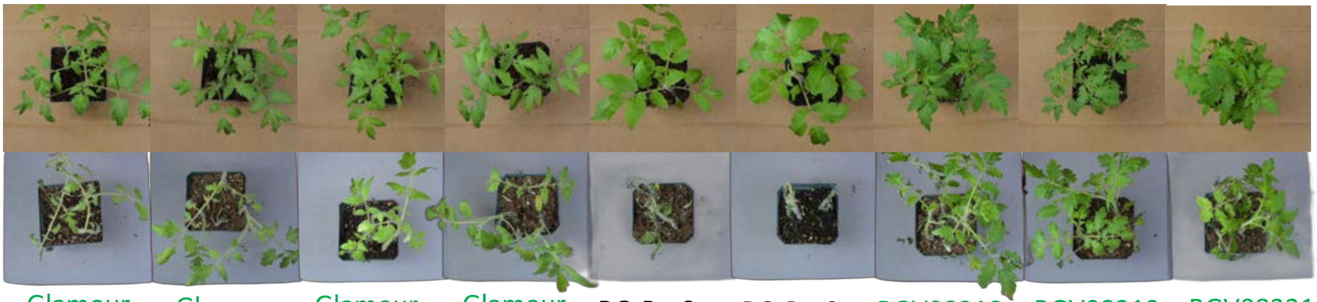

Glamour Glamour Glamour Glamour RG-PtoS RG-PtoS BGV08218 BGV08219 BGV08221

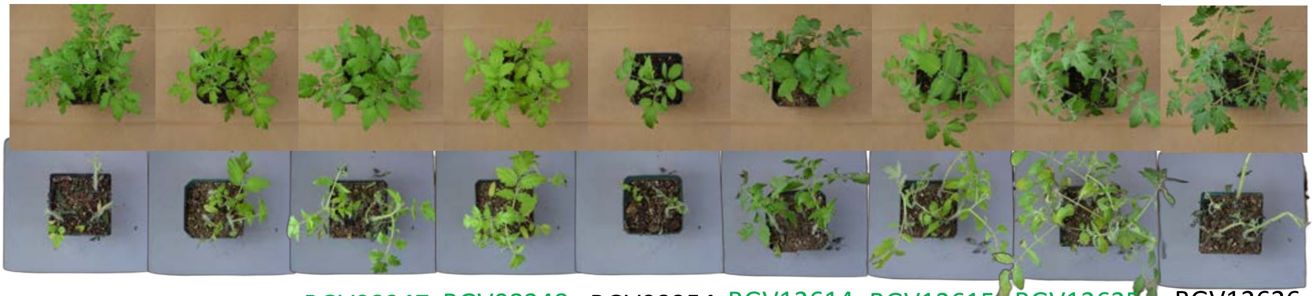

BGV08223 BGV08345 BGV08347 BGV08348 BGV08354 BGV12614 BGV12615 BGV12625 BGV12626

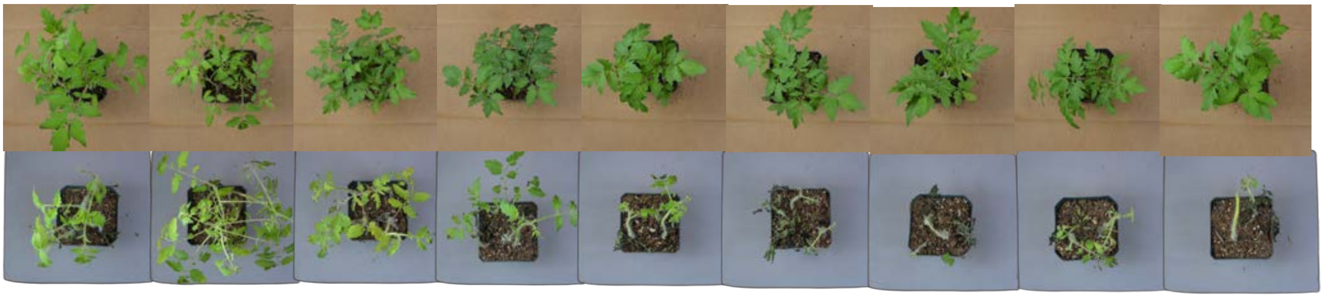

BGV12627 BGV12639 BGV12640 BGV13134 BGV13175 BGV13945 BGV14508 BGV14515 BGV14516

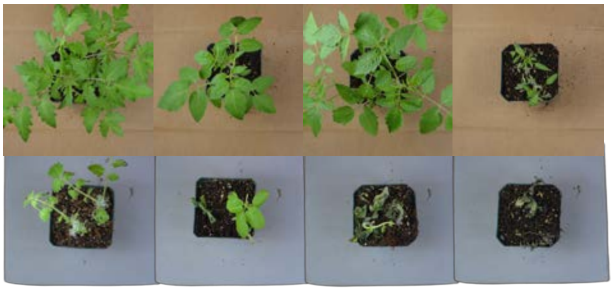

BGV14522 BGV15380 BGV15382 BGV15726

c)

Exp 1 :

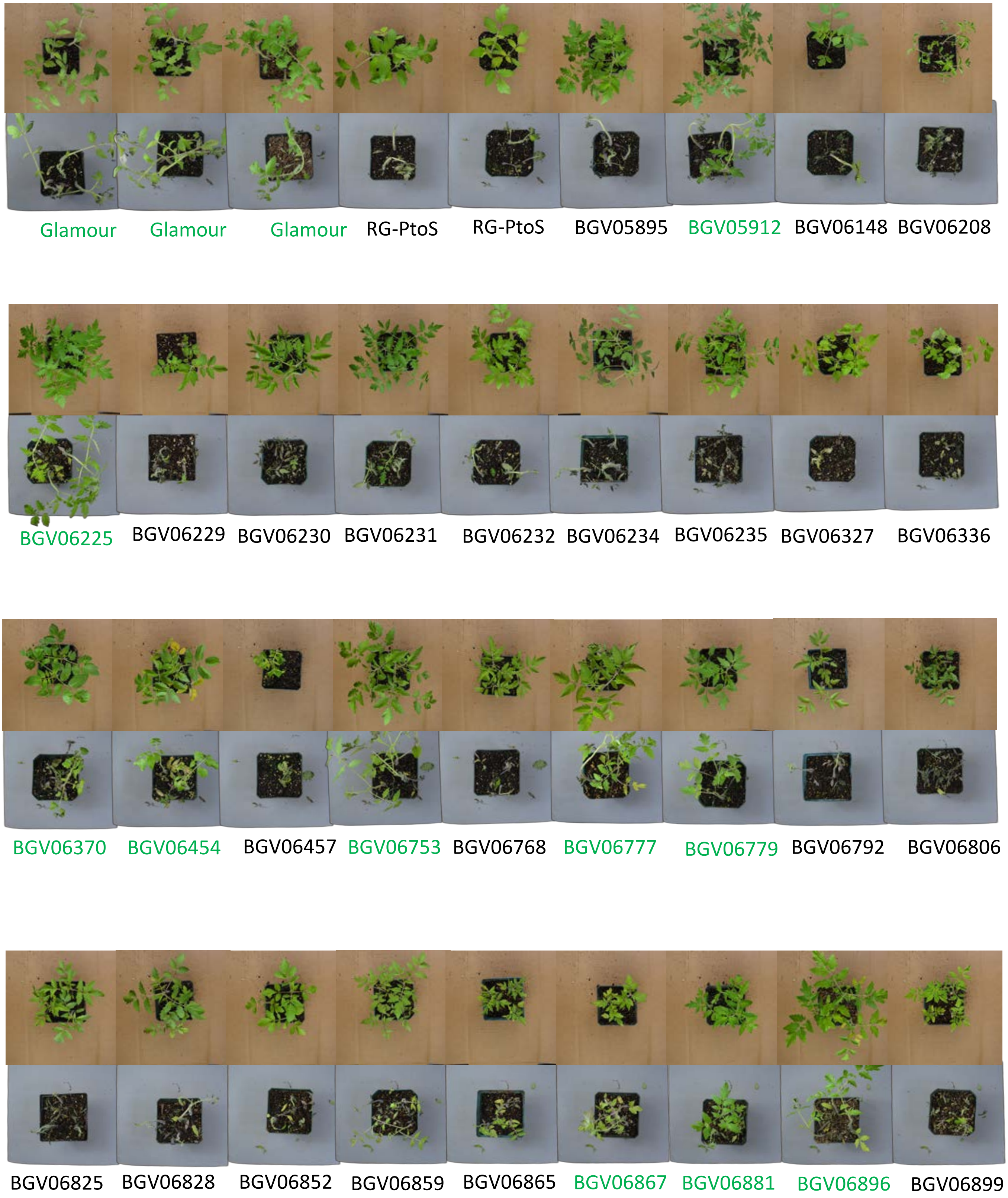
Glamour
Glamour
Glamour
RG-PtoS
RG-PtoS
BGV05895
BGV05912
BGV06148
BGV06208
BGV06225
BGV06229
BGV06230
BGV06231
BGV06232
BGV06234
BGV06235
BGV06327
BGV06336
BGV06370
BGV06454
BGV06457
BGV06753
BGV06768
BGV06777
BGV06779
BGV06792
BGV06806
BGV06825
BGV06828
BGV06852
BGV06859
BGV06865
BGV06867
BGV06881
BGV06896
BGV06899

Exp 2 :

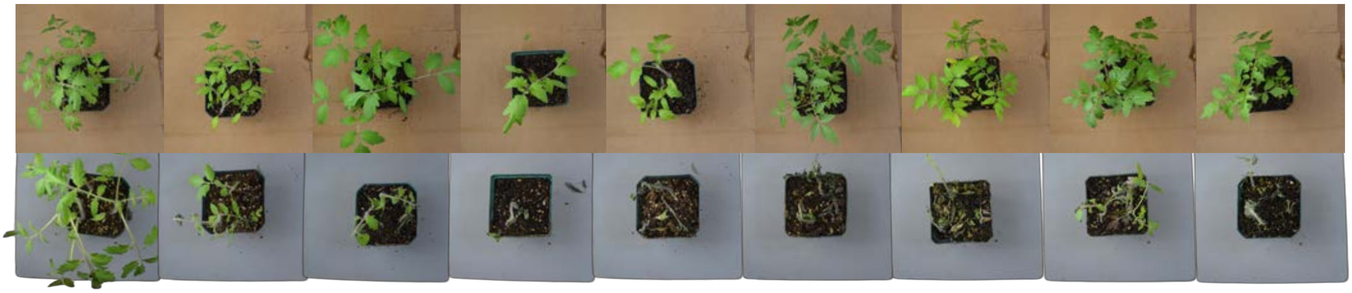

Glamour Glamour Glamour RG-PtoS RG-PtoS BGV06906 BGV06907 BGV06910 BGV06927

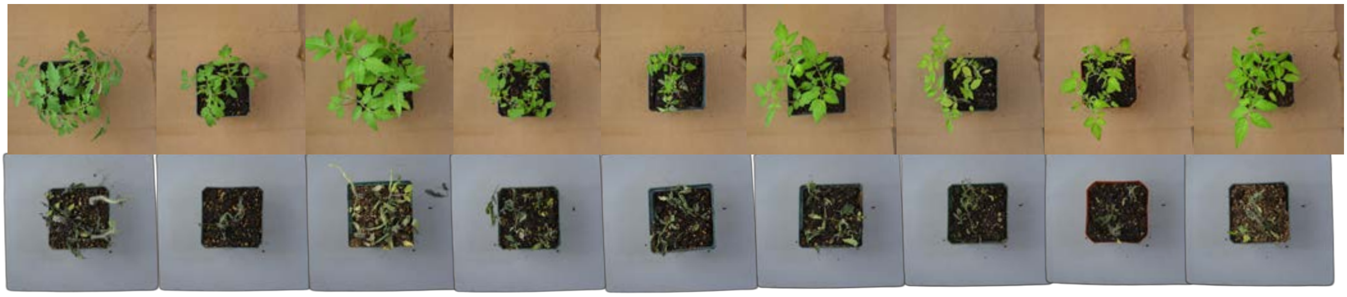

BGV06931 BGV06934 BGV07023 BGV07109 BGV07111 BGV07149 BGV07151 BGV07152 BGV07155

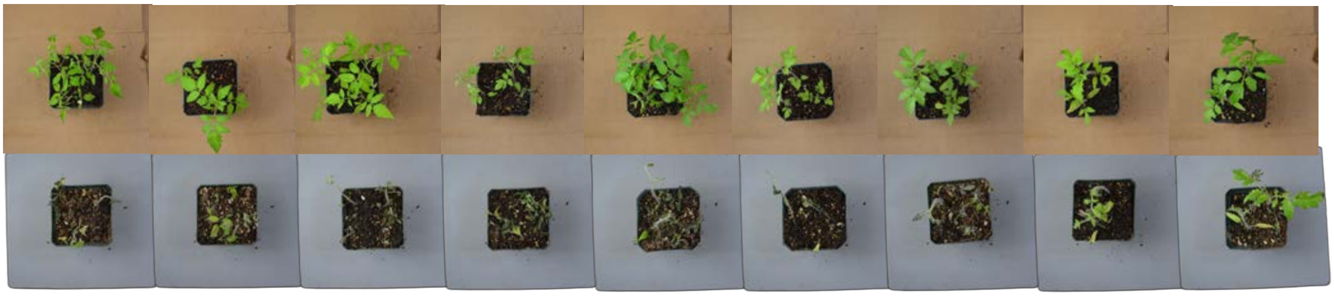

BGV07158 BGV07861 BGV07194 BGV07198 BGV07339 BGV07366 BGV07854 BGV07857 BGV07862

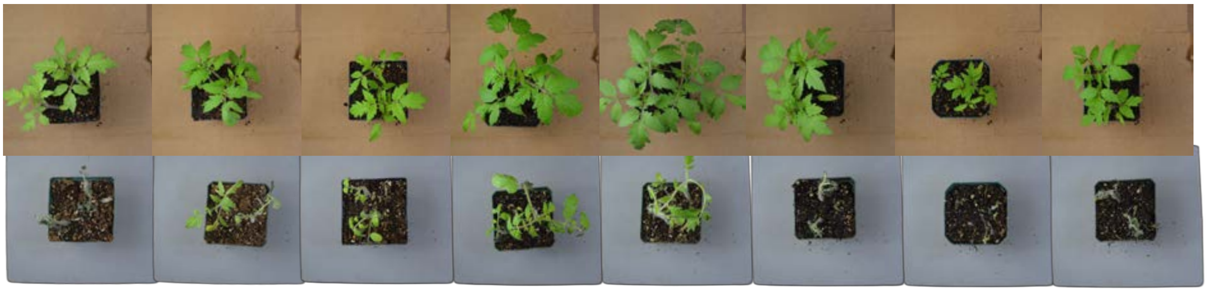

BGV07863 BGV07865 BGV07871 BGV07875 BGV07876 BGV07878 BGV07894 BGV07895

Exp 3 :

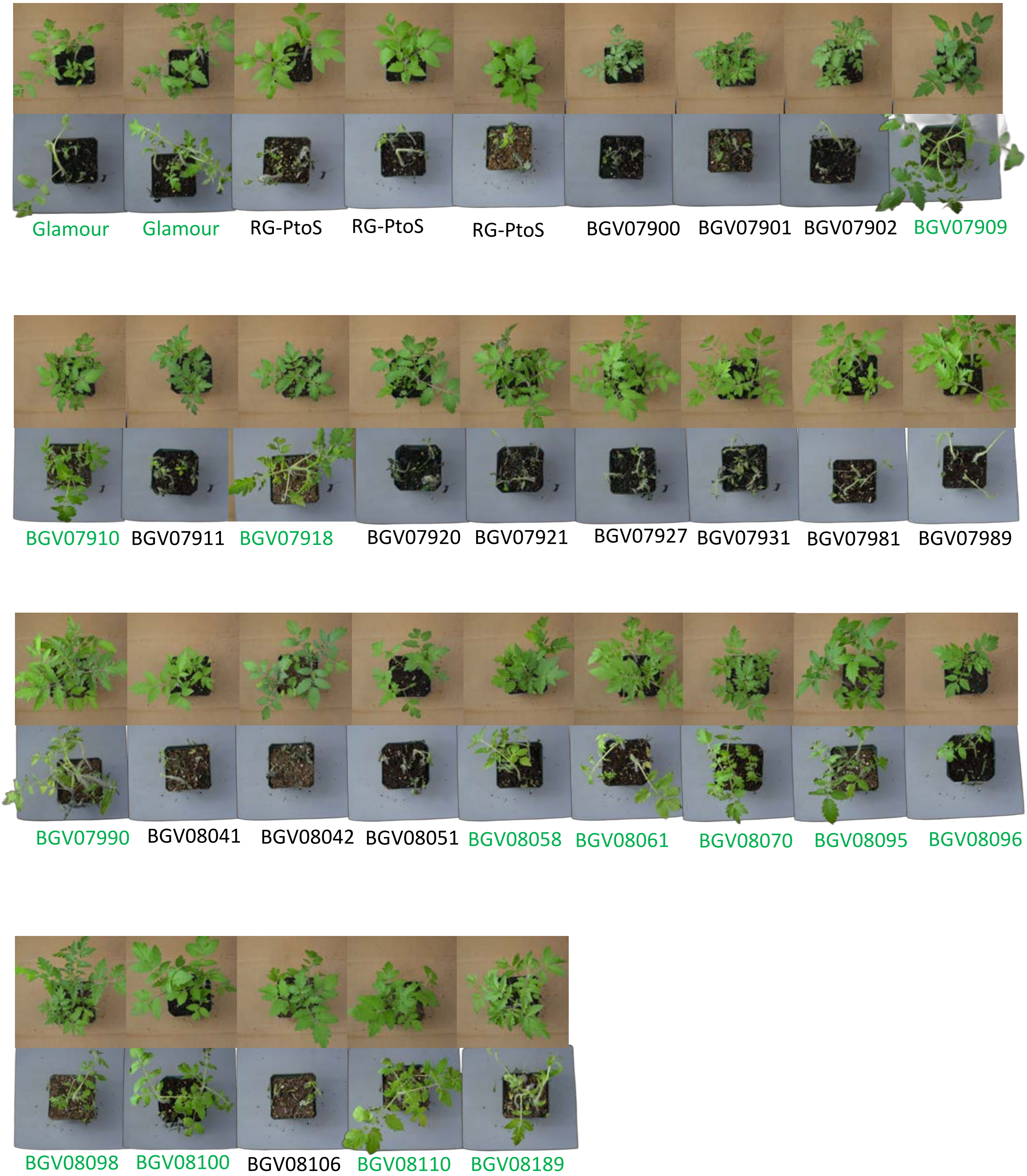
Glamour
Glamour
RG-PtoS
RG-PtoS
RG-PtoS
BGV07900
BGV07901
BGV07902
BGV07909
BGV07910
BGV07911
BGV07918
BGV07920
BGV07921
BGV07927
BGV07931
BGV07981
BGV07989
BGV07990
BGV08041
BGV08042
BGV08051
BGV08058
BGV08061
BGV08070
BGV08095
BGV08096
BGV08098
BGV08100
BGV08106
BGV08110
BGV08189

Exp 4 :

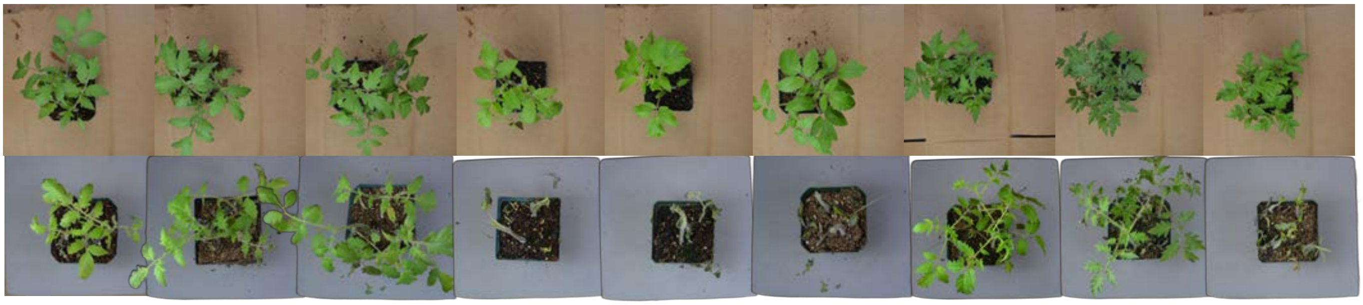

Glamour Glamour Glamour RG-PtoS RG-PtoS RG-PtoS BGV08218 BGV08219 BGV08221

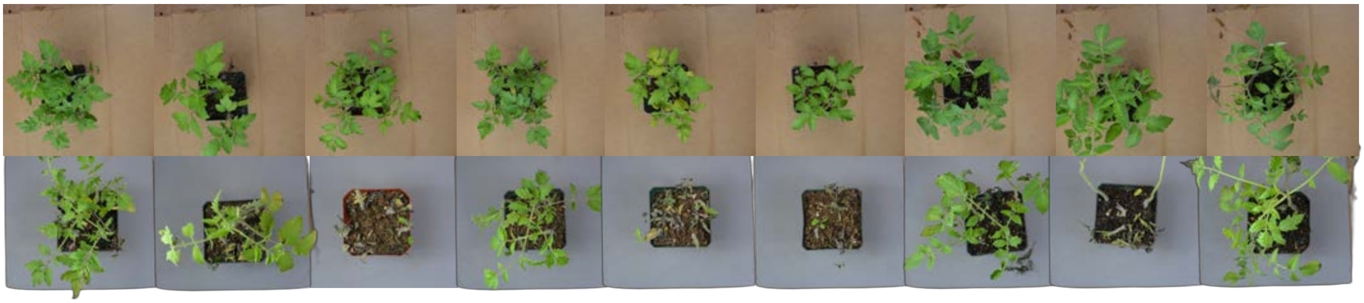

BGV08223 BGV08224 BGV08345 BGV08347 BGV08348 BGV08354 BGV12614 BGV12615 BGV12625

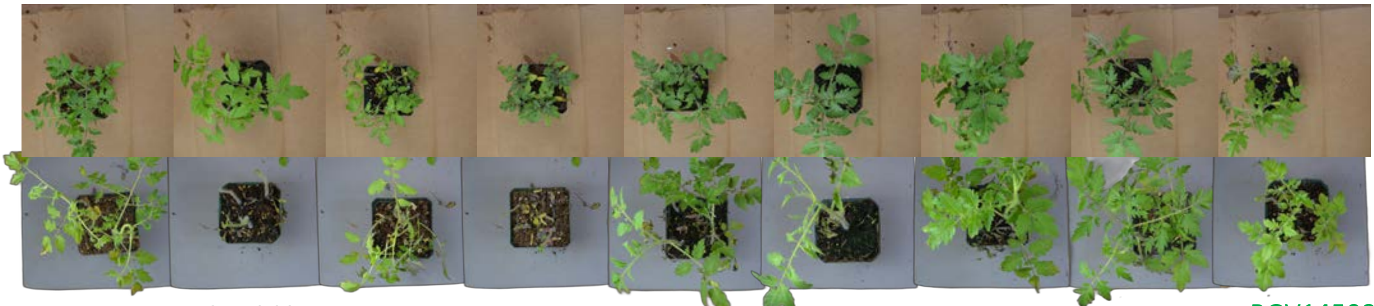

BGV12626 BGV12627 BGV12639 BGV12640 BGV13134 BGV13161 BGV13175 BGV13945 BGV14508

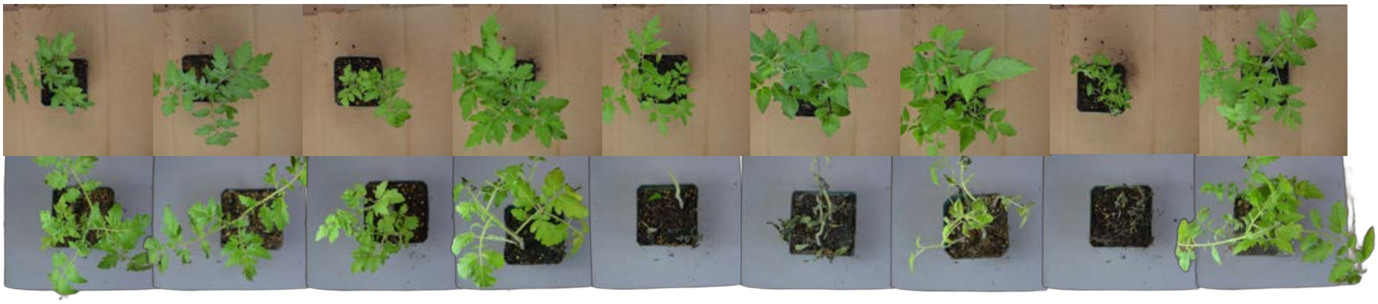

BGV14515 BGV14516 BGV14518 BGV14519 BGV14522 BGV15380 BGV15382 BGV15726 BGV15730

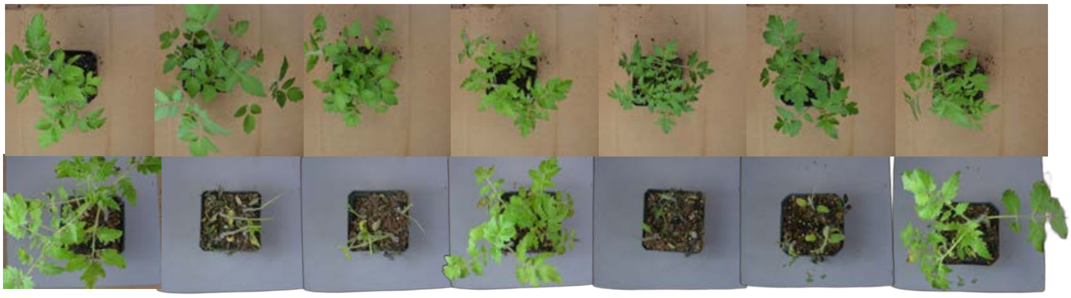

BGV15734 BGV16047 LA1712 LA2309 PI129026 PI406890 PI487625

d)

Exp 1 :

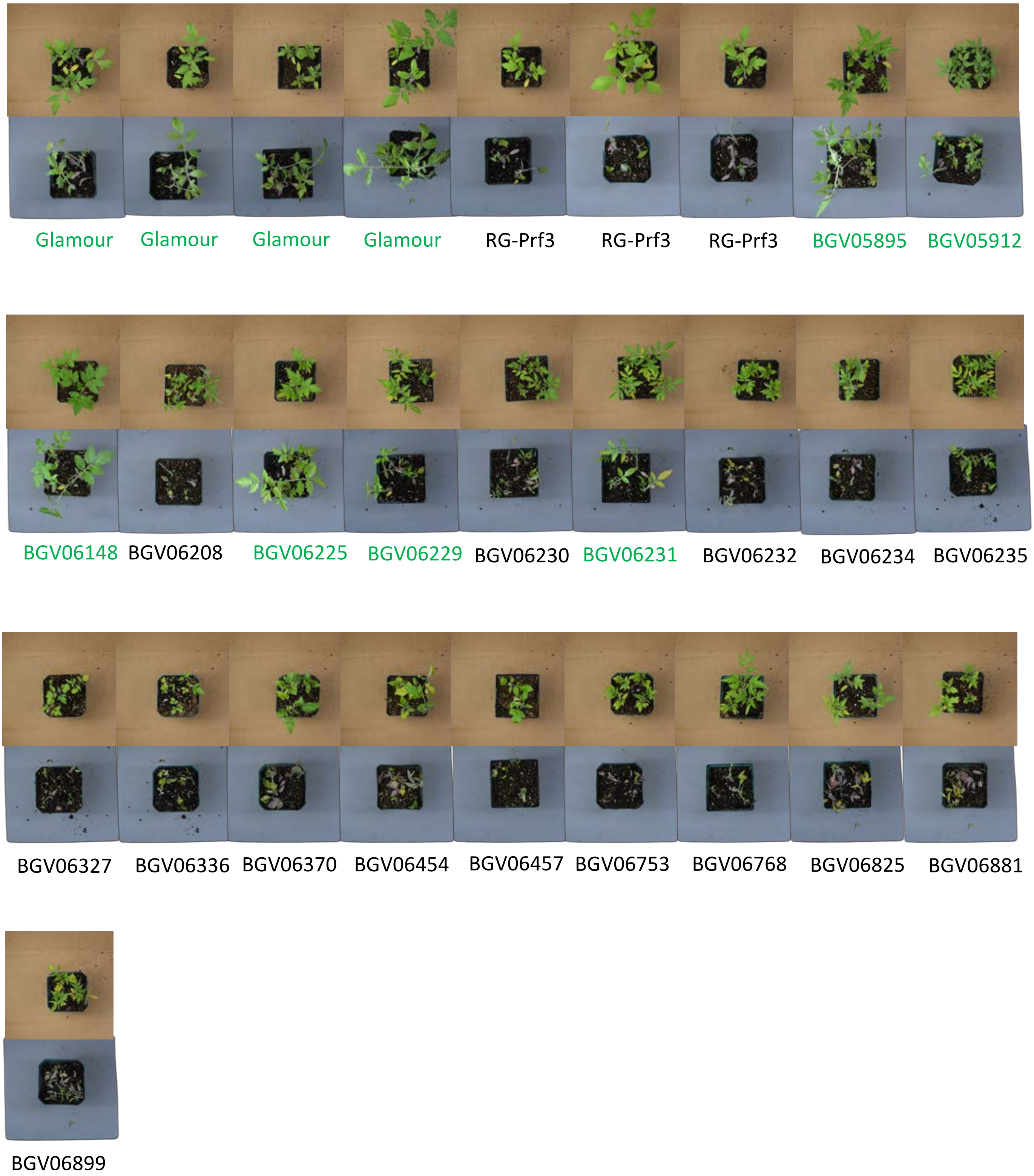
Glamour
Glamour
Glamour
Glamour
RG-Prf3
RG-Prf3
RG-Prf3
BGV05895
BGV05912
BGV06148
BGV06208
BGV06225
BGV06229
BGV06230
BGV06231
BGV06232
BGV06234
BGV06235
BGV06327
BGV06336
BGV06370
BGV06454
BGV06457
BGV06753
BGV06768
BGV06825
BGV06881
BGV06899

Exp 2 :

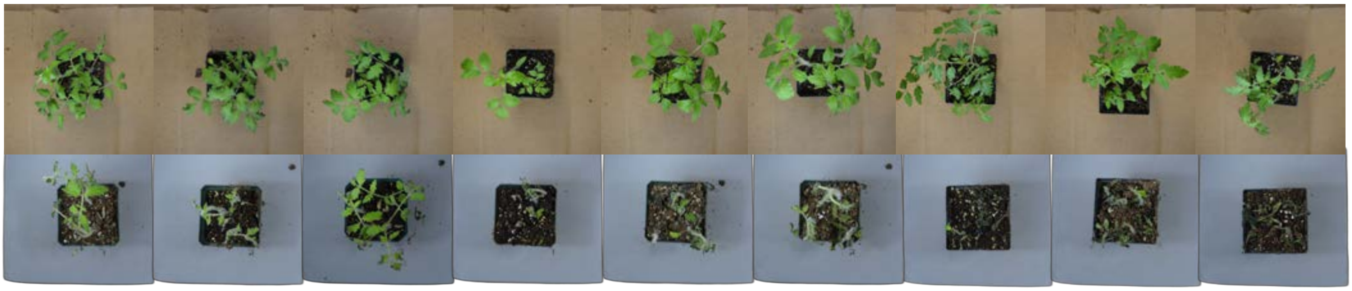

Glamour Glamour Glamour RG-Prf3 RG-Prf3 RG-Prf3 BGV06906 BGV06907 BGV06910

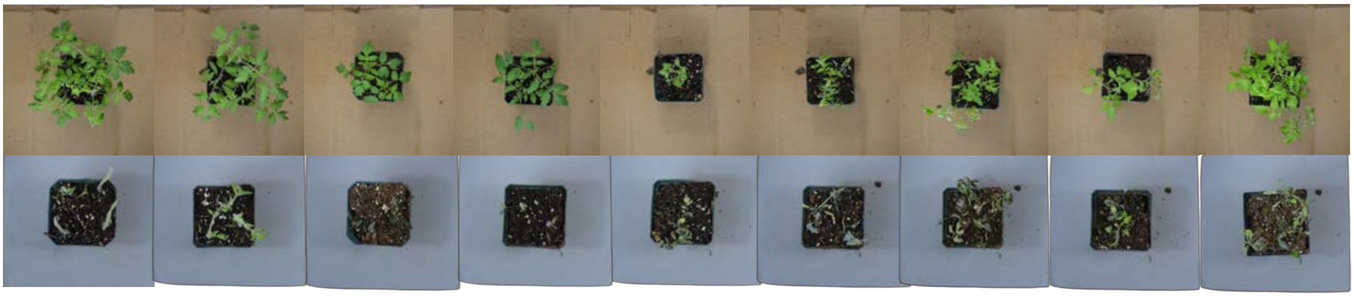

BGV06927 BGV06931 BGV06934 BGV07023 BGV07109 BGV07111 BGV07151 BGV07152 BGV07155

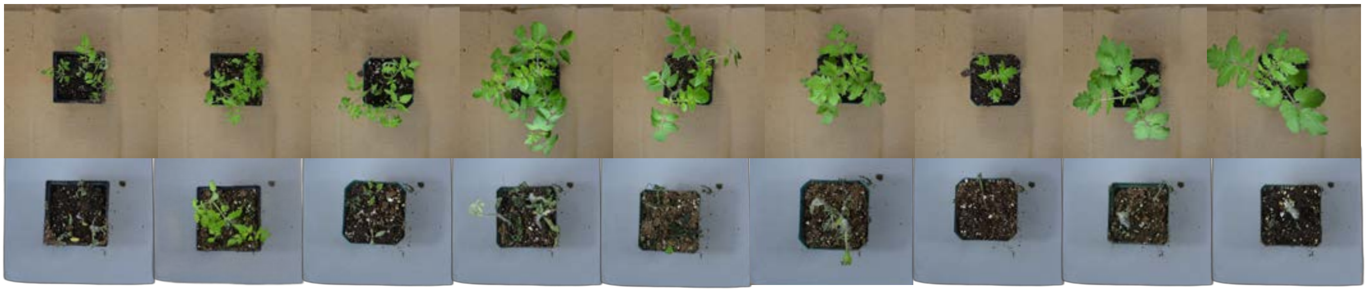

BGV07158 BGV07161 BGV07194 BGV07339 BGV07366 BGV07854 BGV07857 BGV07862 BGV07863

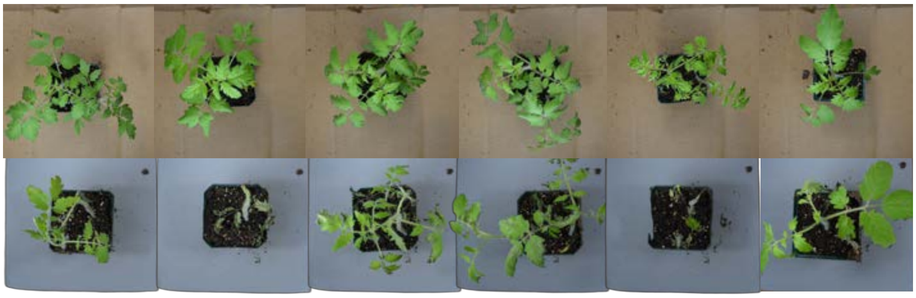

BGV07865 BGV07871 BGV07875 BGV07876 BGV07894 BGV07895

Exp 3 :

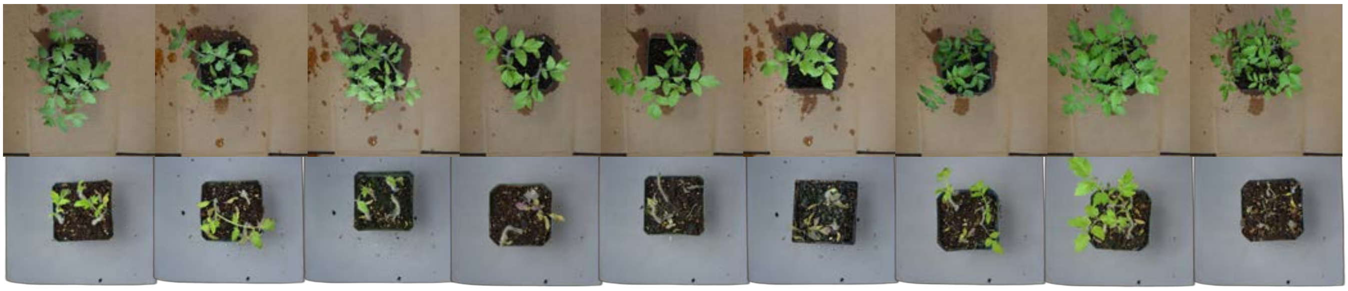

Glamour Glamour Glamour RG-PtoS RG-PtoS RG-PtoS BGV07921 BGV07927 BGV07931

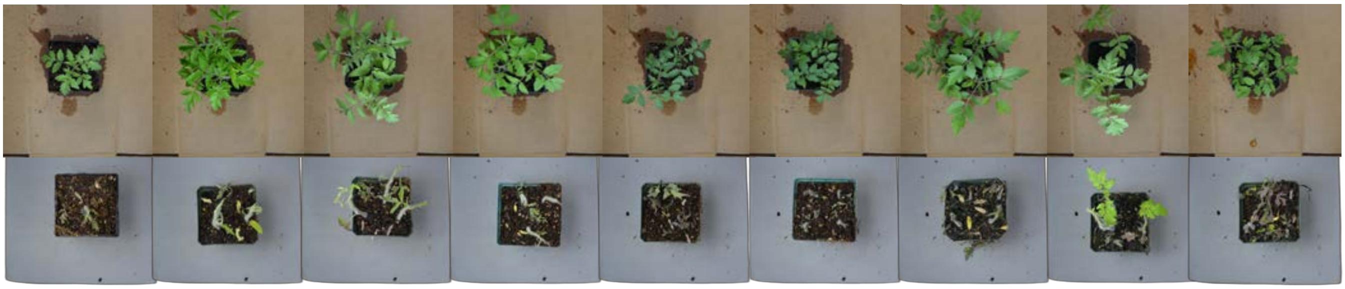

BGV07981 BGV07989 BGV07990 BGV08041 BGV08042 BGV08051 BGV08058 BGV08061 BGV08070

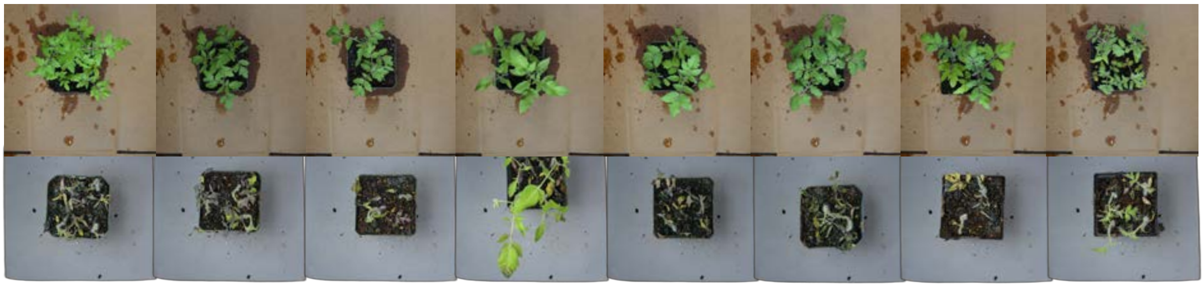

BGV08077 BGV08095 BGV08096 BGV08098 BGV08100 BGV08106 BGV08110 BGV08189

Exp 4 :

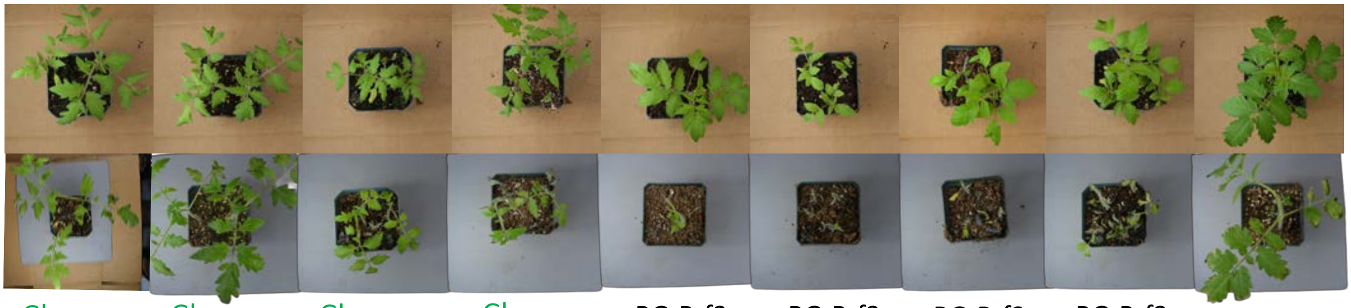

Glamour Glamour Glamour Glamour RG-Prf3 RG-Prf3 RG-Prf3 RG-Prf3 BGV08218

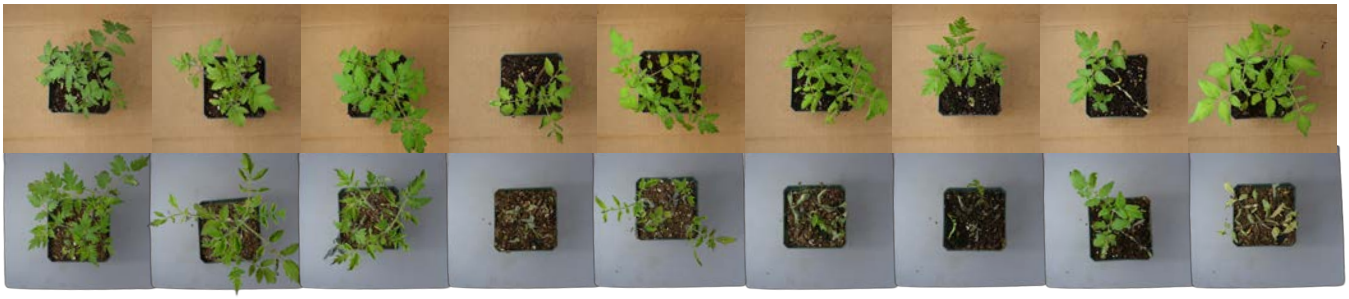

BGV08219 BGV08221 BGV08223 BGV08345 BGV08347 BGV08348 BGV08354 BGV12614 BGV12615

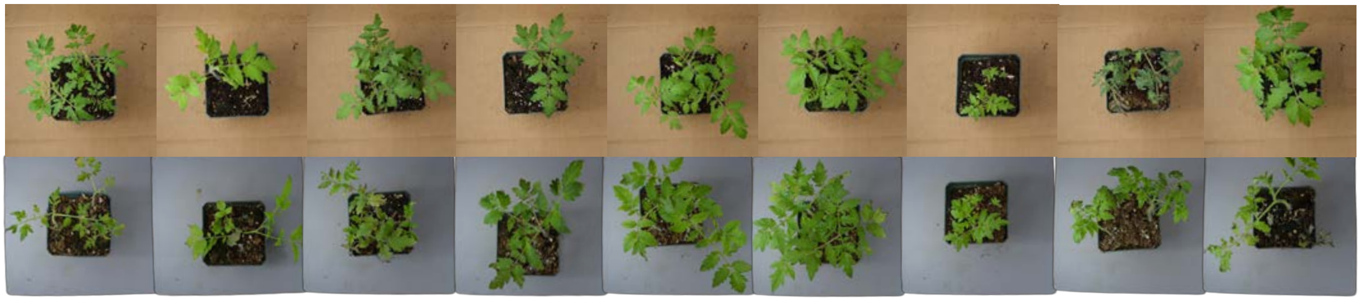

BGV12626 BGV12627 BGV12640 BGV13134 BGV13161 BGV13945 BGV14508 BGV14515 BGV14516

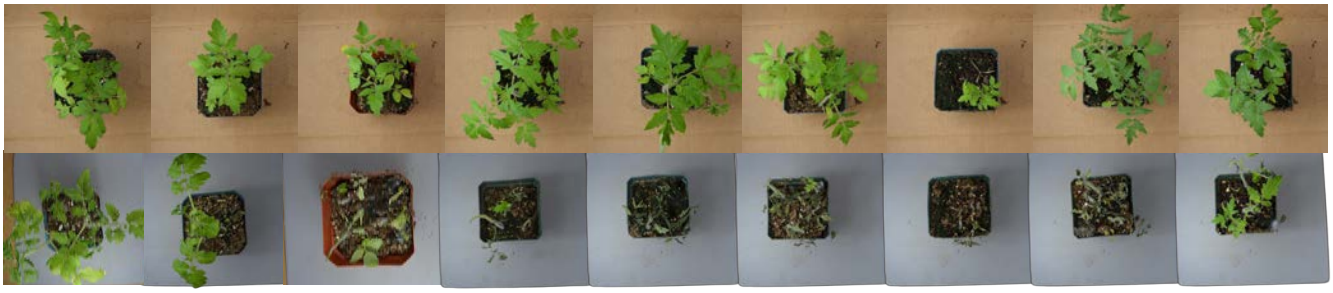

BGV14518 BGV14519 BGV15382 BGV15730 BGV15734 LA1712 LA2309 PI129026 PI406890

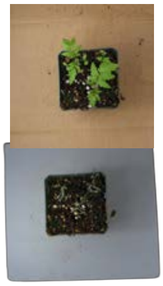

PI487625

e)

Exp 1 :

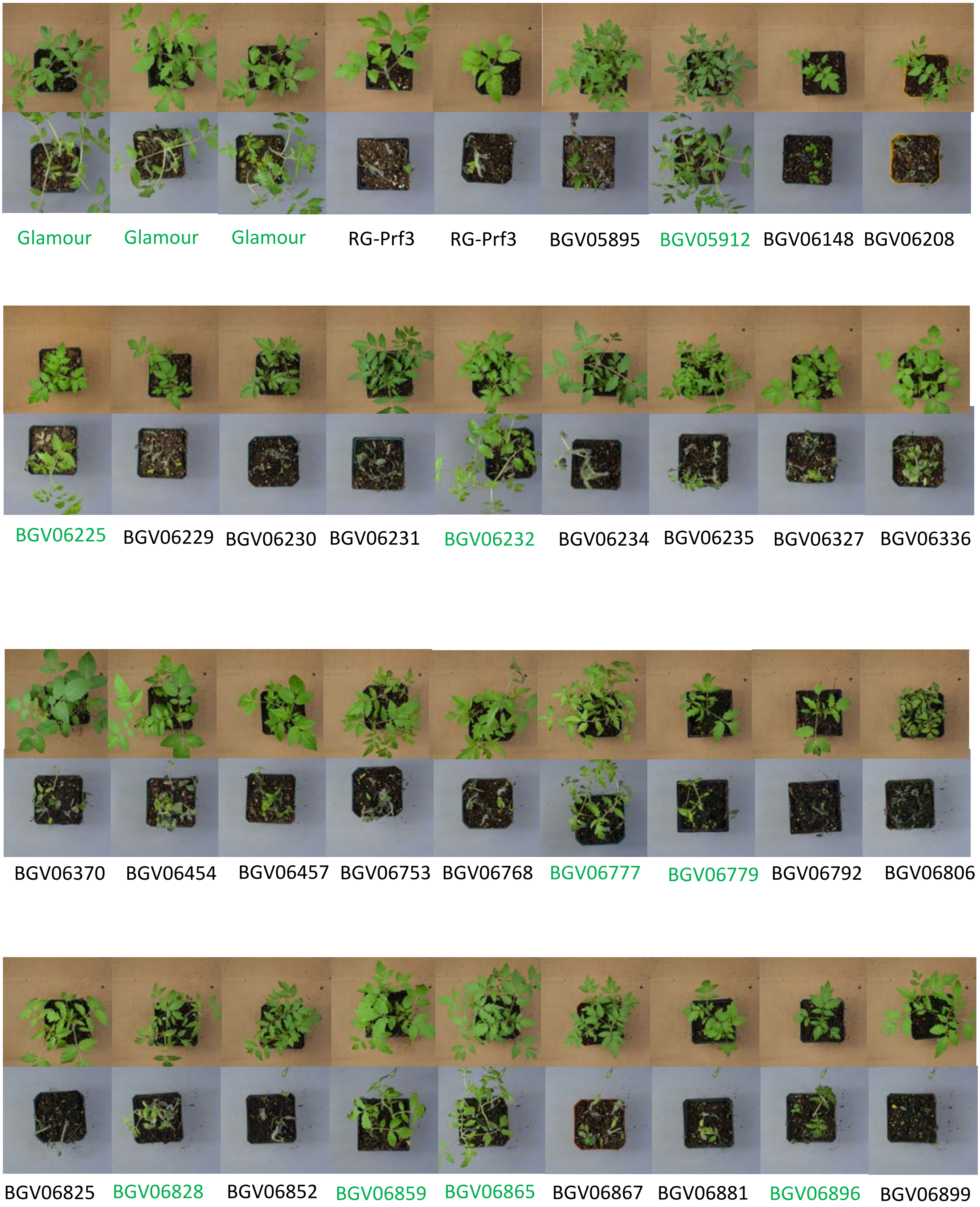

Glamour
Glamour
Glamour
RG-Prf3
RG-Prf3
BGV05895
BGV05912
BGV06148
BGV06208
BGV06225
BGV06229
BGV06230
BGV06231
BGV06232
BGV06234
BGV06235
BGV06327
BGV06336
BGV06370
BGV06454
BGV06457
BGV06753
BGV06768
BGV06777
BGV06779
BGV06792
BGV06806
BGV06825
BGV06828
BGV06852
BGV06859
BGV06865
BGV06867
BGV06881
BGV06896
BGV06899

Exp 2 :

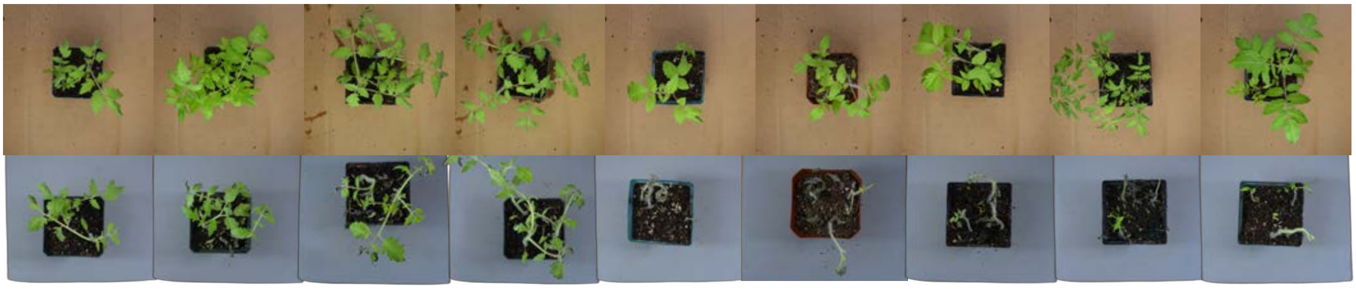

Glamour Glamour Glamour Glamour RG-Prf3 RG-Prf3 RG-Prf3 BGV06906 BGV06907

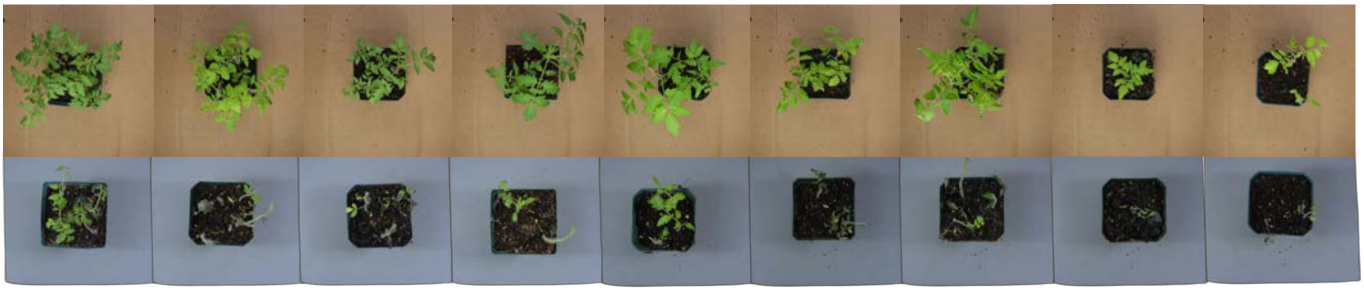

BGV06910 BGV06927 BGV06931 BGV06934 BGV07023 BGV07109 BGV07111 BGV07149 BGV07151

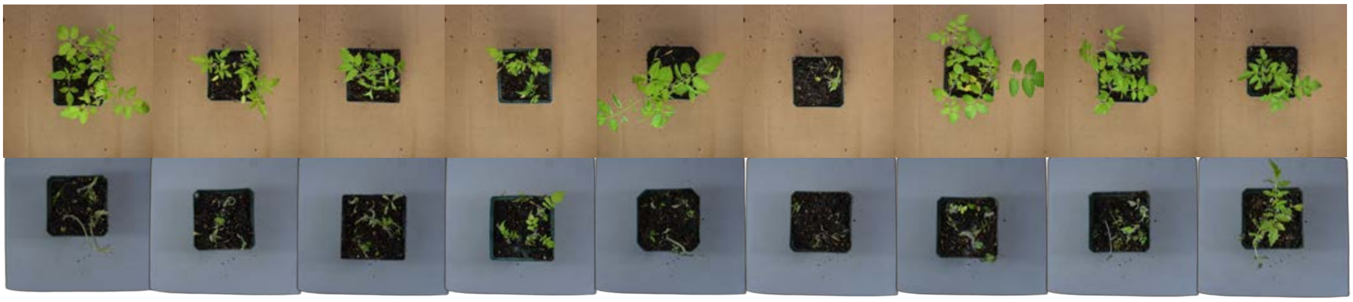

BGV07152 BGV07155 BGV07158 BGV07161 BGV07194 BGV07198 BGV07339 BGV07366 BGV07854

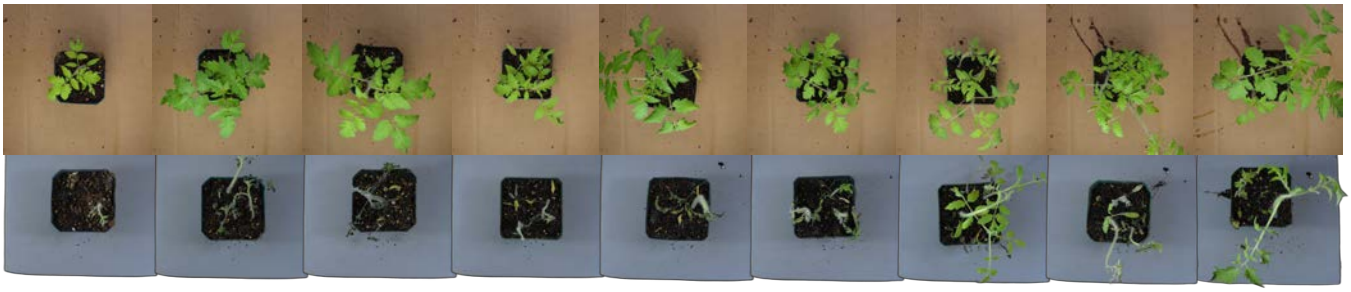

BGV07857 BGV07862 BGV07863 BGV07865 BGV07871 BGV07875 BGV07876 BGV07894 BGV07895

Exp 3 :

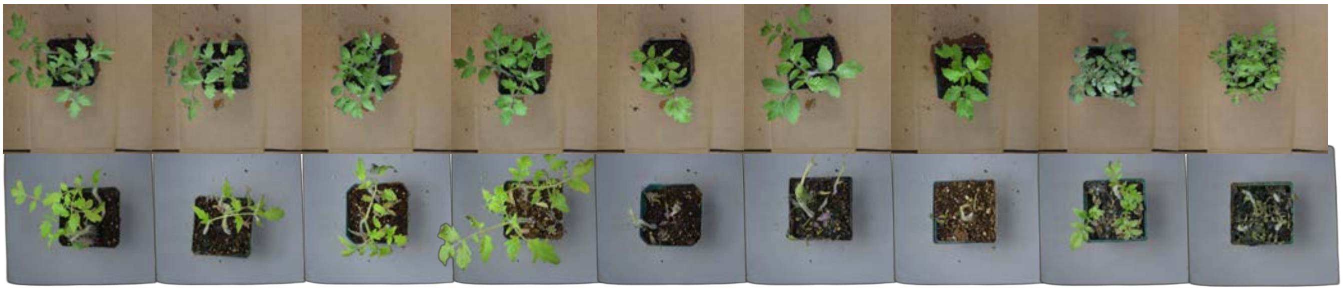

Glamour Glamour Glamour Glamour RG-Prf3 RG-Prf3 RG-Prf3 BGV07900 BGV07901

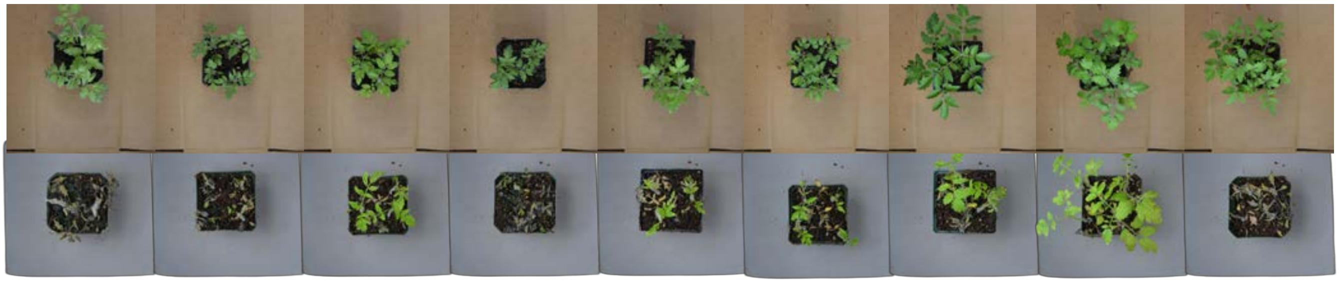

BGV07902 BGV07909 BGV07910 BGV07911 BGV07918 BGV07920 BGV07921 BGV07927 BGV07931

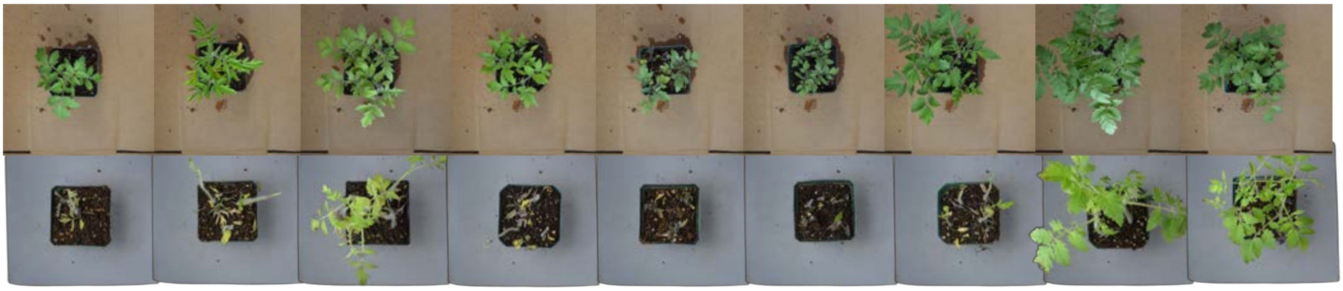

BGV07981 BGV07989 BGV07990 BGV08041 BGV08042 BGV08051 BGV08058 BGV08061 BGV08070

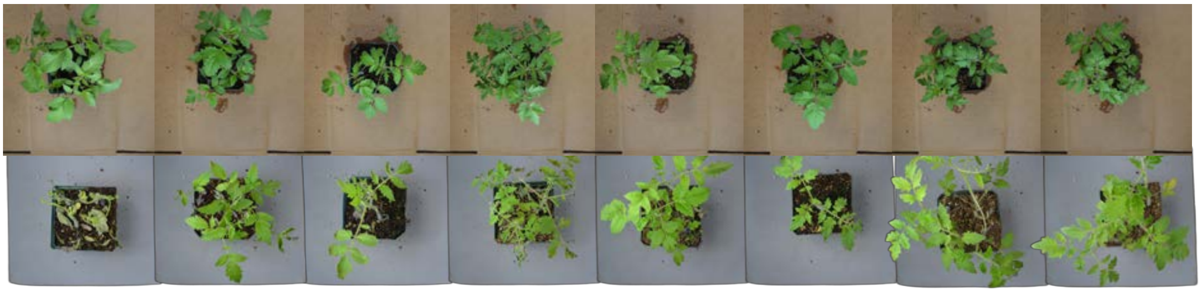

BGV08077 BGV08095 BGV08096 BGV08098 BGV08100 BGV08106 BGV08110 BGV08189

Exp 4 :

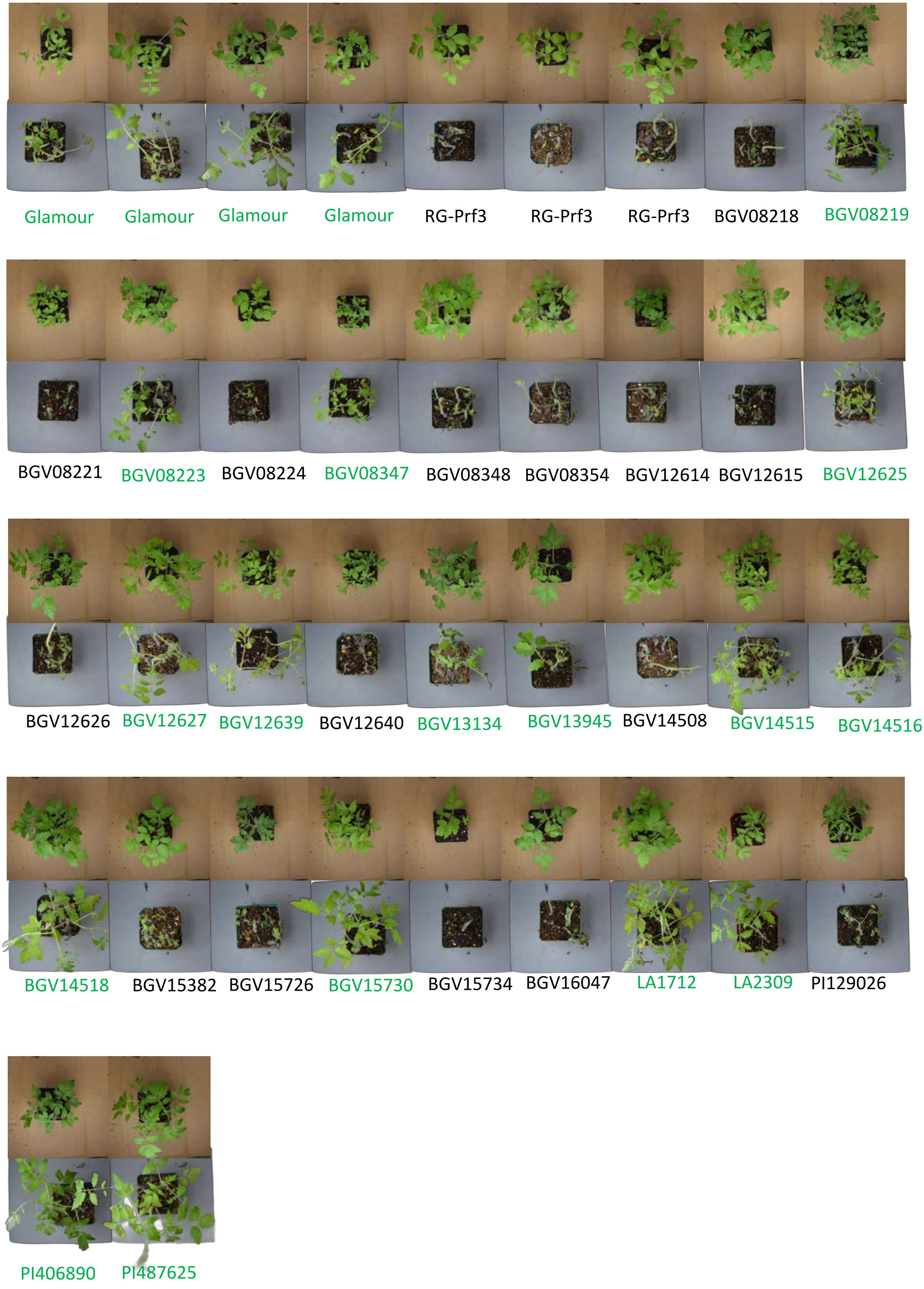

Glamour
Glamour
Glamour
Glamour
RG-Prf3
RG-Prf3
RG-Prf3
BGV08218
BGV08219
BGV08221
BGV08223
BGV08224
BGV08347
BGV08348
BGV08354
BGV12614
BGV12615
BGV12625
BGV12626
BGV12627
BGV12639
BGV12640
BGV13134
BGV13945
BGV14508
BGV14515
BGV14516
BGV14518
BGV15382
BGV15726
BGV15730
BGV15734
BGV16047
LA1712
LA2309
PI129026
PI406890
PI487625

Supplementary Figure 8. Compiled images of screening of the tomato diversity collection. Images of tomato plants spray inoculated with PtoDC3000 Δ avrPto Δ hopAB1 expressing the (a) Empty Vector, (b) HopAB1j, (c) HopAA1q, (d) HopBC1b, and (e) HopBF1a. Photos on a brown background (above) represent the plants before inoculation and photos on a grey background (below) are the same plants 7 days post-inoculation. tomato line names are presented below the images. A green label indicates that an ETI is observed. For (b), (c), (d) and (e), Glamour plants are used as control for ETI elicitation and either RG PtoS or RG Prf3 are used as a control for disease.

Supplementary Figure 9

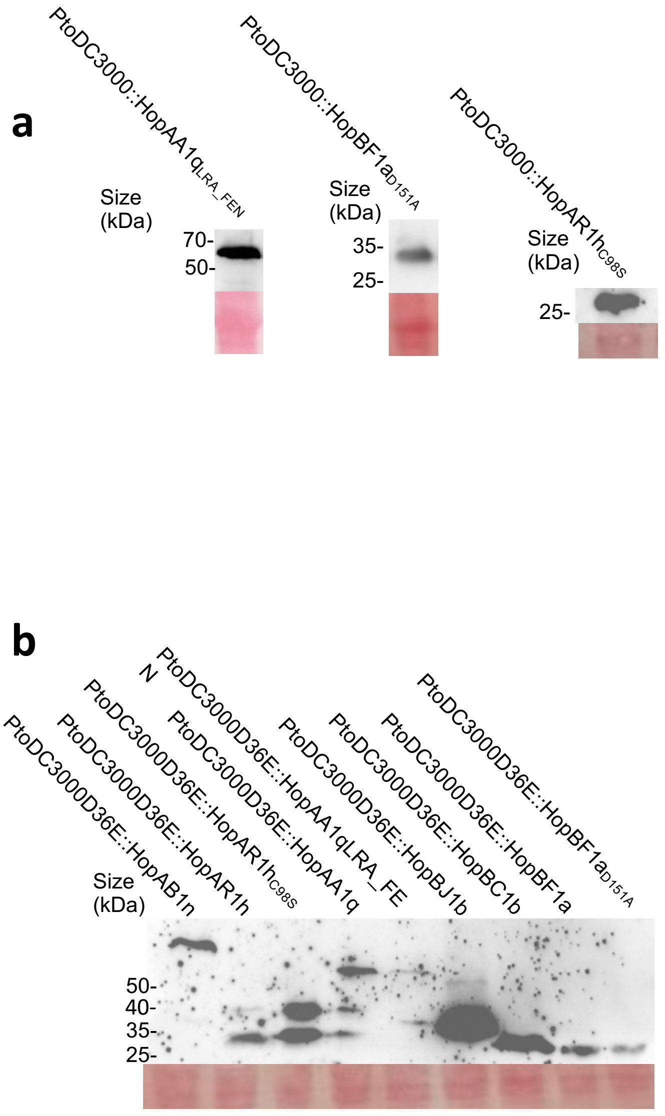

Supplementary Figure 9. Immunoblots Depicting Expression of strain generated in this study. Immunoblots against the HA tag of the three catalytic mutant representative alleles in PtoDC3000. HA tag is present on wild-type PsyTEC contructs[23] from which the mutants were derived by site-directed mutagenesis. **(a)** and the nine alleles in PtoD3000 D36E **(b)** used for HR assays following overnight growth in *hrp*-inducing minimal media. Ponceau S staining of the membrane is presented under each immunoblot. Numbers to the left of the immunoblots indicate molecular weight standards in kDa.

Supplementary Figure 10

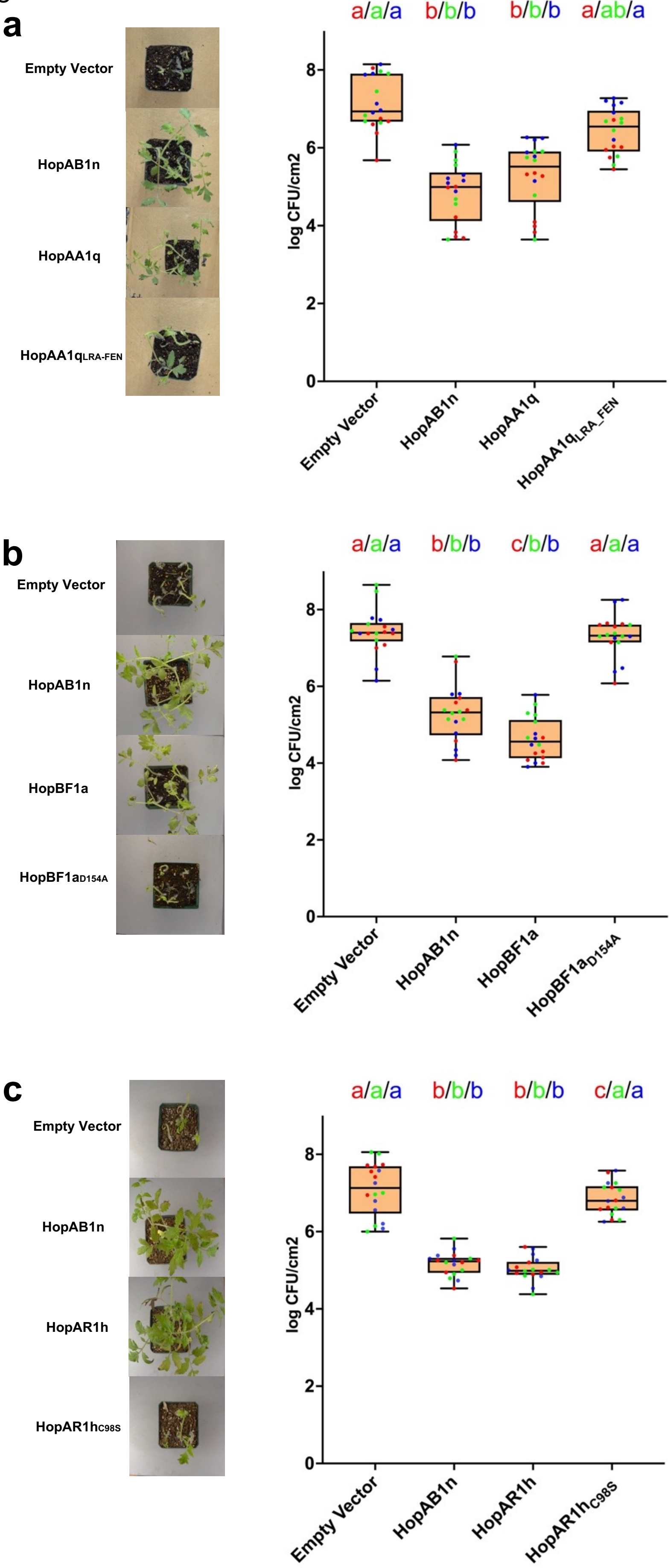

Supplementary Figure 10. Enzymatic activities of HopAA, HopAR, and HopBF are required to trigger an ETI. (a) Bacterial growth assays were conducted in tomato plants using PtoDC3000 carrying HopAA1q and a catalytic mutant $HopAA1q_{LRA\_FEN}$[23] **(b)** HopBF1a and a catalytic mutant $HopBF1a_{D154A}$, **(c)** and HopAR1h and a catalytic mutant $HopAR1h_{C98S}$. The Empty Vector strain was used as a control for disease, while the HopAB1n allele served as a control for ETI elicitation. Each dot represents a plant, with dots of the same color indicating plants from the same replicate. Box plots display pooled data from three replicates (n=6 plants per replicate) with error bars representing SEM. The boxes show the first quartile, median, and third quartile, with whiskers extending to the smallest and largest values. Letters are used to indicate groups following ANOVA *post-hoc* Tukey-test ($P < 0.05$). The color of the letter indicates the statistical group of the same color replicates. The left panels show representative pictures taken 7 days post-inoculation.

Supplementary Figure 11

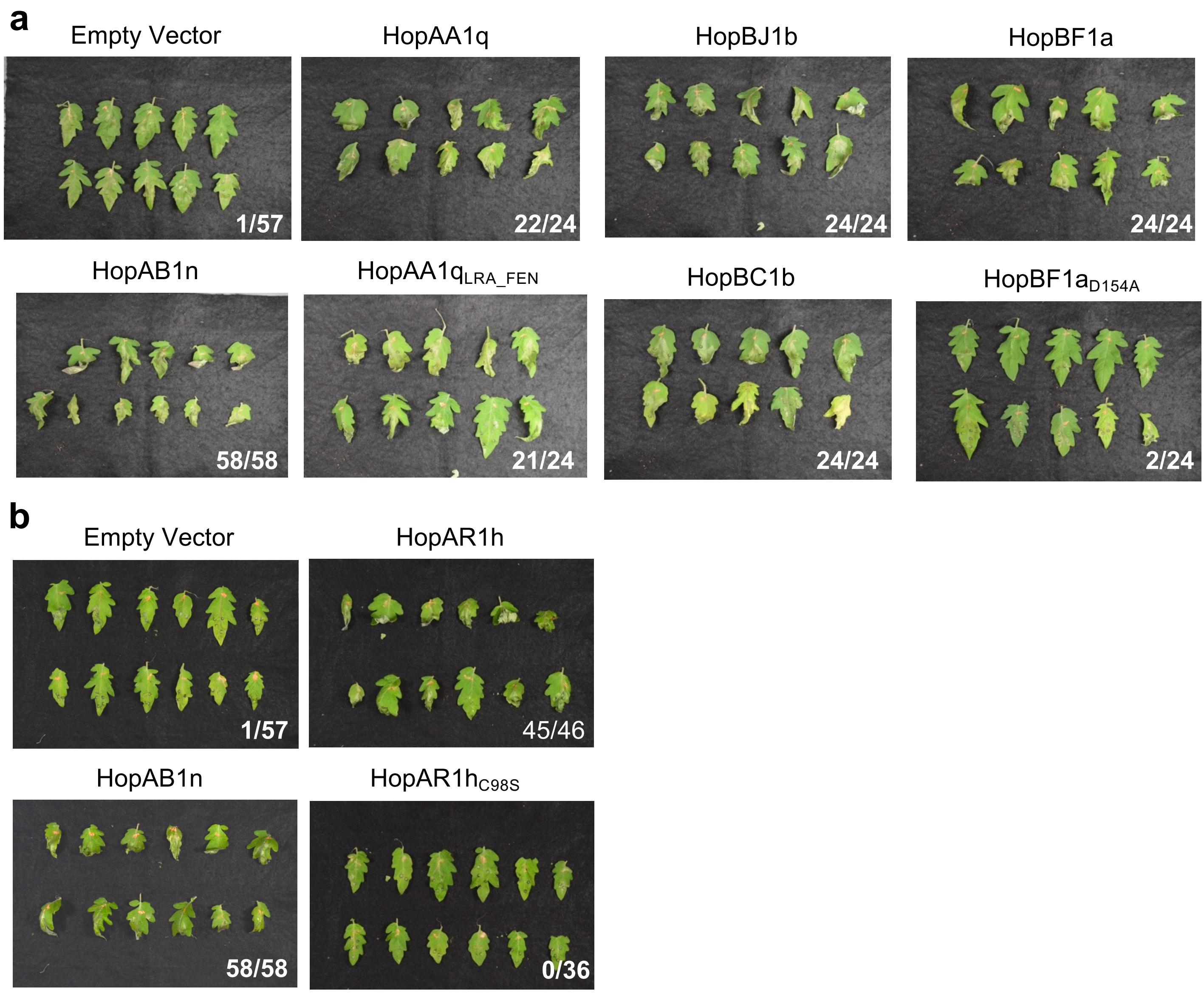

Supplementary Figure 11. HR phenotypes for newly identified ETI-eliciting effector alleles. The effectorless polymutant strain PtoDC3000D36E strain[39] carrying the Empty Vector and HopAB1n controls, the newly identified ETI-eliciting alleles HopAA1q, HopBJ1b, HopBC1b, HopBF1a and catalytic mutants $HopAA1q_{LRA\_FEN}$ and $HopBF1a_{D154A}$ (a), and HopAR1h and a catalytic mutant $HopAR1h_{C98S}$(b) were infiltrated into tomato leaves. Numbers in white embedded in the photos indicate the total number of observed macroscopic tissue collapses observed over the total number of infiltrated leaves. Pictures were taken 20-24 hours post-infiltration.

Supplementary Figure 12

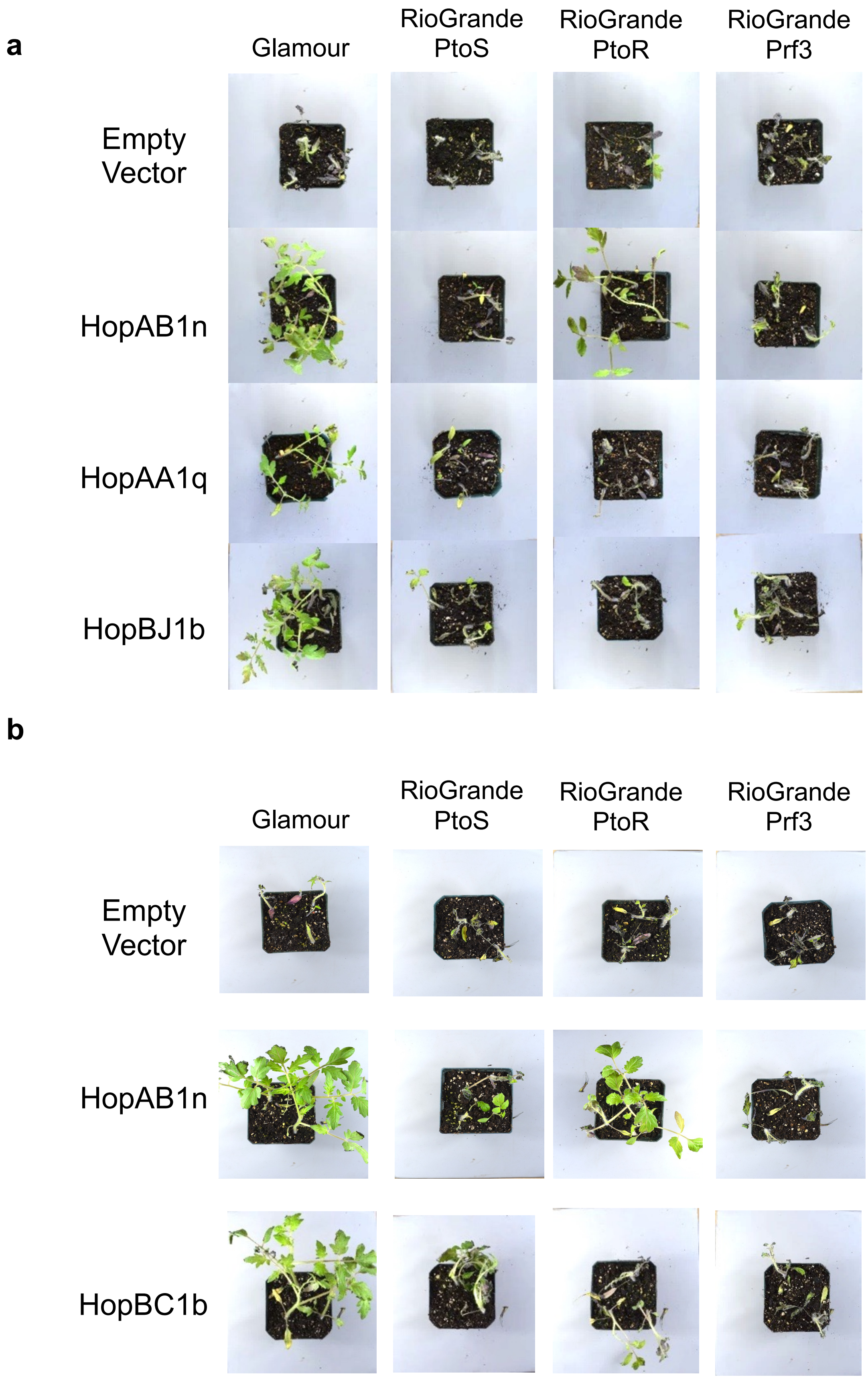

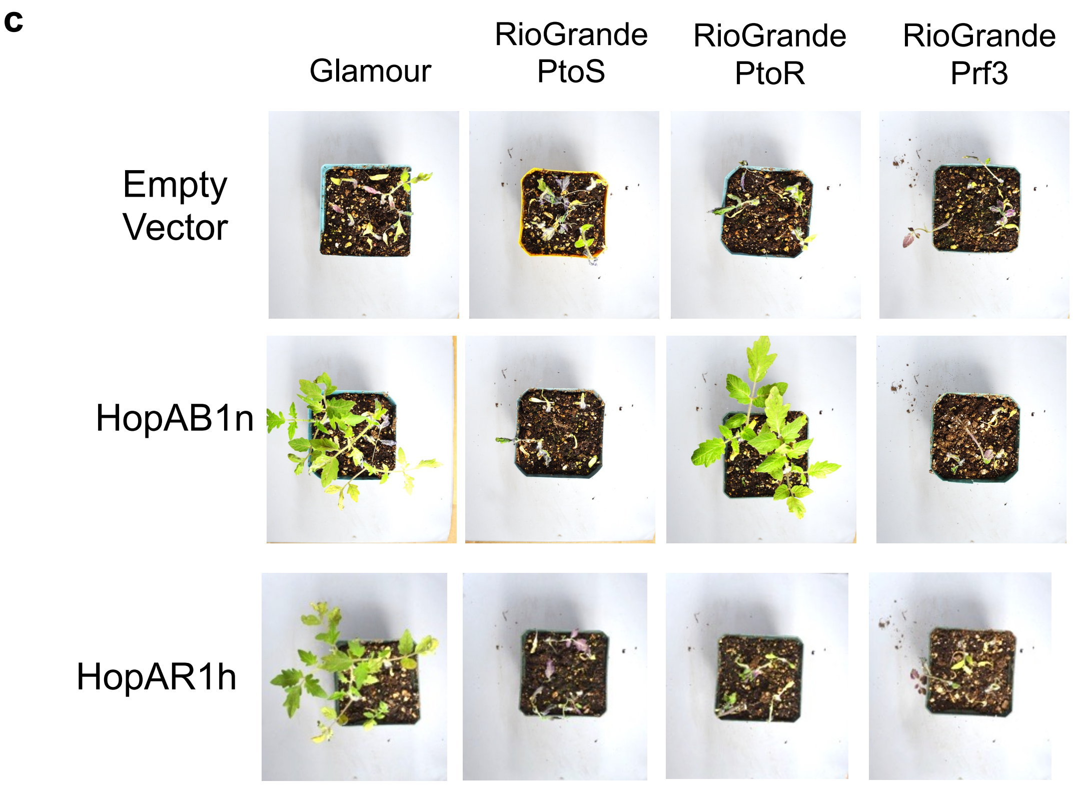
c
Glamour
RioGrande PtoS
RioGrande PtoR
RioGrande Prf3
Empty Vector
HopAB1n
HopAR1h

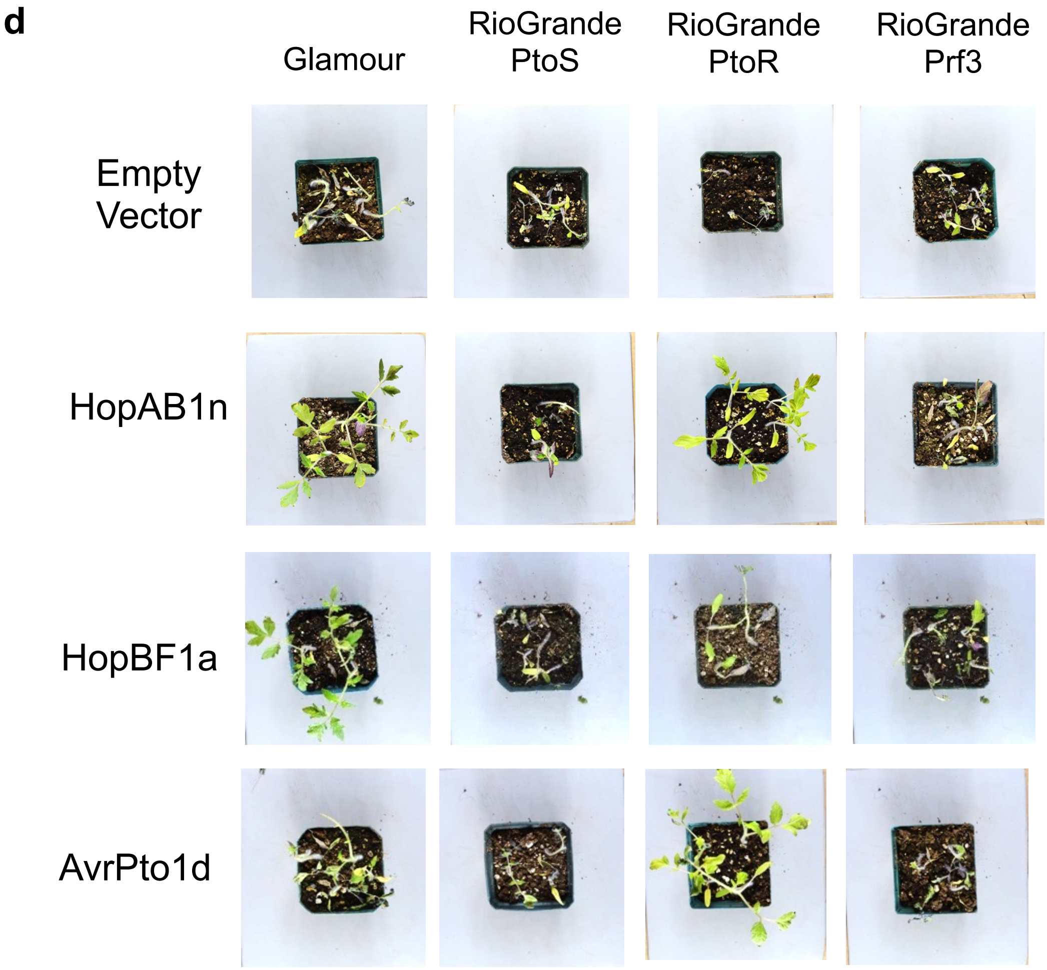
d
Glamour
RioGrande PtoS
RioGrande PtoR
RioGrande Prf3
Empty Vector
HopAB1n
HopBF1a
AvrPto1d

Supplementary Figure 12. Newly identified ETI are distinct from HopAB1 Tomato plants expressing Pto (RioGrande-PtoR) or lacking Pto (RioGrande-PtoS), RioGrande-PtoR plants with a deletion in the *Prf* gene (RioGrande Prf 3), and tomato var. Glamour plants were spray-inoculated with PtoDC3000$^{\Delta avrPto\Delta hopAB1}$ carrying HopAA1q and HopBJ1b (a), HopBC1b (b), HopAR1h (c), HopBF1a and AvrPto1d (d). PtoDC3000$^{\Delta avrPto\Delta hopAB1}$ carrying an empty vector or HopAB1n were used as controls for all the individual experiments. Pictures were taken seven days post-inoculation. The experiments were repeated at least twice with similar results

Supplementary Figure 13

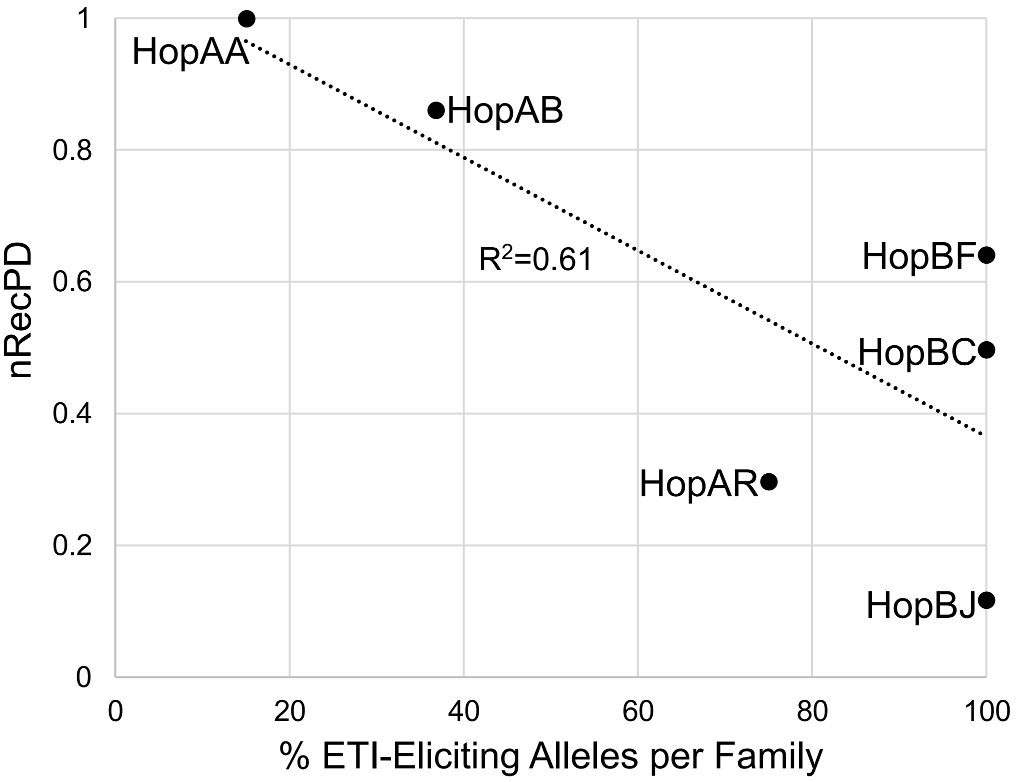

Supplementary Figure 13. Association between nRecPD and percent of ETI-eliciting alleles per family. nRecPD compares individual gene genealogies to the core genome phylogeny to quantify the relative importance of non-vertical inheritance in the evolution history of a gene[41]. nRecPD values range from 0.0 to 1.0, with the former reflecting genes evolving primarily through horizontal evolutionary process, and the latter reflecting genes evolving primarily through vertical descent[41].

Supplementary Figure 14

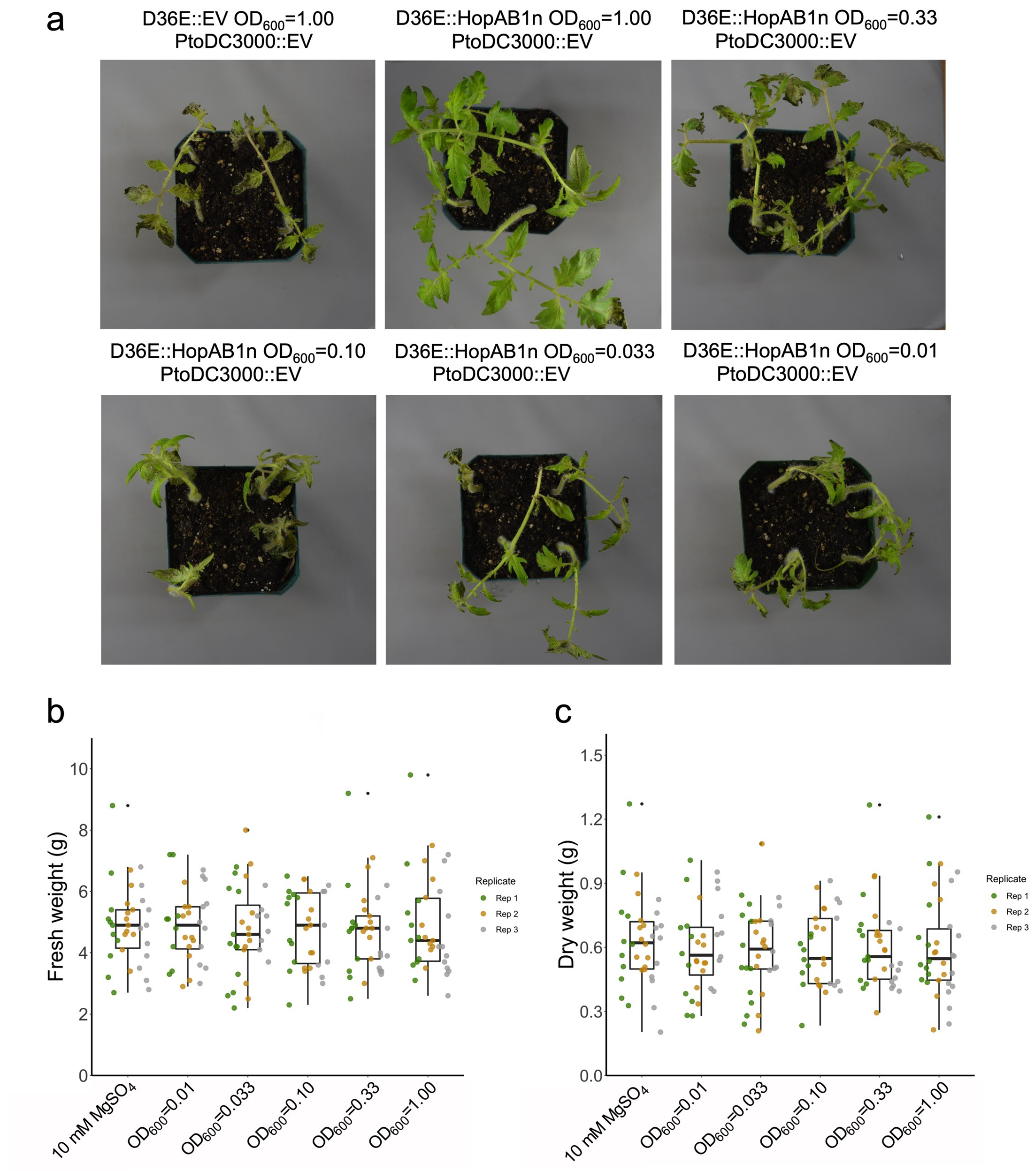

Supplementary Figure 14 . HopAB1n-mediated ETI immunoprotection in tomatoes is dose-dependent with no significant plant growth detriment. (a) Four-week-old tomato cv. Glamour plants were co-spray-inoculated with the effectorless polymutant PtoDC3000D36E (D36E) carrying an empty vector (EV) or the effector HopAB1n at different Optical Densities (OD) at 600 nm (1.00, 0.33, 0.10, 0.033, and 0.01), along with the PtoDC3000 strain. Pictures were taken seven days post-inoculation and are representative of the results of three independent experiments (b, c) Tomato cv. Glamour plants were spray-inoculated with 10 mM $MgSO_4$ or D36E carrying the effector HopAB1n at OD_{600}=1.00, OD_{600}=0.33, OD_{600}=0.10, OD_{600}=0.033 or OD_{600}=0.01. Above-ground plant fresh weight (b) and dry weight (c) were measured three weeks post-inoculation. Each dot represents the weight of one plant. Three experimental replicates are represented in the boxplot graphs(For fresh weight (a), n = 11, 9, 12, 11, 11, 11 individual plants for replicate 1; n = 12, 12, 12, 12, 12, 11 individual plants for replicate 2; n = 11, 11, 8, 8, 10, 12 individual plants for replicate 3. For dry weight (b), n= 11, 11, 12, 9, 11, 11 individual plants for replicate 1; n = 12, 11, 12, 12, 12, 11 individual plants for replicate 2; n = 11, 10, 8, 8, 10, 12 individual plants for replicate 3). The boxes show the first quartile, median, and third quartile, with whiskers extending to the smallest and largest values. No statistically significant differences were observed between treatments for individual replicate by ANOVA Tukey's *post hoc* test.

Supplementary Figure 15

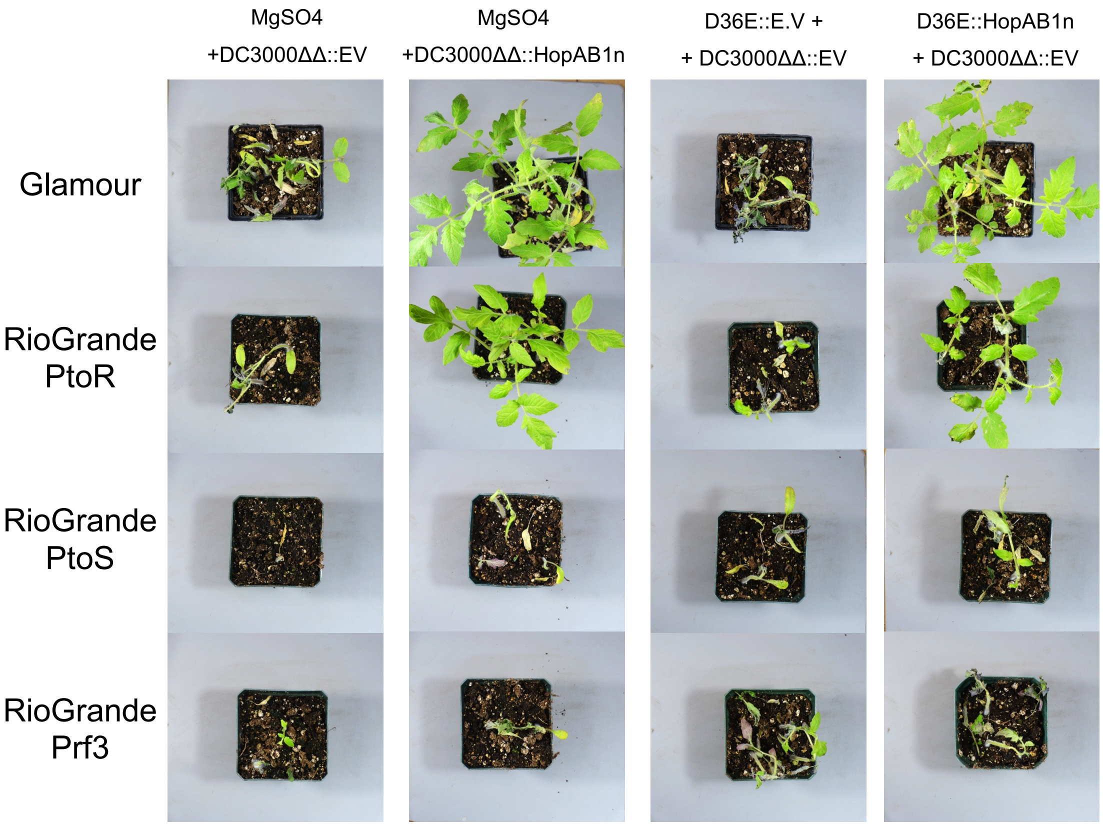

Supplementary Figure 15. The *Pto/Prf* locus is essential for HopAB1n-triggered protection. Tomato plants expressing Pto (RioGrande-PtoR) or lacking Pto (RioGrande-PtoS), Rio Grande-PtoR plants with a deletion in the *Prf* gene (RioGrande Prf 3), and tomato Glamour plants were first spray-inoculated with the effectorless polymutant PtoDC3000D36E[39] (D36E) carrying an empty vector or the effector HopAB1n, or with $MgSO_4$. Twenty-four hours later, they were inoculated with PtoDC3000$^{\Delta avrPto\Delta hopAB}$ (PtoDC3000ΔΔ) carrying an empty vector or HopAB1n. Pictures were taken seven days post-inoculation. The experiments were repeated twice with similar results.
