# Supplementary material for: The effector-triggered immunity landscape of tomato against Pseudomonas syringae
Source: Nat Commun. 2024 Jun 14;15:5102. doi: 10.1038/s41467-024-49425-4 (PMC11178782; doi:10.1038/s41467-024-49425-4)
Supplement: Supplementary file 3 — Description of Additional Supplementary Files [file 41467_2024_49425_MOESM3_ESM.pdf]

Description of Additional Supplementary Files:

Supplementary Data 1: Summary of primary ETI screening in tomato cv. Glamour.

Supplementary Data 2: Summary of primary ETI screening in Wild tomato species.
